# TSPYL5 Promotes Triple‐Negative Breast Cancer Metastasis by Antagonizing USP10‐Mediated PTEN Stabilization to Unleash a ZEB1‐Dependent EMT Program

**DOI:** 10.1002/advs.202520273

**Published:** 2026-06-04

**Authors:** Jiaying Shi, Ming Yi, Shengyu Xie, Zhaokun Wang, Xinyue Zhang, Yangwei Zhang, Rui Tang, Yuan Yang, Yunqiang Liu

**Affiliations:** ^1^ Department of Medical Genetics State Key Laboratory of Biotherapy West China Hospital, Sichuan University Chengdu China; ^2^ Department of Rehabilitation Medicine Xuanwu Hospital, Capital Medical University Beijing China; ^3^ Frontiers Science Center for Disease‐related Molecular Network State Key Laboratory of Biotherapy West China Hospital Sichuan University Chengdu China

**Keywords:** epithelial‐mesenchymal transition, metastasis, PTEN, triple‐negative breast cancer, TSPYL5

## Abstract

Hyperactivation of the PI3K/AKT pathway is a hallmark of metastatic triple‐negative breast cancer (TNBC), but its drivers in TNBC retaining wild‐type PTEN are poorly understood. Here, we identify Testis‐Specific Y‐Like Protein 5 (TSPYL5) ‐the top metastasis‐associated gene from an unbiased bioinformatics screen‐ as a master regulator that resolves this paradox. Clinically, *TSPYL5* amplification and overexpression are robust predictors of metastatic progression and poor prognosis. High‐resolution single‐cell and spatial analyses reveal that *TSPYL5* defines a malignant subpopulation with stem‐like, genomically unstable, and pro‐metastatic properties. Functionally, TSPYL5 is sufficient to drive spontaneous polymetastasis from orthotopic tumors and is indispensable for post‐intravasation colonization, culminating in overt liver metastases in 60% of animals—a phenotype absent in controls. Mechanistically, TSPYL5 sequesters the deubiquitinase USP10, thereby preventing it from stabilizing the tumor suppressor PTEN. This TSPYL5‐USP10 interaction triggers the proteasomal degradation of PTEN, circumventing its wild‐type status to hyperactivate PI3K/AKT signaling and unleash a ZEB1‐driven metastatic program. This study delineates a complete TSPYL5‐USP10‐PTEN axis, providing a new paradigm for the post‐translational tumor suppressor inactivation in TNBC. Our work validates TSPYL5 as a biomarker for PI3K pathway dependency and establishes the TSPYL5‐USP10 interface as a tractable therapeutic target to restore PTEN function and combat metastatic TNBC.

## Graphical abstract

The hyperactivation of PI3K/AKT signaling in PTEN wild‐type triple‐negative breast cancer represents a clinical paradox. We delineate a novel post‐translational regulatory axis wherein the oncogene TSPYL5 competitively antagonizes the deubiquitinase USP10. This sequesters USP10 from PTEN, triggering proteasomal degradation of the tumor suppressor, unleashing a ZEB1‐dependent metastatic program and revealing a druggable protein‐protein interface.

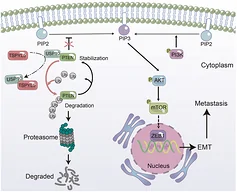

## 1 Introduction

Breast cancer (BRCA) is the leading cause of cancer incidence in women worldwide, with its lethality primarily driven by the development of distant metastases [1]. A significant fraction of patients with early‐stage disease eventually relapse with incurable metastatic tumors, often years after initial therapy [2]. This highlights the profound clinical challenge of disseminated tumor cells that can survive in a dormant state before colonizing vital organs, leading to organ failure and death [3]. Therefore, elucidating the molecular mechanisms that govern the entire metastatic cascade is critical for developing therapies that can prevent or eradicate metastatic disease.

This challenge is particularly acute in triple‐negative breast cancer (TNBC), an aggressive subtype accounting for 10%–20% of BRCA cases [4, 5, 6, 7, 8, 9, 10]. Defined by the absence of targetable hormone and HER2 receptors, TNBC treatment has historically relied on conventional chemotherapy, which is frequently undermined by resistance [11, 12, 13, 14]. While recent targeted agents have offered modest benefits to specific subsets, the therapeutic options for most patients remain limited, and the 5‐year survival for metastatic TNBC is ∼12% [15]. The profound heterogeneity of TNBC suggests that targeting single signaling pathways is unlikely to be broadly effective. Instead, a more promising strategy is to identify and disable “master regulators” that coordinate multiple oncogenic programs to drive metastasis [16].

Two interconnected biological processes are central to TNBC progression: aberrant signal transduction and pro‐metastatic cellular reprogramming. The PI3K/AKT pathway is one of the most frequently hyperactivated signaling axes in TNBC, yet a long‐standing paradox exists: this hyperactivation often occurs in tumors that retain wild‐type *PTEN*, the pathway's critical negative regulator [12, 17, 18, 19]. This points to non‐genetic mechanisms, such as post‐translational modifications that destabilize the PTEN protein, though the key upstream factors controlling this process in TNBC remain largely unknown. Concurrently, TNBC metastasis relies on cellular plasticity programs like the epithelial‐to‐mesenchymal transition (EMT), which is orchestrated by master transcription factors such as ZEB1, SNAIL, and TWIST [20, 21]. However, the specific signaling events that initiate and sustain a ZEB1‐dependent EMT program in TNBC are not fully elucidated. Whether a common upstream regulator coordinates PTEN stability with the induction of pro‐metastatic programs like EMT represents a critical unanswered question.

In this study, through an unbiased bioinformatics screen of breast cancer samples, we identified *testis‐specific Y‐Like protein 5* (*TSPYL5*) as the top metastasis‐associated gene that provides a unifying solution to these questions. We established TSPYL5 as a master regulator orchestrating oncogenic signaling with pro‐metastatic cellular reprogramming in TNBC through a previously uncharacterized cascade. Mechanistically, TSPYL5 directly binds and antagonizes the deubiquitinase USP10, leading to the proteasomal degradation of PTEN. The resulting loss of PTEN function subsequently unleashes sustained PI3K/AKT pathway activation, which in turn drives a ZEB1‐dependent EMT program. Collectively, our study delineates the TSPYL5‐USP10‐PTEN‐AKT‐ZEB1 regulatory axis as a fundamental driver of TNBC metastatic progression and validates TSPYL5 as a high‐priority therapeutic target.

## 2 Results

### 2.1 TSPYL5 is a Novel Biomarker Associated With Metastasis and Poor Prognosis in Breast Cancer

**FIGURE 1:**
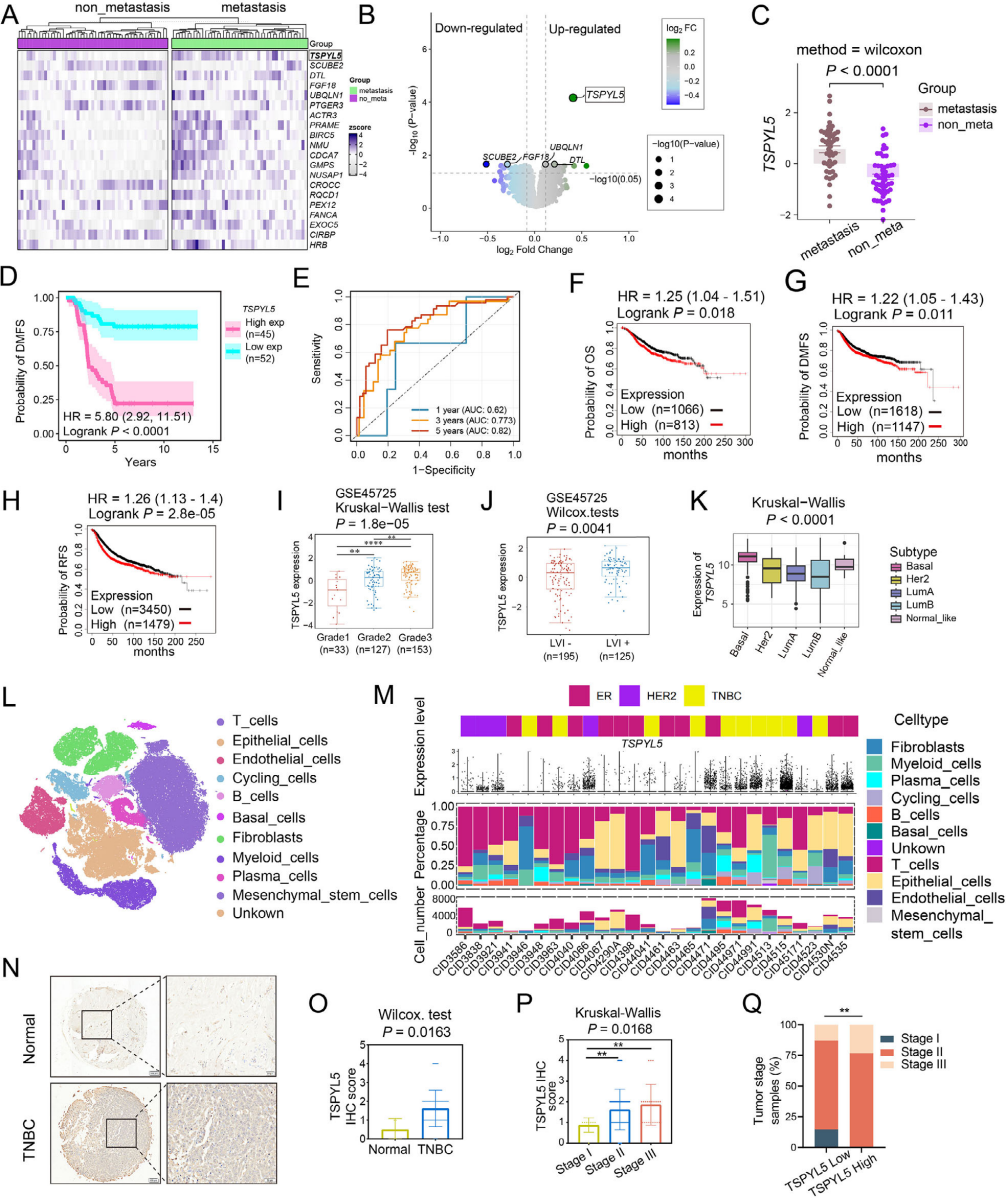
High TSPYL5 Expression Correlates with Metastasis and Predicts Poor Prognosis in Breast Cancer. (A) Heatmap displaying the top 20 DEGs between primary tumors from breast cancer patients who developed distant metastases (Metastatic, n = 46) and those who remained disease‐free (Non‐metastatic, n = 51). (B) Volcano plot visualizing the DEGs from the same cohort, highlighting *TSPYL5* as a top upregulated gene in the metastatic cohort. (C) Box plot comparing *TSPYL5* mRNA expression (log2[TPM]) between metastatic and non‐metastatic primary tumors. (D) Kaplan‐Meier analysis of DMFS in breast cancer patients stratified by high versus low *TSPYL5* expression. (E) Time‐dependent Receiver Operating Characteristic (ROC) curve analysis assessing the predictive power of *TSPYL5* for DMFS at 1, 3, and 5 years. Area Under the Curve (AUC) values are indicated. (F–H) Kaplan‐Meier analyses from the KMplotter database validating the association between *TSPYL5* expression and OS (F), DMFS (G), and RFS (H) in breast cancer. (I,J) Validation of *TSPYL5* expression in the GSE45725 cohort, stratified by pathological grade (I) and LVI status (J). (K) Box plot of *TSPYL5* expression across different breast cancer subtypes in the TCGA‐BRCA cohort. (L) t‐SNE visualization of 96 976 single cells from breast cancer tissues, with major cell types annotated by color. (M) Feature plots showing the specific expression of *TSPYL5* across different breast cancer subtypes at the single‐cell level. (N) Representative immunohistochemical staining of TSPYL5 in TNBC and adjacent normal breast tissue from a tissue microarray (TMA) cohort (n = 120). (O) Box plot showing the significantly elevated TSPYL5 protein level (quantified by IHC score) in tumor tissues compared to adjacent normal breast tissue. (P) Box plot showing a progressive increase in TSPYL5 protein level with advancing clinical stages (I, II, III) of TNBC. (Q) Stacked bar chart showing the distribution of clinical stages within TSPYL5‐high and TSPYL5‐low TNBC patient groups, as defined by median IHC score. *p*‐value was determined by Chi‐square test. DMFS: Distant metastasis‐free survival; OS: overall survival; RFS: relapse‐free survival. Sample sizes (n) are explicitly labeled in each panel or represent the entire specified cohort. Statistical significance was evaluated using a two‐tailed, unpaired Student's *t*‐test, Wilcoxon rank‐sum test (C, J, O), Kruskal‐Wallis test (I, K, P), the Kaplan‐Meier method with the log‐rank test (D, F, G, H), ROC curve analysis (E), or Pearson's Chi‐square test (Q). *p < 0.05, **p < 0.01, ***p < 0.001, ****p < 0.0001; ns, not significant.

To uncover putative drivers of breast cancer metastasis, we re‐analyzed the landmark microarray dataset from van ’t Veer et al. *Nature* 2002 study [22], comparing gene expression profiles of primary tumors from patients who subsequently developed distant metastases with those who remained disease‐free. From this unbiased screen, the *TSPYL5* gene emerged as the most statistically significant upregulated gene in the metastatic cohort, ranking first by adjusted *P*‐value (Figure 1A–C). Consistent with this finding, Kaplan‐Meier analysis revealed that high *TSPYL5* expression was strongly associated with significantly shorter distant metastasis ‐ free survival (DMFS; HR = 5.8, log‐rank *p* < 0.0001, Figure 1D). Furthermore, time‐dependent ROC analysis confirmed *TSPYL5* as a robust predictor of metastatic outcome, as its predictive power strengthened over time, reaching an AUC of 0.82 at 5 years (Figure 1E). To validate the generalizability of *TSPYL5*’s prognostic value, we interrogated multiple large‐scale, independent breast cancer patient cohorts using the KMplotter database [23]. This analysis confirmed that high *TSPYL5* expression is a potent predictor of adverse outcomes associated with significantly shorter overall survival (OS; HR = 1.25, *p* = 0.018, Figure 1F), DMFS (HR = 1.22, *p* = 0.011, Figure 1G), and recurrence‐free survival (RFS; HR = 1.26, *p* = 2.8e‐05, Figure 1H). Critically, this strong association with metastatic potential was substantiated at the pathological level in the GSE45725 cohort, where *TSPYL5* levels were higher in tumors exhibiting high tumor grade (*p* = 1.8e‐05, Figure 1I) and lymphovascular invasion (LVI; *p* = 0.0041, Figure 1J). As LVI represents a key early step in the metastatic cascade, this finding reinforced the involvement of *TSPYL5* in the metastatic progression of breast cancer.

Moreover, this pro‐metastatic signature of *TSPYL5* was particularly pronounced within the most aggressive subtypes of breast cancer. Stratification of the TCGA‐BRCA cohort revealed that *TSPYL5* expression was most highly enriched in basal‐like breast cancer (BLBC), a subtype defined by its high propensity for distant metastasis (Figure 1K, Figure S1A, B). Remarkably, high *TSPYL5* expression was significantly associated with shorter OS exclusively in patients with BLBC (*p* = 0.041; Figure S1C), whereas no significant prognostic impact was observed across Luminal A, Luminal B, or HER2‐enriched subtypes (all *p* > 0.05; Figure S1D–F). Consistent with this clinical observation, interrogation of the Cancer Cell Line Encyclopedia (CCLE) database independently corroborated that *TSPYL5* expression is generally higher in TNBC cell lines relative to HER2‐enriched or Luminal cell lines (Figure S1G,H). Furthermore, we reanalyzed the single‐cell RNA sequencing (scRNA‐seq) data from 26 breast cancer samples (including 11 ER+, 5 HER2+, 10 TNBC), [24] and generated a comprehensive cellular atlas of 96 976 individual cells, which resolved into 27 distinct cell subpopulations spanning ten major cell types (Figure 1L, Figure S2A–K). Among them, *TSPYL5* expression was predominantly found in cells from TNBC samples, while being markedly lower in ER+ or HER2+ tumors (Figure 1M). Importantly, to anchor our bioinformatics findings at the protein level and assess their clinical relevance, we performed IHC analysis on a well‐characterized tissue microarray (TMA) of our own cohort, comprising 110 TNBC patient samples (clinicopathological data in Table S1). This analysis decisively confirmed that TSPYL5 protein is significantly elevated in tumor tissues compared to adjacent normal breast tissues (Figure 1N,O). More strikingly, we observed a progressive and significant increase in TSPYL5 protein levels with advancing clinical stage (Figure 1P), leading to a clear enrichment of stage III tumors within the TSPYL5‐high patient group (Figure 1Q). Collectively, these results from bulk transcriptomic, cell line, single‐cell, and protein‐level analyses revealed that TSPYL5 is predominantly enriched in the aggressive TNBC subtype, with a higher risk of recurrence and metastasis.

Additionally, we investigated the genomic basis for this pro‐metastatic expression pattern of *TSPYL5*. We found that *TSPYL5* is frequently amplified (7%–20%) across the breast cancer cohorts (Figure S3A). This amplification was strongly associated with features indicative of high metastatic propensity, including higher tumor histologic grade, an enrichment within the aggressive basal‐like subtype, and ultimately, higher mortality (Figure S3B). Furthermore, *TSPYL5* amplification was associated with a higher mutational burden (Figure S3C), and an elevated Buffa hypoxia score, acting as a known driver of metastatic progression (Figure S3D). Crucially, *TSPYL5* amplification itself was a robust prognosticator of poor prognosis, leading to significantly shorter OS (log‐rank *p* = 7.119e ‐ 3, Figure S3E) and higher recurrence rates (log‐rank *p* = 3.356e‐5, Figure S3F). Thus, *TSPYL5* amplification serves as a genomic basis for its overexpression and dictates metastatic potential in breast cancer.

### 2.2 *TSPYL5* Defines a Subpopulation of Malignant, Stem‐Like Epithelial Cells With Pro‐Metastatic and Genomically Unstable Features

**FIGURE 2:**
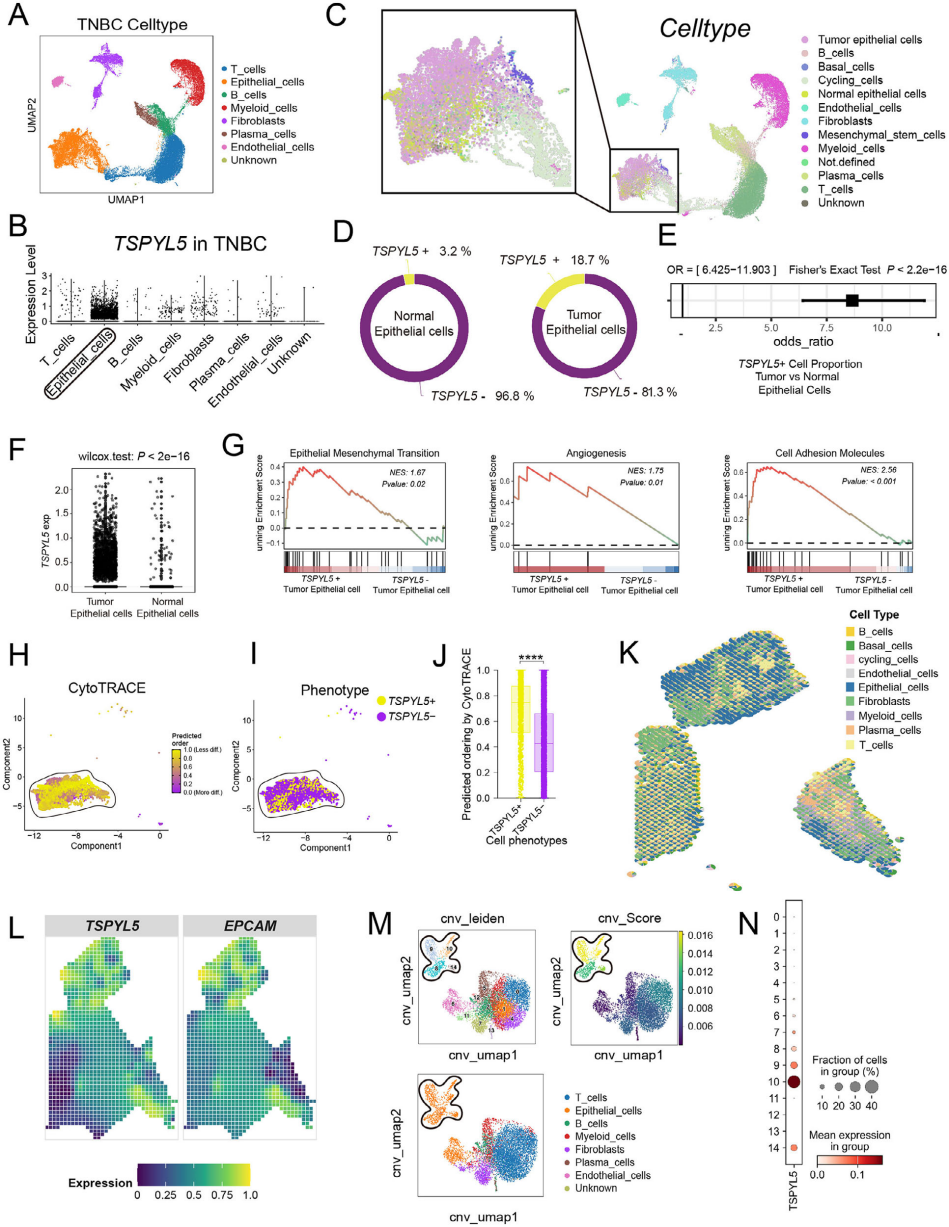
*TSPYL5* Defines a Subpopulation of Malignant, Stem‐Like Epithelial Cells with Pro‐Metastatic and Genomically Unstable Features. (A) UMAP visualization of 40 982 single cells from the TNBC tissue, with major cell types annotated by color. (B) Bar plot illustrating the cell‐type specific expression of *TSPYL5*, highlighting its enrichment within the epithelial cell compartment. (C) UMAP visualization illustrating the distinct clustering of the epithelial cell compartment. Malignant epithelial cells are highlighted in pink, whereas normal epithelial cells are depicted in light yellow. (D) A donut chart showing the distribution of *TSPYL5*+ cells in aneuploid (malignant) and diploid (normal) epithelial populations. Malignancy was inferred by assessing aneuploidy using the copyKAT algorithm. (E) Quantification confirms a significant enrichment of *TSPYL5*+ cells within the malignant epithelial population (P‐value by Fisher's exact test). (F) Box plot showing higher *TSPYL5* mRNA expression in malignant versus normal epithelial cells. (G) GSEA plots showing that *TSPYL5*‐positive malignant epithelial cells are significantly enriched for hallmark metastatic gene signatures, including EMT, angiogenesis, and cell adhesion. (H,I) UMAP visualization of the malignant epithelial cell subset, with cells colored by their differentiation potential (stemness score) as calculated by CytoTRACE (H) and with cells colored to distinguish *TSPYL5*+ (yellow) and *TSPYL5*‐ (purple) populations (I), illustrating co‐localization of *TSPYL5* expression with high stemness scores. (J) Box plot quantifying the significantly higher stemness scores in *TSPYL5*+ versus *TSPYL5*‐ malignant epithelial cells. (K) Spatial transcriptomics analysis of a TNBC tissue section showing the spatial distribution of deconvoluted cell types. (L) Spatial feature plot illustrating that *TSPYL5* expression co‐localizes with the epithelial marker *EPCAM* within tumor regions. (M) UMAP of all single cells colored by cluster identity (top left), cell type (bottom left), and inferred copy number variation (CNV) score (right). (N) Dot plot depicting *TSPYL5* expression across all identified cell clusters. Dot size is proportional to the percentage of cells expressing the gene within each cluster, while the color scale reflects the average expression level. Statistical significance was determined by Fisher's exact test (E) or a two‐tailed Wilcoxon rank‐sum test (F, J). GSEA (G) was computed using the fgsea package. ****p < 0.0001.

To dissect the cellular basis of TSPYL5's role in TNBC, our single‐cell analysis further revealed that *TSPYL5* expression was predominantly localized to the epithelial cell compartment (Figure 2A,B, Figure S4A–E). Gene Set Enrichment Analysis (GSEA) of this population showed that *TSPYL5*‐expressing cells were significantly enriched for gene signatures associated with metastasis, including epithelial‐mesenchymal transition, angiogenesis, and cell adhesion (Figure S4F). Furthermore, these cells displayed higher CytoTRACE scores, indicative of a less differentiated, stem‐like state (Figure S4G–I). This prompted us to hypothesize that these pro‐tumorigenic properties were specifically driven by the malignant, rather than the normal, epithelial cell compartment. To test this, we first segregated the epithelial compartment into malignant (aneuploid) and normal (diploid) populations using the copyKAT algorithm (Figure 2C). This analysis revealed a stark and significant enrichment of *TSPYL5*‐positive cells within the malignant population compared to their normal counterparts (18.7% vs. 3.2%, respectively; *p* < 2.2e ‐ 16, Figure 2D–F). Then, we restricted our functional analyses to this malignant epithelial subset. Crucially, the association with metastatic pathways (Figure 2G) and stem‐like potential (Figure 2H–J) was not only maintained but significantly strengthened. Collectively, these findings establish that *TSPYL5* expression delineates a distinct subpopulation of malignant epithelial cells characterized by heightened stemness and potent pro‐metastatic transcriptional properties necessary for tumor progression.

To validate the in situ spatial distribution of *TSPYL5* within the native tumor architecture, we leveraged spatial transcriptomics on TNBC tissues (Figure S5A,B). Spatial deconvolution analysis confirmed that *TSPYL5* expression was predominantly co‐localized with malignant epithelial cell signatures in situ (Figure 2K,L). More revealingly, InferCNV analysis of the spatial data demonstrated that epithelial regions with the highest *TSPYL5* expression (clusters 8, 9, 10, and 14) displayed significantly elevated CNV scores (Figure 2M,N). This finding forges a critical link between *TSPYL5* expression and genomic instability, a key enabling characteristic that fuels tumor evolution and the generation of aggressive, metastatic subclones. Therefore, by marking these genomically unstable hotspots within the tumor, *TSPYL5* identifies the very cellular populations poised for metastatic dissemination.

Taken together, these high‐resolution analyses establish that *TSPYL5* marks a distinct subpopulation of malignant epithelial cells in TNBC characterized by a triad of aggressive features: a pro‐metastatic transcriptional program, enhanced stemness and genomic instability, positioning it as a key factor in tumor evolution and metastasis.

### 2.3 TSPYL5 Functionally Drives Multiple Hallmarks of TNBC Aggression In Vitro

**FIGURE 3:**
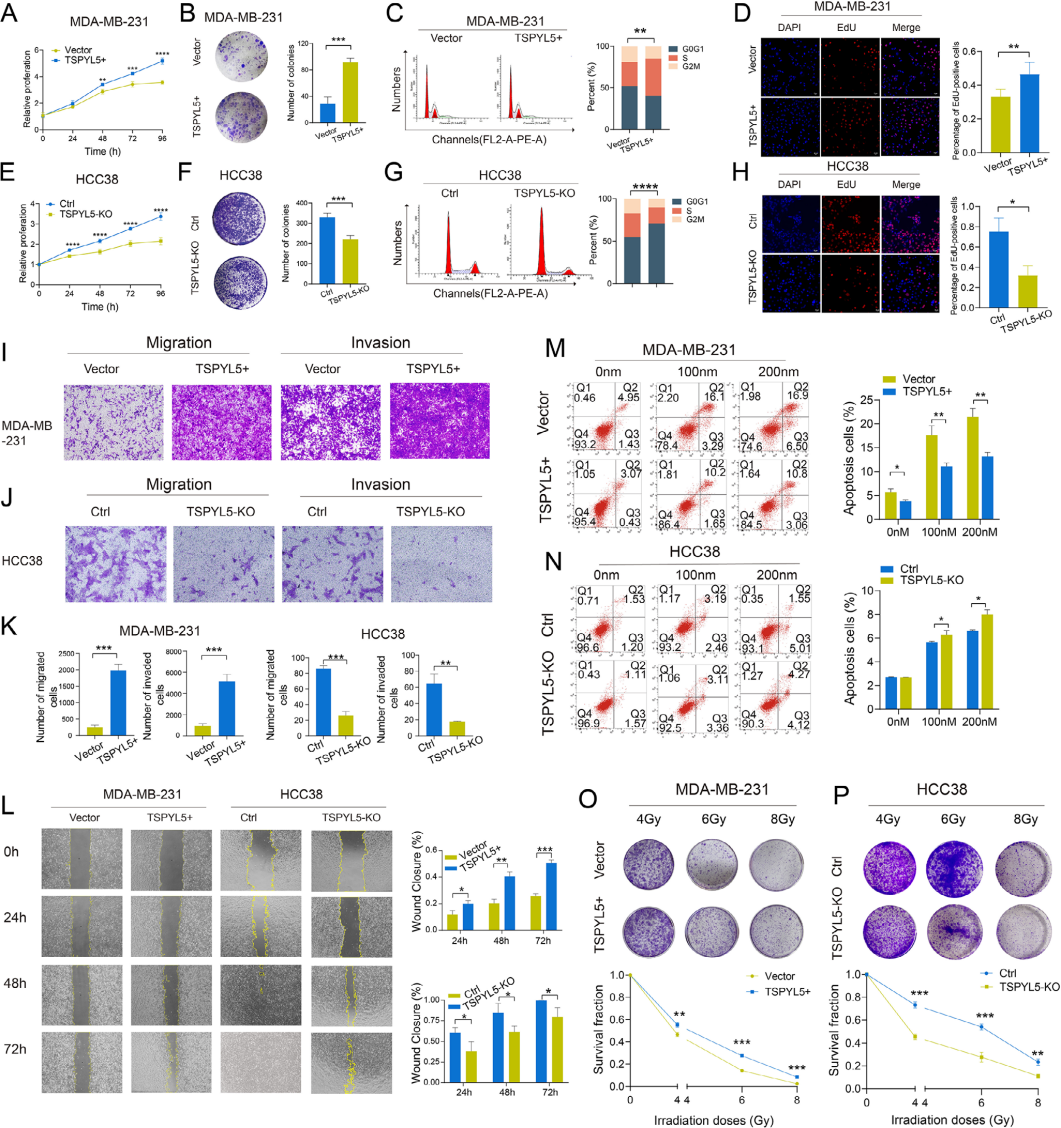
TSPYL5 Functionally Drives Multiple Hallmarks of TNBC Aggression in vitro. (A–D) TSPYL5‐overexpression in MDA‐MB‐231 cells significantly enhanced cell proliferation (A), colony formation (B), G1/S phase transition (C) and DNA synthesis (D). (E–H) TSPYL5‐knockout in HCC38 cells suppressed cell proliferation (E), colony formation (F), G1/S phase transition (G) and DNA synthesis (H). Scale bars of D and H: 20 µm. (I–K) Transwell assays showing that TSPYL5‐overexpression in MDA‐MB‐231 cells enhanced cell migration and invasion (I, K), and TSPYL5‐knockout in HCC38 cells suppressed cell migration and invasion (J, K). (L) Wound‐healing assays showing that TSPYL5‐overexpression in MDA‐MB‐231 cells enhanced cell migration, while TSPYL5‐knockout in HCC38 cells suppressed cell migration. (M, N) TSPYL5‐overexpression in MDA‐MB‐231 cells suppressed (M) while TSPYL5‐knockout in HCC38 cells aggravated (N) paclitaxel‐induced cell apoptosis. (O, P) TSPYL5‐overexpression in MDA‐MB‐231 cells enhanced (O) while TSPYL5‐knockout in HCC38 cells decreased (P) the cell livability after the γ‐irradiation treatment. Quantitative data are presented as the mean ± SD from at least three biological replicates. Statistical significance was determined by a two‐tailed Student's *t*‐test or one‐way ANOVA followed by Tukey's post hoc test for multiple comparisons. *p < 0.05, **p < 0.01, ***p < 0.001, ****p < 0.0001; ns, not significant.

To investigate the role of TSPYL5 in TNBC, we selected four commonly used TNBC cell lines including the low‐TSPYL5‐expressing cell line MDA‐MB‐231 and three high‐TSPYL5‐expressing cell lines HCC38, BT‐20 and BT‐549, and performed complementary loss‐ and gain‐of‐function studies. Lentiviral‐mediated TSPYL5 overexpression in MDA‐MB‐231 robustly promoted cell proliferation (Figure 3A), colony formation (Figure 3B), G1/S phase transition (Figure 3C) and DNA synthesis (Figure 3D), enhanced cell invasive and migratory capacities (Figure 3I,K,L), and also augmented the resistance to paclitaxel and γ‐radiation treatments (Figure 3M,O). Conversely, genetic ablation of *TSPYL5* via CRISPR/Cas9 in high‐expressing TNBC cells resulted in a profound suppression of above oncogenic phenotypes (Figure 3E–H,J–L,N,P, Figure S6A–O). These findings provided in vitro evidence supporting the aggressive phenotypes predicted by our multi‐omic analyses, establishing TSPYL5 as a potential driver of proliferation, metastasis, and therapeutic resistance in TNBC.

### 2.4 TSPYL5 Unleashes Aggressive Tumor Growth and Spontaneous Metastasis In Vivo

**FIGURE 4:**
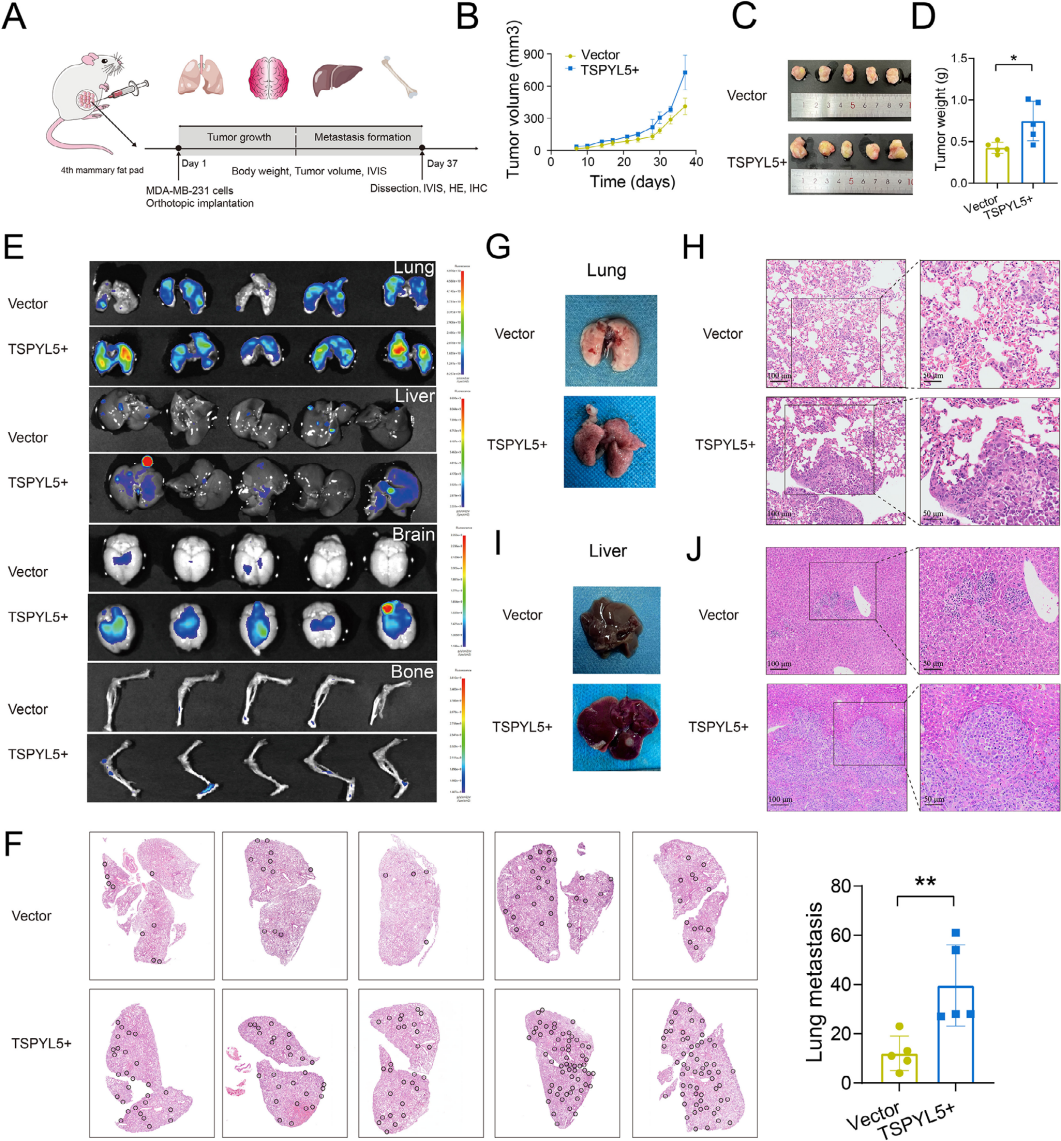
TSPYL5 Unleashes Aggressive Tumor Growth and Spontaneous Metastasis in vivo. (A) Schematic illustrating the experimental design of the orthotopic xenograft model in NCG mice. (B) Primary tumor growth curves for mice bearing tumors derived from TSPYL5+ or Vector cells. Tumor volume was measured at the indicated time points. (C) Representative gross images of primary tumors at the study endpoint. (D) Quantification of primary tumor weights at necropsy. (E) Ex vivo fluorescence imaging of major organs demonstrating metastatic burden. (F) Representative H&E‐stained sections of lung tissue and quantification of metastatic foci per lung. (G‐J) Representative gross images and corresponding H&E‐stained sections of metastatic lesions in the lungs (G, H) and liver (I, J) from mice in both groups. Scale bars of H and J: 100 µm (left panels) and 50 µm (right magnified panels). Data are presented as mean ± SD of n = 5 mice per group. Statistical significance was determined by a two‐way ANOVA for tumor growth curves (B) and a two‐tailed Student's t‐test for endpoint analyses (D, F). *p < 0.05, **p < 0.01.

To assess the impact of TSPYL5 on tumor progression in vivo, we established an orthotopic xenograft model (Figure 4A). TSPYL5 overexpression (TSPYL5+) conferred a dramatic growth advantage to MDA‐MB‐231 cells implanted in the mammary fat pad of NCG mice. Longitudinal monitoring revealed a markedly higher tumor growth rate in TSPYL5+ group compared to controls throughout the study duration (Figure 4B). This culminated in profoundly larger final tumor volumes (728.1 ± 162.1 mm^3^ vs. 412.0 ± 75.7 mm^3^, *p* = 0.0015) and weights (0.71 ± 0.24 g vs. 0.41 ± 0.08 g, *p* = 0.0269) compared to controls (Figure 4C,D).

More critically, this aggressive primary growth was coupled with a devastating increase in spontaneous metastatic dissemination. Ex vivo fluorescence imaging revealed a markedly higher metastatic burden in the TSPYL5+ group across multiple sites including lungs, livers, brains, and bones (Figure 4E). Notably, histopathological quantification of the lungs, a primary site for TNBC metastasis, revealed a 3.4‐fold increase in metastatic foci in the TSPYL5+ group compared to controls (37.33 ± 15.79 vs. 11 ± 6.72 lesions per mouse; *p* = 0.0037, Figure 4F). These lesions were validated as malignant by H&E staining, showing characteristic features of invasive growth and disrupted tissue architecture (Figure 4G–J). Taken together, these data provide powerful evidence that TSPYL5 promotes the entire metastatic cascade from a primary tumor, transforming localized disease into a systemic threat.

### 2.5 TSPYL5 Facilitates Post‐Intravasation Stages of the Metastatic Cascade

**FIGURE 5:**
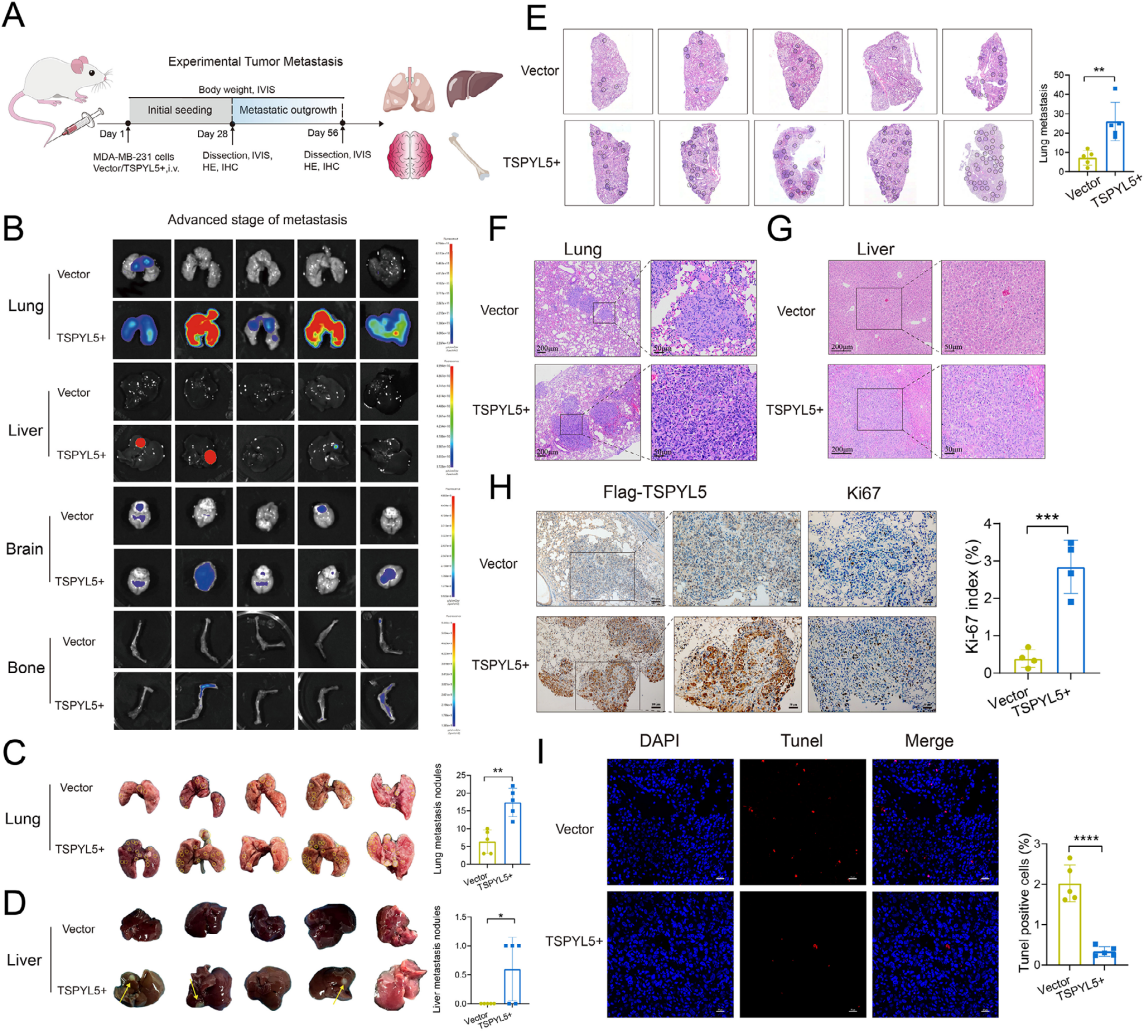
TSPYL5 Promotes the Colonization and Outgrowth Stages of Metastasis In Vivo. (A) Schematic of the experimental metastasis model, where MDA‐MB‐231 cells stably expressing TSPYL5 (TSPYL5+) or a vector control were injected via the tail vein into NCG mice. (B) Representative ex vivo fluorescence imaging of major organs at 8 weeks post‐injection, revealing an increased metastatic burden (lungs, liver, brain, bone) in the TSPYL5+ group. (C, D) Gross photographs showing a higher number of macroscopically visible metastatic nodules on the lungs (C) and livers (D) (indicated by circles and arrows) in the TSPYL5+ group. (E) Representative H&E‐stained lung sections, revealing increased metastatic foci in the TSPYL5+ group. (F, G) High‐magnification H&E images showing the morphology of established metastatic lesions. Scale bars: 200 µm (left panels) and 50 µm (right magnified panels). (H) IHC for the Flag epitope confirmed expression of Flag‐TSPYL5 within metastatic nodules. Scale bars: 100 µm (left panels) and 50 µm (right magnified panels). IHC for the proliferation marker Ki67 in lung metastases (Scale bars: 50 µm) and quantification of the Ki67‐positive index demonstrated increased proliferation in TSPYL5+ lesions. (I) Representative immunofluorescence images of a TUNEL assay showing a decreased percentage of apoptotic cells in TSPYL5+ lesions. Scale bars: 20 µm. Data are presented as mean ± SD of n = 5 mice per experimental group. Statistical significance was determined using a two‐tailed, unpaired Student's *t*‐test. *p < 0.05, **p < 0.01, ***p < 0.001, ****p < 0.0001.

Having established TSPYL5's role in spontaneous metastasis, a process encompassing local invasion and intravasation, we next sought to isolate its function in post‐intravasation events—namely, circulatory survival, extravasation, and colonization. To this end, we further employed an experimental metastasis model using tail vein injection of MDA‐MB‐231 cells (TSPYL5+ vs. Vector) and implemented a temporal design to dissect initial seeding (4 weeks) from subsequent outgrowth (8 weeks, Figure 5A).

At 4 weeks post‐injection, the TSPYL5+ group exhibited a potent colonization advantage in the lungs, livers, and brains (Figure S7A). Notably, mice injected with TSPYL5+ cells exhibited a markedly higher pulmonary metastatic burden, confirmed by both ex vivo imaging and a significant increase in histologically verified metastatic foci (Figure S7A–D). Moreover, while hepatic metastases were microscopic at this stage, H&E staining of fluorescence‐guided liver sections revealed distinct metastatic lesions exclusively in the TSPYL5+ group (Figure S7E). This enhanced colonization was mechanistically underpinned by a profound survival and proliferative advantage, evidenced by higher Ki‐67 expression and suppressed apoptosis (TUNEL assay) within the nascent metastatic foci (Figure S7F–I).

The consequences of this initial advantage became profoundly evident over time. By 8 weeks, ex vivo fluorescence imaging showed a markedly higher metastatic burden in the TSPYL5+ group across multiple sites including lungs, livers, brains, and bones (Figure 5B). Specifically, the TSPYL5+ group presented a significantly elevated number of visible metastatic nodules on the surfaces of lungs and livers (Figure 5C,D). Notably, 60% of TSPYL5+ mice developed large, overt liver tumors, a phenotype entirely absent in the control group. Histological analysis confirmed these lesions exhibited features of invasive growth and disrupted tissue architecture (Figure 5E–G). This aggressive outgrowth was underpinned by persistently elevated Ki‐67 expression (*p* < 0.001, Figure 5H) and suppressed apoptosis within the established metastases (*p* < 0.0001, Figure 5I). Thus, through temporal dissection of the metastatic journey, we demonstrate that TSPYL5 is not merely an initiator but a sustained enabler of metastatic progression, equipping cancer cells with the critical capabilities required to conquer distant organs.

To rigorously corroborate these findings in vivo, we further evaluated the impact of TSPYL5 loss‐of‐function utilizing stable TSPYL5‐knockout (TSPYL5‐KO) BT‐549 cells. In the orthotopic mammary fat pad xenograft model, TSPYL5 depletion profoundly impaired TNBC tumorigenesis, resulting in significantly decelerated tumor growth trajectories and reduced terminal tumor volumes and weights (Figure S8A–C). Furthermore, using an experimental tail‐vein metastasis model, bioluminescence imaging revealed that TSPYL5 ablation attenuated lung metastatic colonization (Figure S8D), a finding confirmed by macroscopic nodule quantification and subsequent histological evaluation via H&E staining (Figure S8E–H). Collectively, these in vivo loss‐of‐function phenotypes complement our gain‐of‐function data, establishing TSPYL5 as an indispensable driver of both TNBC primary growth and distant metastasis.

### 2.6 TSPYL5 Promotes TNBC Metastasis by Inducing a ZEB1‐Dependent Epithelial‐to‐Mesenchymal Transition

**FIGURE 6:**
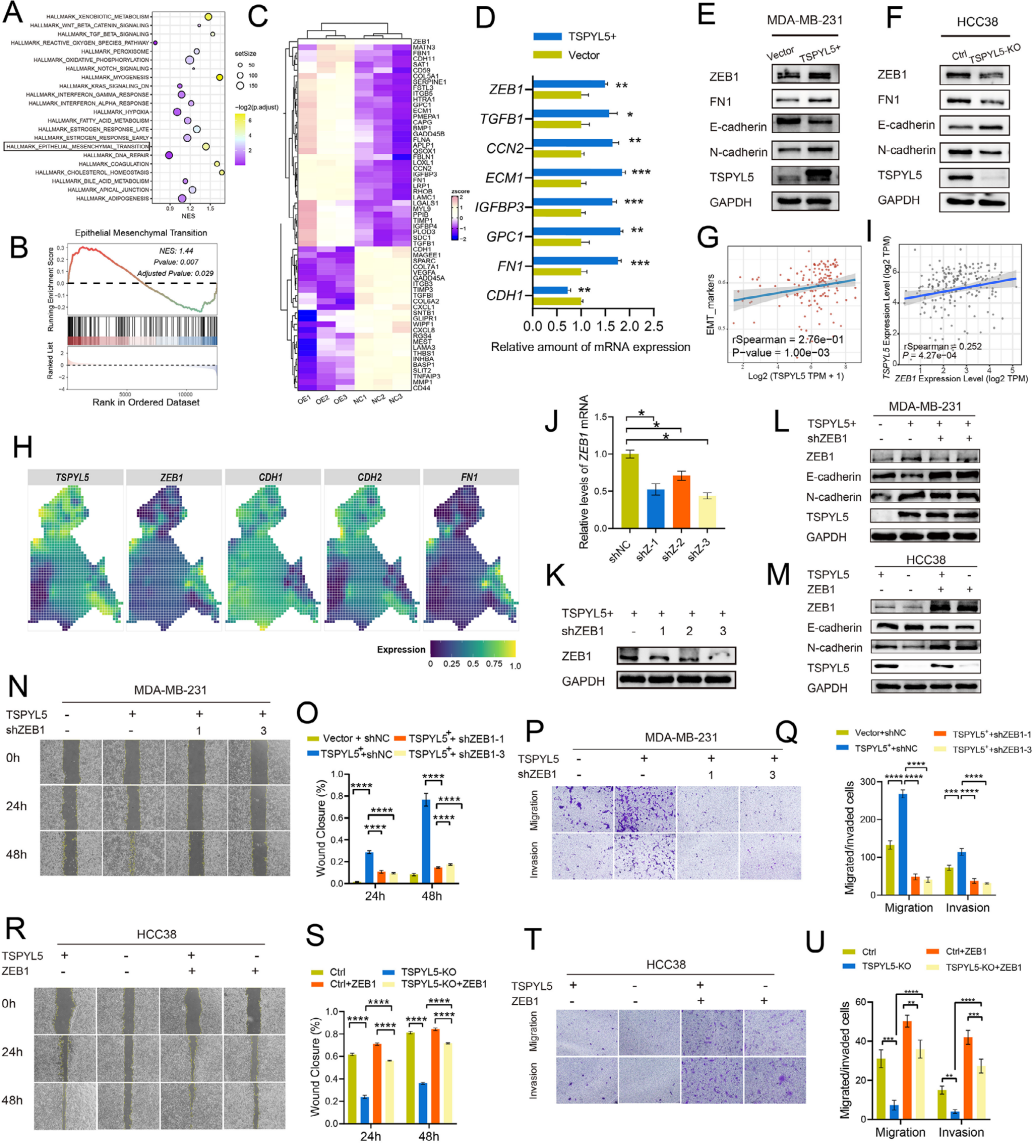
TSPYL5 Drives Metastasis by Inducing a ZEB1‐Dependent Epithelial‐to‐Mesenchymal Transition. (A) Dot plot showing the GSEA results for MSigDB Hallmark gene sets of the transcriptomic analysis. The x‐axis shows the Normalized Enrichment Score (NES), the dot size corresponds to the gene set size, and the color scale indicates the FDR q‐value. (B) GSEA plot showing significant enrichment of the Hallmark EMT gene set in TSPYL5+ cells. (C) Heatmap illustrating the differential expression of core EMT‐associated genes identified from RNA‐seq. (D) RT‐qPCR analysis confirming the upregulation of mesenchymal markers and downregulation of epithelial markers in TSPYL5+ cells. Data were normalized to *GAPDH* and are presented as fold change relative to vector control cells. (E, F) Western blot analysis of key EMT protein markers in TSPYL5+ (E) and TSPYL5‐KO (F) cells versus controls. (G) Correlation analysis between *TSPYL5* expression and EMT pathway scores in the TCGA‐BLBC cohort. (H) Spatial transcriptomics analysis demonstrating co‐localization of *TSPYL5* with EMT regulators within the tumor microenvironment. (I) Correlation analysis of *TSPYL5* and *ZEB1* mRNA expression in the TCGA‐BLBC cohort. (J, K) Validation of ZEB1 knockdown (KD) efficiency by RT‐qPCR (J) and Western blot (K) in MDA‐MB‐231 cells transduced with three distinct shRNAs against *ZEB1*. (L) Western blot demonstrating that ZEB1 knockdown reverses the EMT marker changes induced by TSPYL5‐overexpression in MDA‐MB‐231 cells. (M) Western blot demonstrating that ZEB1 overexpression restores the EMT marker changes induced by TSPYL5‐knockout in HCC38 cells. (N, O) Wound healing assays showing that ZEB1 knockdown abrogates the enhanced migration of TSPYL5‐overexpressing MDA‐MB‐231 cells. (P, Q) Transwell migration and invasion assays demonstrating that ZEB1 knockdown impairs the pro‐migratory and pro‐invasive effects of TSPYL5. (R, S) Wound healing assays showing that ZEB1 overexpression rescues the impaired cell migration in TSPYL5‐KO HCC38 cells. (T, U) Transwell migration and invasion assays demonstrating that ZEB1 overexpression restores the migratory and invasive capabilities of TSPYL5‐KO HCC38 cells. Quantitative data are presented as the mean ± SD of at least n = 3 independent experiments. Statistical significance was determined by Spearman's rank correlation test (Rs; G, I), two‐way repeated‐measures ANOVA (O, S), one‐way ANOVA followed by Tuke's post hoc test (Q, U), or a two‐tailed Student's *t*‐test (D, J). *p < 0.05, **p < 0.01, ***p < 0.001, ****p < 0.0001; ns, not significant.

To elucidate the molecular mechanisms underpinning TSPYL5‐driven metastasis, we performed RNA sequencing on TSPYL5+ MDA‐MB‐231 cells and their corresponding lung metastatic lesions (Figure S9A–F). We identified 3235 DEGs in MDA‐MB‐231 cells (1739 upregulated and 1496 downregulated DEGs) and 1749 DEGs (844 up‐regulated and 905 down‐regulated DEGs) in lung metastatic lesions. Further GSEA analysis of the results revealed that the EMT pathway was significantly enriched (NES = 1.44, *p* = 0.007, Figure 6A–C). To validate this bioinformatic finding, we confirmed profound shifts in key EMT‐associated genes (*ZEB1*, *TGFB1*, *CCN2*, *ECM1*, *IGFBP3*, *GPC1*, *FN1*, *CDH1*) via RT‐qPCR analysis (Figure 6D). Subsequent Western blot analysis confirmed these changes at the protein level, demonstrating a classic EMT signature: the robust upregulation of mesenchymal markers (e.g., ZEB1, FN1, N‐cadherin) coupled with the concomitant loss of the epithelial adhesion molecule E‐cadherin (Figure 6E,F) in TSPYL5+ cells, with TSPYL5‐KO cells showing the converse pattern. These data strongly indicated that TSPYL5 executes its pro‐metastatic function by instigating the EMT program.

To establish the clinical relevance of this finding, we interrogated the link between TSPYL5 and EMT across multiple patient data platforms. In TCGA data from basal‐like breast cancers, TSPYL5 expression was significantly and positively correlated with EMT pathway scores (Rs = 0.276, *p* = 0.0001, Figure 6G). This association was echoed at the single‐cell level, where we previously observed significant EMT signature enrichment in *TSPYL5*‐positive malignant epithelial cells (Figure 2G). Finally, spatial transcriptomics provided architectural proof of this connection, revealing a significant co‐localization of *TSPYL5* with the EMT regulators *ZEB1*, *FN1*, *CDH1*, and *CDH2* within the tumor microenvironment (Figure 6H). This multi‐modal evidence from patient cohorts firmly establishes EMT as a core program associated with TSPYL5 expression in TNBC.

Given ZEB1's established role as a master transcription factor of EMT, its significant positive correlation with *TSPYL5* expression (Rs = 0.252, *p* = 4.27e‐04, Figure 6I) prompted us to hypothesize that ZEB1 functions as the critical downstream effector mediating TSPYL5's pro‐metastatic activity. To test this, we silenced ZEB1 using shRNA in TSPYL5+ cells (Figure 6J,K). Strikingly, ZEB1 knockdown was sufficient to reverse the TSPYL5‐induced EMT phenotype, restoring E‐cadherin expression and suppressing N‐cadherin levels (Figure 6L). Functionally, this molecular reversal translated into a profound attenuation of cellular aggression; ZEB1 depletion significantly impaired the enhanced migration and invasion conferred by TSPYL5 (Figure 6N–Q). In the reciprocal experiment, re‐expression of ZEB1 in TSPYL5‐knockout cells was sufficient to rescue their invasive and migratory capabilities (Figure 6M,R–U). Taken together, these data establish a linear regulatory axis where TSPYL5 promotes TNBC metastasis by transcriptionally upregulating the master regulator ZEB1, thereby driving a potent, pro‐invasive EMT program.

### 2.7 TSPYL5 Hijacks the PI3K‐AKT‐mTOR Axis to Unleash a Metastatic EMT Program

**FIGURE 7:**
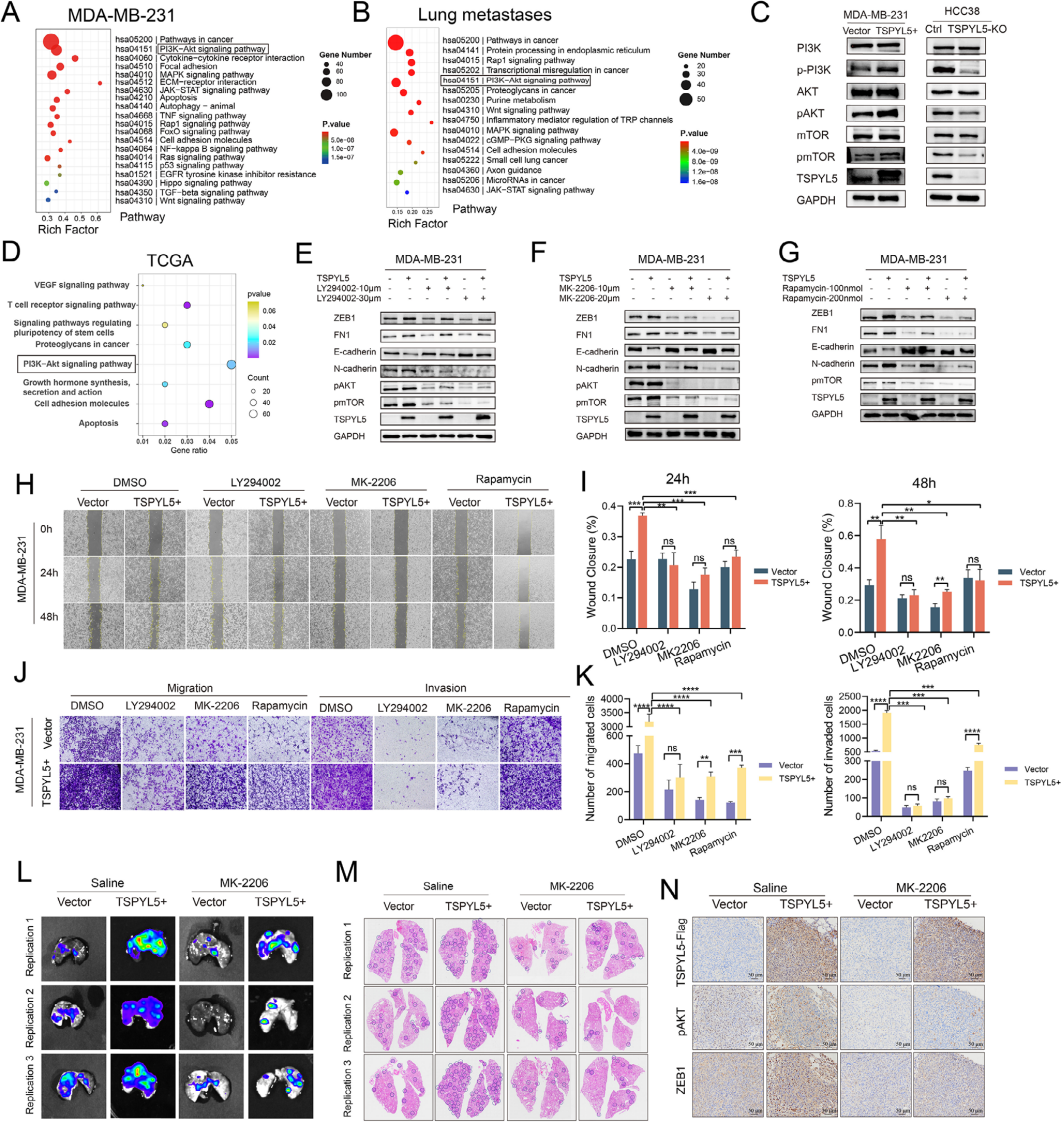
TSPYL5 Hijacks the PI3K‐AKT‐mTOR Axis to Unleash a Metastatic EMT Program. (A,B) KEGG pathway enrichment analysis for RNA‐sequencing data of TSPYL5‐overexpressing MDA‐MB‐231 cells (A) and lung metastases from an experimental metastasis model (B), both highlighting the PI3K‐AKT pathway. (C) Western blot analysis confirming the activation of key PI3K‐AKT‐mTOR pathway components in TSPYL5+ cells and inactivation in TSPYL5‐KO cells. (D) KEGG pathway analysis of DEGs from the TCGA‐BLBC cohort, stratified by *TSPYL5* mRNA expression quartiles. (E‐G) Western blot analysis of EMT markers in TSPYL5+ cells treated with the PI3K inhibitor LY294002 (E), the AKT inhibitor MK‐2206 (F), or the mTOR inhibitor Rapamycin (G). (H,I) Wound healing assays showing that inhibition of the PI3K‐AKT‐mTOR pathway suppresses the enhanced migration of TSPYL5+ cells. Representative images (H) and quantification (I) are shown. (J,K) Transwell migration and invasion assays demonstrating that pharmacological blockade of the PI3K‐AKT‐mTOR pathway impairs the pro‐migratory and pro‐invasive phenotypes induced by TSPYL5. Representative images (J) and quantification (K) are shown. (L,M) Representative images of ex vivo bioluminescence and H&E staining of lungs showing reduced metastatic burden upon MK‐2206 treatment. (N) IHC analysis of lung metastatic nodules for Flag‐TSPYL5, p‐AKT, and ZEB1, demonstrating pathway inhibition in tumors upon MK‐2206 treatment. Scale bars, 50 µm. Data in (I) and (K) are presented as mean ± SD from three independent experiments. Statistical significance was determined by one‐way ANOVA with Dunnett's post hoc test for multiple comparisons against a control. *p < 0.05, **p < 0.01, ***p < 0.001, ****p < 0.0001; ns, not significant.

Having established that TSPYL5 induces a ZEB1‐dependent EMT program, we sought to identify the upstream signaling cascade responsible for this activation. An unbiased screen of our transcriptome data against the KEGG database pinpointed the PI3K‐AKT signaling pathway as a significantly enriched signaling program in both TSPYL5‐overexpressing cells and their metastatic lesions (Figure 7A,B). Western blot analysis confirmed that TSPYL5 overexpression robustly increased the phosphorylation, and thus activation, of PI3K, AKT, and its key downstream effector, mTOR (Figure 7C). Conversely, TSPYL5 knockout resulted in a corresponding decrease in the activation of this axis. Critically, this effect was specific, as TSPYL5 did not perturb other canonical EMT‐regulatory pathways [25], including Wnt/β‐catenin, TGF‐β, ERK, or IL6/STAT3 (Figure S10A,B), isolating PI3K‐AKT‐mTOR as the principal signaling conduit for TSPYL5. To establish the clinical relevance of this mechanism, we analyzed the TCGA‐BLBC cohort and confirmed that *TSPYL5*‐high tumors exhibited a significant enrichment of PI3K‐AKT signaling signatures compared to *TSPYL5*‐low tumors (Figure 7D, Figure S11A,B).

To determine whether PI3K‐AKT‐mTOR signaling is functionally required for TSPYL5‐mediated EMT and metastasis, we performed a series of pharmacological rescue experiments. In TSPYL5‐overexpressing cells, inhibition of PI3K (LY294002), AKT (MK‐2206), or mTOR (Rapamycin) robustly abrogated the established EMT phenotype. This was evidenced by the restoration of E‐cadherin expression and concomitant suppression of mesenchymal markers ZEB1, FN1, and N‐cadherin (Figure 7E–G, Figure S12A–H). This molecular reversion translated into a profound functional rescue, as the enhanced migration and invasion conferred by TSPYL5 were nullified by these inhibitors (Figure 7H–K). Corroborating this dependency, pharmacological inhibition in TSPYL5‐KO HCC38 cells further attenuated their residual migratory and invasive capacity (Figure S13A–G).

To validate this crucial dependency in vivo, we employed an experimental metastasis model coupled with systemic AKT inhibition (Figure S14A). While TSPYL5 overexpression dramatically enhanced lung colonization, administration of the AKT inhibitor MK‐2206 effectively normalized the metastatic burden to control levels (Figure 7L,M, Figure S14B). This phenotypic rescue was mirrored at the molecular level, with IHC analysis confirming reduced phospho‐AKT and ZEB1 expression in metastatic lesions from inhibitor‐treated mice (Figure 7N). Collectively, these bidirectional pharmacological interrogations in vitro and in vivo establish a linear cascade whereby TSPYL5 activates the PI3K‐AKT‐mTOR axis to induce a ZEB1‐driven EMT program, thereby conferring metastatic competence upon TNBC cells.

### 2.8 TSPYL5 Activates the PI3K‐AKT Pathway by Antagonizing USP10‐Dependent PTEN Stabilization

**FIGURE 8:**
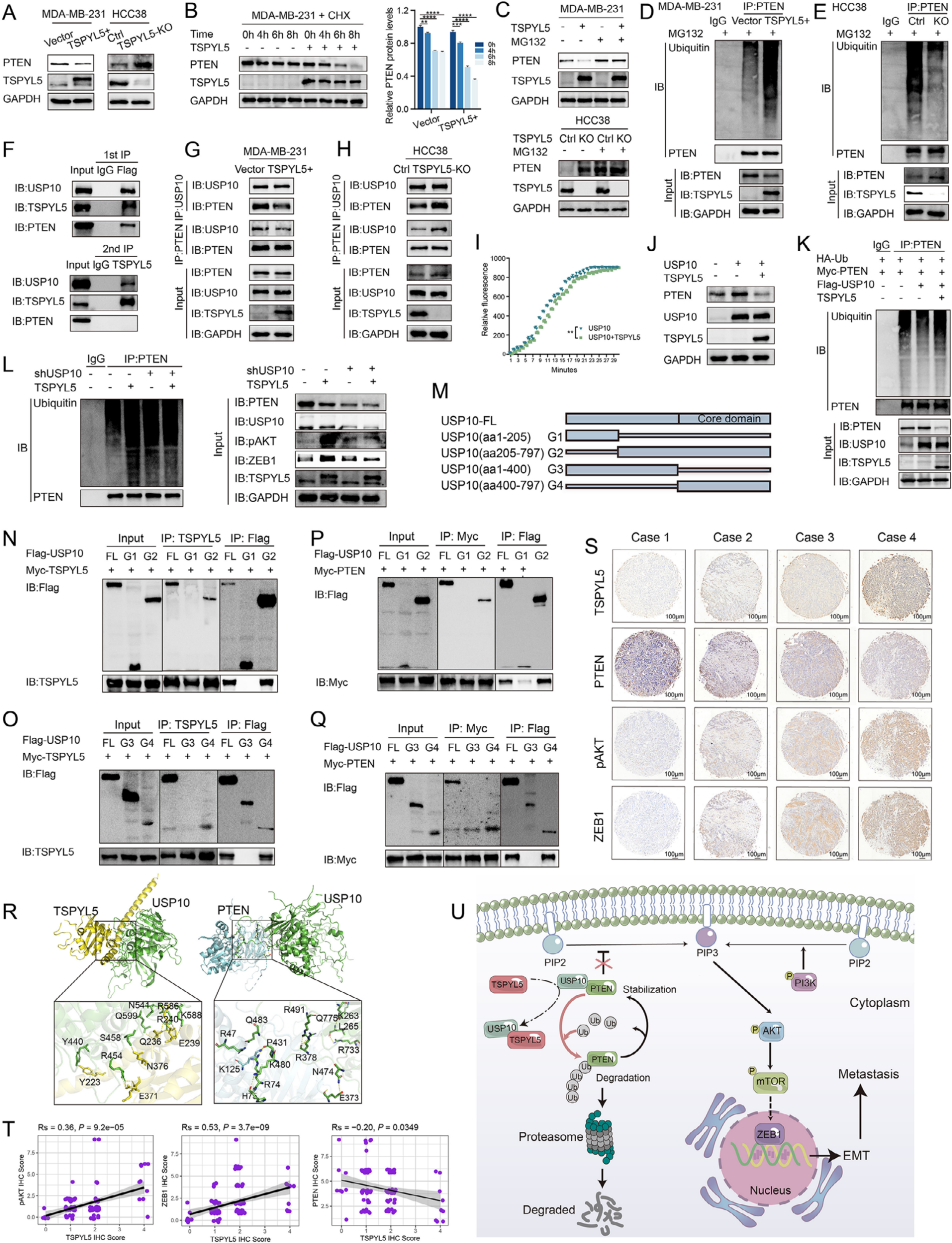
TSPYL5 Promotes PTEN Degradation by Competitively Inhibiting the Deubiquitinase USP10. (A) Western blot analysis of PTEN protein levels in TSPYL5+ MDA‐MB‐231 cells and TSPYL5‐KO HCC38 cells. (B) Western blot showing that TSPYL5overexpression shortened the half‐life of endogenous PTEN in MDA‐MB‐231 cells treated by cycloheximide (CHX). (C) Western blot showing that treatment with the proteasome inhibitor MG132 relieved the TSPYL5‐induced downregulation of PTEN in MDA‐MB‐231 and HCC38 cells. (D,E) Co‐IP assays showing the increased ubiquitylation of PTEN in TSPYL5+ cells (D) and the reduced PTEN ubiquitylation in TSPYL5‐KO cells (E). (F) Sequential immunoprecipitation (Re‐IP) analysis demonstrating that TSPYL5, USP10, and PTEN do not form a stable ternary complex. (G,H) Competitive Co‐IP assays showing that TSPYL5 displaces PTEN from USP10. (I) In vitro deubiquitination assay demonstrating that recombinant TSPYL5 protein inhibits the ability of USP10 to deubiquitinate its substrate. (J,K) USP10‐overexpression increasing PTEN protein levels (J) and decreasing its ubiquitination (K), effects that are reversed by co‐expression of TSPYL5. (L) Western blot showing USP10 knockdown promotes PTEN ubiquitination and degradation, leading to downstream AKT activation and ZEB1 upregulation. USP10 silencing counteracts the ability of TSPYL5 overexpression to further drive these phenotypic changes. (M) Schematic of full‐length (FL) USP10 and its truncation mutants used for domain mapping. (N,O) Co‐IP assays showing the interaction between Myc‐TSPYL5 with various Flag‐tagged USP10 truncations. (P,Q) Co‐IP assays showing the interaction between Myc‐PTEN with the Flag‐tagged USP10 truncations. (R) Molecular docking simulation illustrating the proposed competitive binding of TSPYL5 (yellow) and PTEN (blue) to the same region on USP10 (green). (S) Representative IHC images from a tissue microarray (TMA) of 110 TNBC cases, showing expression patterns of TSPYL5, PTEN, p ‐AKT, and ZEB1. Scale bars: 100 µm. (T) Correlation analysis from the TNBC TMA showing the correlation between TSPYL5 IHC scores and the levels of p‐AKT, ZEB1 and PTEN. (U) A schematic model illustrating the proposed pathway: TSPYL5 competitively binds USP10, preventing PTEN deubiquitination and leading to its degradation. This activates the PI3K‐AKT‐mTOR pathway, ultimately inducing an EMT program to drive metastasis in TNBC. Statistical significance for correlations (T) was determined using Spearman's rank correlation coefficient (Rs). *p < 0.05, **p < 0.01, ***p < 0.001, ****p < 0.0001.

To objectively identify the molecular bridge linking TSPYL5 to the complex PI3K‐AKT‐mTOR cascade, we intersected TSPYL5‐associated proteins with MSigDB‐annotated AKT pathway regulators. Subsequent Protein‐Protein Interaction (PPI) network analysis pinpointed PTEN as the prominent hub bridging these two networks (Figure S15A). Prompted by this unbiased screening and PTEN's established role as the master pathway repressor of PI3K‐AKT‐mTOR signaling [26, 27, 28, 29, 30, 31], we investigated whether TSPYL5 activates this signaling by modulating PTEN. The results showed that overexpression of TSPYL5 in MDA‐MB‐231 cells and lung metastases markedly diminished PTEN protein levels, while its knockdown in HCC38 cells stabilized the protein (Figure 8A, Figure S15B). This regulation might occur post‐translationally, as *PTEN* mRNA levels remained unaffected by TSPYL5 modulation (Figure S15C). Indeed, cycloheximide (CHX) chase assays revealed that TSPYL5 significantly shortened the half‐life of endogenous PTEN, an effect that was completely abrogated by the proteasome inhibitor MG132 (Figure 8B,C, Figure S15D). Congruently, the level of endogenous PTEN polyubiquitination was directly correlated with TSPYL5 expression, confirming that TSPYL5 promotes the proteasomal degradation of PTEN (Figure 8D,E).

Given that the dynamic ubiquitination of PTEN implicates a role for deubiquitinases (DUBs), we sought to identify the specific DUB antagonized by TSPYL5. Guided by a recent functional screen of 48 DUBs that identified USP10 as a highly effective deubiquitinase for stabilizing PTEN and suppressing AKT activation [32], we prioritized USP10 as our primary candidate. Indeed, our Co‐immunoprecipitation (Co‐IP) assays subsequently confirmed the interactions of between USP10 and TSPYL5, as well as between USP10 and PTEN in both MDA‐MB‐231 and HCC38 cells (Figure S15E,F). However, sequential Co‐IP failed to isolate a stable ternary complex, suggesting these interactions are dynamic or mutually exclusive (Figure 8F). The mutually exclusive nature of these interactions suggested a competitive binding model. Indeed, competitive Co‐IP assays revealed that TSPYL5 directly displaces PTEN from USP10 (Figure 8G,H). This physical sequestration was coupled with potent enzymatic inhibition, as TSPYL5 abrogated the deubiquitinase activity of USP10 in vitro (Figure 8I). This antagonistic mechanism—where TSPYL5 binding prevents USP10 from accessing and stabilizing PTEN—ultimately promotes the polyubiquitination of PTEN and targets it for proteasomal destruction (Figure 8J,K).

To determine whether TSPYL5 relies on USP10 to drive downstream signaling, we evaluated the effects of USP10 depletion. Crucially, while USP10 knockdown alone induced PTEN degradation and AKT/ZEB1 activation, it effectively abrogated the ability of TSPYL5 overexpression to further induce these molecular changes (Figure 8L). This provides direct evidence that TSPYL5's regulation of the downstream PTEN/PI3K/AKT axis is mechanistically dependent on the presence of endogenous USP10. To further substantiate the functional causality between these molecular events and cellular phenotypes, we performed rescue assays. Consistent with our mechanistic model, the enhanced cell invasive and migratory capacities driven by TSPYL5 overexpression were robustly abrogated by the concurrent ectopic expression of either USP10 or PTEN (Figure S16A–D). Together, these molecular and phenotypic rescues confirm that the pro‐metastatic function of TSPYL5 fundamentally relies on dismantling the USP10‐PTEN signaling axis.

To delineate the structural basis for this competitive binding, we mapped the interaction domains. Domain mapping experiments using a series of truncated USP10 constructs revealed that both TSPYL5 and PTEN interact with the same C‐terminal region of USP10 (amino acids 400–797) (Figure 8M–Q). This shared binding interface was further corroborated by molecular docking simulations (Figure 8R), which predicted overlapping binding sites for TSPYL5 and PTEN within this domain, providing a compelling structural rationale for their mutually exclusive interaction.

Finally, to ascertain the clinical relevance of this newly identified axis, we performed IHC analysis on a cohort of 110 TNBC tissue samples. The analysis demonstrated a significant positive correlation between TSPYL5 expression and levels of both p‐AKT (Rs = 0.36, *p* < 0.0001) and ZEB1 (Rs = 0.53, *p* < 0.0001). Conversely, a significant inverse correlation was observed between TSPYL5 and PTEN protein expression (Rs = −0.20, *p* = 0.0349) (Figure 8S, T; Figure S17A–D). Collectively, these clinical findings validate our mechanistic observations in patient‐derived tissues and firmly establish the TSPYL5‐USP10‐PTEN axis as a pivotal driver of PI3K/AKT/mTOR pathway hyperactivity and the resultant EMT‐induced metastasis in TNBC (Figure 8U).

## 3 Discussion

Metastatic TNBC remains a disease with a dire prognosis, largely due to its intrinsic aggressiveness and the absence of effective targeted therapies [33]. While hyperactivation of the PI3K‐AKT pathway is a known hallmark of TNBC, the upstream drivers are not fully elucidated for a significant fraction of tumors [34]. This study addresses this critical knowledge gap by identifying a novel, linear signaling axis initiated by TSPYL5. We provide comprehensive evidence that TSPYL5 functions as a potent oncogene in TNBC, orchestrating a cascade that dismantles PTEN‐mediated tumor suppression to drive metastatic progression. The central discovery is a previously undescribed mechanism of pathway activation, wherein TSPYL5 acts as a competitive antagonist of the deubiquitinase USP10, leading to the functional inactivation of PTEN. This work not only provides a new paradigm for PI3K pathway activation but also uncovers a therapeutically tractable vulnerability in TNBC.

### 3.1 TSPYL5 Defines a Genomically Unstable, Metastasis‐Initiating Cell State

The *TSPYL5* gene is located in chromosome 8q22. The amplification of this region has been detected in 21%–27% of primary breast tumors and is associated with a higher propensity of metastatic recurrence [35]. Our findings position TSPYL5 not merely as a biomarker, but as a key determinant of a distinct and highly aggressive malignant cell state. Through an integrated multi‐omics approach, we demonstrate that *TSPYL5* expression delineates a subpopulation of malignant epithelial cells characterized by a dangerous triad of features: a CSC‐like phenotype, a pro‐metastatic transcriptional program, and profound genomic instability. The enrichment of *TSPYL5* within cells exhibiting high stemness scores and expressing gene signatures for EMT and angiogenesis provides a cellular basis for its association with metastasis. CSCs are widely recognized as the cellular drivers of tumor initiation, therapeutic resistance, and metastatic dissemination [36]. Our functional experiments corroborate this, showing that TSPYL5 expression is sufficient to enhance proliferation, invasion, and resistance to standard therapies.

Crucially, our spatial transcriptomics analysis forges a direct link between *TSPYL5* expression and genomic instability, revealing that tumor regions with the highest *TSPYL5* levels display significantly elevated CNV scores. Genomic instability is the engine of tumor evolution, providing the genetic diversity required for subclones to acquire metastatic traits and adapt to new microenvironments. By marking these genomically unstable hotspots, *TSPYL5* identifies the very cellular populations poised for metastatic dissemination. This suggests that TSPYL5 may function as a nexus protein that integrates signals for maintaining a CSC‐like state with the mechanisms that fuel tumor evolution. It does not simply promote one hallmark of cancer; rather, it appears to orchestrate the convergence of multiple aggressive features within a specific cell population, creating a “perfect storm” for the development of systemic disease.

### 3.2 A Hierarchical Cascade: Unraveling the TSPYL5‐USP10‐PTEN‐PI3K‐ZEB1 Pathway

This study delineates a complete, hierarchical signaling cascade from TSPYL5 to metastatic gene expression, with the novelty residing in both its specificity and the unique molecular mechanism at its apex. Our unbiased transcriptomic screen and subsequent pharmacological validation demonstrated that TSPYL5 specifically activates the PI3K‐AKT‐mTOR pathway, with no significant effect on other canonical EMT‐regulatory pathways such as Wnt/β‐catenin or TGF‐β. This specificity isolates the PI3K‐AKT axis as the principal conduit for TSPYL5's oncogenic output, designating it as a clean and rational therapeutic target.

The lynchpin of this cascade is the post‐translational degradation of the tumor suppressor PTEN, a critical negative regulator of the PI3K pathway [33]. While the functional loss of PTEN through genetic deletion or mutation is a frequent event in cancer, its activity is also tightly controlled by post‐translational modifications. Specifically, PTEN stability is maintained by a dynamic balance between ubiquitination and deubiquitination, a process where the key DUBs are not fully elucidated [37]. Our study addressed this by first identifying USP10 as a bona fide PTEN DUB. More importantly, we revealed a novel mechanism for achieving functional PTEN loss in TNBC that retain wild‐type PTEN: TSPYL5 drives PTEN degradation by competitively inhibiting USP10. By physically sequestering USP10, TSPYL5 prevents it from accessing and deubiquitinating PTEN, thereby targeting PTEN for proteasomal degradation. This mechanism of competitive substrate displacement represents a sophisticated mode of oncogenic signaling distinct from direct genetic alterations. The degradation of PTEN consequently unleashes the PI3K‐AKT pathway, which culminates in the activation of a ZEB1‐dependent EMT program. Our work thus provided the missing upstream link, demonstrating how TSPYL5 overexpression can trigger this entire metastatic program, from a specific post‐translational event to the profound cellular changes characteristic of metastatic TNBC.

### 3.3 Context, Contradiction, and Clarity: The Oncogenic Identity of TSPYL5

The literature surrounding TSPYL5 has been marked by contradiction, with reports of both tumor‐suppressive and oncogenic functions, a characteristic of so‐called “double agent” genes [38]. In cancers such as colorectal and gastric carcinoma, TSPYL5 is often silenced by promoter hypermethylation and functions as a tumor suppressor [39, 40, 41]. Conversely, it has been implicated as an oncogene in lung cancer, neuroblastoma and other breast cancer contexts, where it promotes therapy resistance or p53 degradation [42, 43, 44]. In addition, a crucial role for TSPYL5 was recently reported in maintaining the viability of alternative‐lengthening‐of‐telomeres positive (ALT+) cells by protecting USP7‐dependent protection‐of‐telomeres‐1 from proteasomal degradation [45]. Our present study provided evidence for its potent oncogenic role in TNBC and offered a unifying model that resolves these apparent discrepancies.

We propose that the functional output of TSPYL5 is dictated by its expression level, which is in turn determined by the specific genomic alteration—amplification versus methylation. In cancers where TSPYL5 is a suppressor, its expression is low or silenced. At these physiological or sub‐physiological levels, it may participate in homeostatic cellular functions, such as its role as a histone chaperone [46]. However, in TNBC, the frequent TSPYL5 amplification leads to massive protein overexpression. We posit a “stoichiometric competition” model: at these supraphysiological concentrations, the TSPYL5 protein is abundant enough to effectively outcompete endogenous PTEN for binding to the limited cellular pool of USP10. This stoichiometric imbalance tips the scale from PTEN stabilization to its degradation, unleashing the full oncogenic potential of the PI3K pathway. This dosage‐dependent mechanism provides a clear, context‐specific molecular model that elegantly explains how TSPYL5 can switch from a benign or tumor‐suppressive role at low levels to a potent oncogene when its gene is amplified.

### 3.4 Therapeutic Implications: Targeting a New Vulnerability in TNBC

The TSPYL5‐USP10‐PTEN axis represents a previously untapped therapeutic vulnerability in TNBC, offering both a refined predictive biomarker and a novel, high‐value drug target. Clinical trials of PI3K pathway inhibitors in TNBC have shown promise but are hampered by the need for better patient stratification to identify those most likely to respond [47]. Current biomarkers, such as PIK3CA mutations and PTEN loss, do not capture all tumors with pathway hyperactivation [48]. Our data demonstrate that TSPYL5 amplification is a direct, upstream cause of functional PTEN loss. Therefore, screening for TSPYL5 amplification or overexpression could identify a distinct patient population with PI3K pathway dependency driven by PTEN degradation. This cohort may be particularly sensitive to inhibitors of AKT or PI3K, providing a more precise and potentially broader biomarker strategy.

Perhaps more importantly, the TSPYL5‐USP10 PPI itself emerges as a prime therapeutic target. Targeting PPIs is a challenging but validated modality in oncology [49]. Our work provides a strong rationale for this approach by identifying the specific interacting partners, mapping the interaction domain on USP10, and elucidating a competitive binding mechanism. A small‐molecule inhibitor designed to dock into the TSPYL5‐binding pocket on USP10 could prevent this oncogenic interaction, thereby rescuing PTEN from degradation and restoring its tumor‐suppressive function. This strategy is highly appealing for several reasons: (1) it restores a natural tumor suppressor rather than simply inhibiting a kinase; (2) it acts at the apex of the oncogenic cascade, which may prove more effective and less prone to resistance than targeting downstream nodes; and (3) it could circumvent the complex feedback loops often triggered by mTOR or other kinase inhibitors [50].

### 3.5 Limitations and Future Directions

We acknowledge several limitations in the current study that warrant future investigation. First, the high brain metastasis rates observed via tail‐vein injection are primarily based on macroscopic IVIS imaging in a limited sample size (n = 5 per group), which may introduce statistical bias. To rigorously verify the exact penetrance of brain metastasis, future studies utilizing expanded animal cohorts, coupled with specialized brain‐tropic injection routes (such as intra‐cardiac) and high‐resolution histopathological mapping of micro‐metastases, are strictly required. Second, while our in vitro deubiquitination assays demonstrated that TSPYL5 biochemically inhibits USP10 enzymatic activity using generic internally quenched fluorescence‐diubiquitin (IQF‐DiUb) substrates, the direct consequence of this inhibition on PTEN was primarily validated through in cellulo functional assays. A fully reconstituted cell‐free system utilizing purified PTEN as a specific substrate would further strengthen the biochemical characterization of this mechanism. Finally, Since mTOR requires intermediate effectors to dictate gene transcription, and existing literature implicates downstream targets such as HIF‐1α and STAT3 in direct ZEB1 promoter binding [51, 52] alongside potential post‐transcriptional mechanisms [53], elucidating the exact signaling nodes linking mTOR to ZEB1 in this specific TNBC context warrants future investigation.

Moreover, our present study also lays the foundation for several critical avenues of future research and provides a new framework for understanding and treating metastatic TNBC. A high‐priority next step is to solve the high‐resolution crystal or cryo‐EM structure of the TSPYL5‐USP10 complex to facilitate structure‐based design of small‐molecule inhibitors that disrupt this key PPI. Another compelling question is how TSPYL5's function as a DUB antagonist integrates with its established role as a histone chaperone containing a nucleosome assembly protein (NAP) domain. It is tempting to speculate that these two functions cooperate; for instance, TSPYL5‐mediated PI3K activation may create a permissive cellular state that is then exploited by its chaperone activity to epigenetically remodel chromatin at ZEB1 target genes, thereby locking in the metastatic phenotype. Finally, investigating the upstream genomic pressures that drive the focal amplification of the 8q22.1 locus in TNBC could reveal earlier opportunities for intervention, and prospective clinical studies are essential to validate TSPYL5 amplification as a predictive biomarker for response to PI3K/AKT pathway inhibitors in TNBC patients.

In conclusion, we have identified TSPYL5 as a pivotal oncogenic driver in TNBC, dissecting a complete signaling cascade from gene amplification to metastatic programming. This cascade is centered on a novel mechanism of DUB antagonism that results in the functional loss of PTEN. These findings provide a new molecular explanation for TNBC aggression and, crucially, unveil the TSPYL5‐USP10 interface as a prime therapeutic target to restore tumor suppression and combat this devastating metastatic disease.

## 4 Experimental Section

### 4.1 Bulk RNA‐Seq and Genomic Data Analysis

Bulk RNA‐sequencing data were analyzed from The Cancer Genome Atlas (TCGA, https://portal.gdc.cancer.gov/), Gene Expression Omnibus (GEO, https://www.ncbi.nlm.nih.gov/geo/), and the Genotype‐Tissue Expression (GTEx, normal tissues, https://www.gtexportal.org/home/) cohorts. The microarray dataset from the van ’t Veer et al. cohort [22] was retrieved via the Bioconductor seventyGeneData package (https://bioconductor.org/packages/release/data/experiment/vignettes/seventyGeneData/inst/doc/seventyGeneData.html). *TSPYL5* expression was compared between BRCA tumors and normal tissues (Wilcoxon test), and across PAM50 subtypes (Kruskal‐Wallis test). Prognostic significance for overall survival (OS), distant metastasis‐free survival (DMFS), and recurrence‐free survival (RFS) was evaluated using Kaplan‐Meier analysis (Kaplan‐Meier Plotter, survival R package) with the log‐rank test and univariate Cox regression. For pathway discovery, TCGA‐BLBC samples were stratified by *TSPYL5* expression (upper quartile). Differentially expressed genes (DEGs), identified via limma, were used for pathway enrichment (clusterProfiler), and Gene Set Variation Analysis (GSVA) was performed using MSigDB Hallmark gene sets.

Genomic alterations of *TSPYL5* in the TCGA‐BRCA cohort were analyzed using cBioPortal [19, 54]. This included assessing genomic alteration frequency and survival analysis.

### 4.2 Single‐Cell RNA‐Sequencing Data Processing and Analysis

Publicly available scRNA‐seq data were obtained from the GEO database under accession number GSE202454 [24]. The raw count matrices were processed using the Seurat package (v4.4.1) in R (v4.2.3) [55, 56]. Quality control involved filtering out cells with fewer than 250 detected genes or with a mitochondrial gene content exceeding 20%. Doublets were identified and removed using the DoubletFinder package. Data were normalized using the LogNormalize method, and highly variable genes were identified via the FindVariableFeatures function (vst method). Batch effects across samples were corrected using the Harmony algorithm (RunHarmony).

For dimensionality reduction, Principal Component Analysis (PCA) was performed on the scaled data. The top 50 principal components were used for Uniform Manifold Approximation and Projection (UMAP) and t‐Distributed Stochastic Neighbor Embedding (t‐SNE) for visualization. Unsupervised clustering was performed using the Louvain algorithm with an optimized resolution parameter. DEGs for each cluster were identified using FindAllMarkers (Wilcoxon rank‐sum test, *P*_adj < 0.05, |log2FC| > 0.25) and the COSG package. Cell types were manually annotated based on the expression of canonical marker genes (e.g., *EPCAM* for epithelial cells, *CD3D* for T cells, *COL1A1* for fibroblasts), cross‐referenced with the CellMarker and Cell Taxonomy databases. Cellular differentiation potential was assessed using CytoTRACE [57]. Gene Set Enrichment Analysis (GSEA) was performed using the fgsea package against the Hallmark gene set (MSigDB v7.4.1).

To distinguish malignant from normal epithelial cells, copy number variations (CNVs) were inferred using the copykat package [58]. A pooled population of non‐epithelial cells (immune, endothelial, and stromal cells) from each patient served as the diploid reference. For validation, CNV scores were also calculated using infercnvpy in Python after converting the Seurat object to an AnnData object via the sceasy package.

### 4.3 Spatial Transcriptomics Data Analysis

10× Genomics Visium spatial transcriptomics data were obtained from GEO (GSE176078) [24]. Data were processed using Seurat's spatial analysis pipeline [59]. Spots with fewer than 200 detected genes, 500 UMIs, or over 20% mitochondrial content were excluded. Data were normalized using SCTransform. Dimensionality reduction and clustering were performed as described for the scRNA‐seq analysis. Spatially variable features were identified using FindSpatiallyVariableFeatures. To infer the cellular composition of each spot, we performed deconvolution using CARD, with our annotated scRNA‐seq dataset as the reference [60].

### 4.4 Protein‐Protein Interaction Analysis

Pathway‐associated genes were retrieved from MSigDB using the msigdbr R package. Gene sets of interest were extracted, and duplicated genes were removed. Protein–protein interaction analysis was performed using the STRING database. Interactions among the submitted proteins were retained based on the STRING confidence score. The resulting PPI network was exported and visualized by ggraph R package. Nodes represented proteins and edges represented protein associations. Hub genes were identified according to network topology parameters, particularly node degree.

### 4.5 Cell Culture and Reagents

The human TNBC cell lines utilized in this study, MDA‐MB‐231 (RRID: CVCL_0062), HCC38 (RRID: CVCL_1267), BT‐549 (RRID: CVCL_1092), and BT‐20 (RRID: CVCL_0178), were purchased from the Shanghai Institute of Biochemistry and Cell Biology (Shanghai, China). These lines were originally established by the American Type Culture Collection (ATCC, Manassas, VA, USA). The identity of all cell lines was authenticated by short tandem repeat (STR) profiling, and all cultures tested negative for mycoplasma contamination using a PCR‐based assay. MDA‐MB‐231 and BT‐20 cells were cultured in Dulbecco's Modified Eagle Medium (DMEM), whereas HCC38 and BT‐549 cells were maintained in RPMI‐1640 medium. All media were supplemented with 10% fetal bovine serum (FBS) and 1% penicillin/streptomycin (all reagents from Gibco, Thermo Fisher Scientific, Waltham, MA, USA). HCC38 was supplemented with 10% sodium pyruvate and BT‐549 was supplemented with 10ug/mL insulin. Cells were incubated at 37°C in a humidified atmosphere containing 5% CO_2_. All experiments were performed using cells within 20 passages post‐thawing to ensure genetic stability.

### 4.6 Plasmids, Lentiviral Production, and Generation of Stable Cell Lines

The full‐length human *TSPYL5* coding sequence (NM_033512) was cloned into the GV492 lentiviral vector (GeneChem, Shanghai, China). Plasmids encoding short hairpin RNAs (shRNAs) specifically targeting *ZEB1* (shZEB1) or *USP10* (shUSP10), alongside a scrambled non‐targeting sequence as a negative control (shNC), were also utilized. The exact nucleotide sequences of all shRNAs are detailed in Table S2. For transient gene expression or knockdown, these plasmids were delivered into cells using Lipofectamine 3000 (Invitrogen, Carlsbad, CA, USA) strictly following the manufacturer's protocol, and cells were harvested at 24 to 48 h post‐transfection for subsequent downstream assays.

For stable cell line generation, lentiviral particles were produced and used to infect breast cancer cells at an optimized multiplicity of infection (MOI) in the presence of polybrene (Ubigene, Guangzhou, China). Stable cell pools overexpressing TSPYL5 (NP_277047) or harboring an empty vector control were subsequently selected and maintained in medium supplemented with 1–2 µg/mL puromycin (MedChemExpress, NJ, USA). For targeted gene depletion, *TSPYL5* knockout was achieved utilizing a lentiviral‐based CRISPR/Cas9 system (Ubigene, Guangzhou, China). Following viral transduction and antibiotic selection, single‐cell clones were isolated via standard limiting dilution into 96‐well plates. The knockout efficiency of the expanded clone was rigorously validated by Sanger sequencing of the targeted genomic locus and further confirmed by the complete loss of target protein expression via Western blot analysis.

### 4.7 Quantitative Real‐Time PCR

Total RNA was extracted using the Total RNA Kit I (Yeasen Biotech, Shanghai, China) and reverse‐transcribed into cDNA using the ExonScript RT SuperMix with dsDNase (Rongwei Gene, Chengdu, China). RT‐qPCR was performed using Hieff UNICON Universal Blue qPCR SYBR Green Master Mix (Yeasen Biotech) on a compatible real‐time PCR system. Relative gene expression was calculated using the 2‐ΔΔCt method, with *GAPDH* serving as the endogenous control. All reactions were performed in triplicate. The RT‒qPCR primers for the target genes are listed in Table S3.

### 4.8 Immunoprecipitation and Western Blot Analysis

For immunoprecipitation (IP), cells were lysed in IP buffer supplemented with protease and phosphatase inhibitors (MedChemExpress, NJ, USA). Cleared lysates were incubated overnight at 4°C with specific antibodies or control IgG, followed by a 2‐h incubation with Protein A+G Agarose beads (Beyotime, Shanghai, China) to capture immune complexes. After washing, bound proteins were eluted by boiling in SDS‐PAGE loading buffer.

For Western blotting, total lysates were also prepared using RIPA buffer. Protein concentrations were determined using a BCA Protein Assay Kit (Solarbio, Beijing, China). Equal protein amounts from total lysates or the entire IP eluate were resolved by SDS‐PAGE, transferred to PVDF membranes (Millipore, Germany), and probed with primary and HRP‐conjugated secondary antibodies after blocking with 5% non‐fat milk. Bands were visualized using an enhanced chemiluminescence (ECL) substrate and quantified with ImageJ software (NIH, Bethesda, MD, USA). GAPDH served as loading controls for total lysates. The primary antibodies used in this study are listed in Table S4.

### 4.9 Cell Proliferation Assays

Cell viability was assessed using the Cell Counting Kit‐8 (CCK‐8, Bioground, Chongqing, China) according to the manufacturer's instructions. Briefly, cells were seeded in 96‐well plates at a density of 3 × 10^3^ cells per well. After treatment for the indicated time periods (24, 48, 72, and 96 h), 10 µL of CCK‐8 solution was added to each well and incubated for 2 h at 37°C. Absorbance was measured at 450 nm using a microplate reader (ThermoForma, USA). Each experiment was performed in triplicate and repeated three times.

Cell proliferation was further evaluated by EdU incorporation using BeyoClick EdU Kit (Alexa Fluor 555, Beyotime, Shanghai, China). Cells were seeded in 12‐well plates, incubated with 10 µM EdU for 2–12 h, fixed with 4% paraformaldehyde for 30 min, permeabilized with 0.5% Triton X‐100 for 15 min, and stained with Click Reaction Solution for 30 min. After DAPI counterstaining, EdU‐positive cells were quantified from five random fields using an Olympus IX71 fluorescence microscope (Tokyo, Japan).

### 4.10 Colony Formation Assay

For analyzing the colony formation ability, cells were seeded in 6‐well plates at a density of 100–1000 cells per well and cultured for 14 days. The medium was changed every 3–4 days. At the end of the experiment, colonies were fixed with 4% paraformaldehyde for 15 min and stained with 0.1% crystal violet for 30 min. Colonies containing more than 50 cells were counted under a microscope.

### 4.11 Cell Cycle Analysis

For examining the cell cycle characterization, cells were harvested, fixed in 70% ice‐cold ethanol overnight at 4°C, and subsequently stained with PI solution (50 µg/mL PI, 200 µg/mL RNase A, 0.1% Triton X‐100 in PBS) for 30 min in the dark. After filtration through a 70 µm strainer, ≥10 000 events were analyzed using a BD FACSCalibur flow cytometer (BD Biosciences, San Jose, CA, USA). Cell cycle distribution was determined using ModFit *LT* software (BD, Topsham, ME, USA).

### 4.12 Cell Migration and Invasion Assays

A wound‐healing assay was performed to investigate cell migration. Cells were seeded in 6‐well plates and grown to 90% confluence. A sterile 200 µL pipette tip was used to create a scratch wound across the cell monolayer. Cells were washed twice with PBS to remove detached cells and then cultured in serum‐free medium. Images of the wounds were captured at 0 h, 24 h, 48 h, 72 h, and 96 h using an inverted microscope (Olympus IX71). The wound closure percentage was calculated using ImageJ software.

Cell migration and invasion were also assessed using Transwell chambers (8 µm pore size; Corning, NY, USA). For the migration assay, (1–10) × 10^4^ cells in 200 µL serum‐free medium were seeded in the upper chamber, while 600 µL medium containing 10% FBS was added to the lower chamber as a chemoattractant. After 24–72 h incubation, non‐migrated cells on the upper surface of the membrane were removed with a cotton swab. Cells that migrated to the lower surface were fixed with 4% paraformaldehyde and stained with 0.1% crystal violet. For the invasion assay, the upper chambers were pre‐coated with Matrigel (Yeasen Biotech) diluted 1:8 in serum‐free medium and allowed to solidify at 37°C for 2 h before seeding cells. The subsequent steps were identical to the migration assay. For both assays, five random fields per well were photographed under a microscope, and the number of migrated or invaded cells was counted using ImageJ software

### 4.13 Apoptosis Analysis

Apoptosis was detected using the Annexin V‐FITC/PI Apoptosis Detection Kit (US Everbright, Suzhou, China) following the manufacturer's protocol. Briefly, paclitaxel‐treated cells were harvested, washed with cold PBS, and resuspended in 1× binding buffer. Cells were then stained with 1 µL Annexin V‐APC and 2 µL PI for 15 min at room temperature in darkness. Apoptotic cells were analyzed using a flow cytometer (Beckman Coulter) within 1 h of staining, and quantified using FlowJo software.

### 4.14 Animal Models

Female NCG mice (4–6 weeks old, GemPharmatech Co., Ltd, Nanjing, China) (Animal Protocol No. GPTAP20211021‐7) were housed in an SPF facility (22 ± 2°C, 50 ± 10% humidity, 12 h light/dark cycle) with ad libitum food and water. To establish an orthotopic xenograft mouse model, MDA‐MB‐231 and BT‐549 stable transfectants were suspended in a 1:1 mixture of PBS and Matrigel. Viable cells (2 × 10^6^ in 200 µL) were maintained on ice for immediate inoculation. Mice were lightly anesthetized (intraperitoneal chloral hydrate), and positioned supine. The right fourth mammary gland was shaved, disinfected (70% ethanol), and injected subcutaneously, ensuring precise delivery into the parenchyma. Tumor growth was tracked every 2–3 days by caliper measurement of orthogonal diameters, with volume calculated as V = 0.5 × length × width^2^. Humane endpoints included tumor volume ≥1000 mm^3^, >20% body weight loss, or distress symptoms. Primary tumors and potential metastatic organs (bone, lung, liver, and brain) were harvested for histopathology or immunofluorescence analysis.

An experimental metastasis model was further used to assess the metastatic potential of TNBC cells in vivo. Briefly, MDA‐MB‐231 and BT‐549 stable transfectants (2 × 10^5^ cells) suspended in 100 µL of sterile PBS were injected into the lateral tail vein of pre‐warmed mice. The successful seeding of cells into the pulmonary circulation was confirmed immediately post‐injection by bioluminescence imaging (BLI). The subsequent development of metastatic lesions was quantified weekly using an IVIS Spectrum system (Aniview100, Photon Technology, Guangzhou, China). At experimental endpoints, distinct organs were harvested for further analysis. Investigators were blinded to the group allocations during the quantitative analysis of bioluminescence signals.

All animal procedures were conducted in accordance with the guidelines of the Experimental Animal Ethics Committee of West China Hospital in Sichuan University (Animal experimental ethics filing number: 2018026a) and in compliance with the ARRIVE guidelines 2.0 (https://arriveguidelines.org/arrive‐guidelines).

### 4.15 Hematoxylin and Eosin Staining

Harvested tissues were fixed in 10% neutral buffered formalin for 24–48 h, routinely processed, and embedded in paraffin. Tissue sections (4–5 µm) were deparaffinized in xylene and rehydrated through a graded ethanol series. For histopathological evaluation, slides were stained with Harris Hematoxylin for 5 min, differentiated in 1% acid alcohol, and blued in 0.2% ammonia water to ensure precise nuclear visualization. Sections were subsequently counterstained with Eosin Y for 1–2 min. Finally, the slides were rapidly dehydrated, cleared, and permanently mounted using a neutral resinous medium. Histological images were captured using a high‐resolution microscope (DMi8; Leica Microsystems, Wetzlar, Germany), and the histopathological features were independently evaluated by two experienced pathologists.

### 4.16 Immunohistochemistry

Deparaffinized sections (4 µm) underwent antigen retrieval in sodium citrate buffer (pH 6.0), followed by endogenous peroxidase blocking (3% H_2_O_2_, 15 min). After blocking with 5% normal goat serum, sections were incubated overnight at 4°C with primary antibodies against primary antibodies [Ki67, TSPYL5, pAKT, ZEB1] (dilutions 1:100–1:500). HRP‐conjugated secondary antibodies and DAB substrate were used for visualization. Sections were counterstained with hematoxylin. IHC score was determined using a semi‐quantitative approach combining staining intensity (0–3: none, weak, moderate, strong) and percentage of positive cells (0–4: 0%, 1%–25%, 26%–50%, 51%–75%, 76%–100%). The final score (0–12) was calculated as the product of these parameters, with expression levels categorized as negative (0), weak (1–4), moderate (5–8), or strong (9–12). All slides were independently evaluated by two expert pathologists to ensure objectivity.

### 4.17 TUNEL Assay

Apoptotic cells in tissue samples were detected using an In Situ Cell Death Detection Kit (Roche Kaiseraugst, Switzerland). After permeabilization with proteinase K, sections were incubated with TUNEL reaction mixture (1 h, 37°C) and counterstained with DAPI. Images were acquired using a Leica TCS SP8 confocal microscope (Wetzlar, Germany). The apoptotic index was calculated as the percentage of TUNEL‐positive cells relative to total DAPI‐positive cells in five random high‐power fields per section.

### 4.18 RNA‐Seq and Transcriptomic analysis

Total RNA was isolated in biological triplicate or quadruplicate from MDA‐MB‐231 cells stably overexpressing TSPYL5 and corresponding empty vector control cells using TRIzol reagent (Life Technologies, CA, USA). RNA integrity and concentration were validated using an Agilent 2100 Bioanalyzer (Agilent, CA, USA). Library preparation and subsequent paired‐end sequencing on an Illumina HiSeq4000 platform were performed by LC‐Bio Technologies (Hangzhou, China).

Raw reads were processed and aligned, and differential gene expression analysis was conducted using the DESeq2 R package. Genes were considered significantly differentially expressed based on thresholds of an adjusted *p*‐value < 0.05 and an absolute log2(Fold Change) ≥ 1. Functional annotation of the resulting gene set was performed through Gene Ontology (GO) and Kyoto Encyclopedia of Genes and Genomes (KEGG) pathway enrichment analyses using the clusterProfiler R package.

### 4.19 Deubiquitinase Activity Assay

Deubiquitinase (DUB) activity was quantified using Internally Quenched Fluorescence‐Diubiquitin (IQF‐DiUb) substrates (LifeSensors, Inc., Malvern, PA, USA). This assay relies on a diubiquitin molecule containing a FRET pair, where a fluorescent reporter (TAMRA) and a quencher are attached to separate ubiquitin units linked by a specific isopeptide bond. Cleavage of the bond by a DUB separates the fluorophore from the quencher, producing a measurable increase in fluorescence. All assays were conducted at room temperature (20°C–25°C) in 96‐well black assay plates and protected from light. Reactions were initiated by mixing the DUB with the IQF‐DiUb substrate in an assay buffer composed of 50 mM Tris, pH 8.0, 0.05% CHAPS, and 10 mM DTT. The final concentration of the DiUb substrate was 200 nM, while DUBs were tested at concentrations ranging from 10 nM to 200 nM. Fluorescence was immediately monitored kinetically for 30–60 min using a plate reader with excitation at 540 nm and emission at 580 nm. Reactions lacking enzyme were included as a negative control, and USP2 core was used as a positive control enzyme.

### 4.20 Statistical Analysis

All statistical analyses were performed using GraphPad Prism software (version 8.0; GraphPad Software, San Diego, CA, USA) and R software (version 4.2.3). Prior to statistical testing, the assumptions of the specific tests were validated: normality of data distribution was evaluated using the Shapiro‐Wilk test, and homogeneity of variances was assessed using the F‐test or Brown‐Forsythe test. Quantitative data are presented as the mean ± standard deviation (SD) for continuous variables. The specific sample size (n) for each statistical analysis, representing independent biological replicates or individual animals, is indicated in the respective figure legends. To assess significant differences, a two‐tailed, unpaired Student's *t*‐test was used for comparisons between two independent groups that met normal distribution and equal variance criteria. Non‐parametric data between two groups were analyzed using the Wilcoxon rank‐sum test. For comparisons involving three or more groups, a one‐way analysis of variance (ANOVA) was employed for normally distributed data, followed by Tukey's or Dunnett's post hoc test for multiple comparisons to adjust the alpha level. Conversely, for non‐parametric data involving three or more groups, the Kruskal‐Wallis test was applied, followed by Dunn's post hoc test. Survival outcomes were estimated by the Kaplan‐Meier method and analyzed using the log‐rank test. Correlations between variables were determined using Spearman's rank correlation test. Categorical variables were evaluated utilizing Fisher's exact test or Pearson's Chi‐square test. Receiver operating characteristic curve analysis was utilized to assess the diagnostic/prognostic value, with the area under the curve calculated. All statistical tests performed were two‐sided, and an alpha level of 0.05 was established a priori. A *p* value of < 0.05 was considered statistically significant.

## Author Contributions

Y.Q.L. and Y.Y. conceived, designed, and supervised the study. J.Y.S. and S.Y.X. conducted the laboratory experiments. M.Y. performed the bioinformatic analysis. Y.Q.L., Y.Y., S.Y.X., Z.K.W., X.Y.Z., Y.W.Z., and R.T. analyzed and interpreted the data. J.Y.S., M.Y., and Y.Q.L. drafted the original manuscript. Y.Q.L. and Y.Y. revised the manuscript. All authors read, contributed to the editing of, and approved the final version of the manuscript.

## Funding

This work was supported by grants from the National Natural Science Foundation of China (81871203) and the Sichuan Provincial Natural Science Foundation(No. 2025ZNSFSC0564).

## Ethics Statement

This study was approved by the Ethics Committee of West China Hospital in Sichuan University (Ethics No. 2018026a). All methods were performed in accordance with the relevant guidelines and regulations. The informed consent for breast cancer tissues was obtained from the patients or their legal guardians.

## Conflicts of Interest

The authors declare no conflict of interest.

## Supporting information




**Supporting File 1**: advs75960‐sup‐0001‐SuppMat.docx.

**Supporting File 2**: advs75960‐sup‐0002‐Data.pdf.

## Data Availability

The data that support the findings of this study are available from the corresponding author upon reasonable request.
