# Supplementary material for: TSPYL5 Promotes Triple‐Negative Breast Cancer Metastasis by Antagonizing USP10‐Mediated PTEN Stabilization to Unleash a ZEB1‐Dependent EMT Program
Source: Adv Sci (Weinh). 2026 Jun 4;13(48):e20273. doi: 10.1002/advs.202520273 (PMC13336384; doi:10.1002/advs.202520273)

Figure 6E

FN1

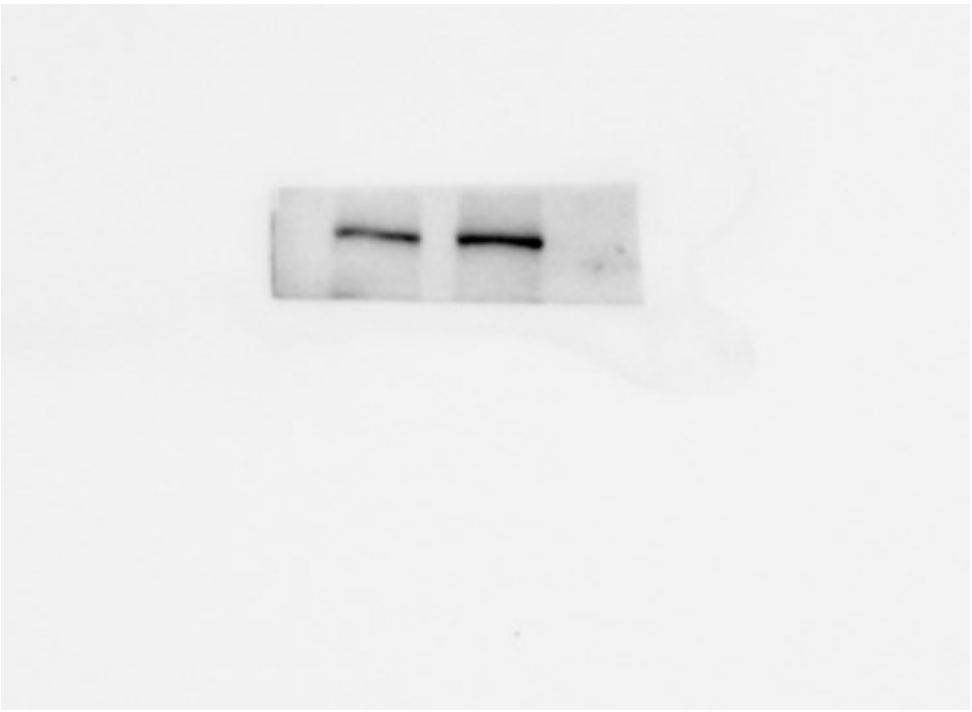

N-cadherin

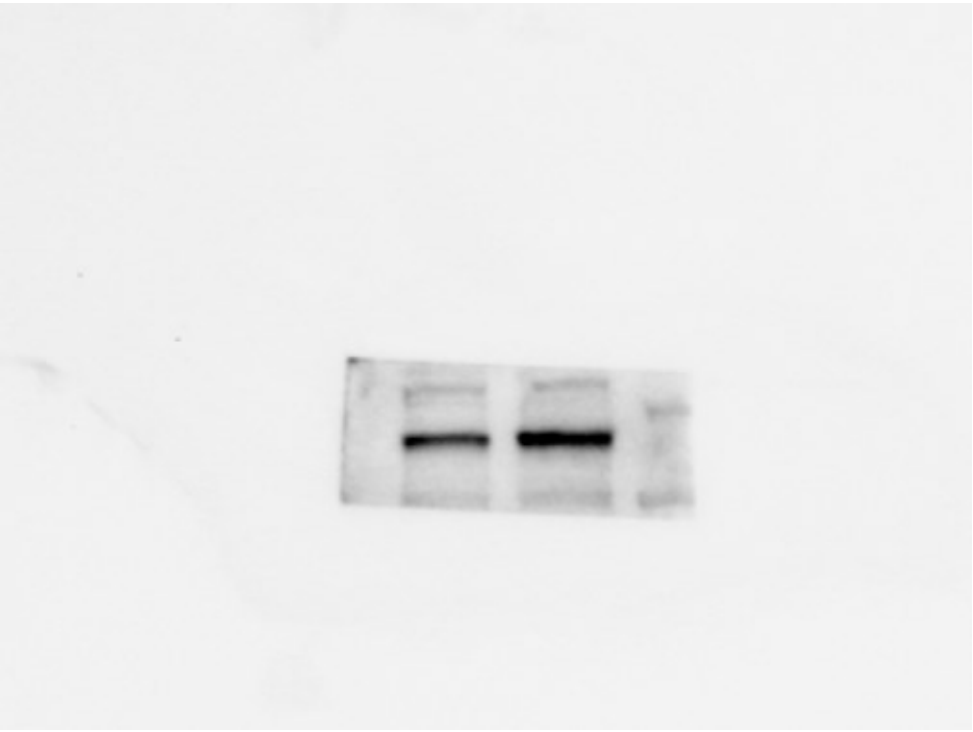

E-cadherin

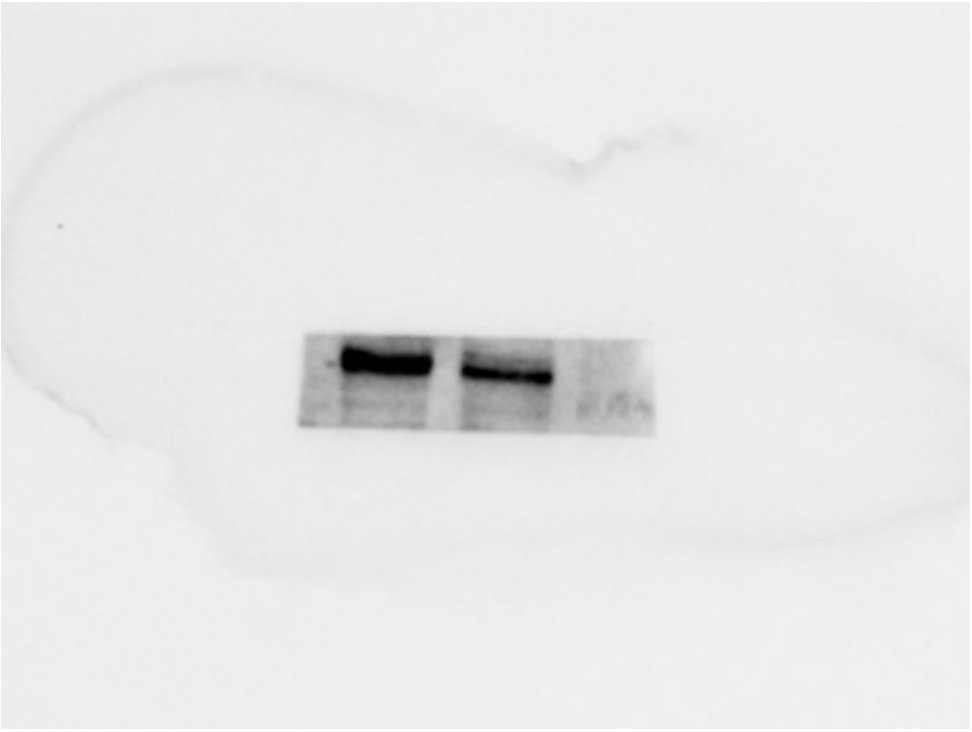

TSPYL5

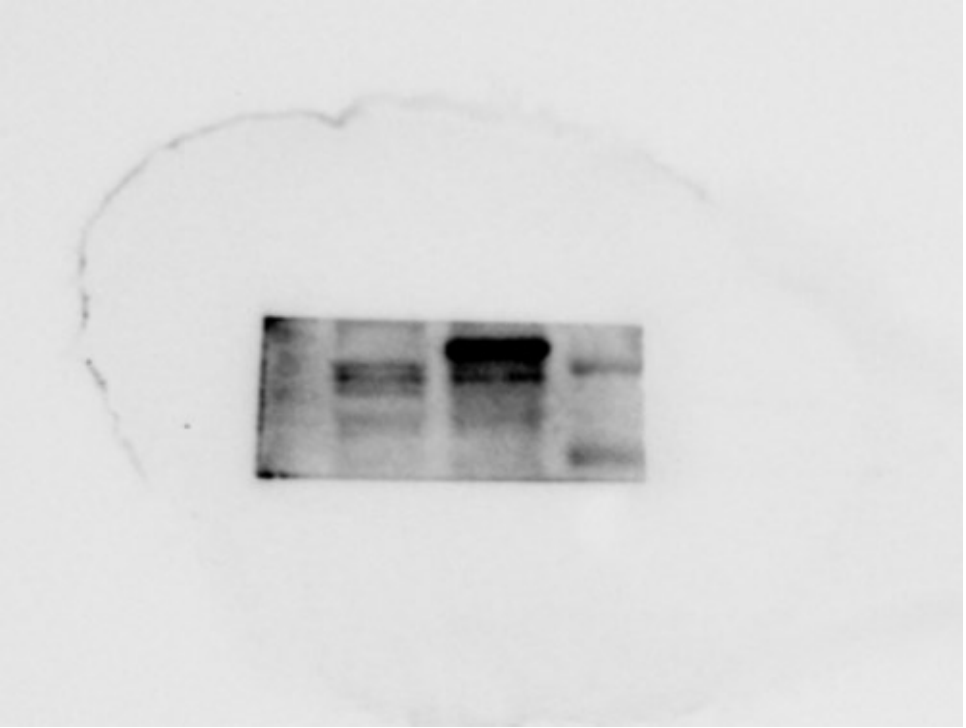

ZEB1

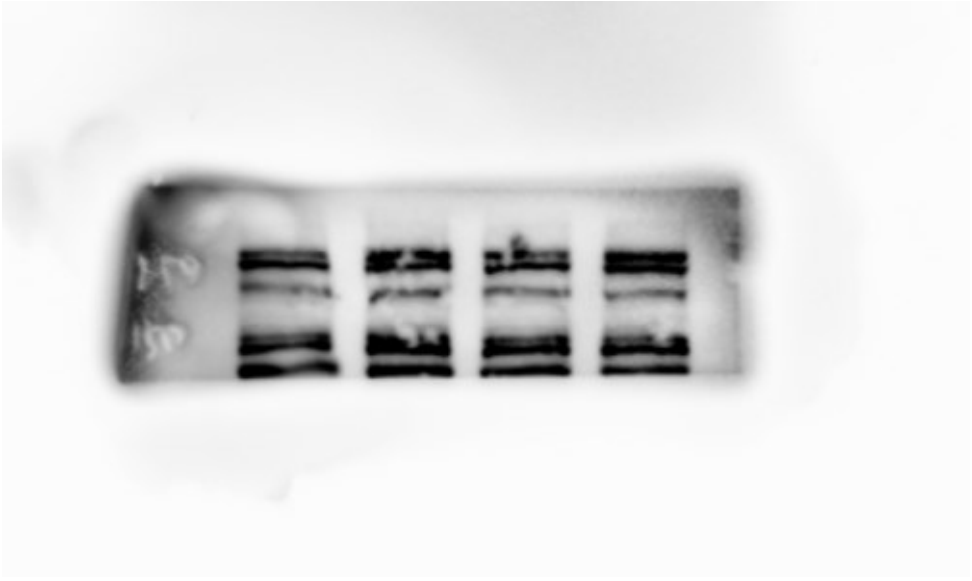

GAPDH

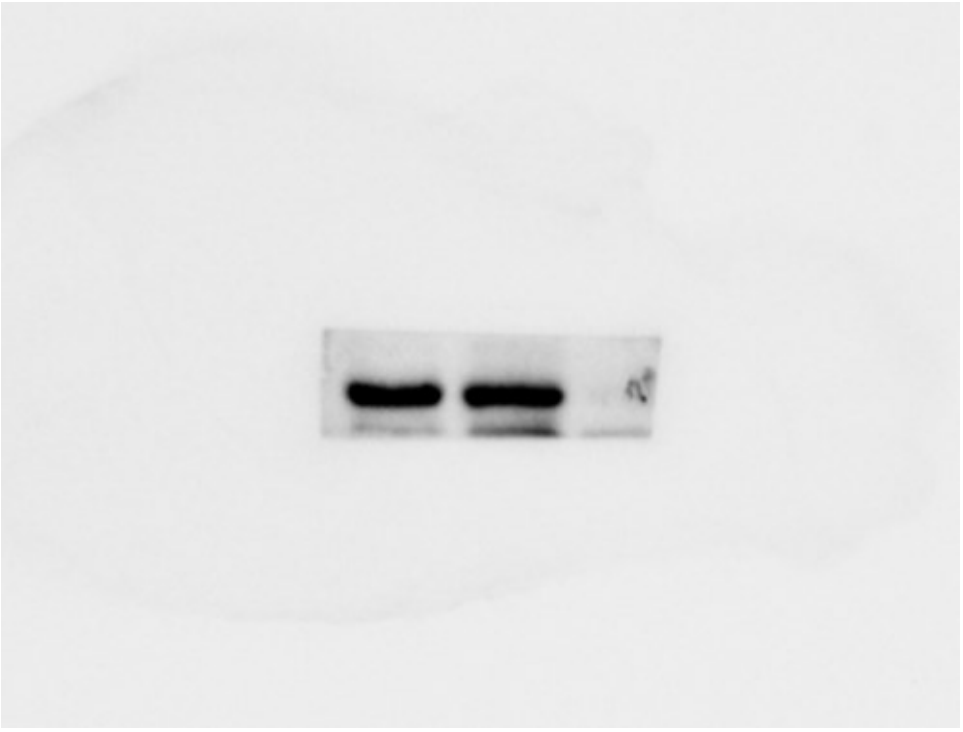

Figure 6F

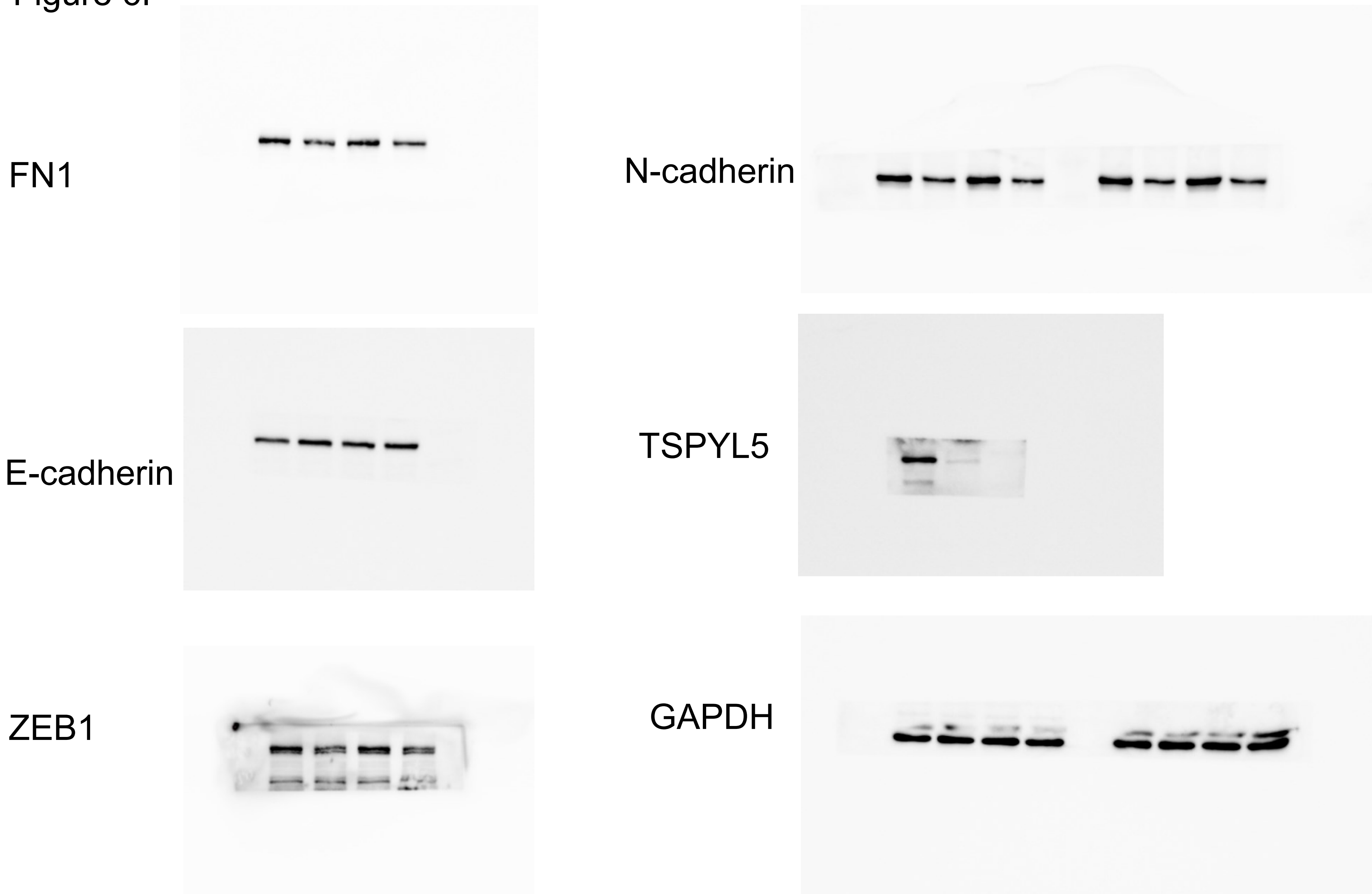

Figure 6K

ZEB1

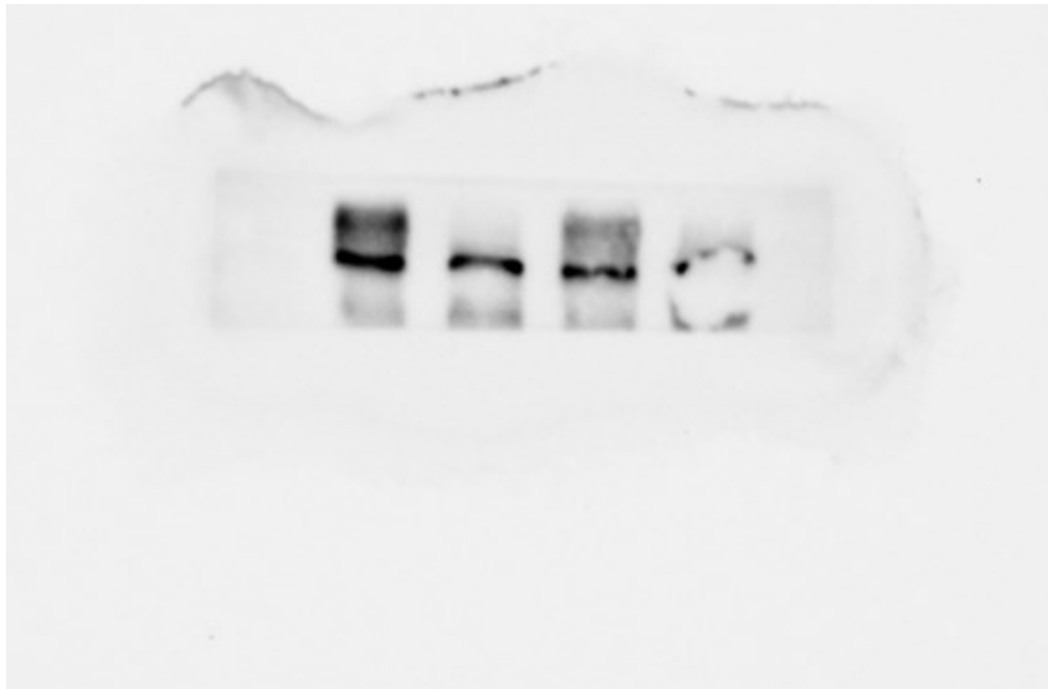

GAPDH

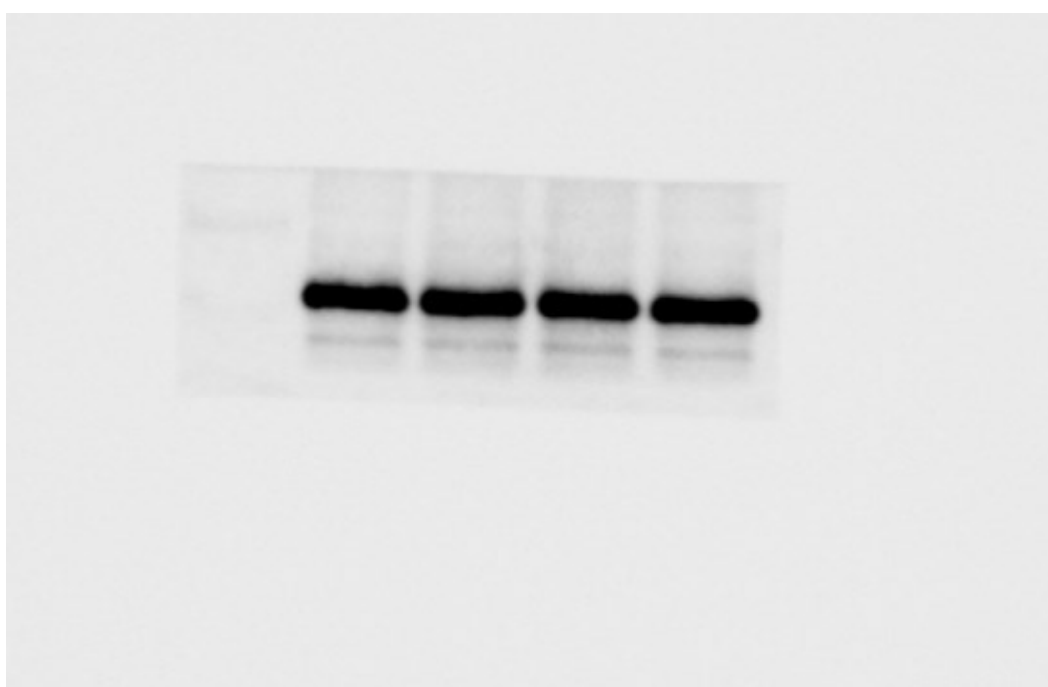

Figure 6L

N-cadherin

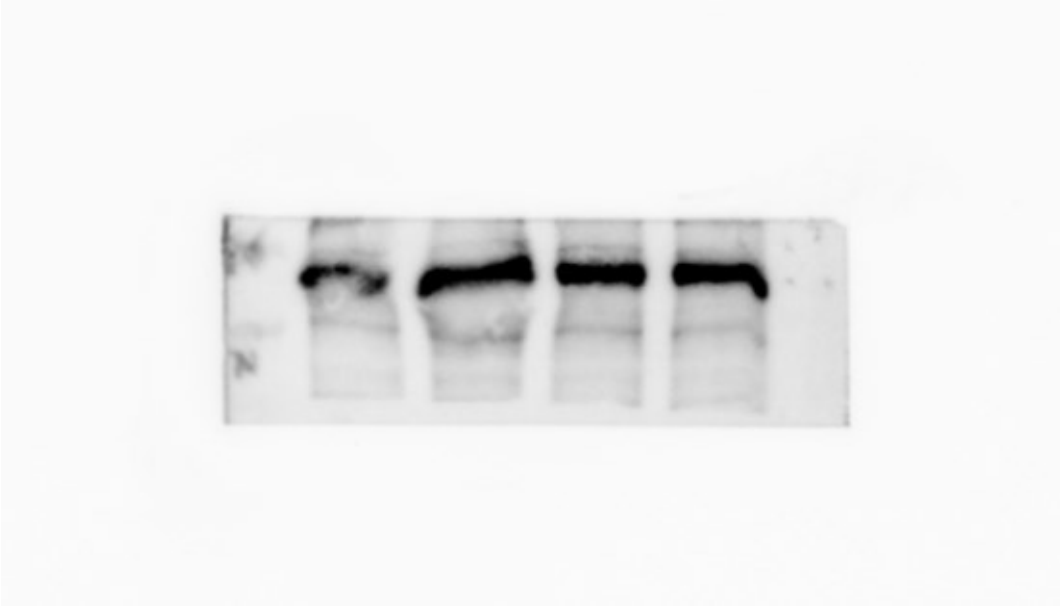

TSPYL5

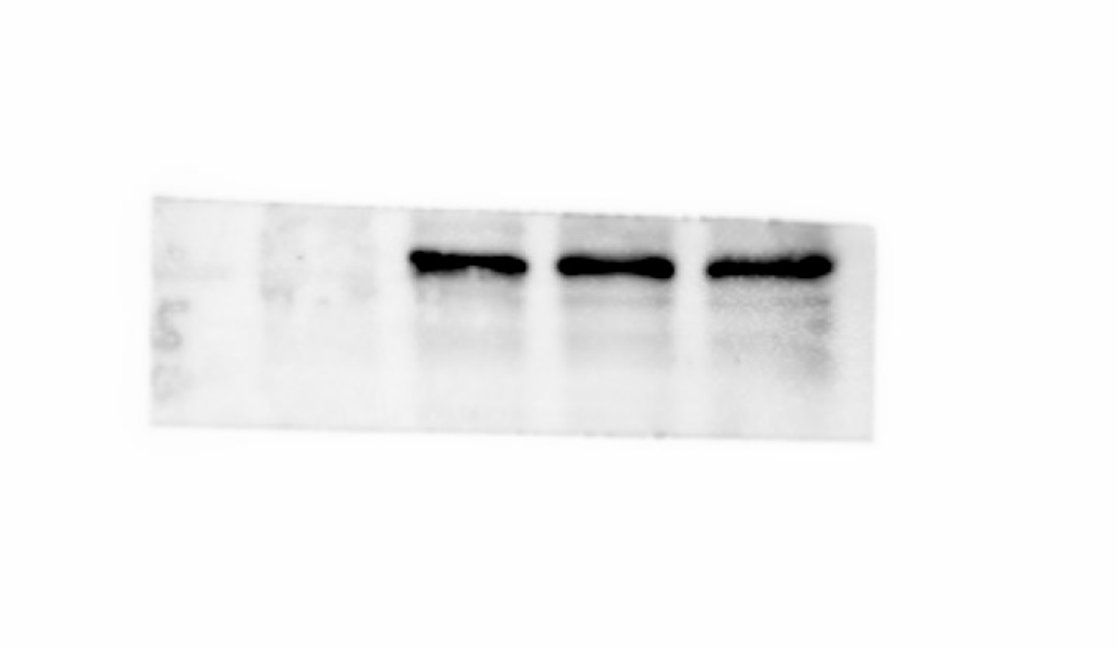

E-cadherin

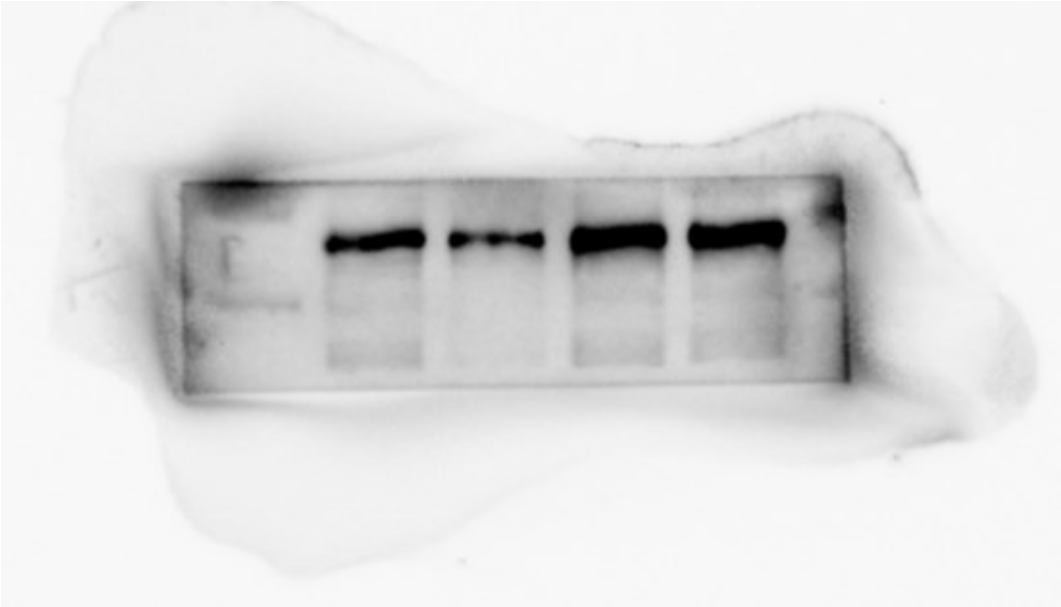

GAPDH

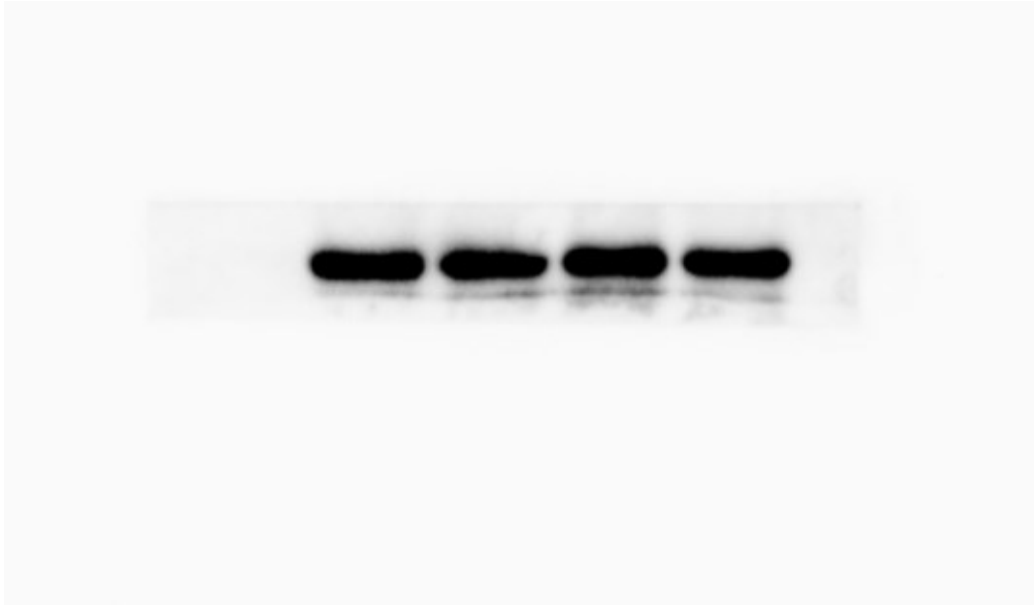

ZEB1

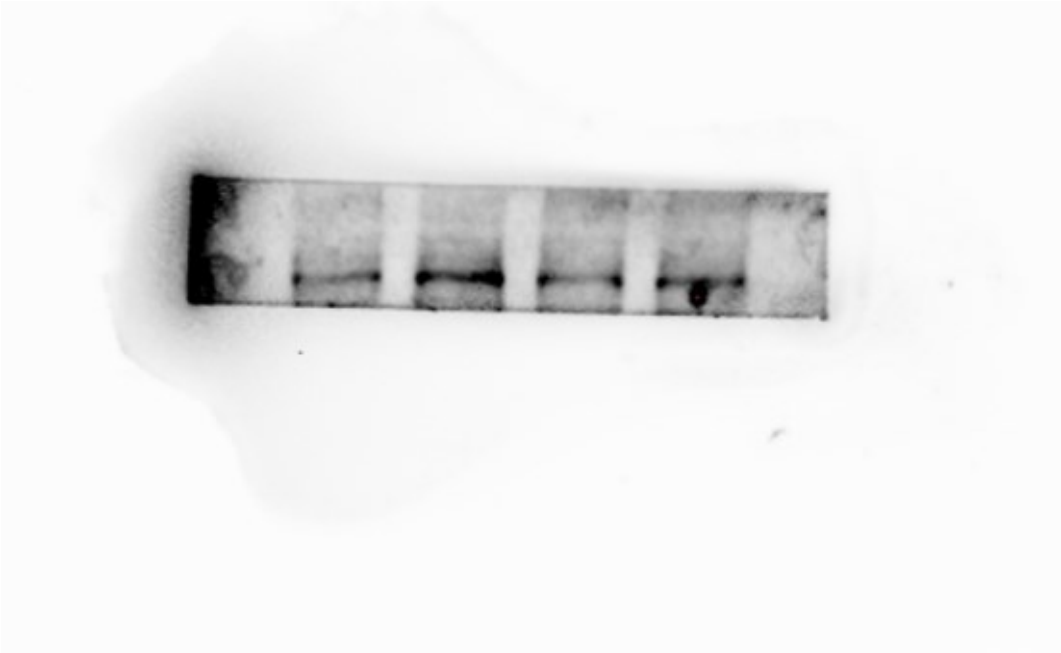

Figure 6M

N-cadherin

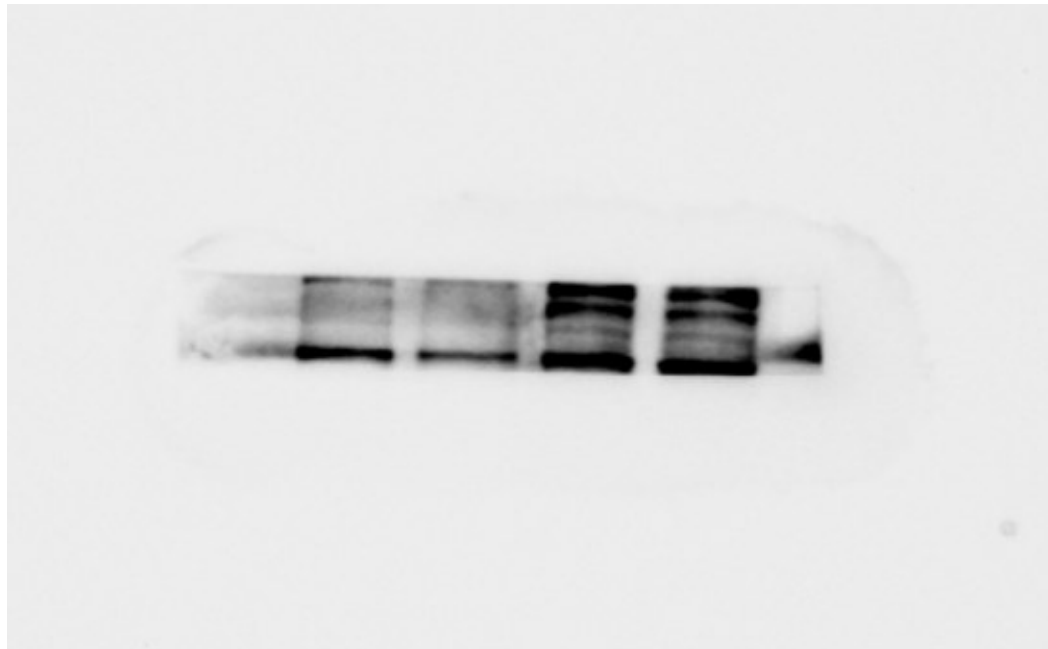

TSPYL5

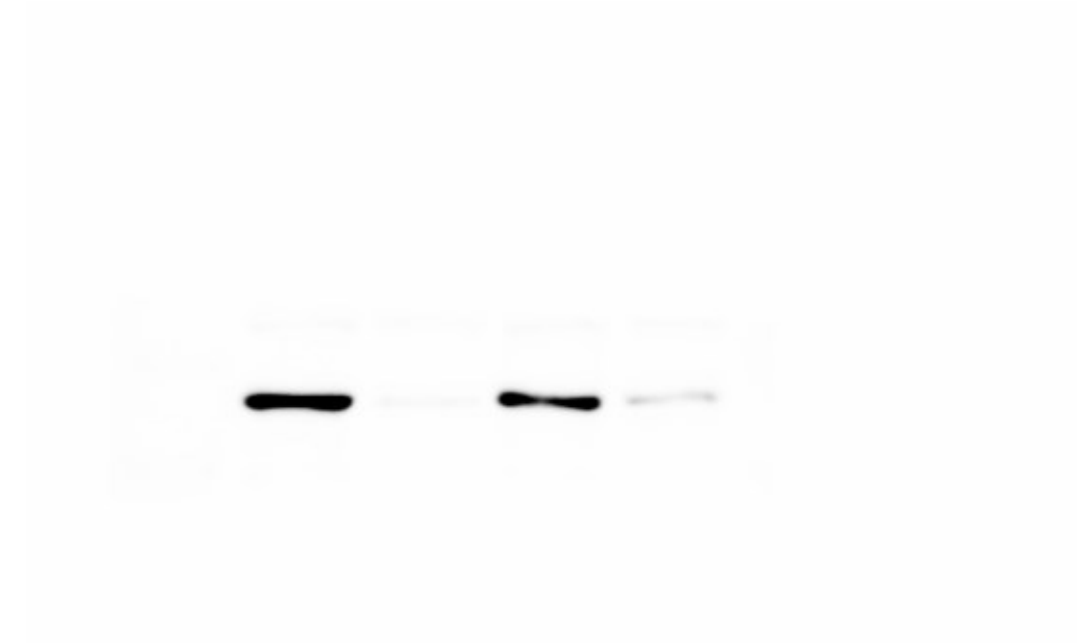

E-cadherin

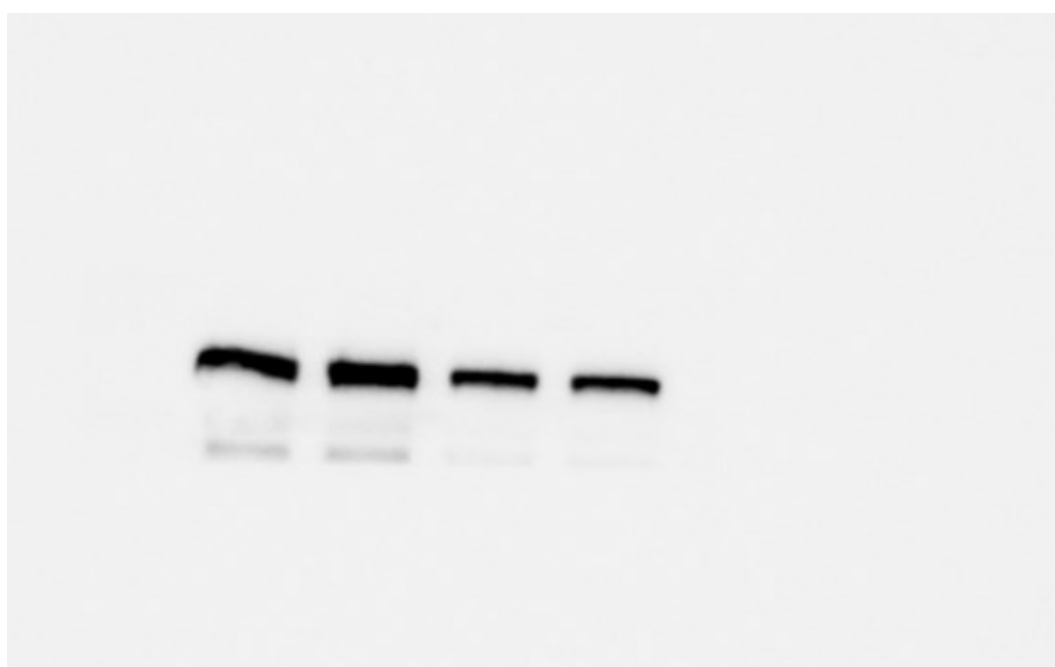

GAPDH

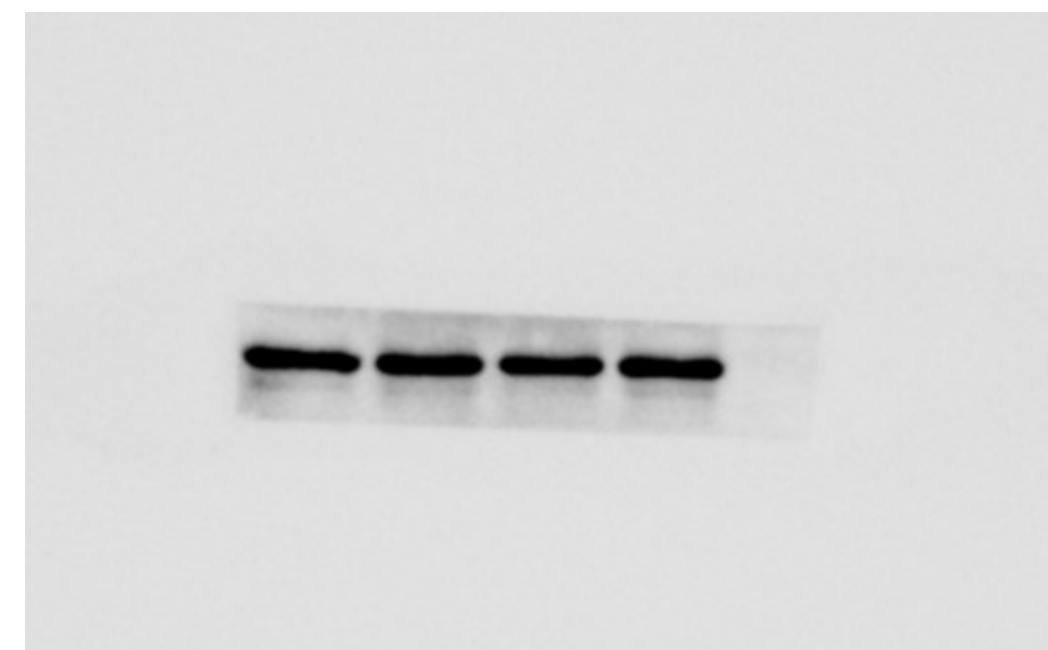

ZEB1

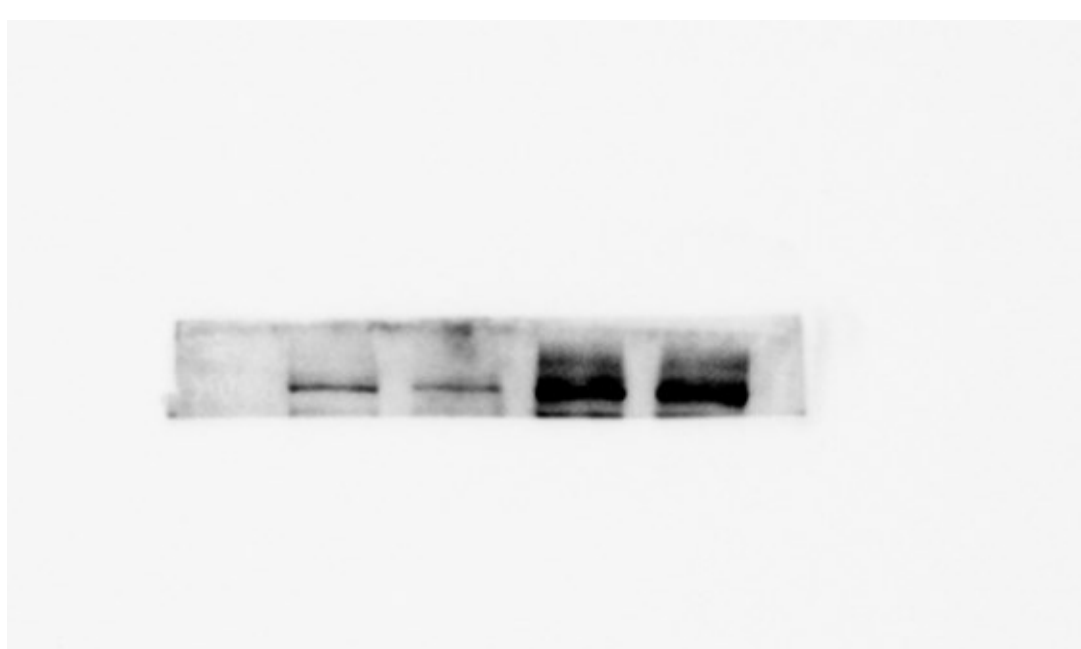

Figure 7C MDA-MB-231

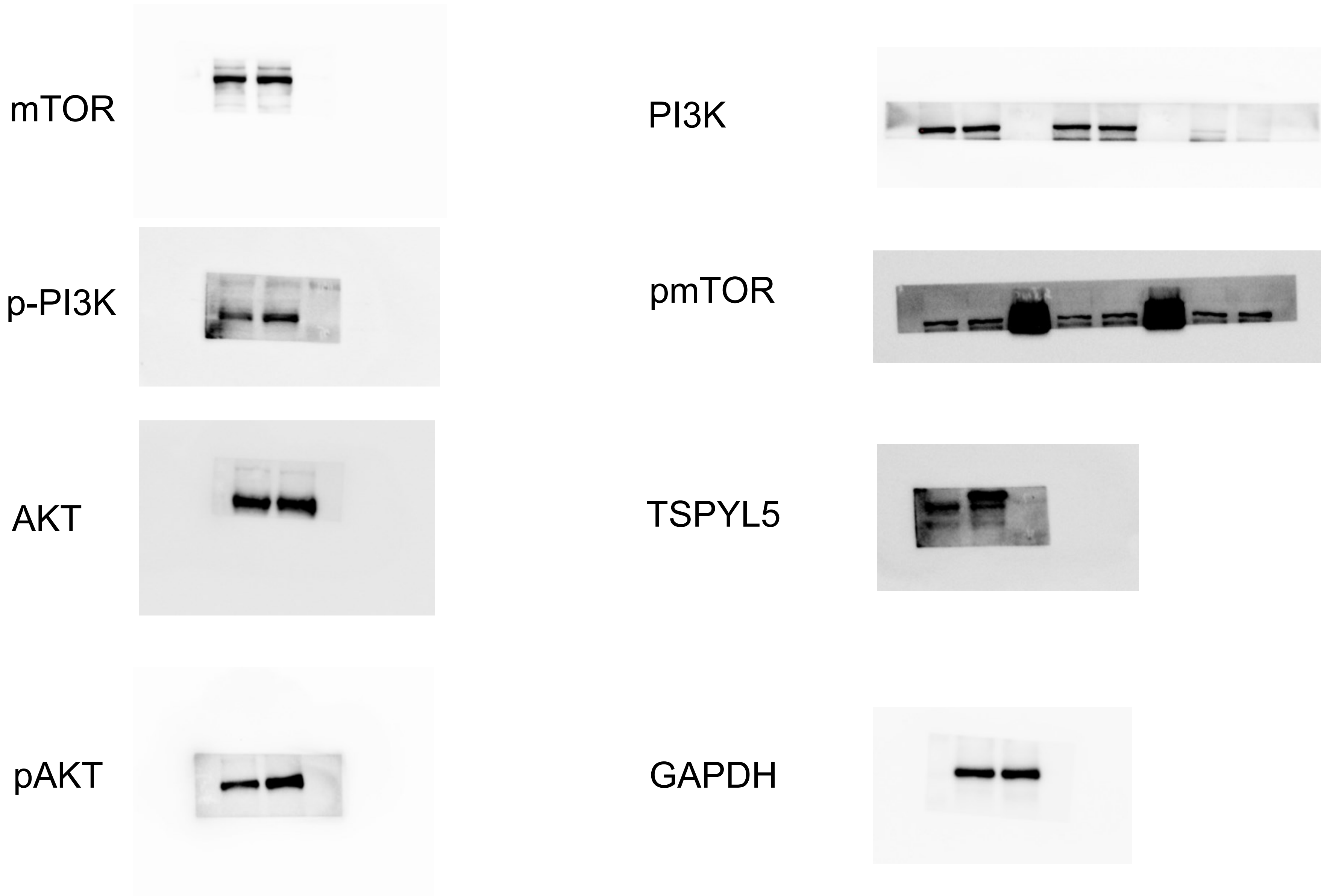

Figure 7C HCC38

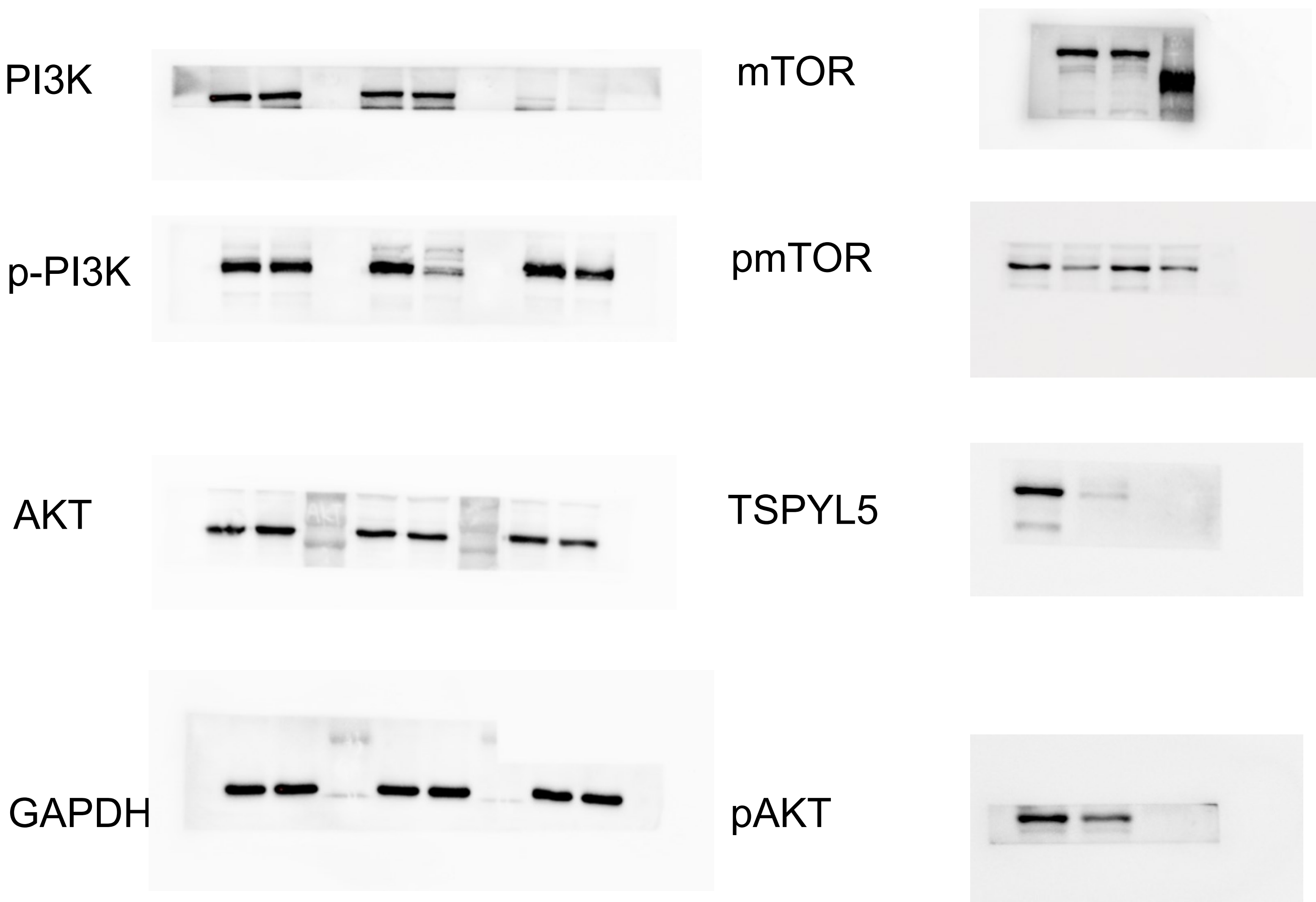

Figure 7E

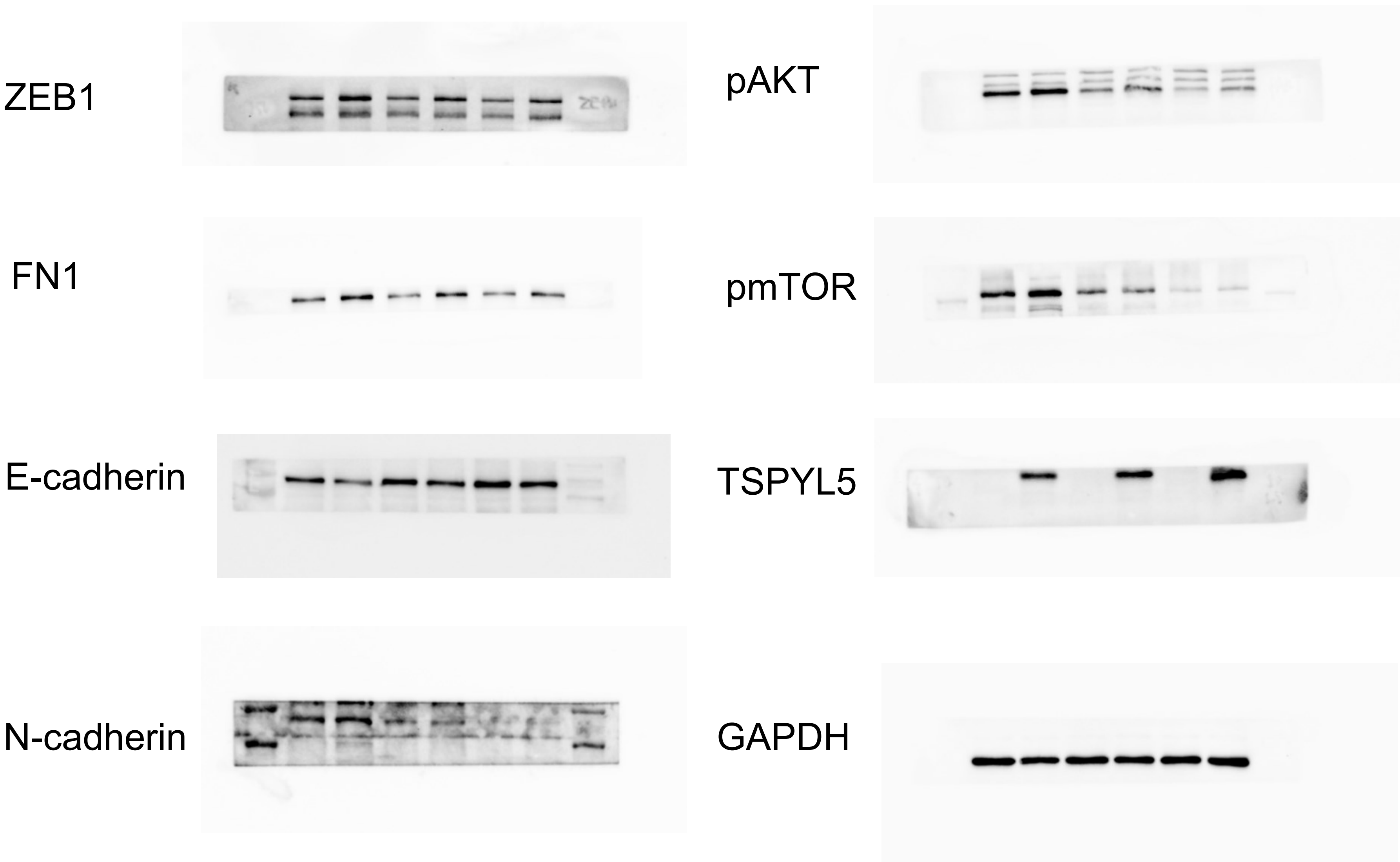

Figure 7F

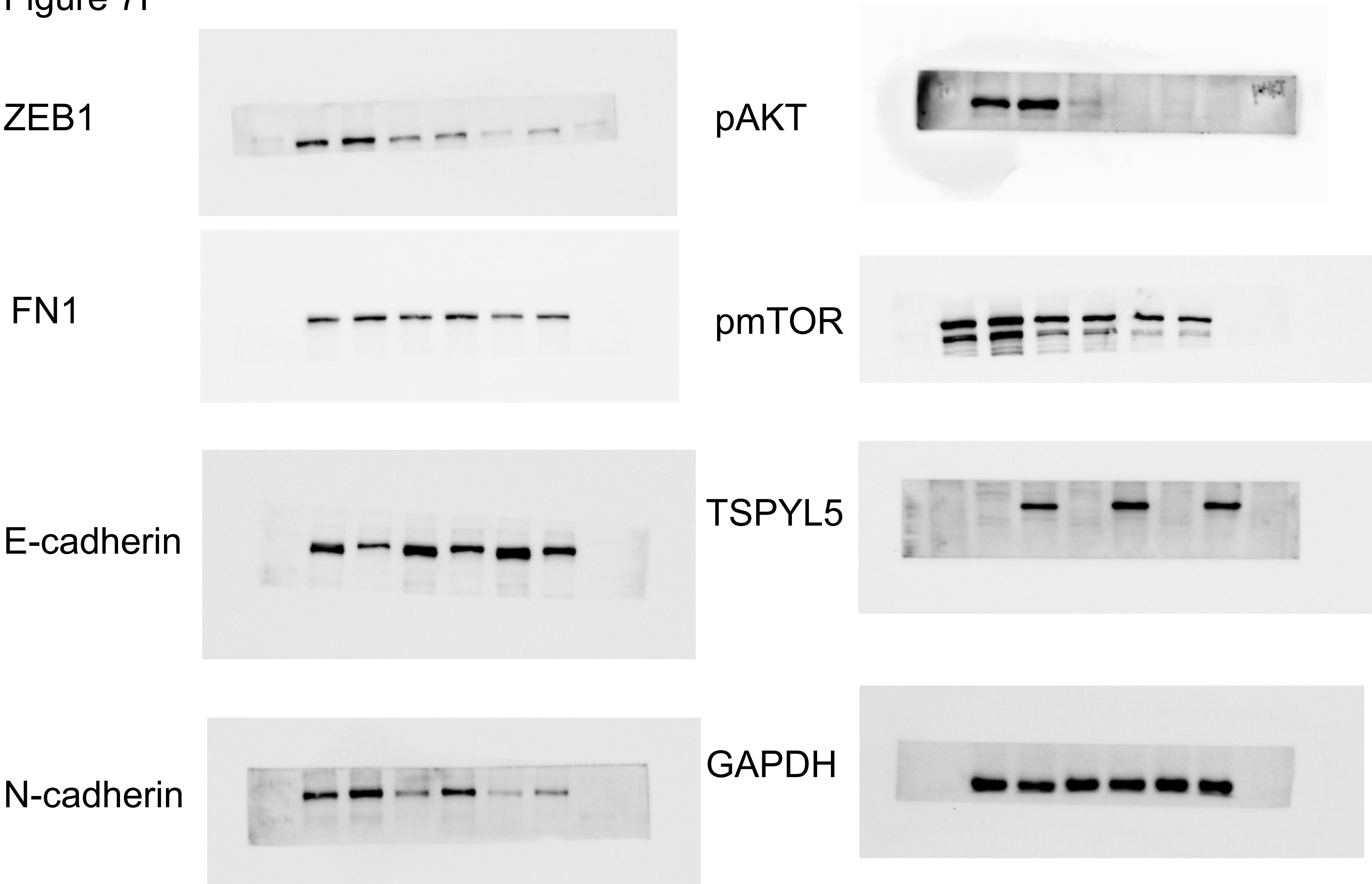

Figure 7G

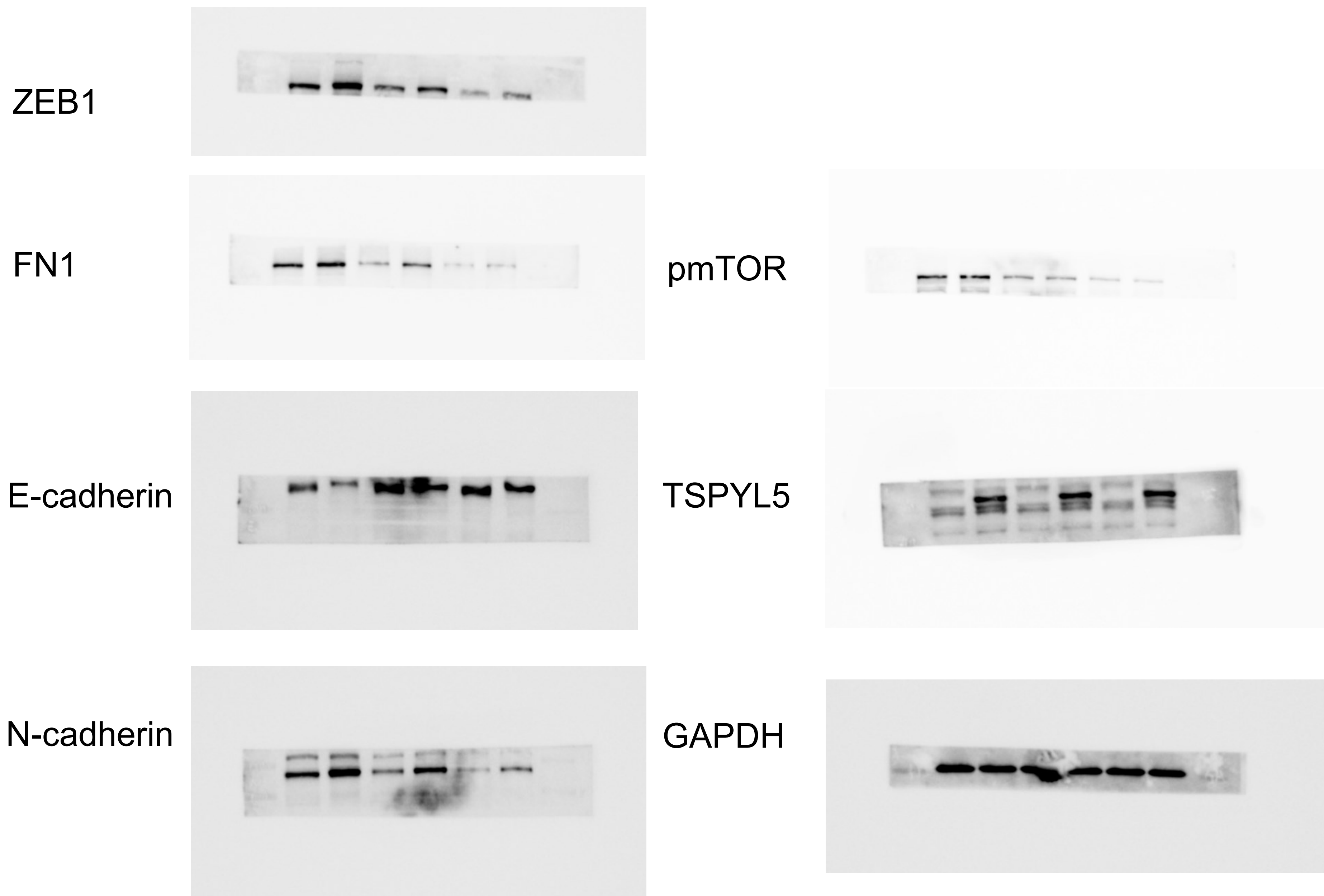

Figure 8A

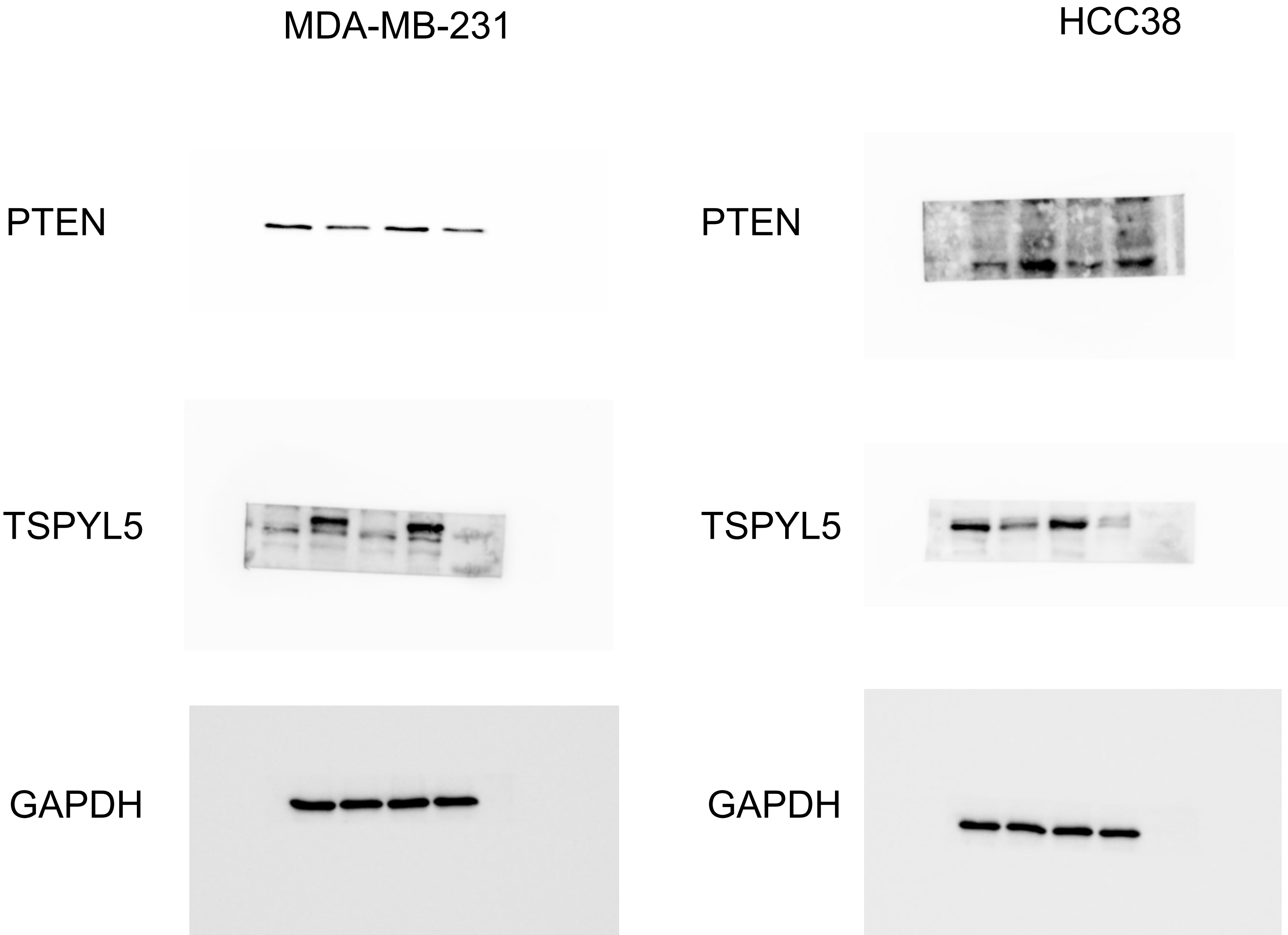

Figure 8B

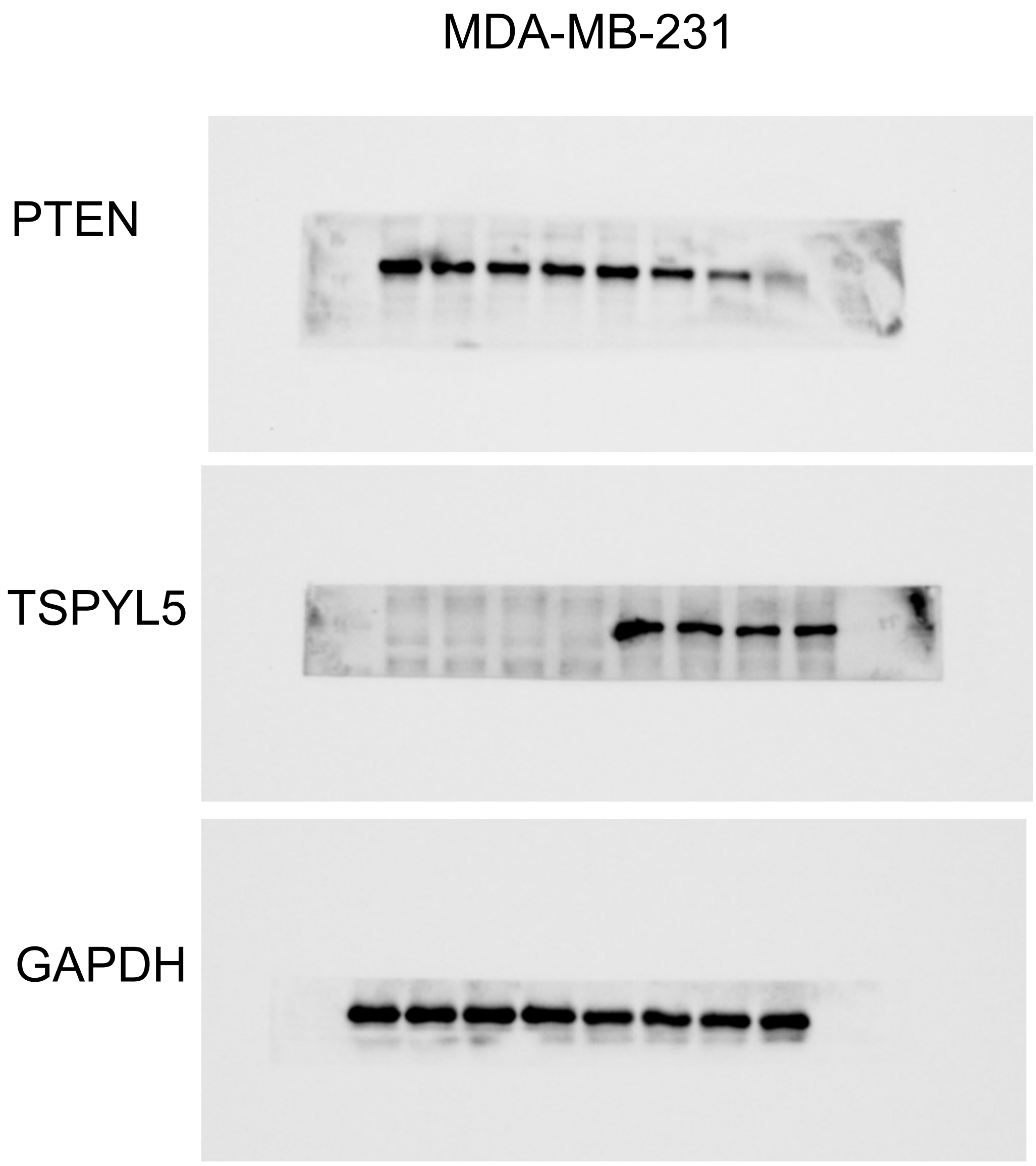

Figure 8C

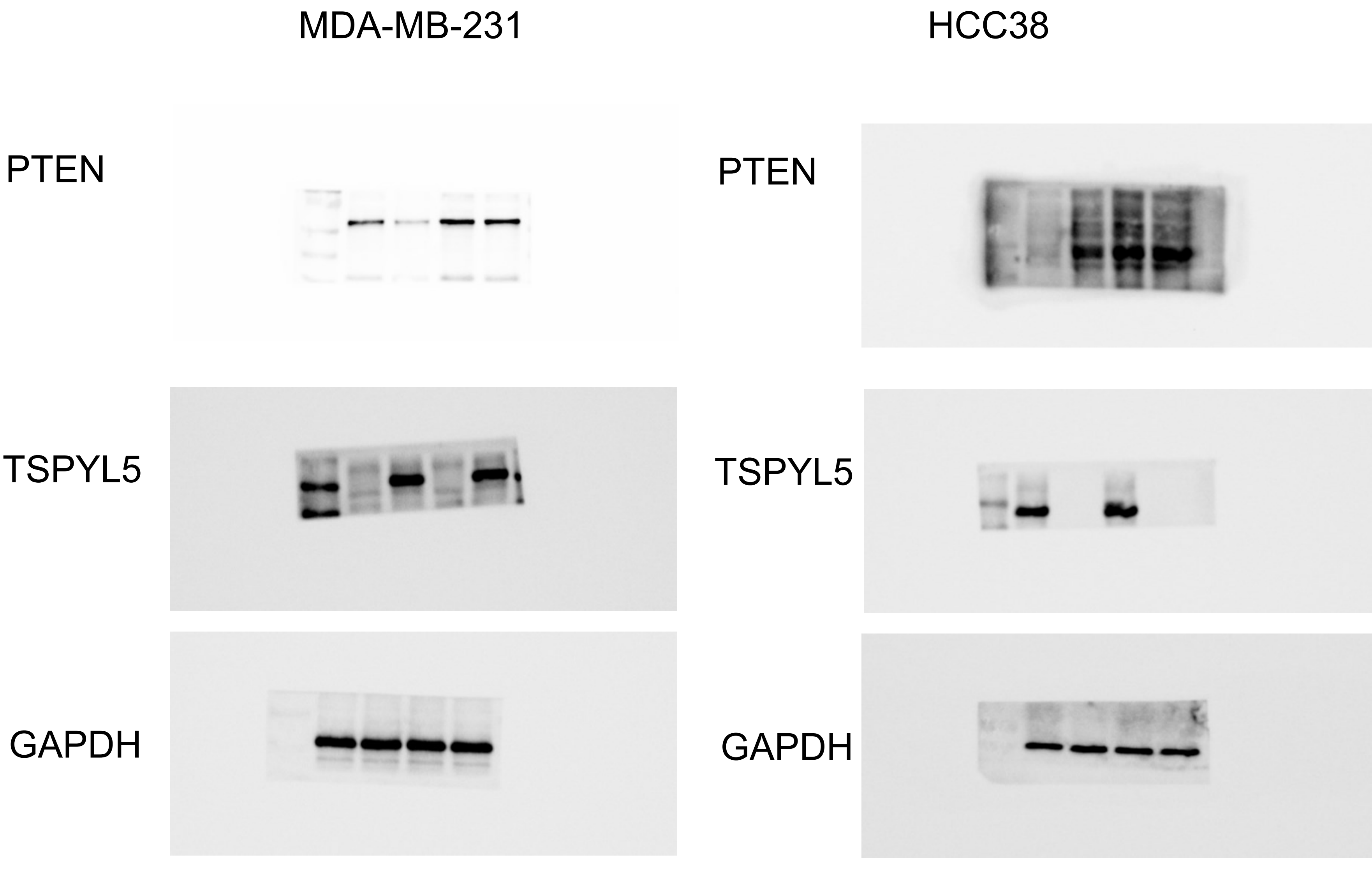

Figure 8D MDA-MB-231

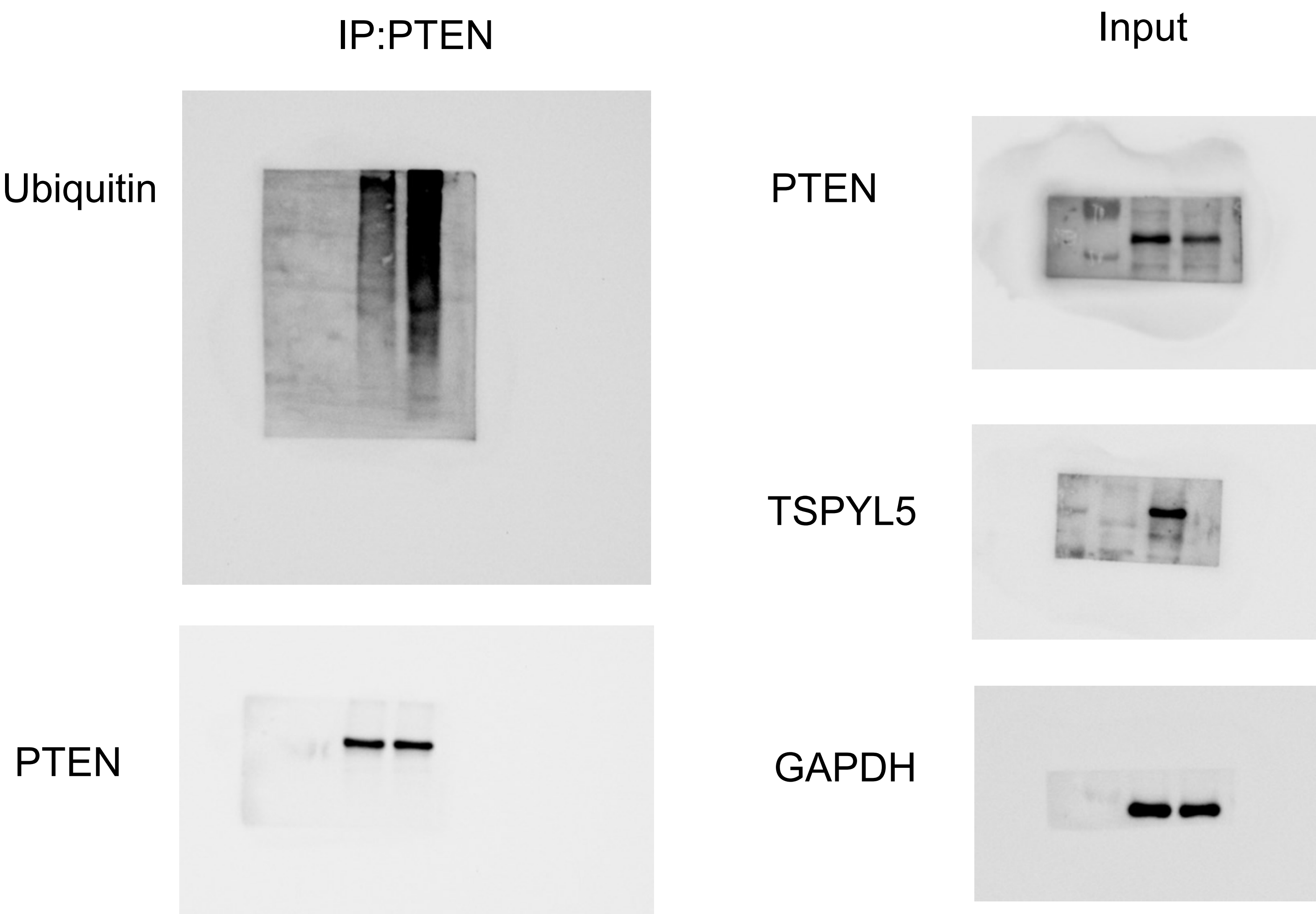

Figure 8E HCC38

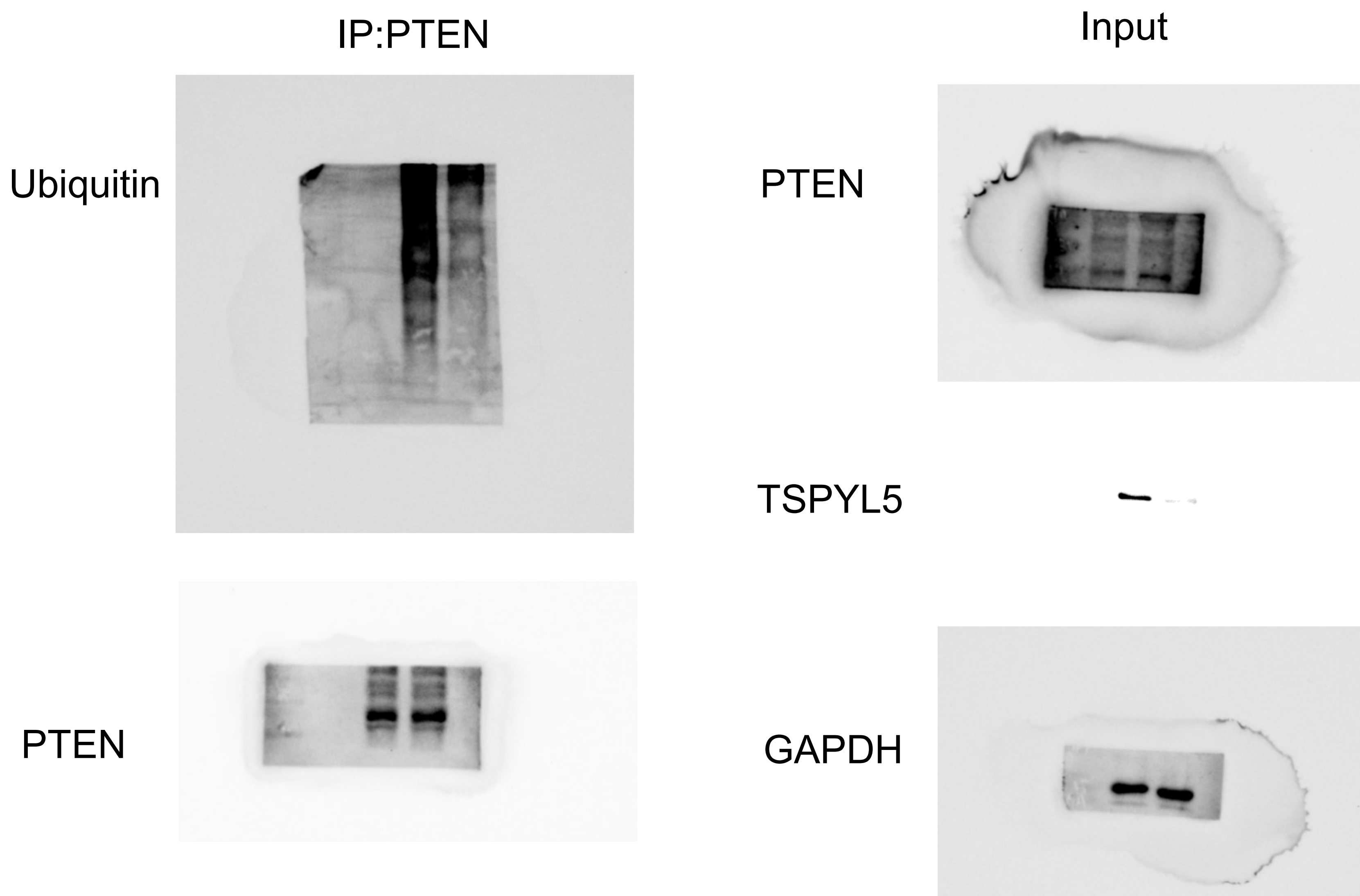

Figure 8F HCC38

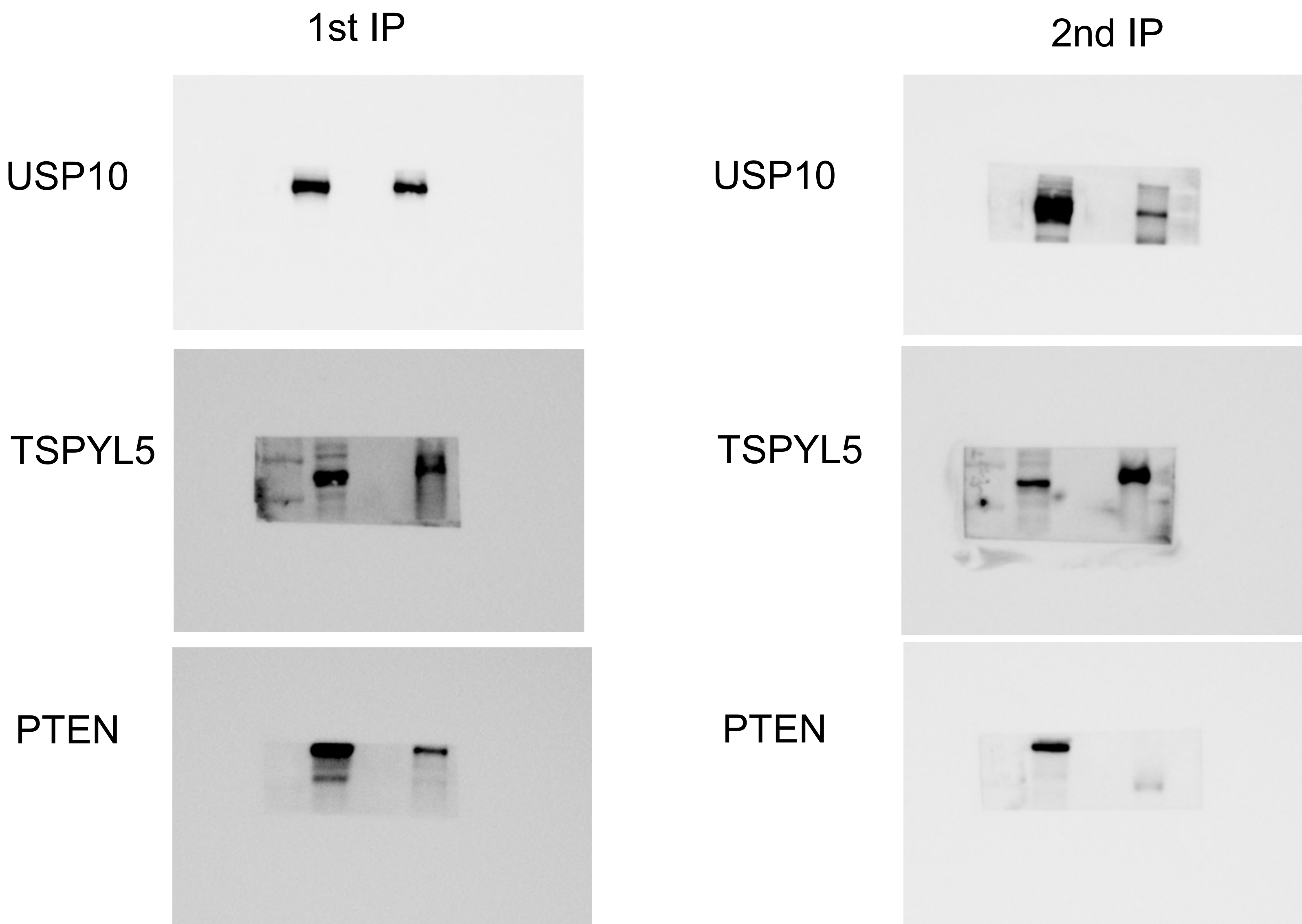

Figure 8G

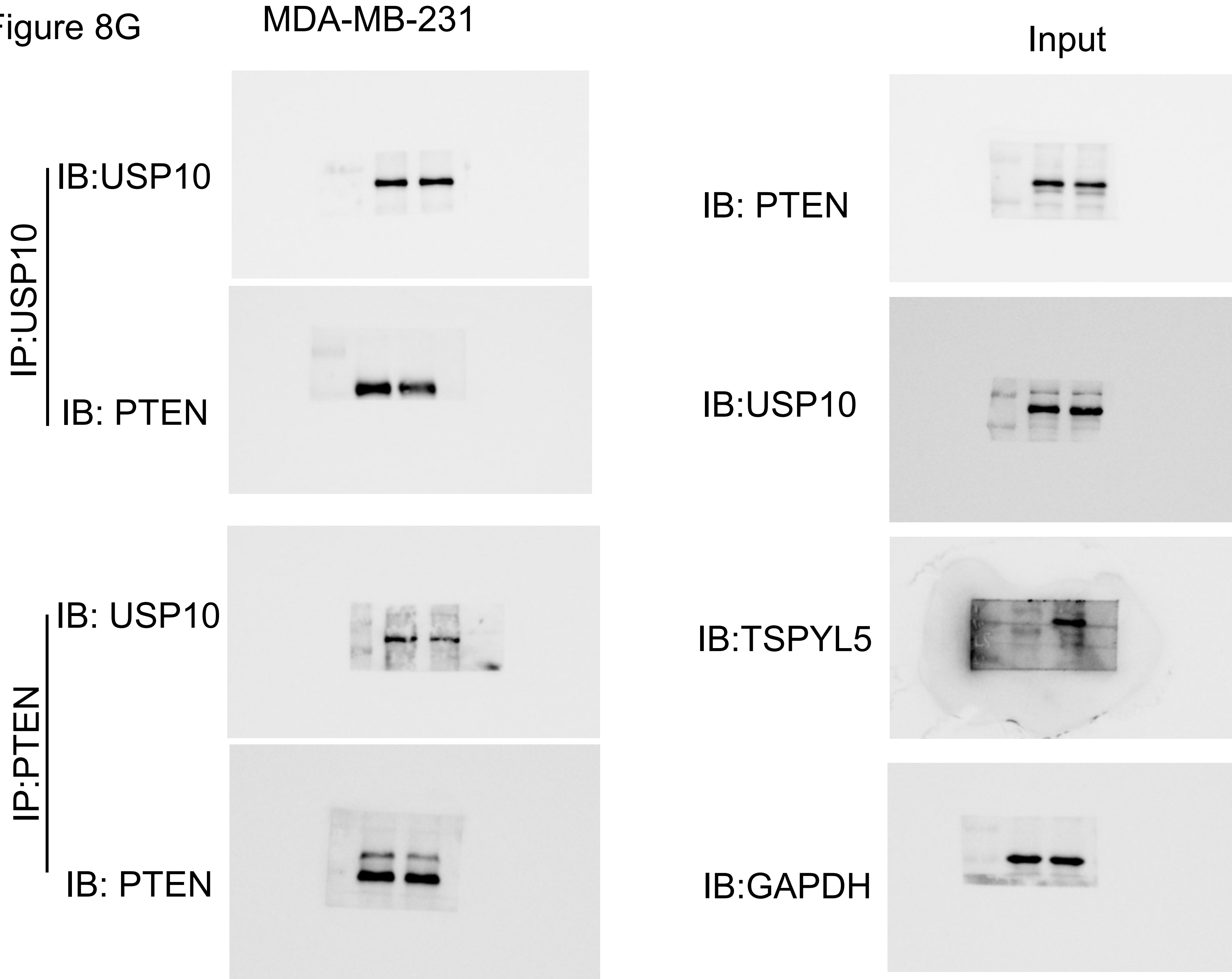

MDA-MB-231
IP:USP10
IB:USP10
IB: PTEN
IP:PTEN
IB: USP10
IB: PTEN
Input
IB: PTEN
IB:USP10
IB:TSPYL5
IB:GAPDH

Figure 8H

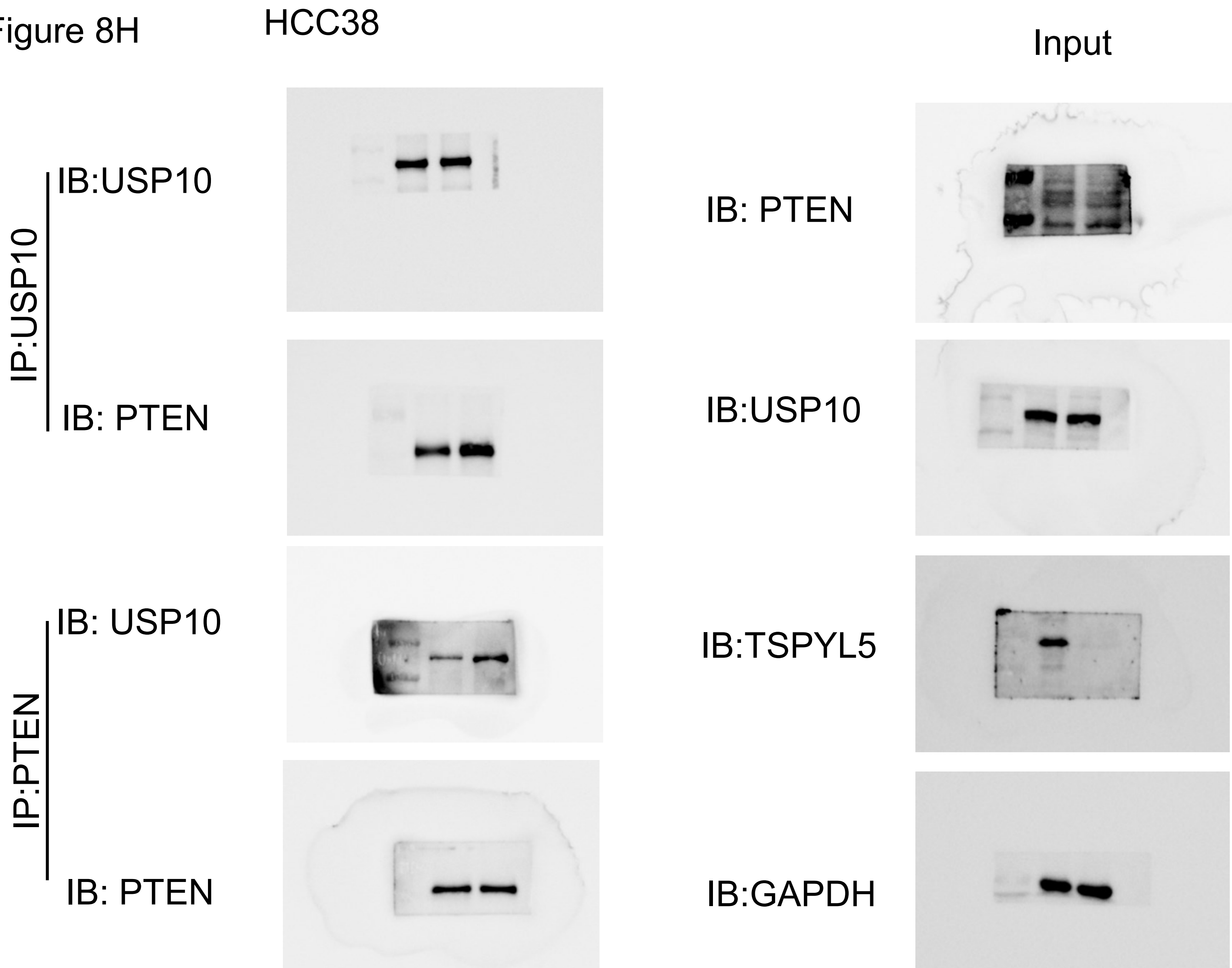
HCC38
Input
IP:USP10
IB:USP10
IB: PTEN
IP:PTEN
IB: USP10
IB: PTEN
IB: PTEN
IB:USP10
IB:TSPYL5
IB:GAPDH

Figure 8J

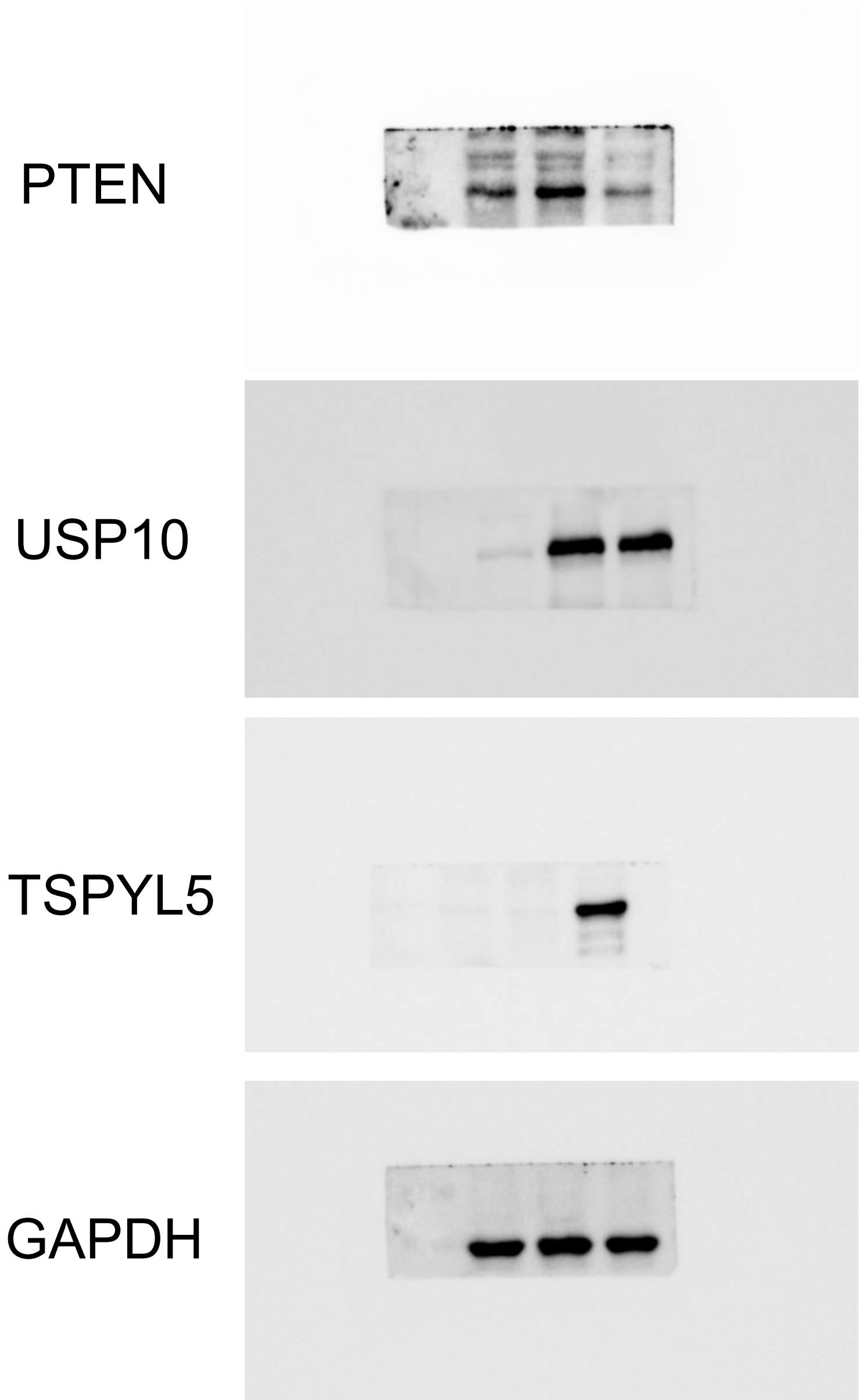

Figure 8K

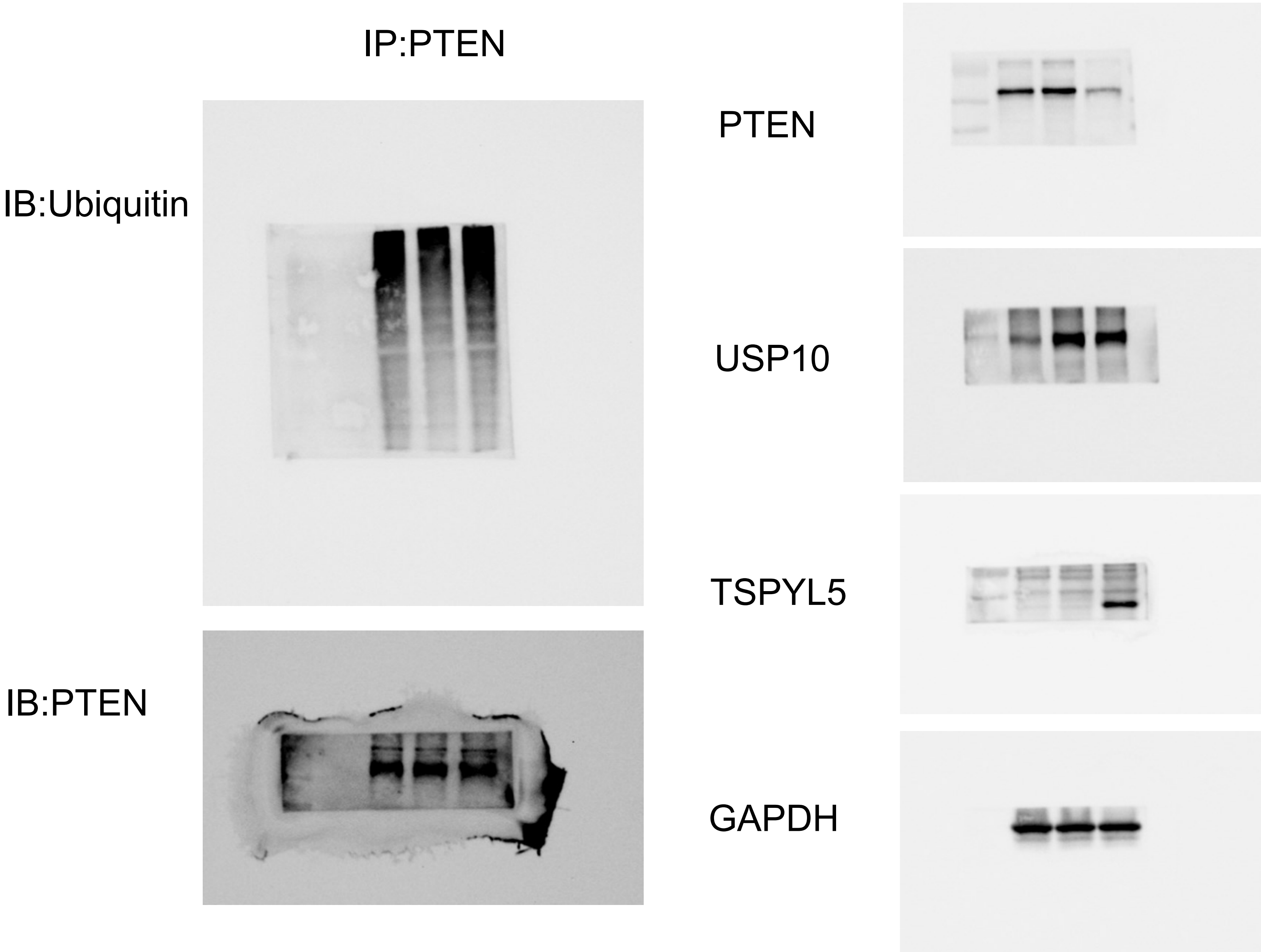

Figure 8L

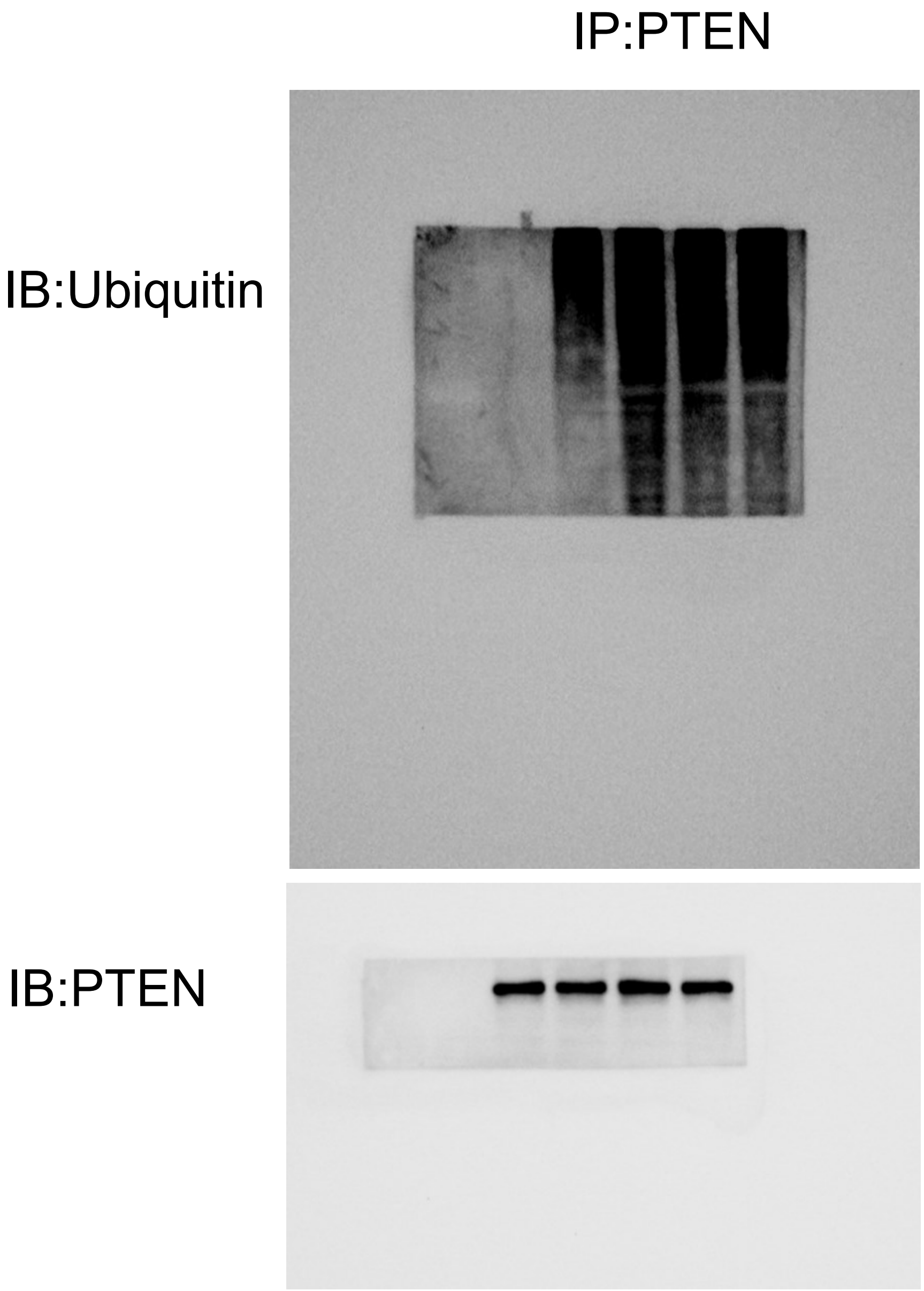

Figure 8L Input

PTEN

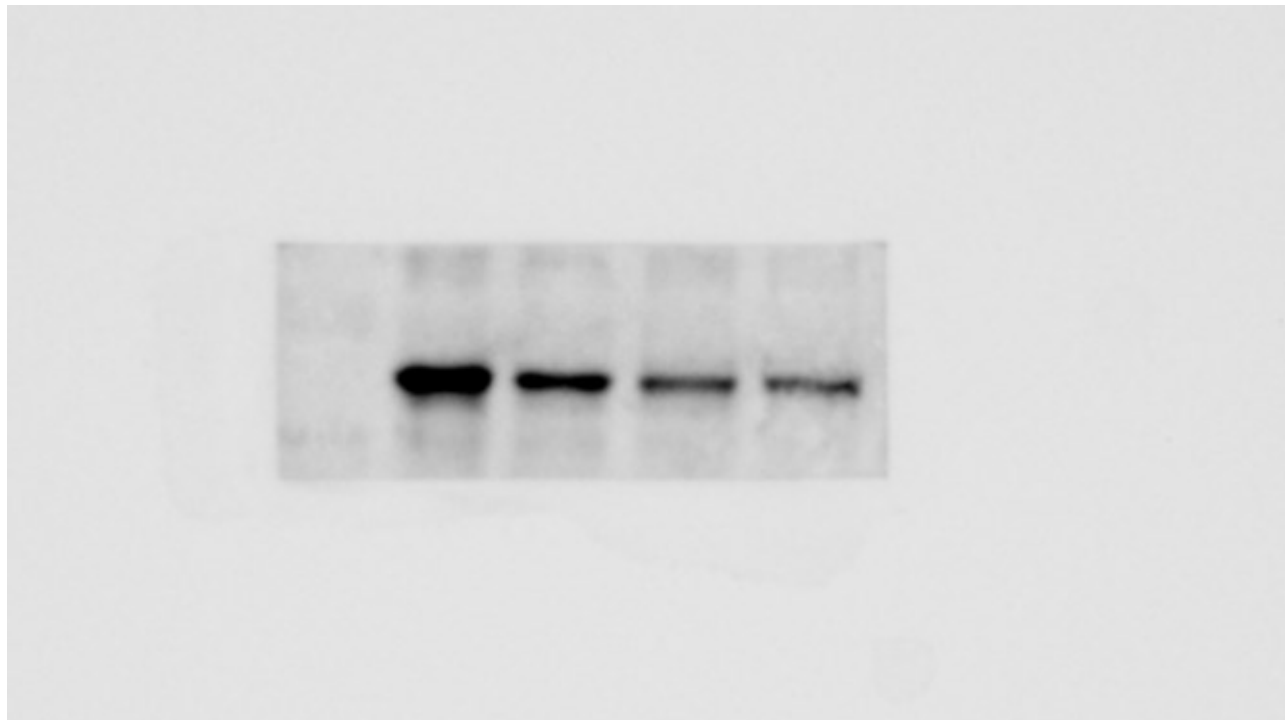

ZEB1

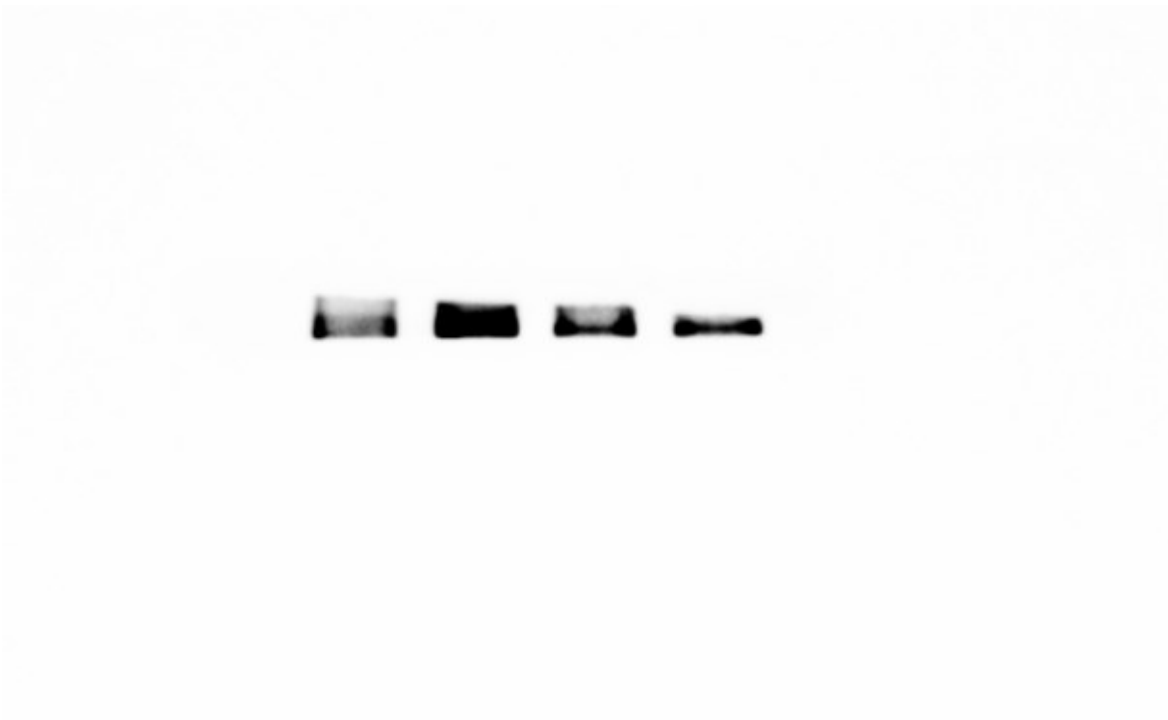

USP10

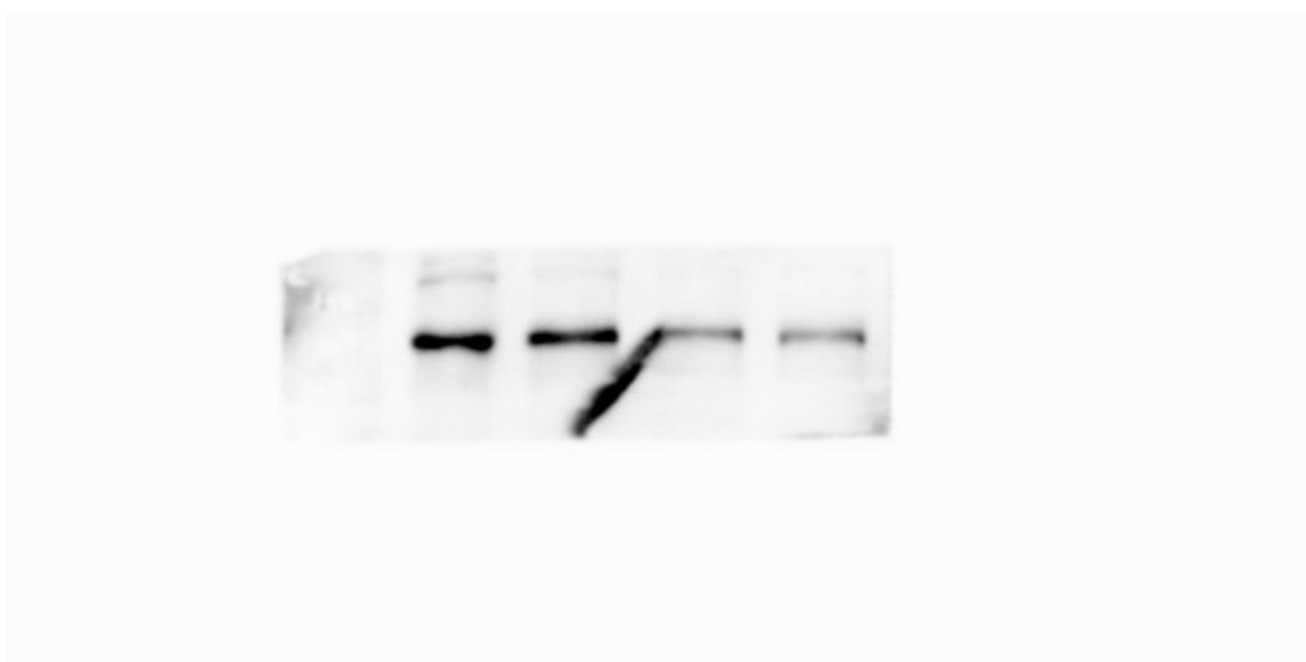

TSPYL5

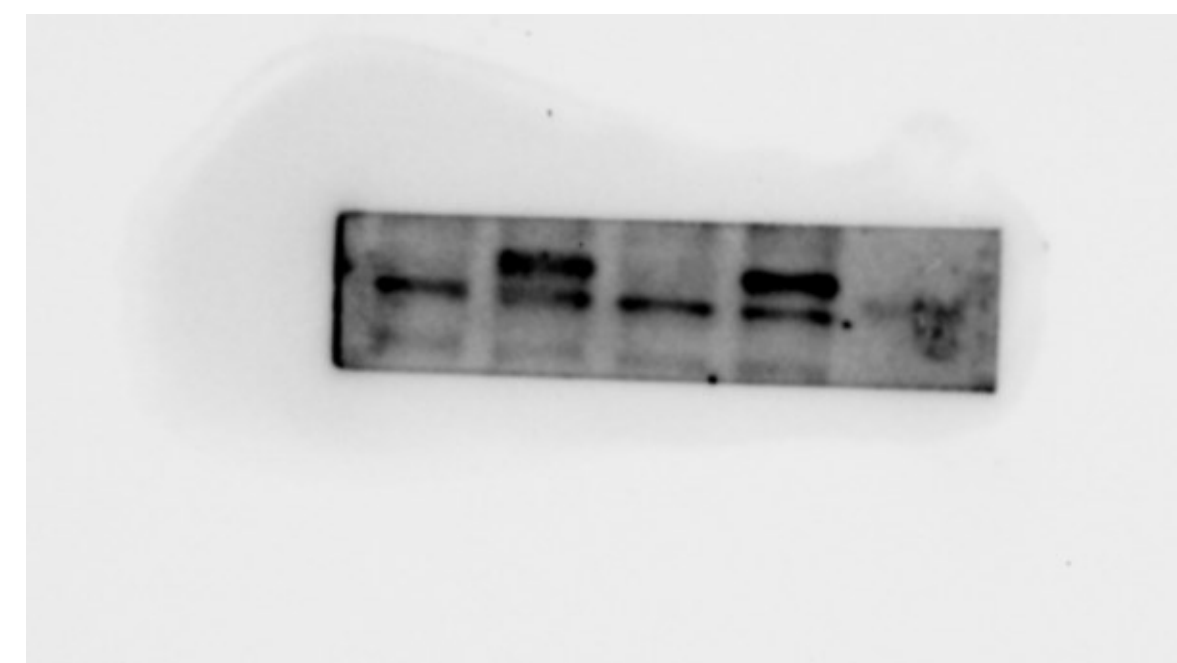

pAKT

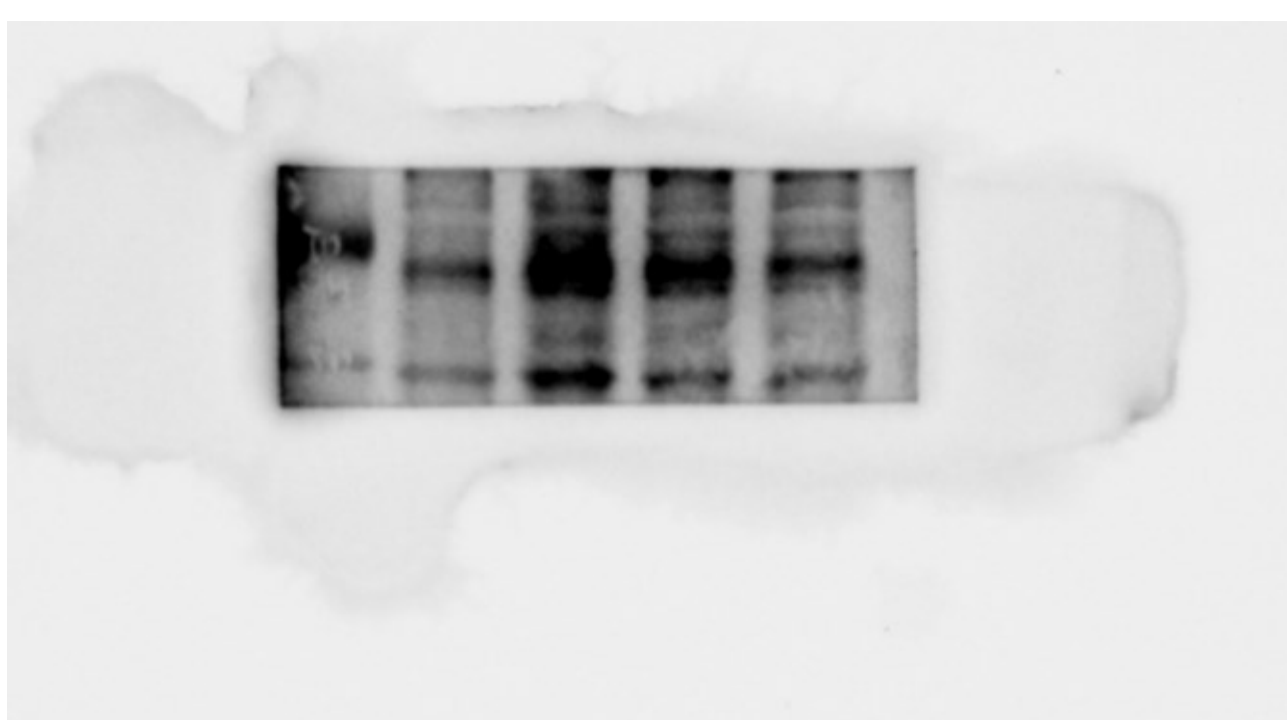

GAPDH

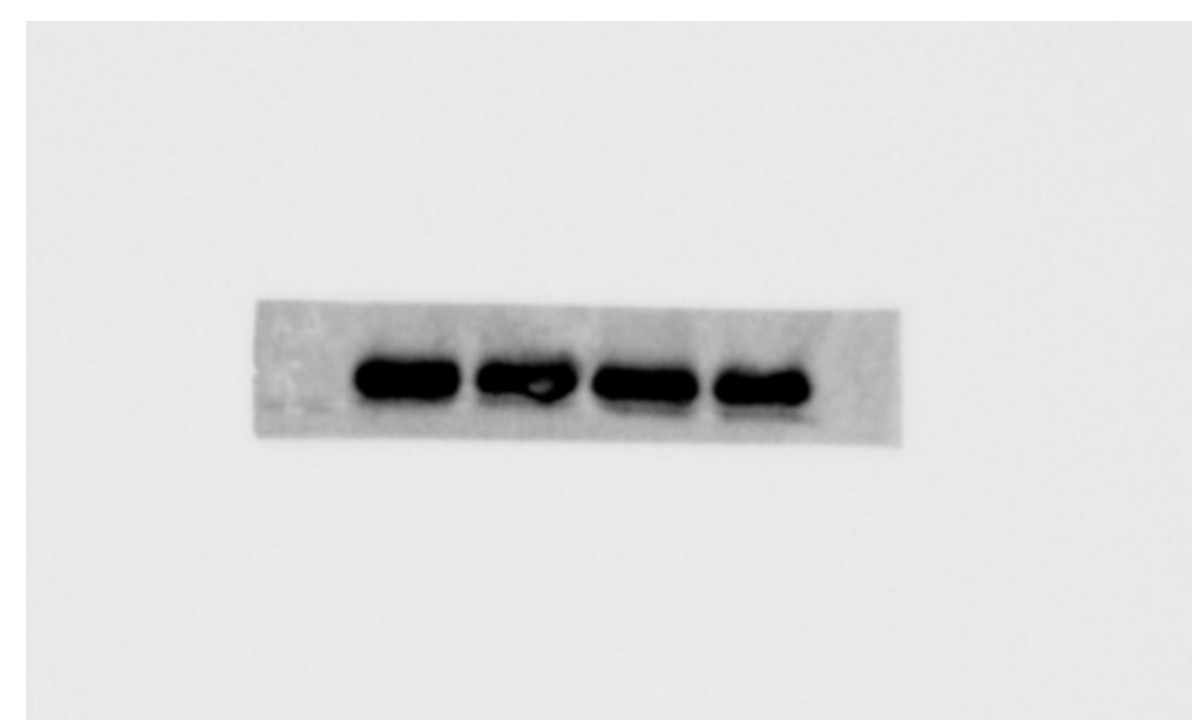

Figure 8N

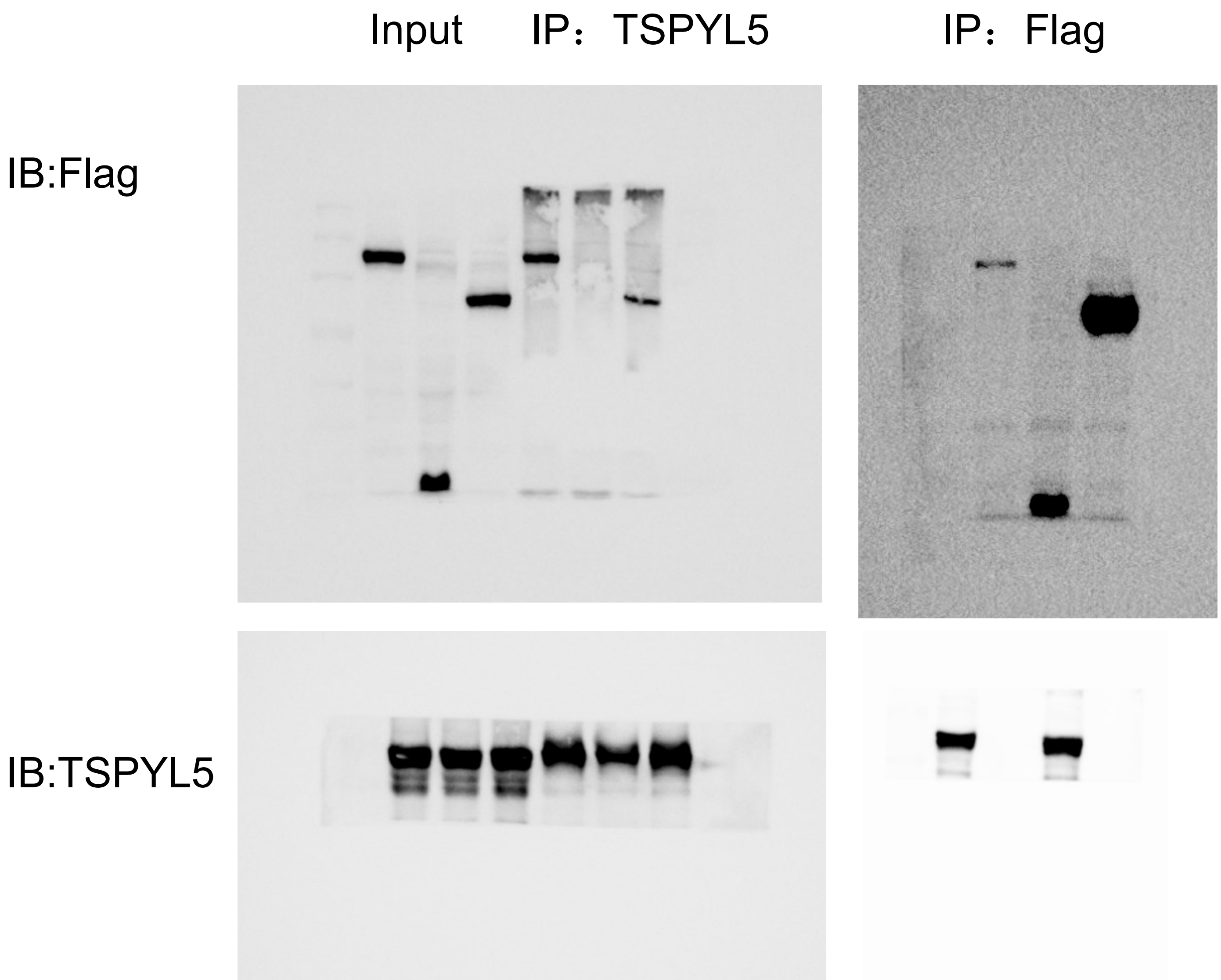

Figure 8O

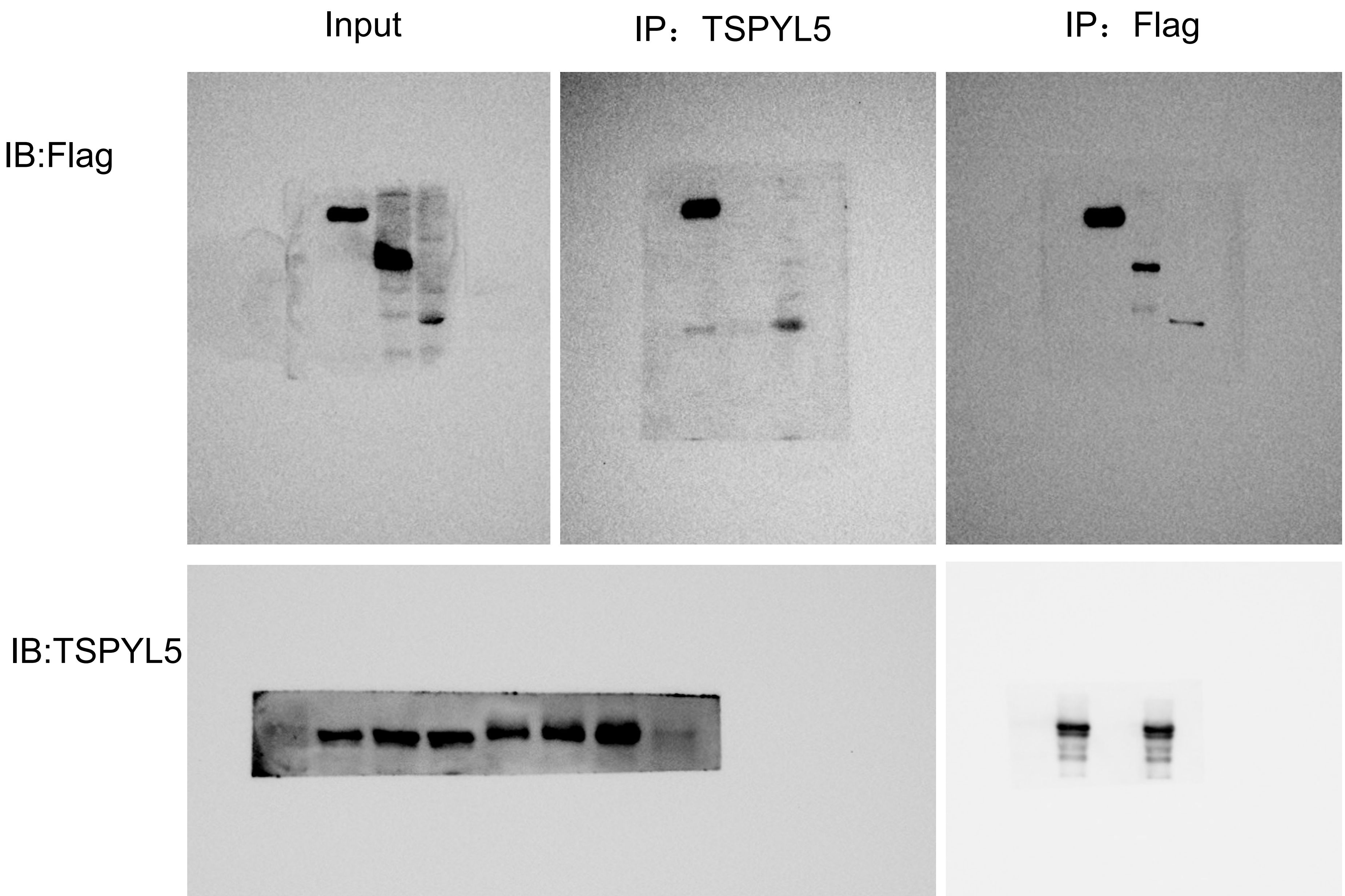

Figure 8P

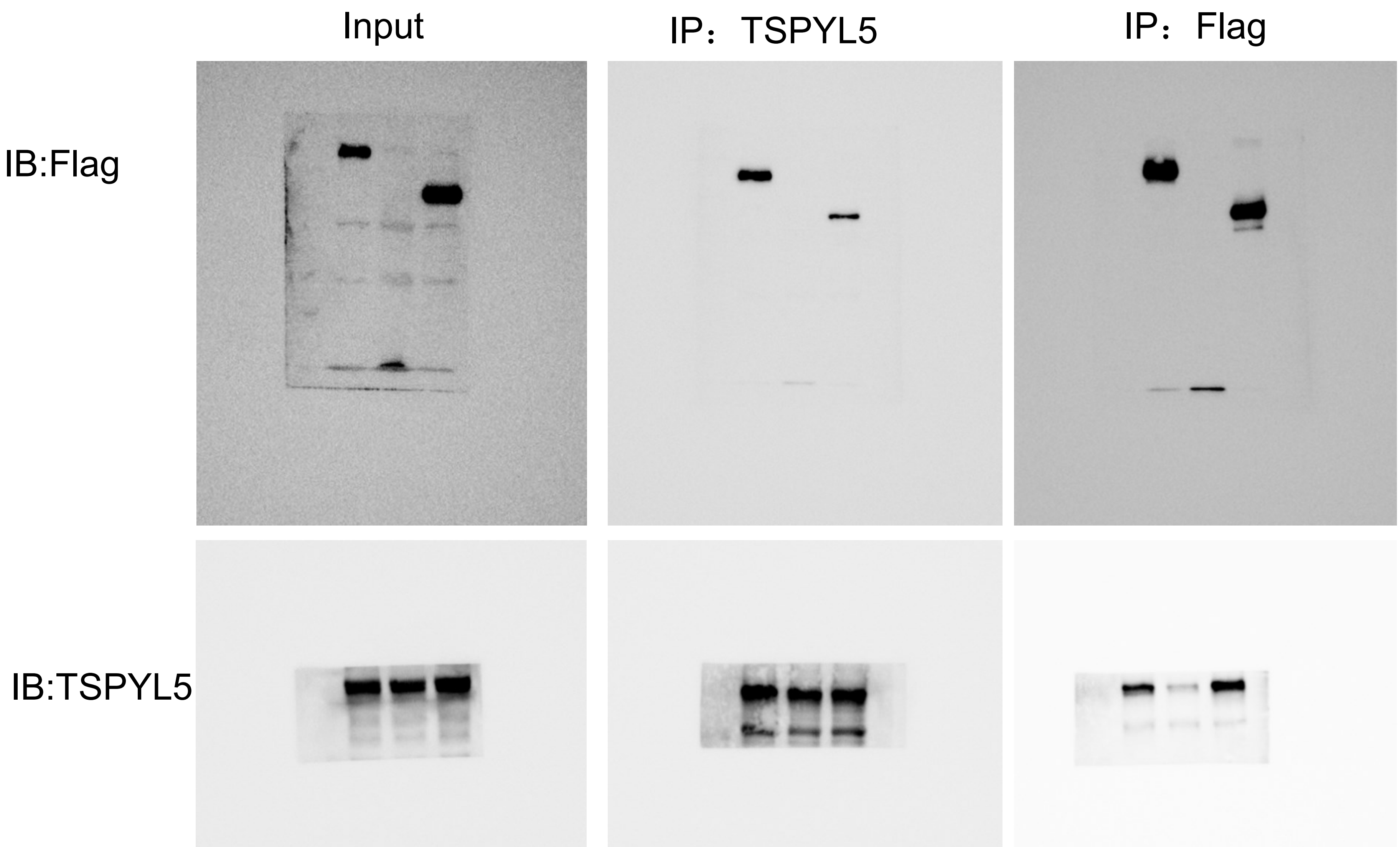

Figure 8Q

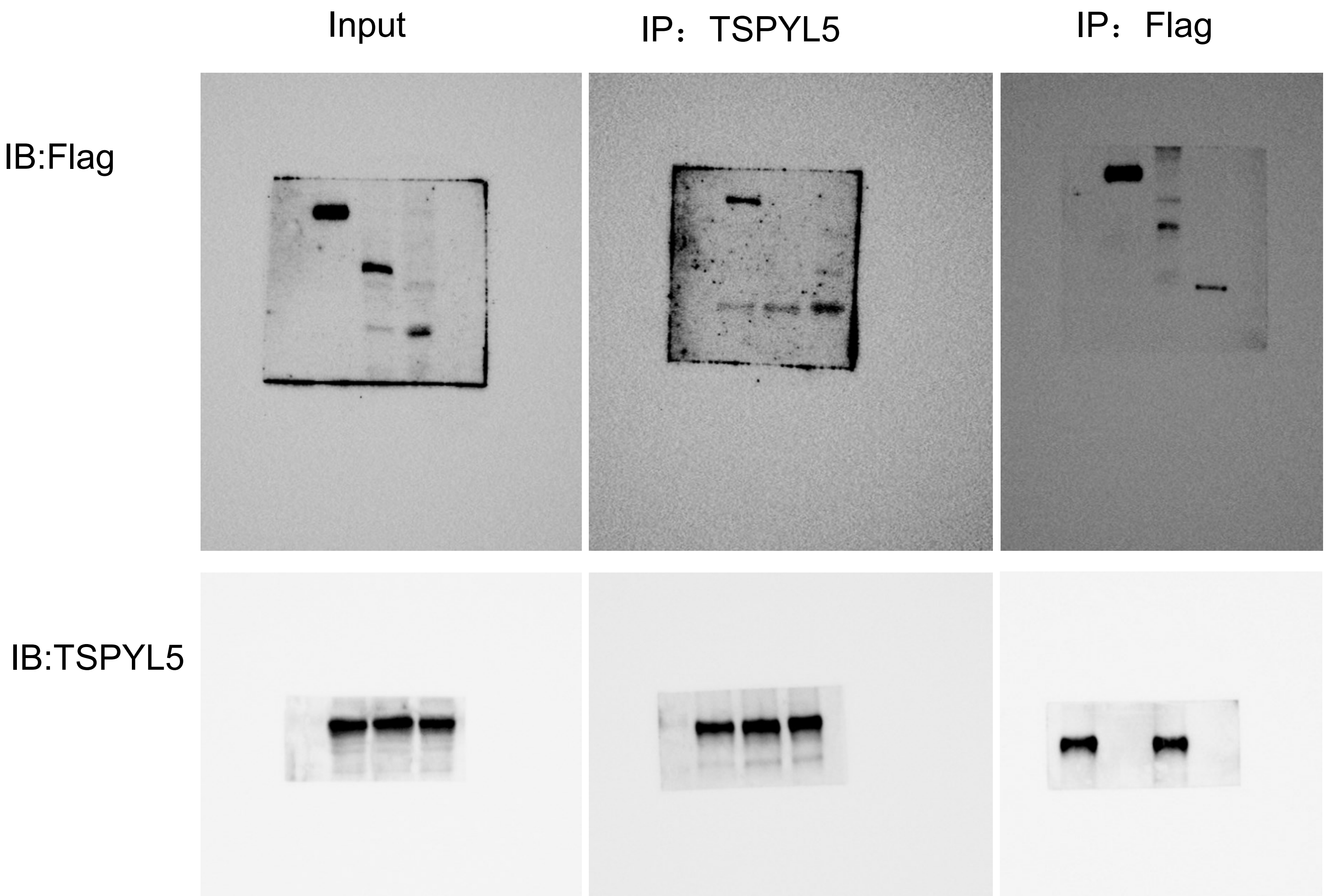

Figure S10B

GSK3β

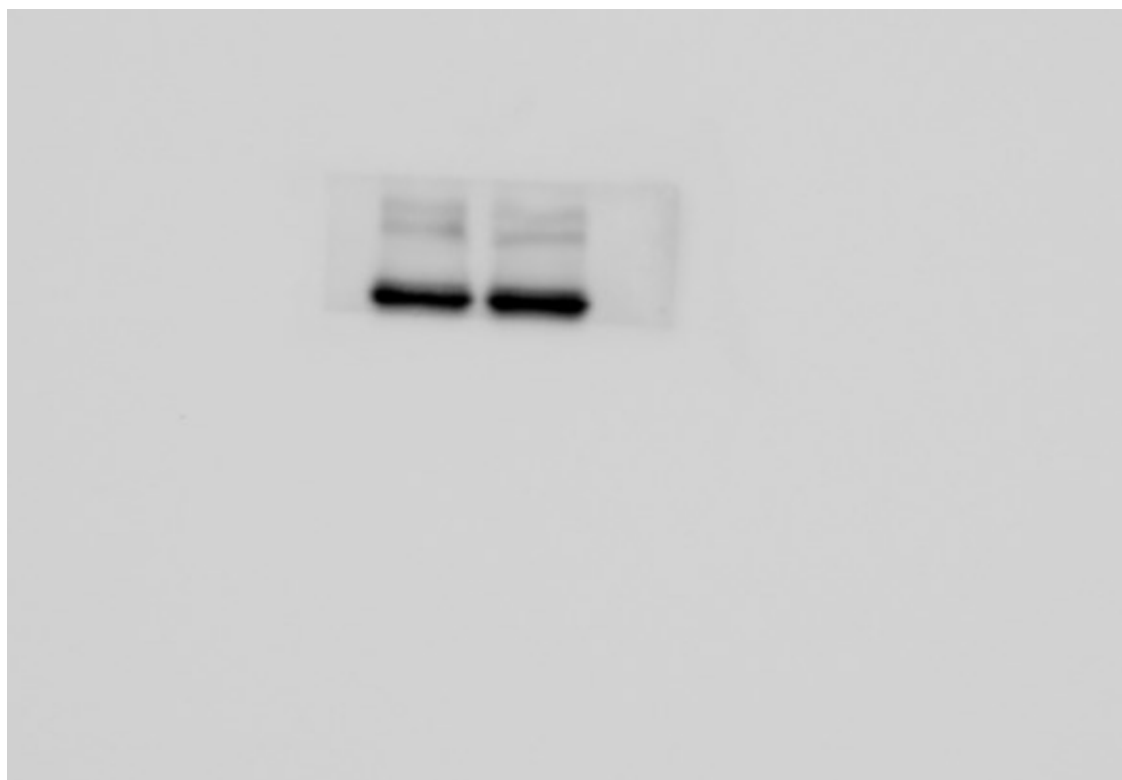

TSPYL5

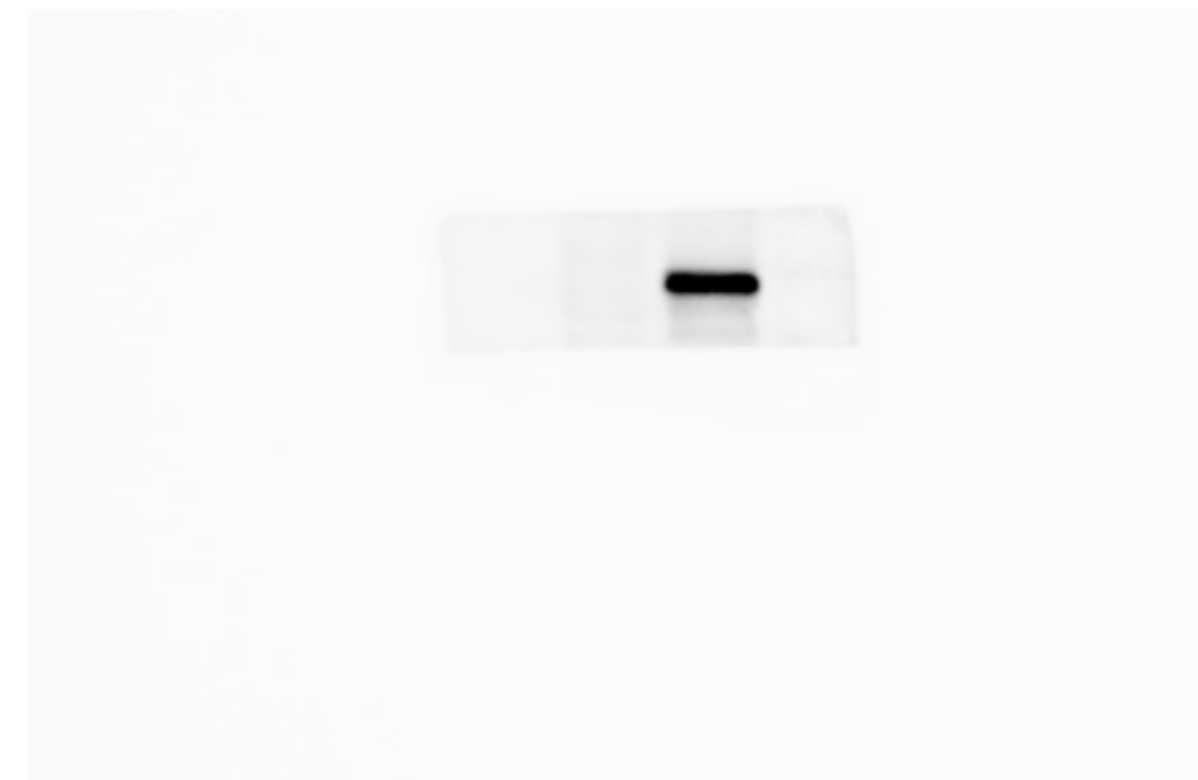

β-catenin

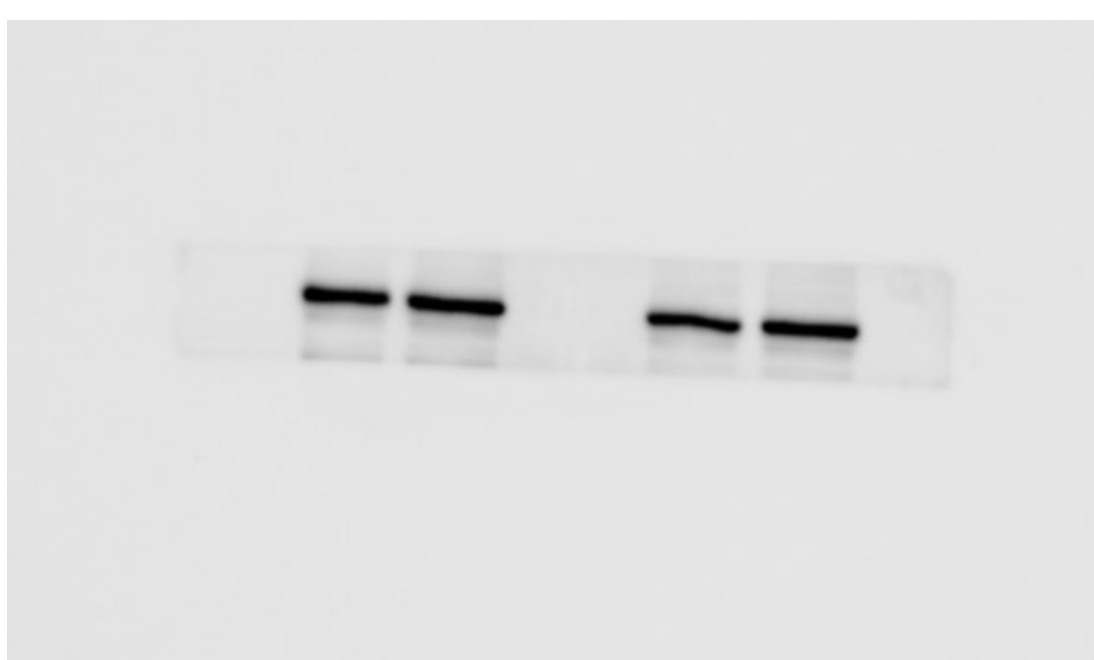

GAPDH

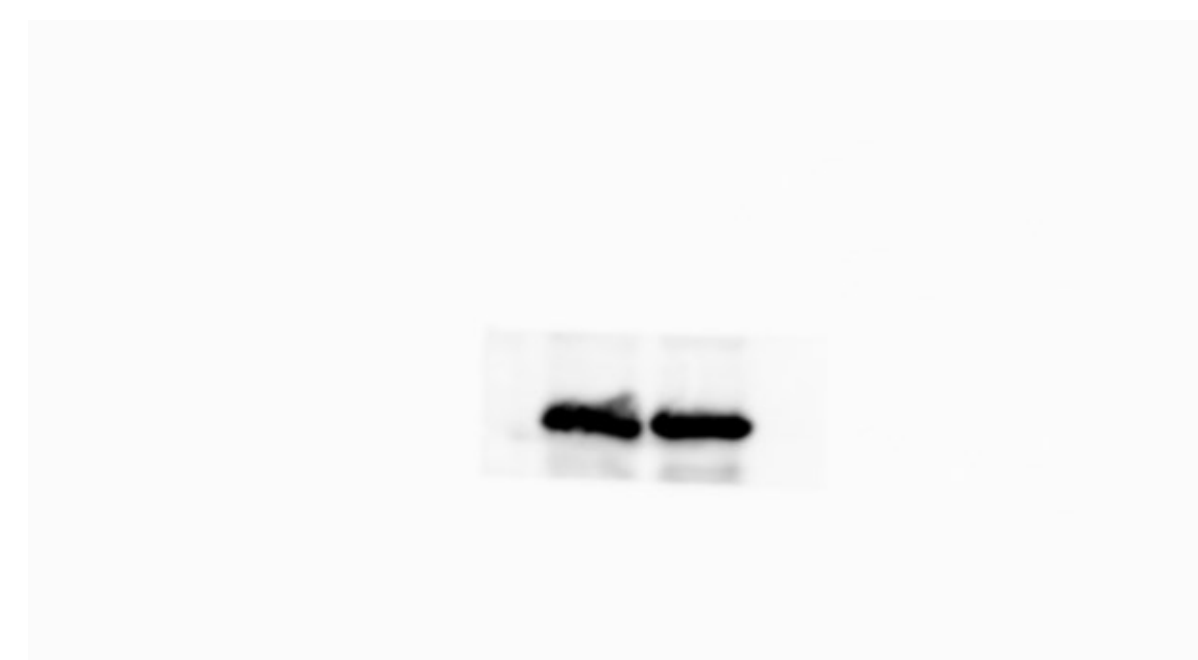

Figure S10B

IL6

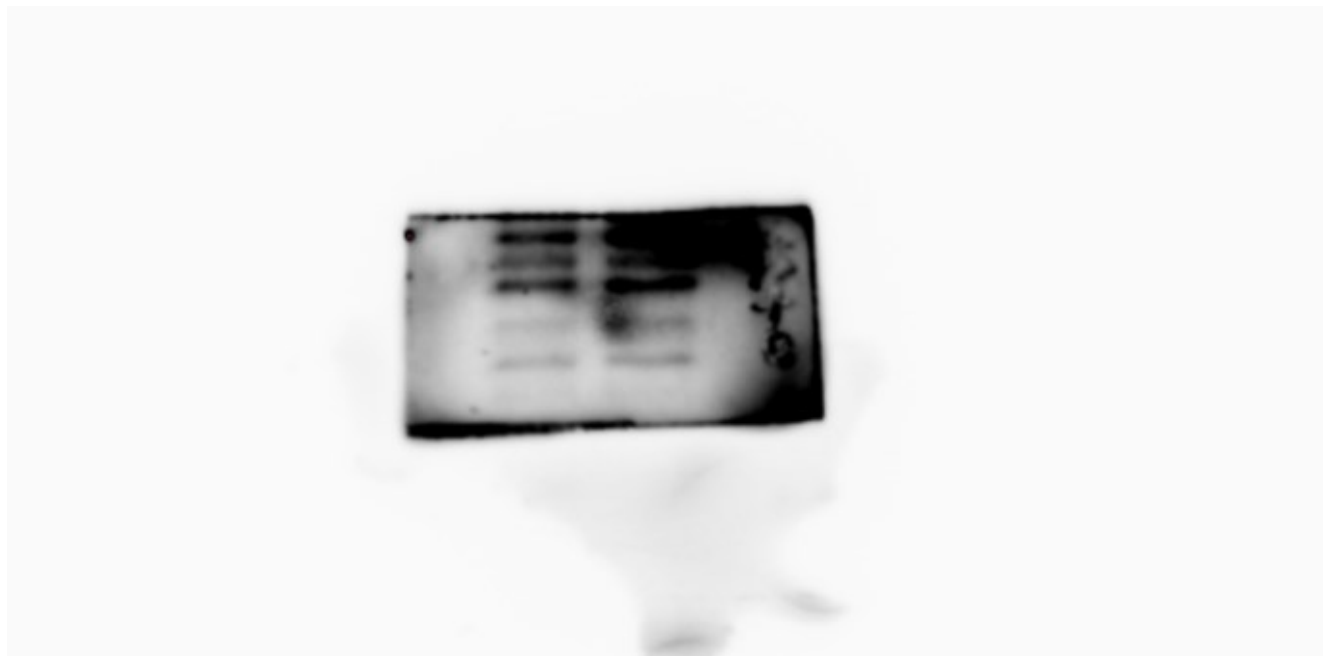

TSPYL5

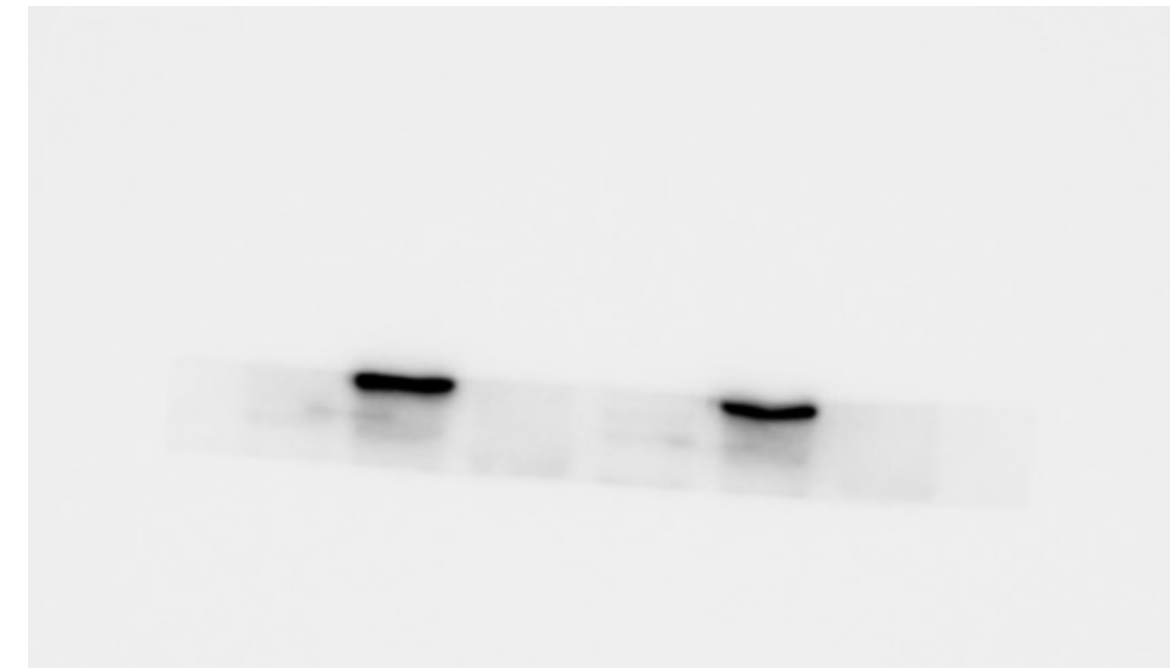

STAT3

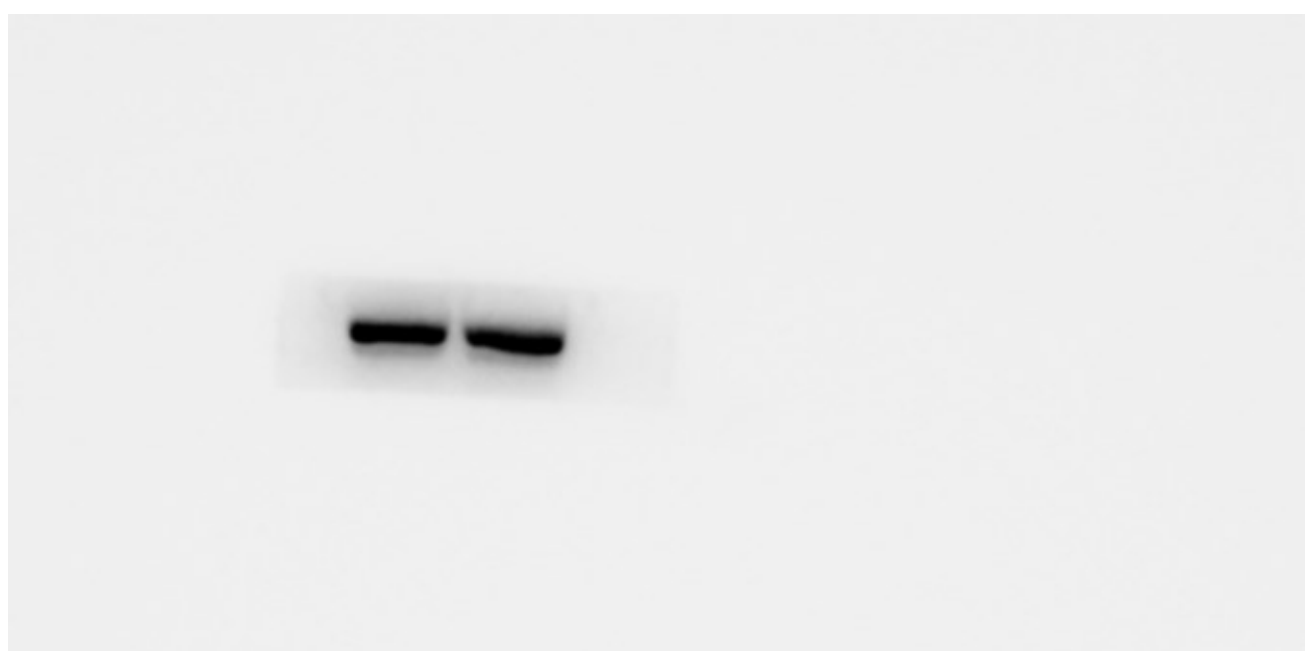

GAPDH

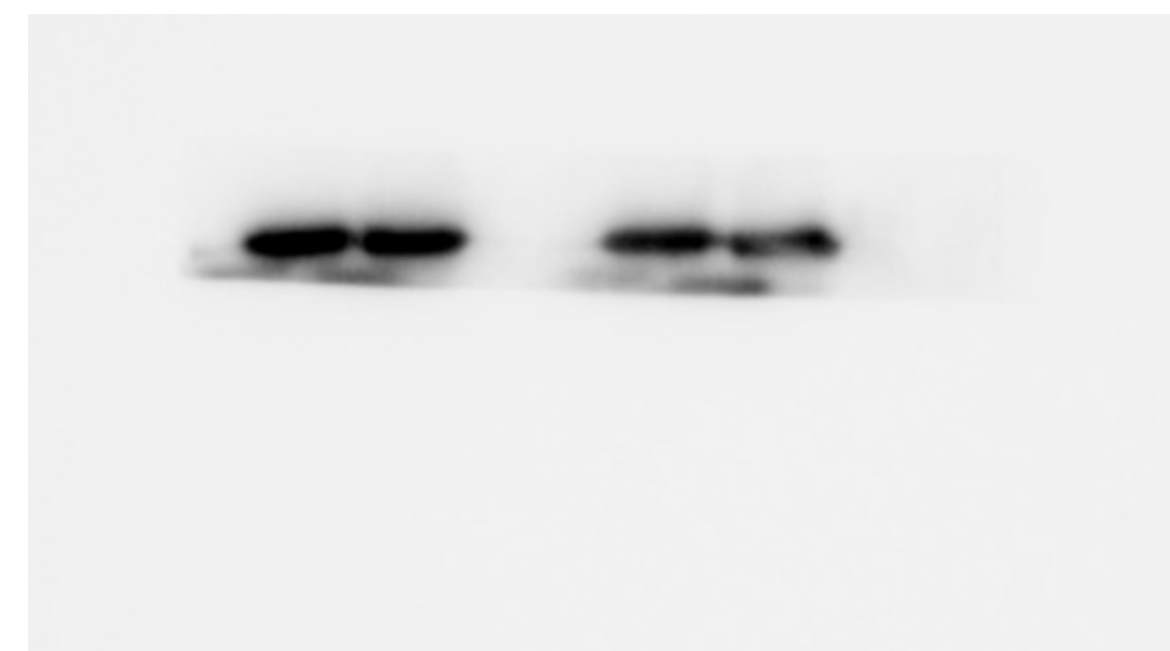

pSTAT3

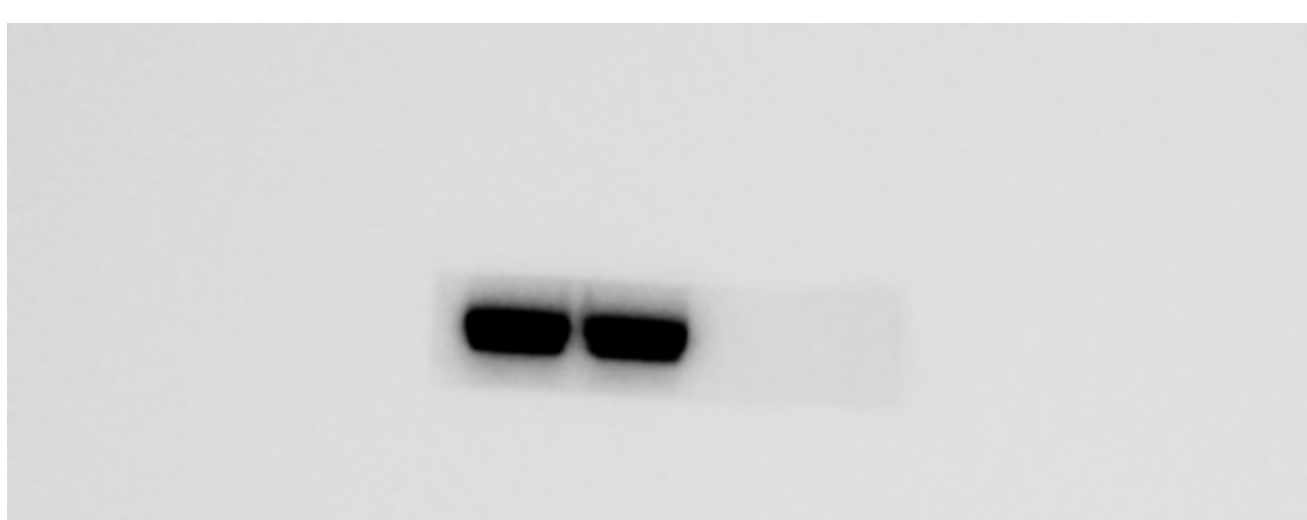

Figure S10B

SMAD2

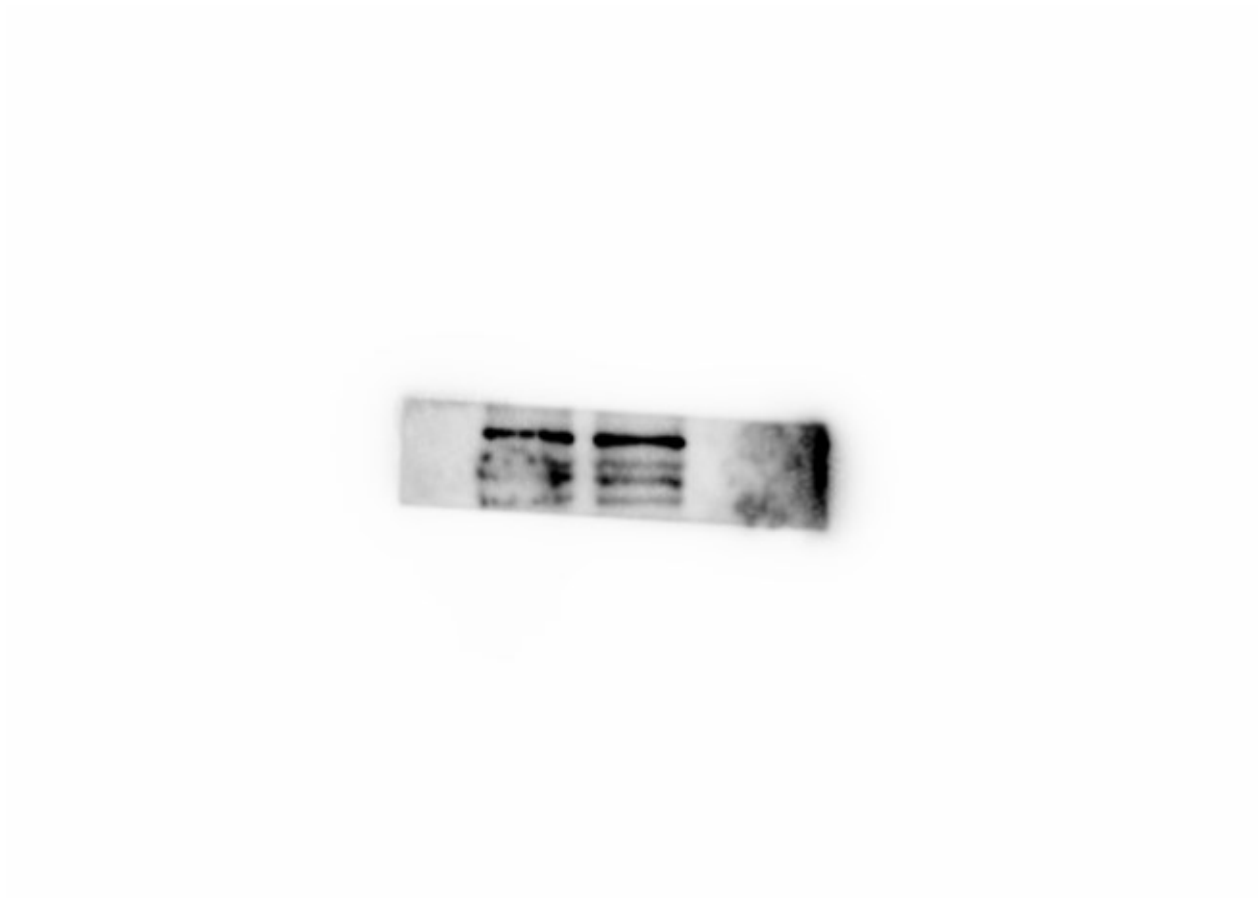

TSPYL5

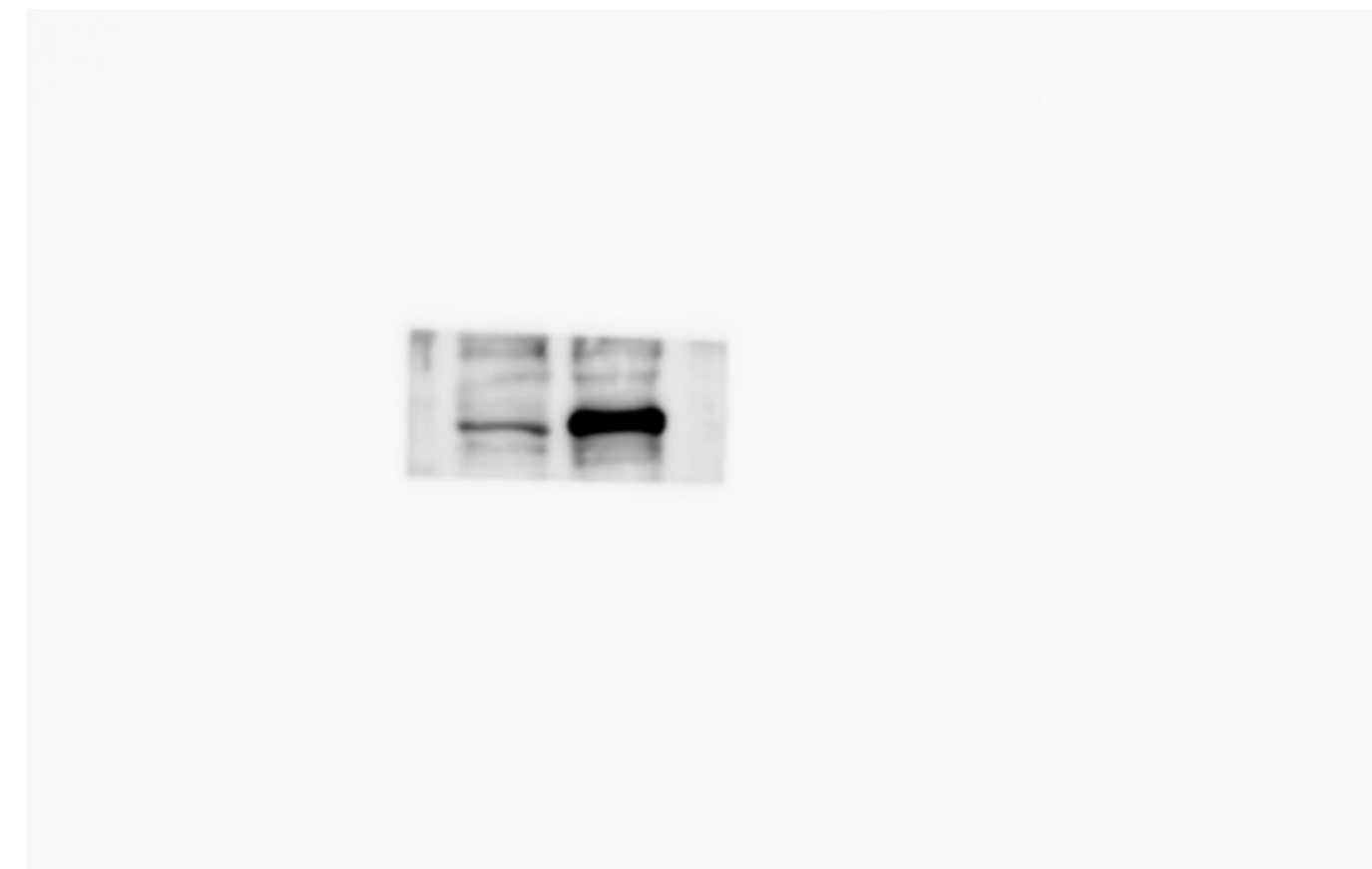

SMAD4

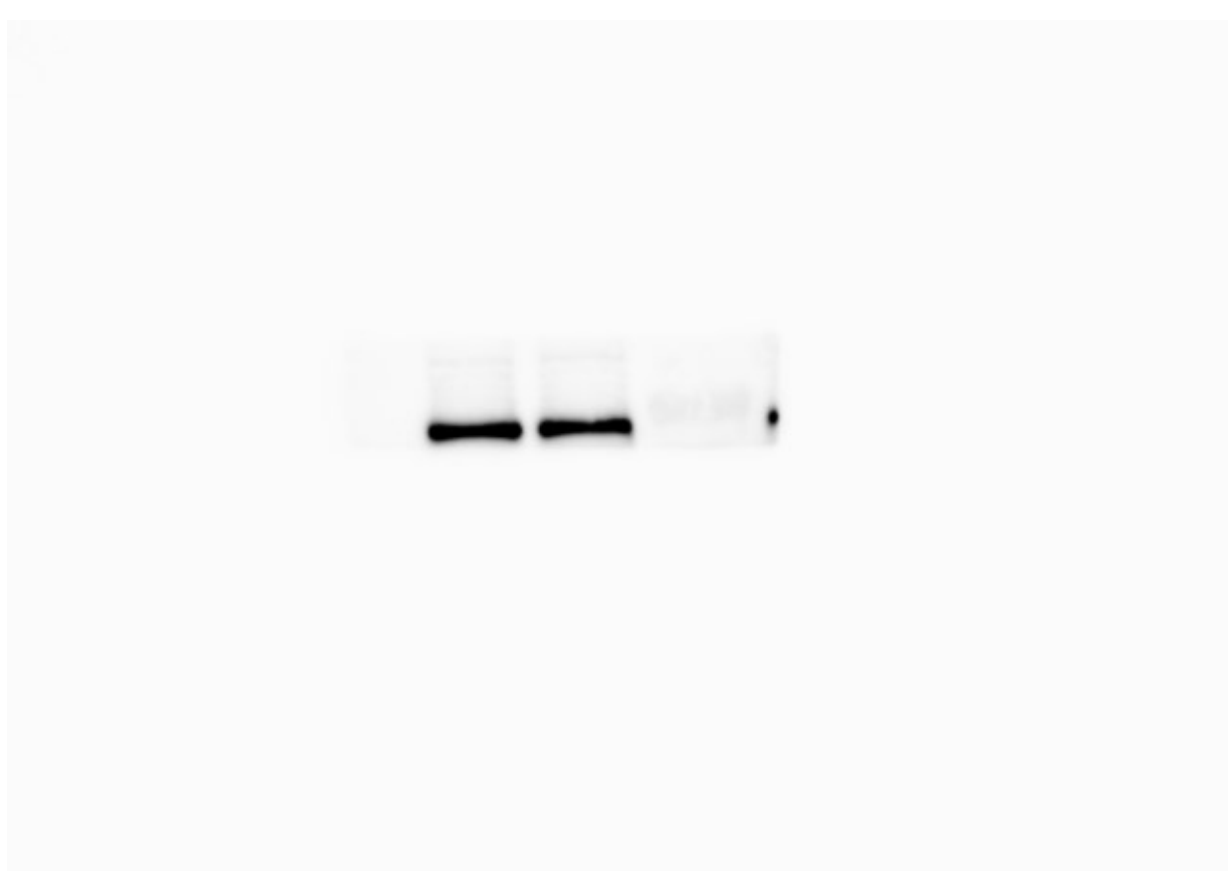

GAPDH

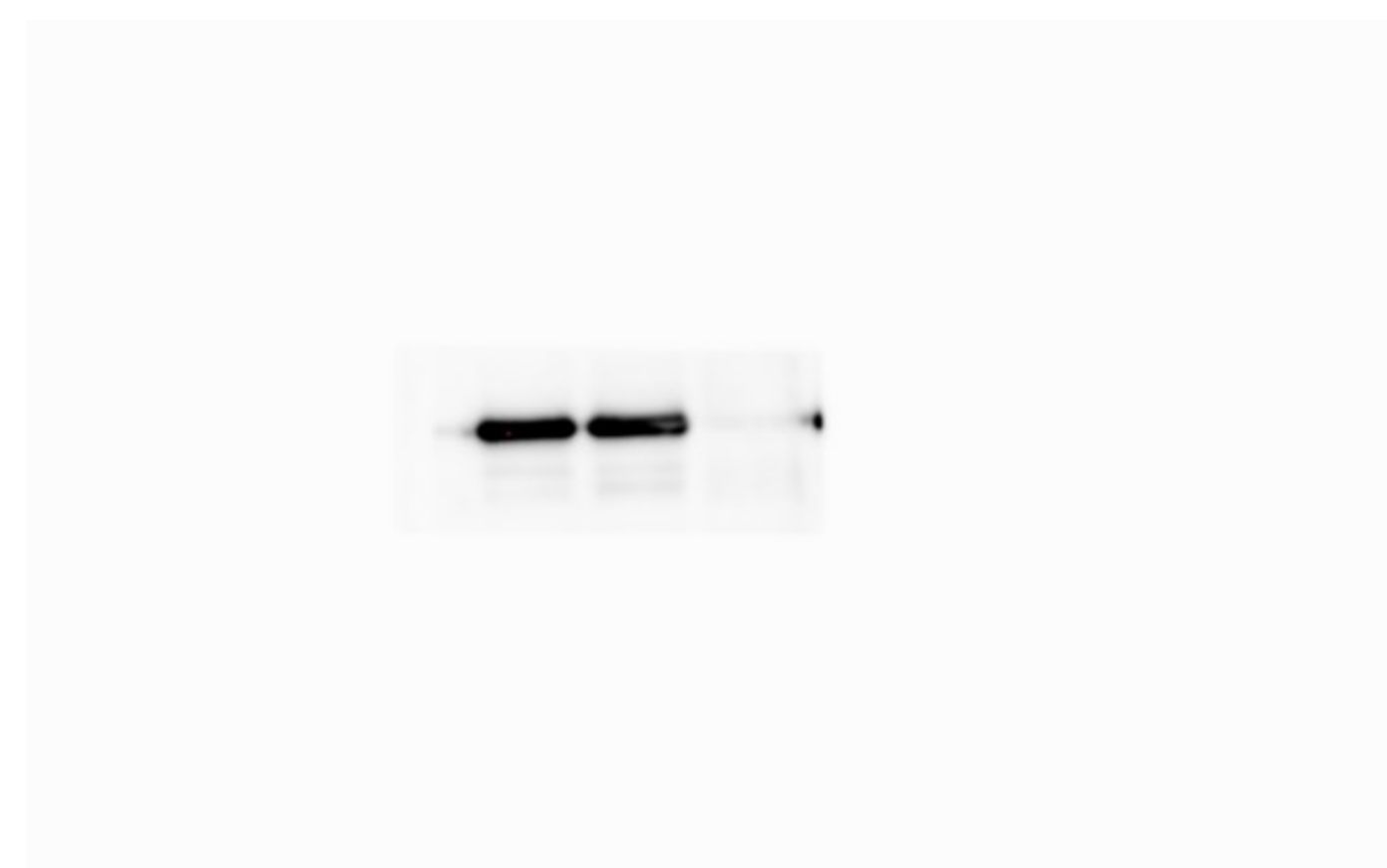

Figure S10B

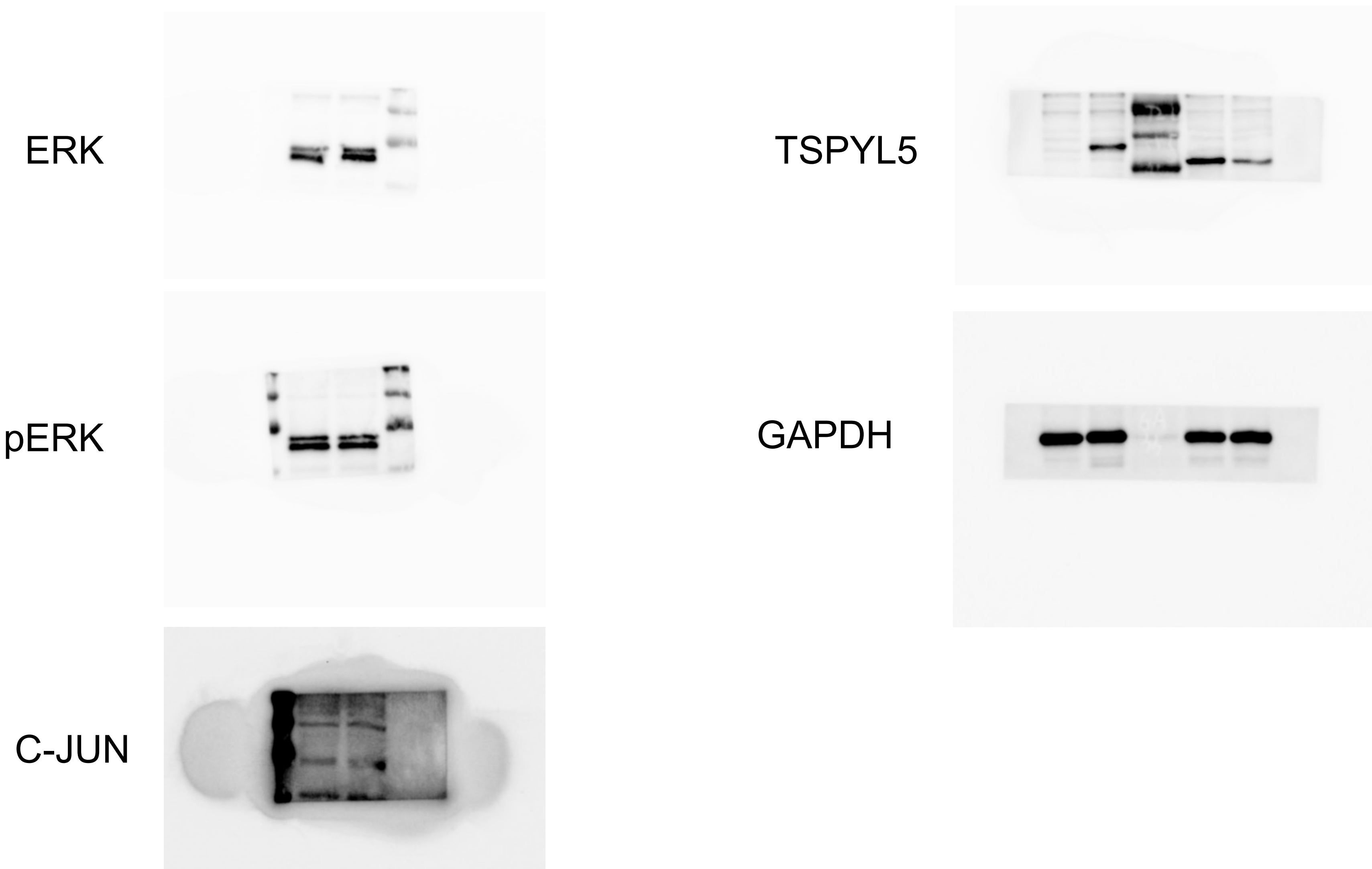

Figure S13A

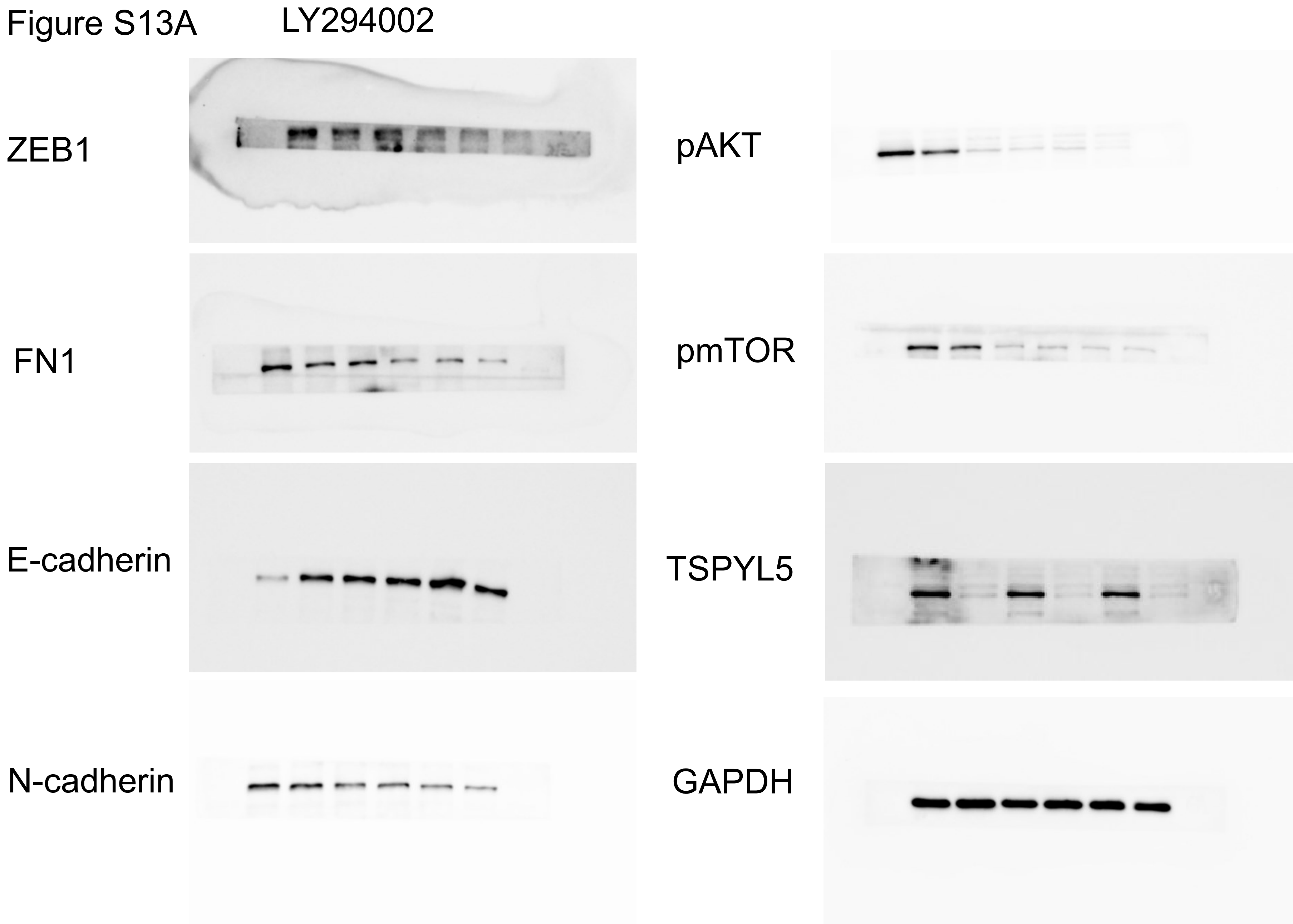
LY294002
ZEB1
pAKT
FN1
pmTOR
E-cadherin
TSPYL5
N-cadherin
GAPDH

Figure S13B

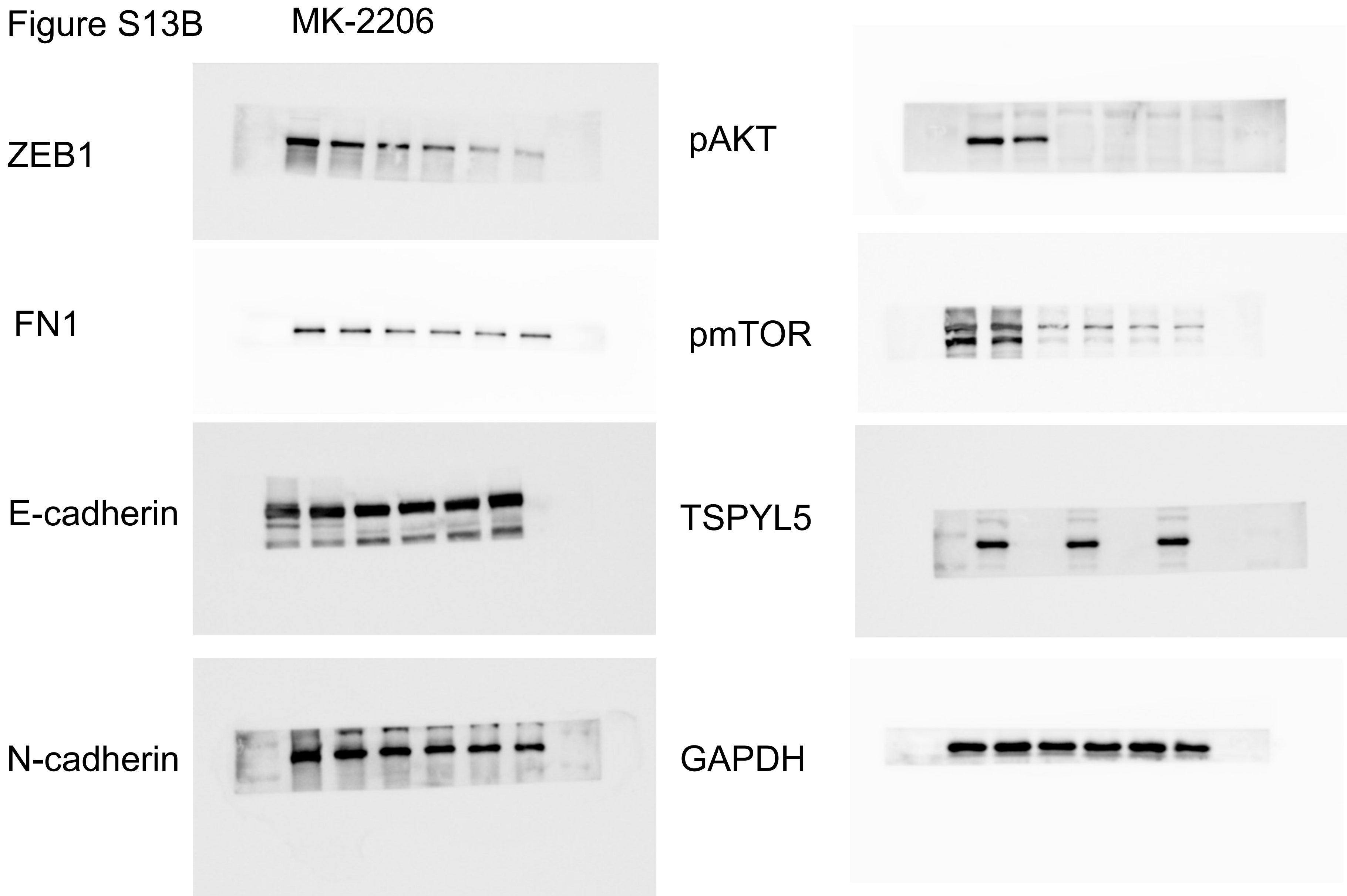
MK-2206
ZEB1
FN1
E-cadherin
N-cadherin
pAKT
pmTOR
TSPYL5
GAPDH

Figure S13C Rapamycin

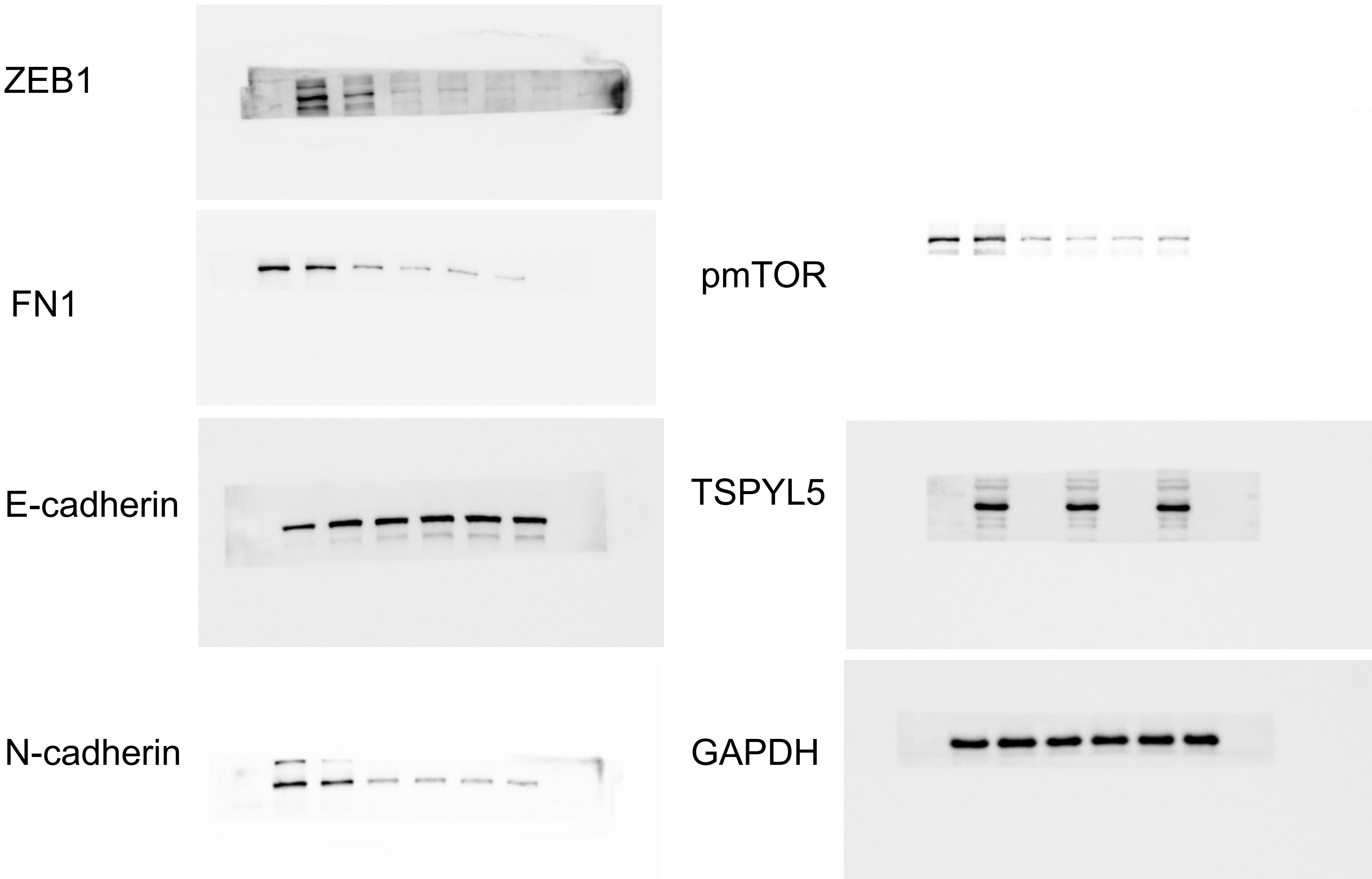

Figure S15B

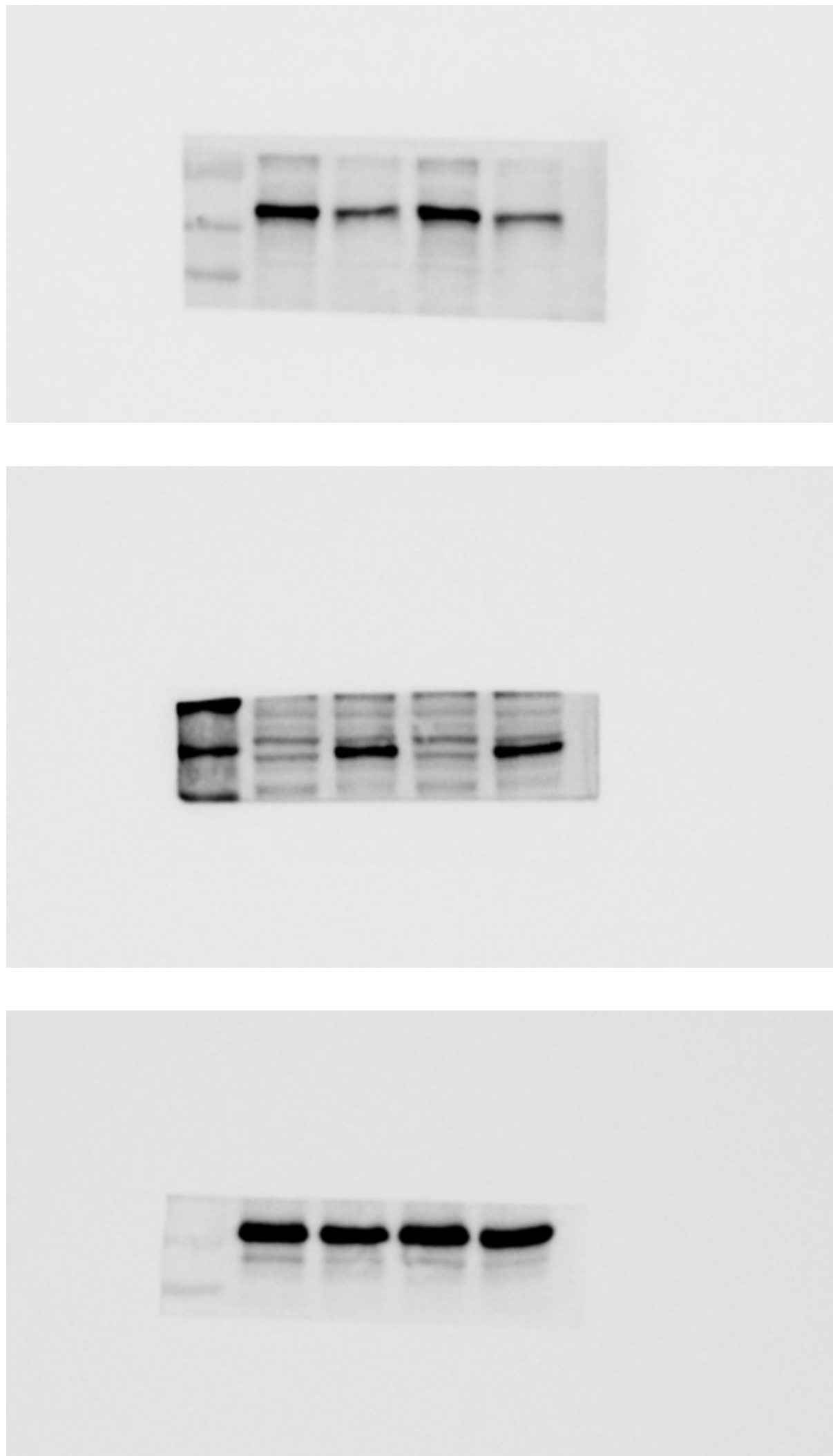

Figure S15D

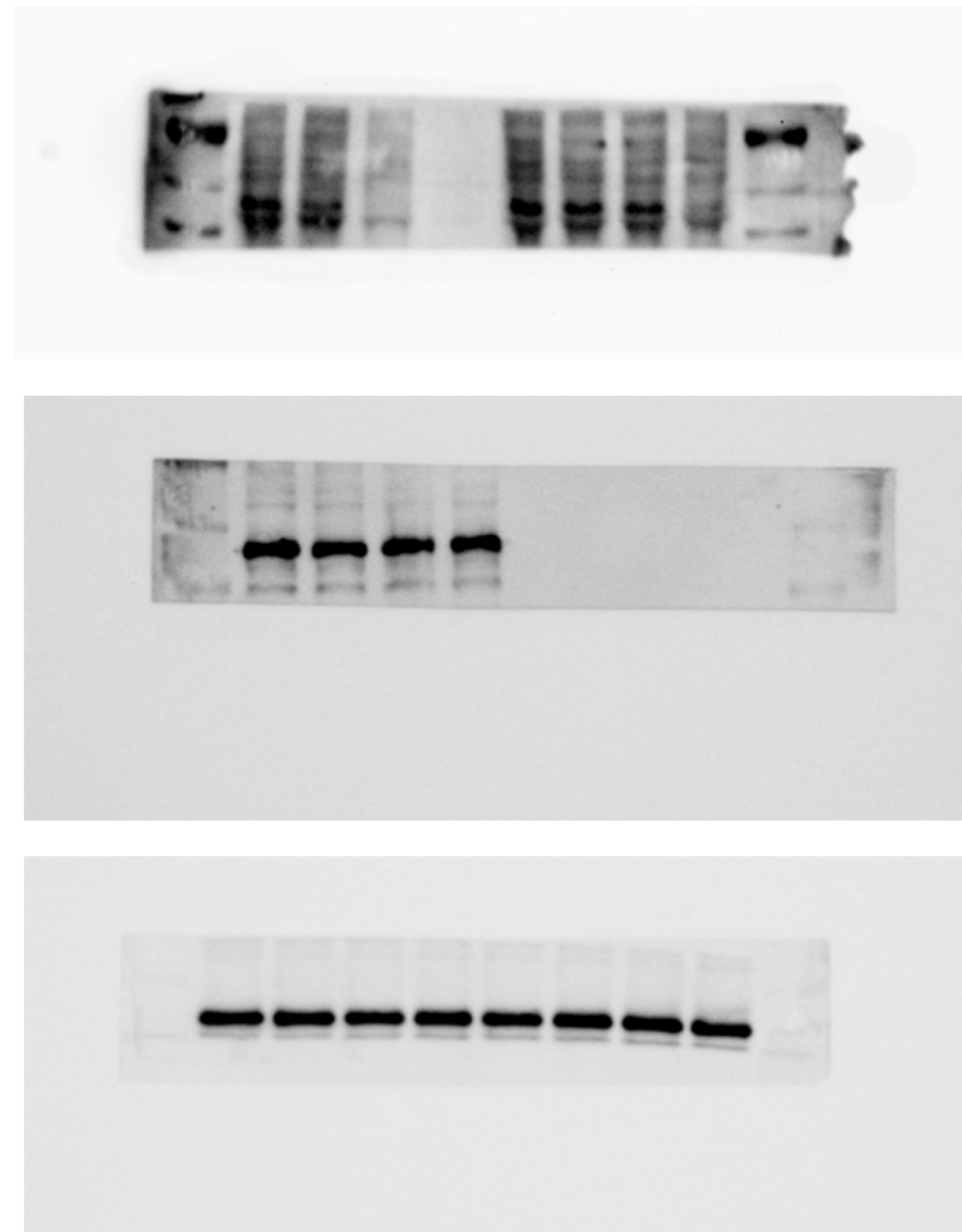

Figure S15E   MDA-MB-231

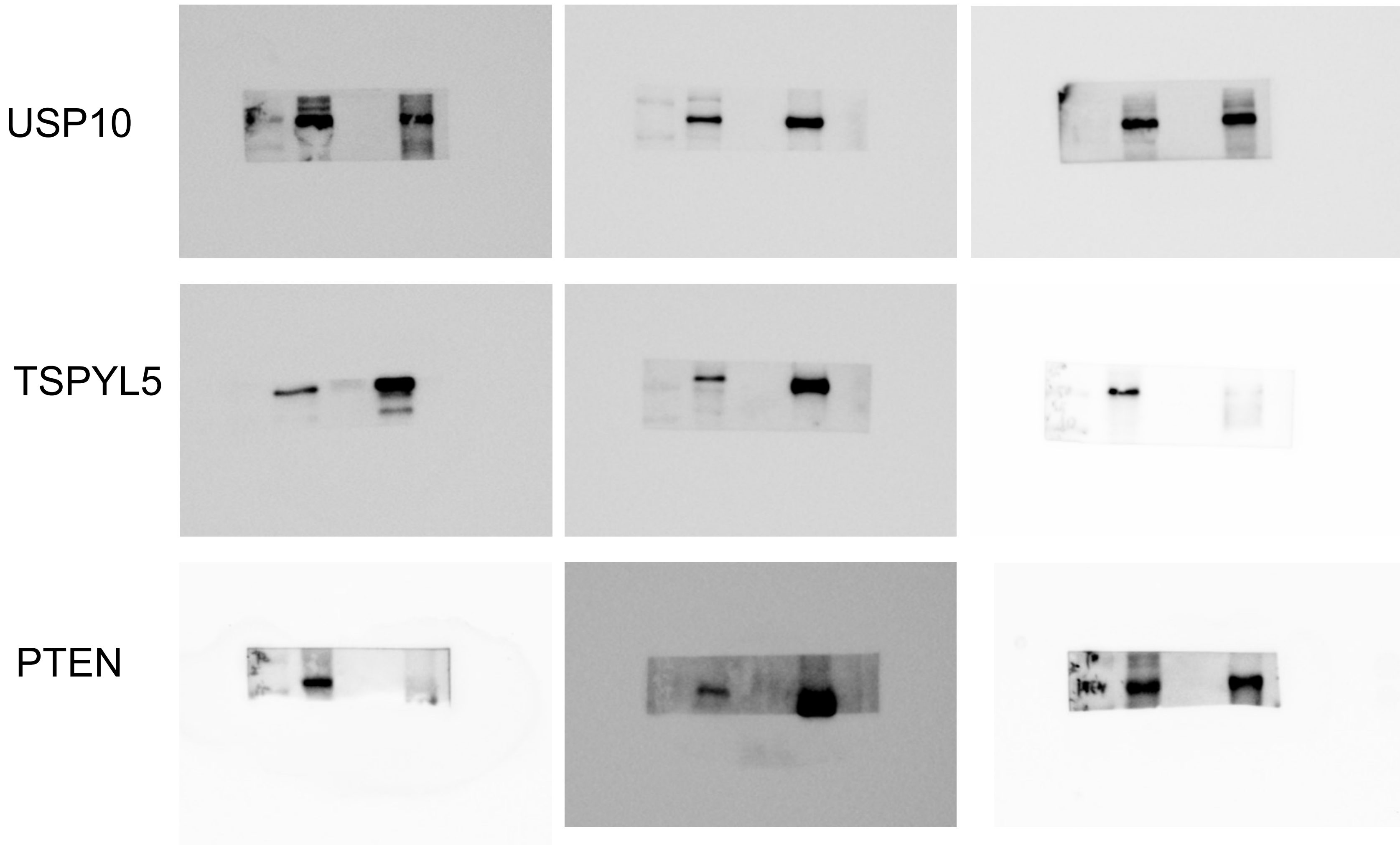

Figure S15F HCC38

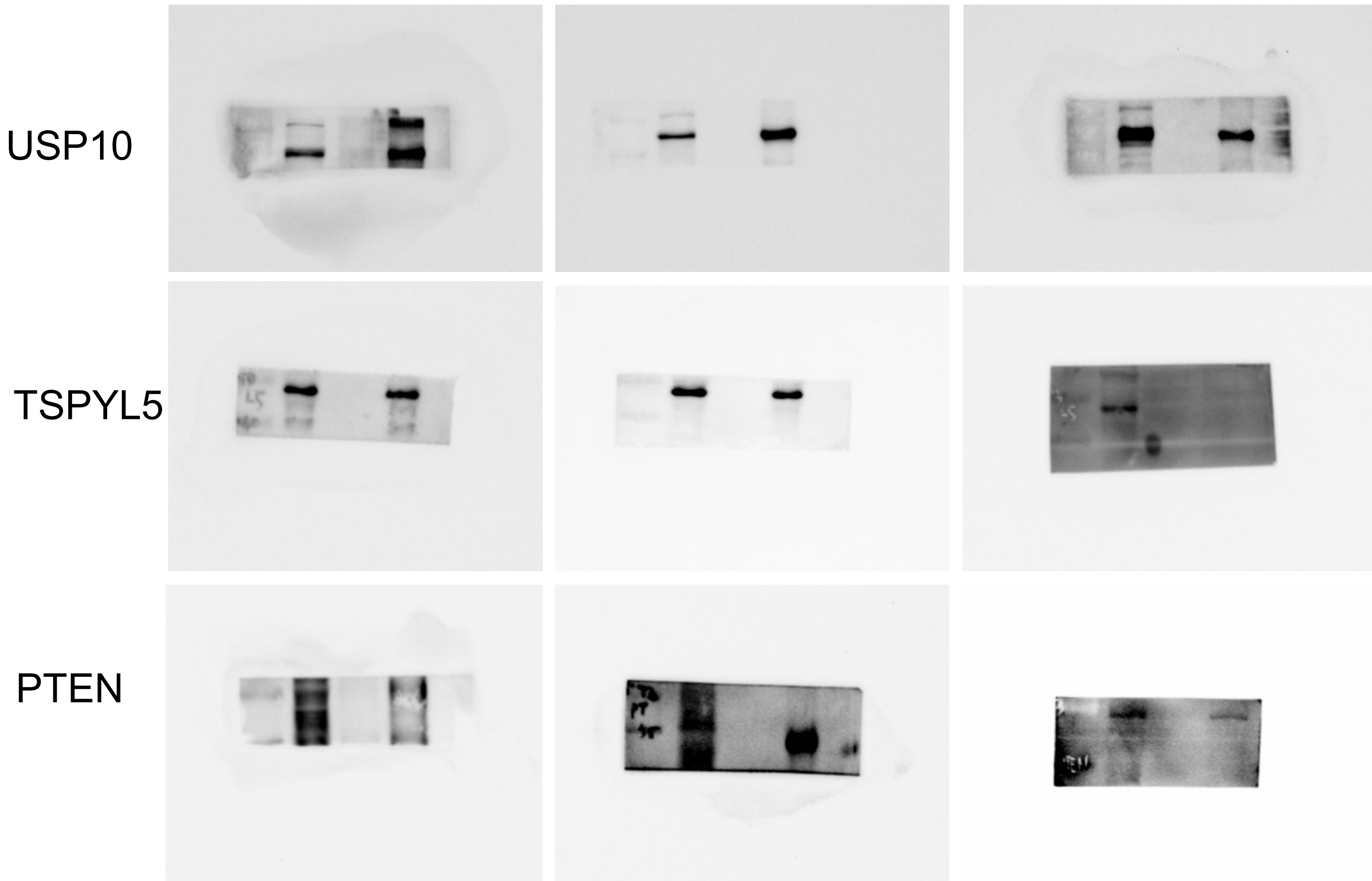

Supplement: Supplementary file 1 [file ADVS-13-e20273-s001.pdf]
