# Supplementary material for: TSPYL5 Promotes Triple‐Negative Breast Cancer Metastasis by Antagonizing USP10‐Mediated PTEN Stabilization to Unleash a ZEB1‐Dependent EMT Program
Source: Adv Sci (Weinh). 2026 Jun 4:e20273. Online ahead of print. doi: 10.1002/advs.202520273 (PMC13336384; doi:10.1002/advs.202520273)
Supplement: Supplementary file 1 — Supporting File 1: advs75960‐sup‐0001‐SuppMat.docx. [file ADVS-9999-e20273-s002.docx]

Supporting Information

TSPYL5 Promotes Triple-Negative Breast Cancer Metastasis by Antagonizing USP10-mediated PTEN Stabilization to Unleash a ZEB1-Dependent EMT Program

Jiaying Shi ^1,2†^, Ming Yi ^1,3†^, Shengyu Xie ^1†^, Zhaokun Wang ^1^, Xinyue Zhang ^1^, Yangwei Zhang ^1^, Rui Tang ^1^, Yuan Yang ^1*^, Yunqiang Liu ^1*^

^1^ Department of Medical Genetics, State Key Laboratory of Biotherapy, West China Hospital, Sichuan University, Chengdu, China

^2^ Department of Rehabilitation Medicine, Xuanwu Hospital, Capital Medical University, Beijing, China

^3^ Frontiers Science Center for Disease-related Molecular Network, State Key Laboratory of Biotherapy, West China Hospital, Sichuan University, Chengdu, China

^†^The first three authors contributed equally to this work.

E-mail: Yunqiang Liu, yq_liu@scu.edu.cn; Yuan Yang, yangyuan@scu.edu.cn

Funding: This work was supported by grants from the National Natural Science Foundation of China (81871203) and the Sichuan Provincial Natural Science Foundation (No. 2025ZNSFSC0564).


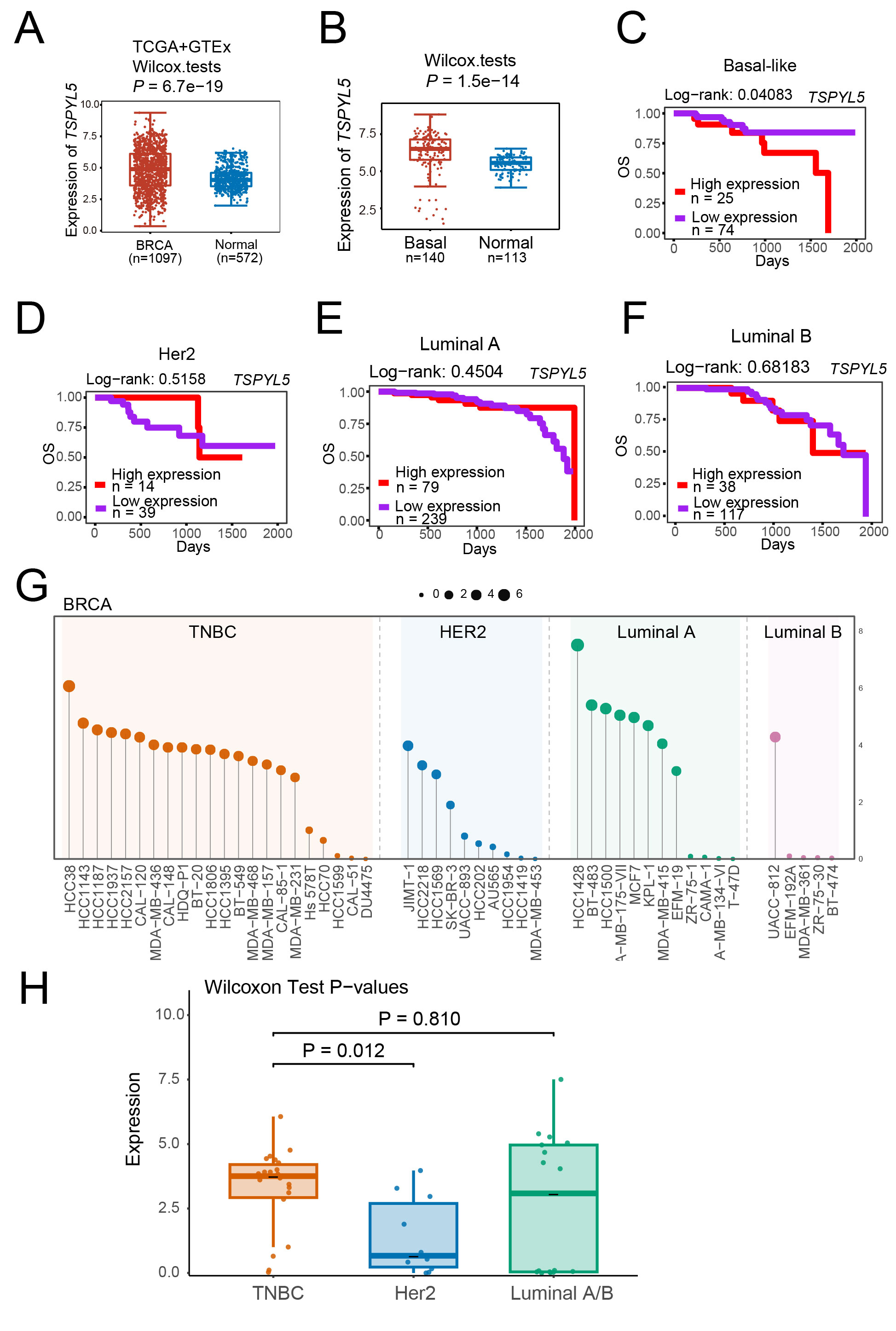


**Figure S1.** Expression patterns and prognostic significance of *TSPYL5* across breast cancer subtypes. (A) Box plot comparing differential mRNA expression of *TSPYL5* between breast cancer (n = 1097) and normal breast tissues (n = 572). Data were sourced from the TCGA and GTEx databases. (B) Comparison of *TSPYL5* mRNA expression specifically between basal-like breast cancer (n=140) and normal breast tissues (n=113) from the TCGA cohort. (C–F) Kaplan-Meier survival analyses illustrating the association between *TSPYL5* expression and overall survival (OS) stratified by *TSPYL5* expression levels across distinct intrinsic molecular subtypes in the TCGA cohort: Basal-like (C), HER2-enriched (D), Luminal A (E), and Luminal B (F). (G, H) Relativele *TSPYL5* mRNA expression levels across various breast cancer cell lines, categorized by intrinsic subtypes, utilizing data from the Cancer Cell Line Encyclopedia (CCLE) database. Statistical significance was determined by a two-tailed Student's t-test or Wilcoxon rank-sum test. Survival differences were evaluated by the log-rank test.

**
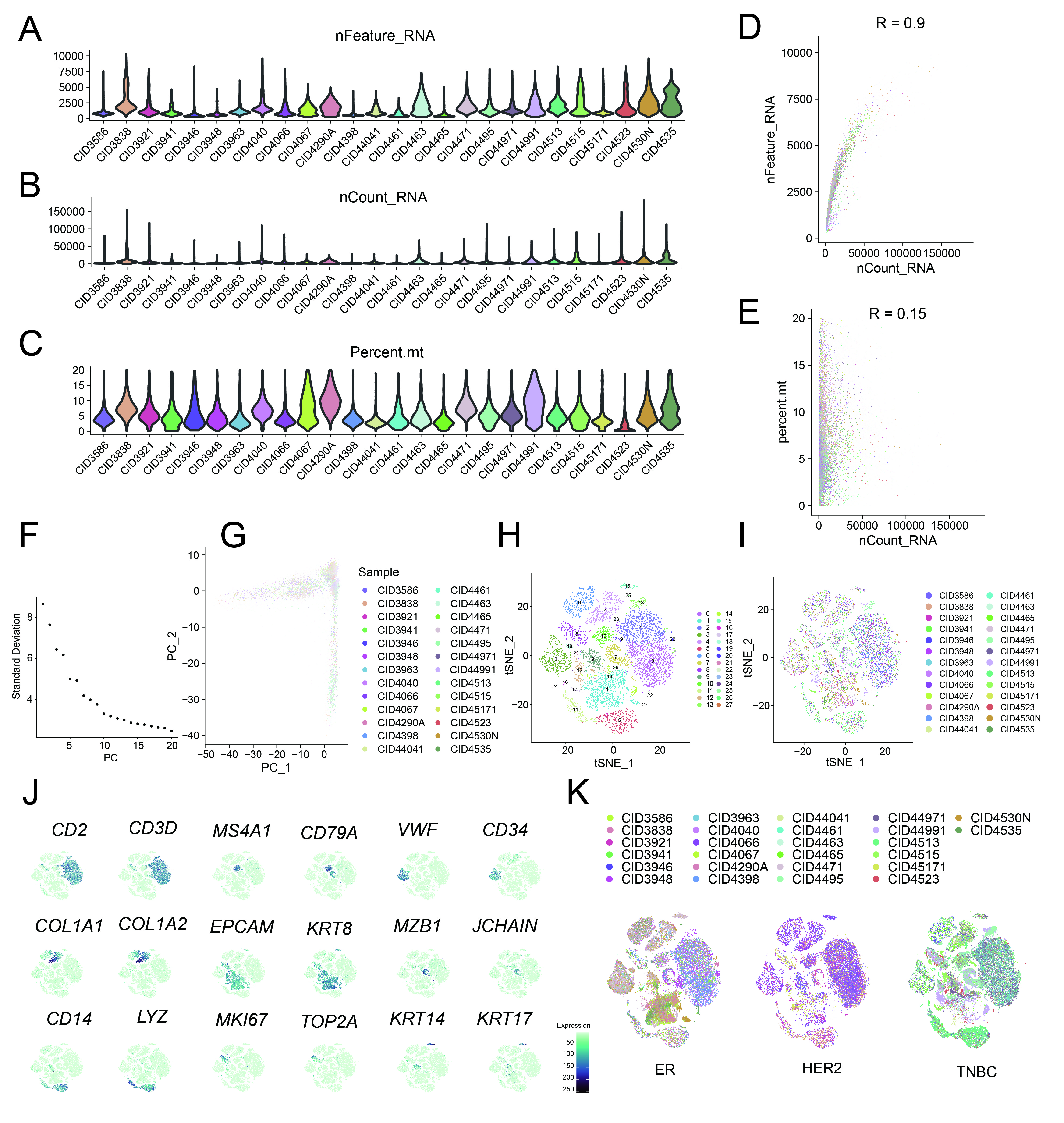
**

**Figure S2.** Quality Control and Integrated Analysis of scRNA-seq Data from 26 Breast Cancer Patient Samples (GSE176078). (A-C) Violin plots displaying the distribution of quality control metrics for single cells: (A) number of genes detected (nFeature_RNA), (B) total UMI counts (nCount_RNA), and (C) percentage of mitochondrial gene expression (percent.mt). (D, E) Correlation analysis between QC metrics, showing a strong positive correlation between nFeature_RNA and nCount_RNA (D), and a weak correlation between nCount_RNA and percent.mt (E). (F) Elbow plot showing the standard deviation of principal components (PCs), used to determine the optimal dimensionality for downstream analysis. (G) PCA plot illustrating cell distribution along the first two principal components (PC_1 and PC_2). (H) t-SNE visualization of all single cells, colored by computationally identified cell clusters. (I) t-SNE plot of cell clusters, colored by the patient sample of origin to assess batch effects. (J) Feature plots overlaid on the t-SNE map, showing the expression and distribution of canonical marker genes for cell type identification. (K) Overview t-SNE plot of 96,976 single cells, with colors representing their originating clinical sample.

**
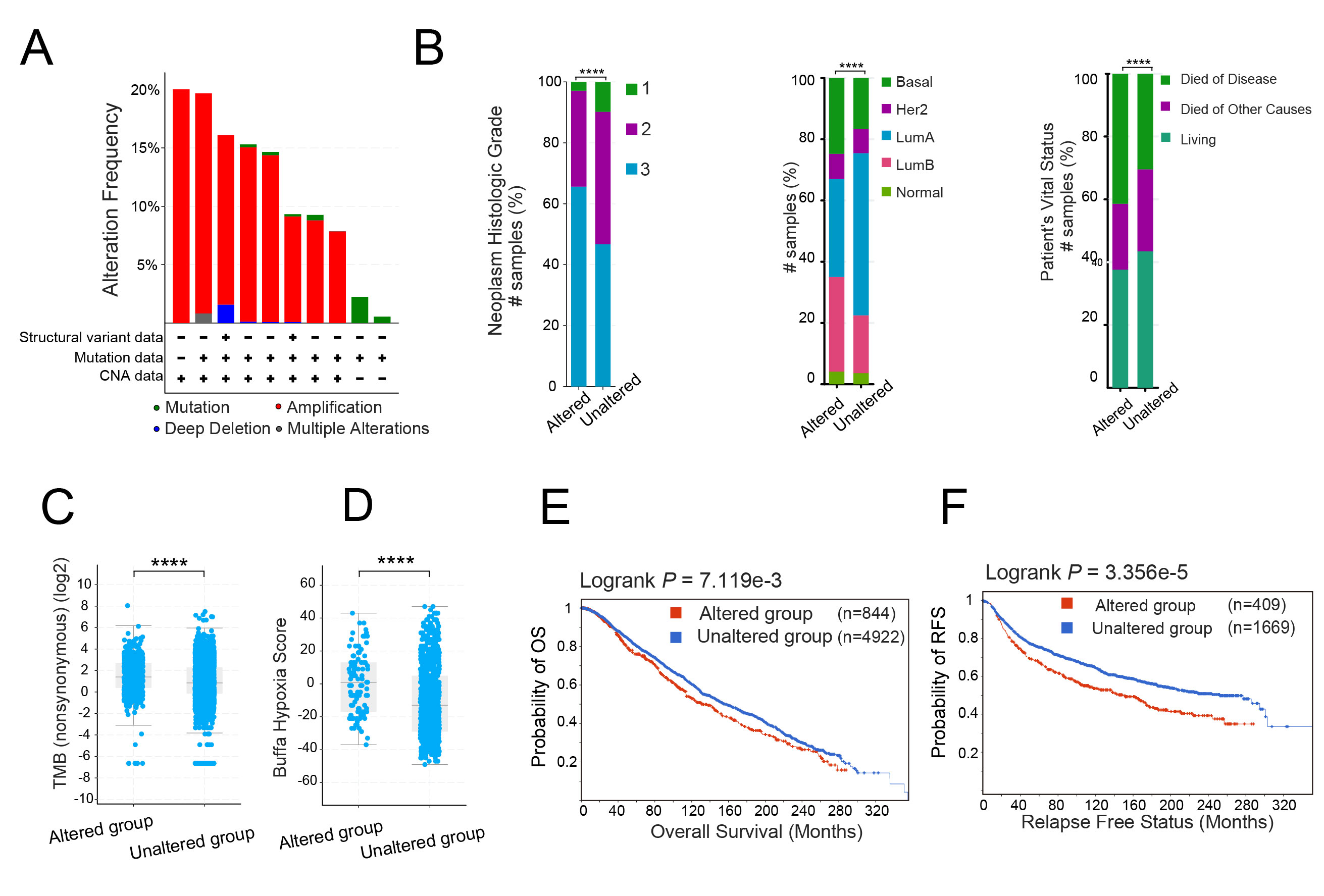
**

**Figure S3.** Genomic Landscape, Clinicopathological associations, and Prognostic Value of *TSPYL5* in Breast Cancer. (A) OncoPrint visualization of *TSPYL5* genomic alterations across 10 breast cancer patient cohorts from cibioportal datasets. (B) Distribution of clinicopathological features—including histological grade, PAM50 intrinsic subtypes, and patient survival status—stratified by *TSPYL5* alteration status (altered vs. unaltered). (C, D) Box plots comparing tumor mutational burden (TMB) (C) and the Buffa hypoxia scores (D) between patients with and without *TSPYL5* genomic alterations*.* (E, F) Kaplan-Meier curves evaluating the association between *TSPYL5* mRNA expression levels and patient prognosis, specifically overall survival (OS) (E) and relapse-free survival (RFS) (F). Prognostic data were obtained from cBioPortal. Differences in continuous variables were determined using a two-tailed Student's t-test or Wilcoxon rank-sum test. Differences in categorical clinical features were analyzed via the Chi-square test (or Fisher's exact test). Survival differences were evaluated by the log-rank test. **P* < 0.05, ***P* < 0.01, ****P* < 0.001, *****P* < 0.0001.

**
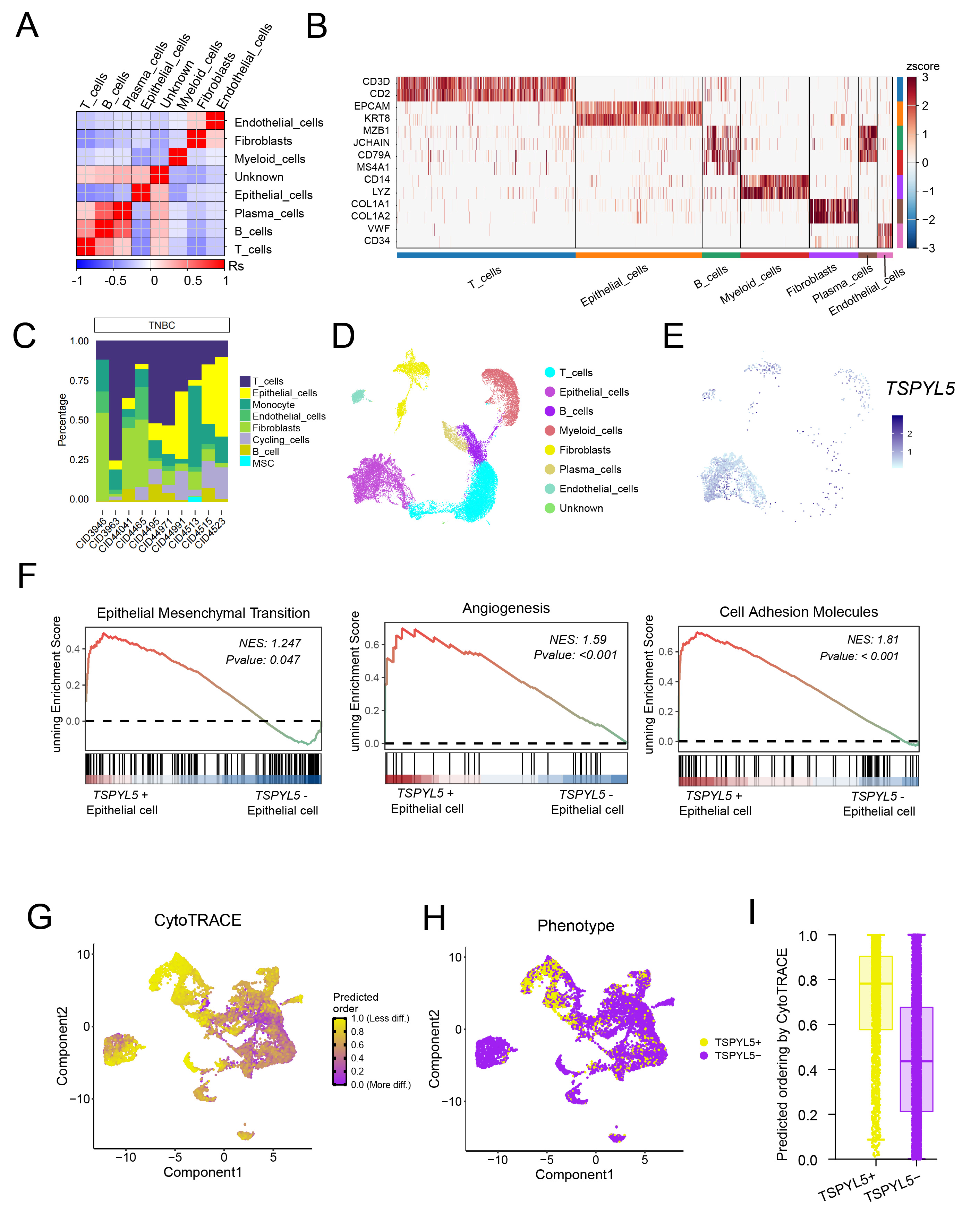
**

**Figure S4.** Single-Cell Transcriptomic Analysis of the TNBC Tumor Microenvironment and Characterization of *TSPYL5*-Expressing Cells. (A) Analysis was performed on a subset of 40,982 single cells from TNBC tissues. Heatmap displaying the strength of inferred cell-cell communication networks between major cell populations in the TNBC microenvironment. (B) Heatmap showing the expression of canonical marker genes used for the annotation of distinct cell types. (C) Stacked bar plot illustrating the relative proportions of cell types across individual TNBC patient samples. (D) UMAP visualization of 40,982 single cells derived from TNBC tissues, with cells colored by their annotated cell type. (E) Feature plot overlaid on the UMAP, demonstrating the specific expression of *TSPYL5* within epithelial cell clusters. (F) GSEA plots indicating that *TSPYL5*-positive epithelial cells are significantly enriched for hallmark gene signatures associated with metastasis, including Epithelial-Mesenchymal Transition (EMT), angiogenesis, and cell adhesion. (G, H) UMAP visualization of the epithelial cell subset. Cells are colored by their stemness score as computed by CytoTRACE (G), and their *TSPYL5* expression status (*TSPYL5*+ in yellow, *TSPYL5*− in purple) (H), showing a clear co-localization. (I) Box plot providing statistical comparison of CytoTRACE stemness scores, showing significantly higher scores in *TSPYL5*+ versus *TSPYL5*− epithelial cells.


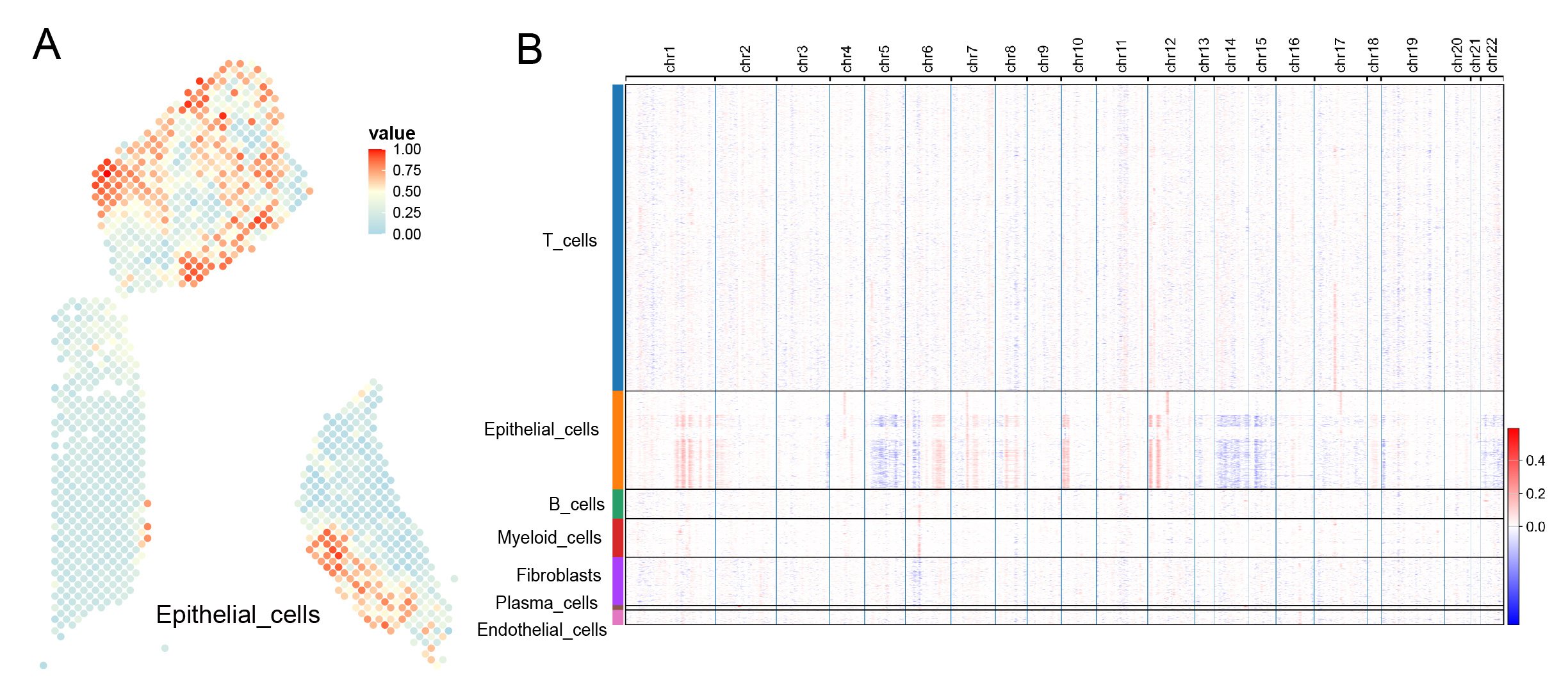


**Figure S5.** Spatial Transcriptomic Analysis of a TNBC Tissue Section to Validate the Cellular Origin of *TSPYL5.* (A) Spatial feature plot from a TNBC tissue section showing the *in situ* co-localization of *TSPYL5* expression with spots annotated as epithelial cells. The overlay confirmed that *TSPYL5* is predominantly expressed within the tumor's epithelial compartment. (B) Heatmap of inferred copy number variations (CNVs) across major cell populations. The analysis revealed a distinct aneuploid profile (gains and losses) in the epithelial cell cluster, confirming its malignant identity.

**
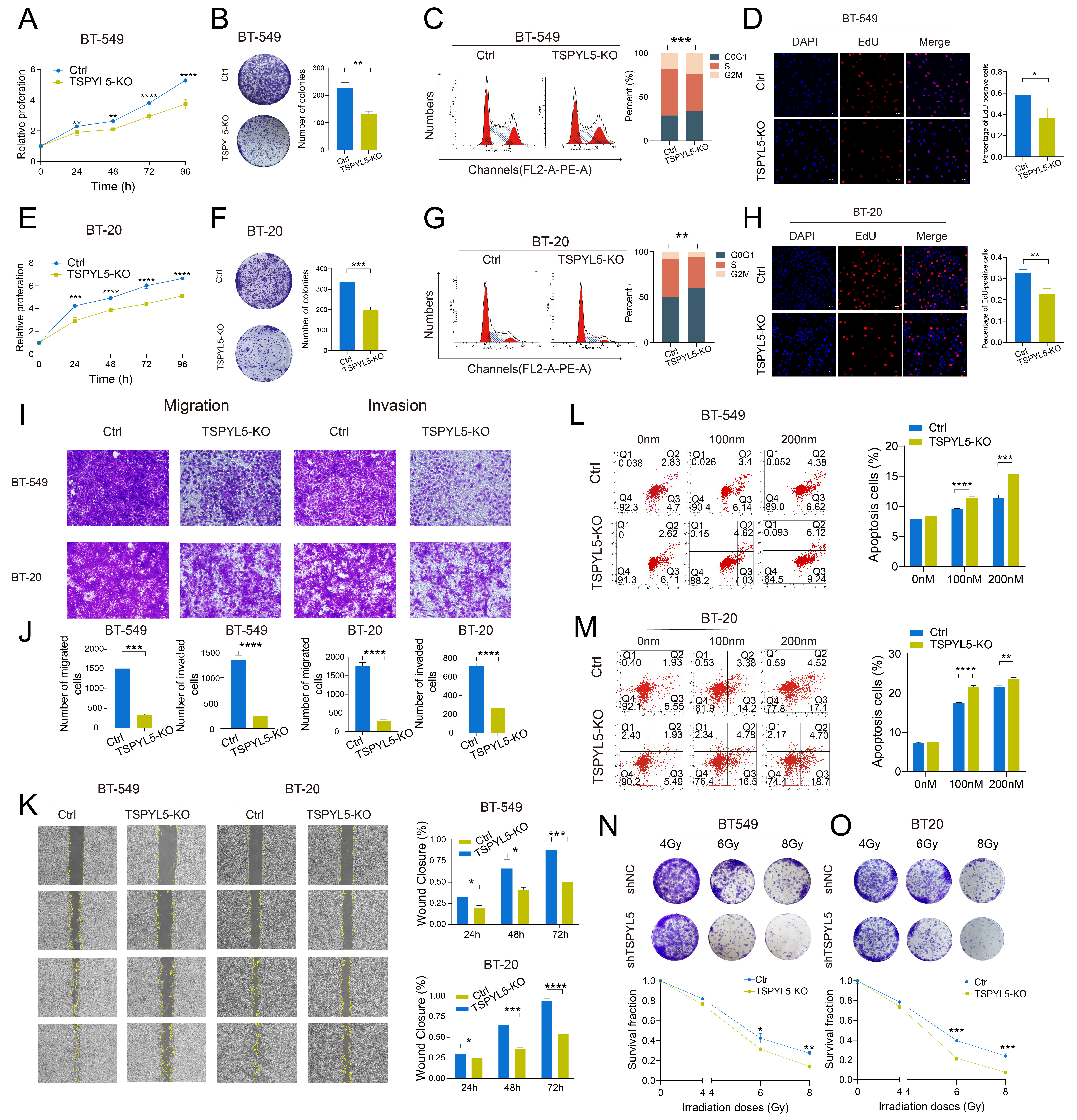
**

**Figure S6.** Genetic Depletion of *TSPYL5* Suppresses Malignant Phenotypes and Enhances Therapeutic Sensitivity in TNBC Cell Lines. (A-D) TSPYL5-knockout in BT-549 cells suppressed cell proliferation (A), colony formation (B), G1/S phase transition (C) and DNA synthesis (D). (E-H) TSPYL5-knockout in BT-20 cells suppressed cell proliferation (E), colony formation (F), G1/S phase transition (G) and DNA synthesis (H). (I, J) Transwell migration and invasion assays showing that TSPYL5-knockout suppressed the migratory and invasive capacity of BT-549 and BT-20 cells. (K) Wound-healing assay showing that TSPYL5- knockout suppressed cell migration. (L, M) Flow cytometric analysis of apoptosis revealing that TSPYL5-knockout aggravated paclitaxel-induced cell apoptosis as indicated by increased Annexin V-positive populations. (N, O) TSPYL5-knockout decreased the cell livability after the γ-irradiation treatment. Data are presented as mean ± SD from at least three independent experiments. Representative images are shown. Statistical significance was determined by a two-tailed Student's t-test or one-way ANOVA. **P* < 0.05, ***P* < 0.01, ****P* < 0.001, *****P* < 0.0001; ns, not significant.

**
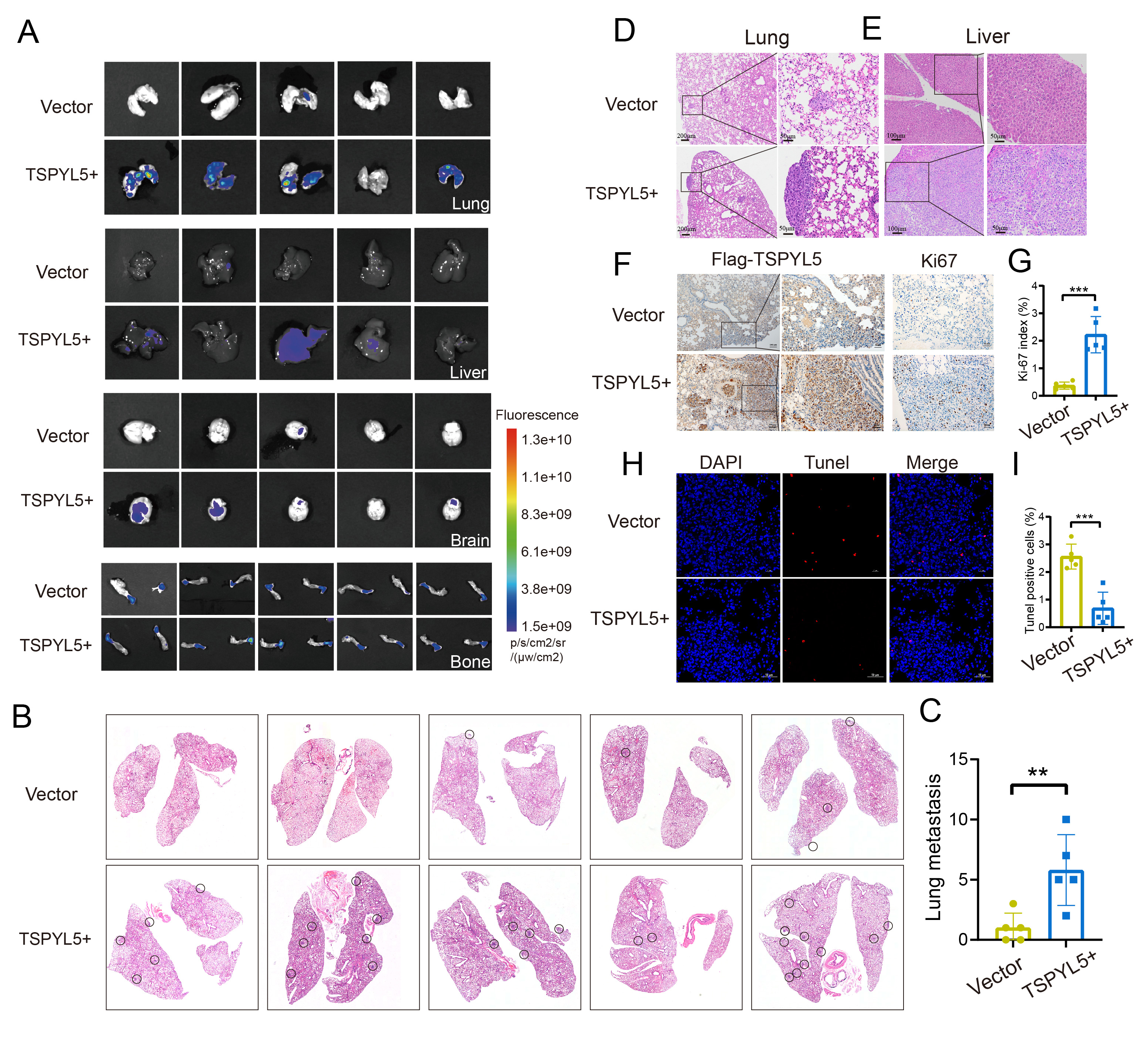
**

**Figure S7.** TSPYL5 Promotes Metastatic Colonization and Enhances the Survival of Cancer Cells in Distant Organs. (A) Representative *ex vivo* fluorescence imaging of major organs at four weeks post-injection revealed increased metastatic burden (lungs, liver, brain, bone) in the TSPYL5+ group. (B) Representative H&E-stained sections of lungs, revealing a significant increase in the number of metastatic foci in the TSPYL5+ group. (C) Quantification of metastatic foci per lung. (D, E) High-magnification H&E images showing the morphology of established metastatic lesions in lung (D) and liver (E). (F) Immunohistochemical (IHC) staining for the Flag epitope, confirming expression of exogenous Flag-TSPYL5 within metastatic nodules. Representative IHC for the proliferation marker Ki67 in lung metastases. (G) Quantification of the Ki67-positive cell percentage. (H) Representative immunofluorescence images of a TUNEL assay to detect apoptotic cells in lung metastases. (I) Quantification of the percentage of TUNEL-positive cells. **P* < 0.05, ***P* < 0.01, ****P* < 0.001, **** *P* < 0.0001; ns, not significant.

**
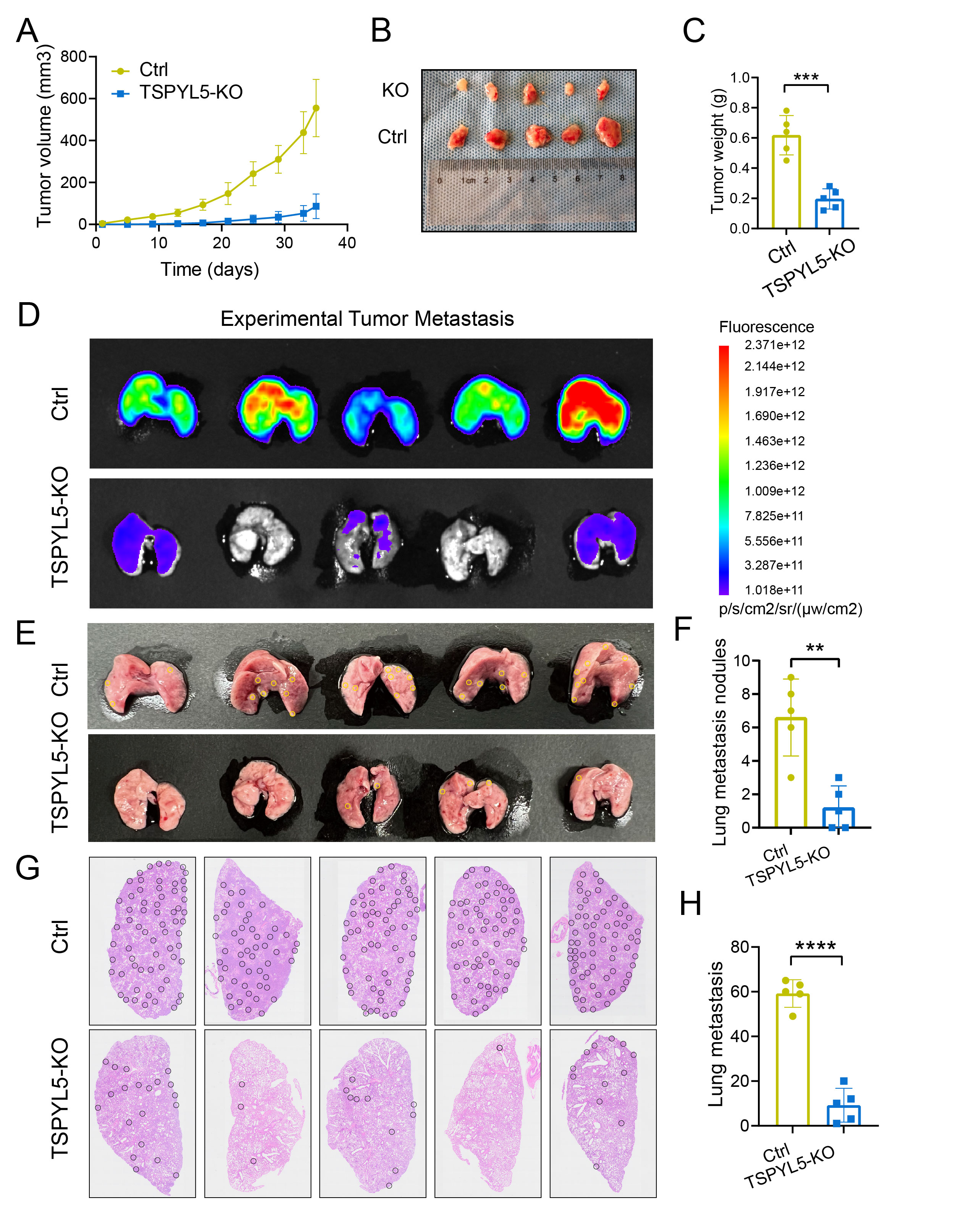
**

**Figure S8.** TSPYL5 depletion suppresses primary TNBC growth and lung metastasis *in vivo*. (A) Primary tumor growth curves of mice implanted with control (Ctrl) or *TSPYL5*-knockout (KO) BT-549 cells. Tumor volumes were monitored at the indicated time points. Representative macroscopic images (B) and final weight quantification (C) of the resected primary tumors at the study endpoint (n = 5 mice per group). (D) *Ex vivo* fluorescence imaging of of excised lungs illustrating the metastatic burden. (E, F) Representative gross photographs of the lungs (E) and quantitative analysis of macroscopically visible metastatic nodules (F). Yellow circles indicate surface metastatic lesions. (G, H) Representative hematoxylin and eosin (H&E)-stained lung sections displaying metastatic lesions (G), with corresponding quantification of histologically confirmed metastatic foci per section (H). Data are presented as mean ± SD. Statistical significance was determined by an unpaired two-tailed Student’s t-test for endpoint analyses. **P* < 0.05, ***P* < 0.01, ****P* < 0.001, *****P* < 0.0001.

**
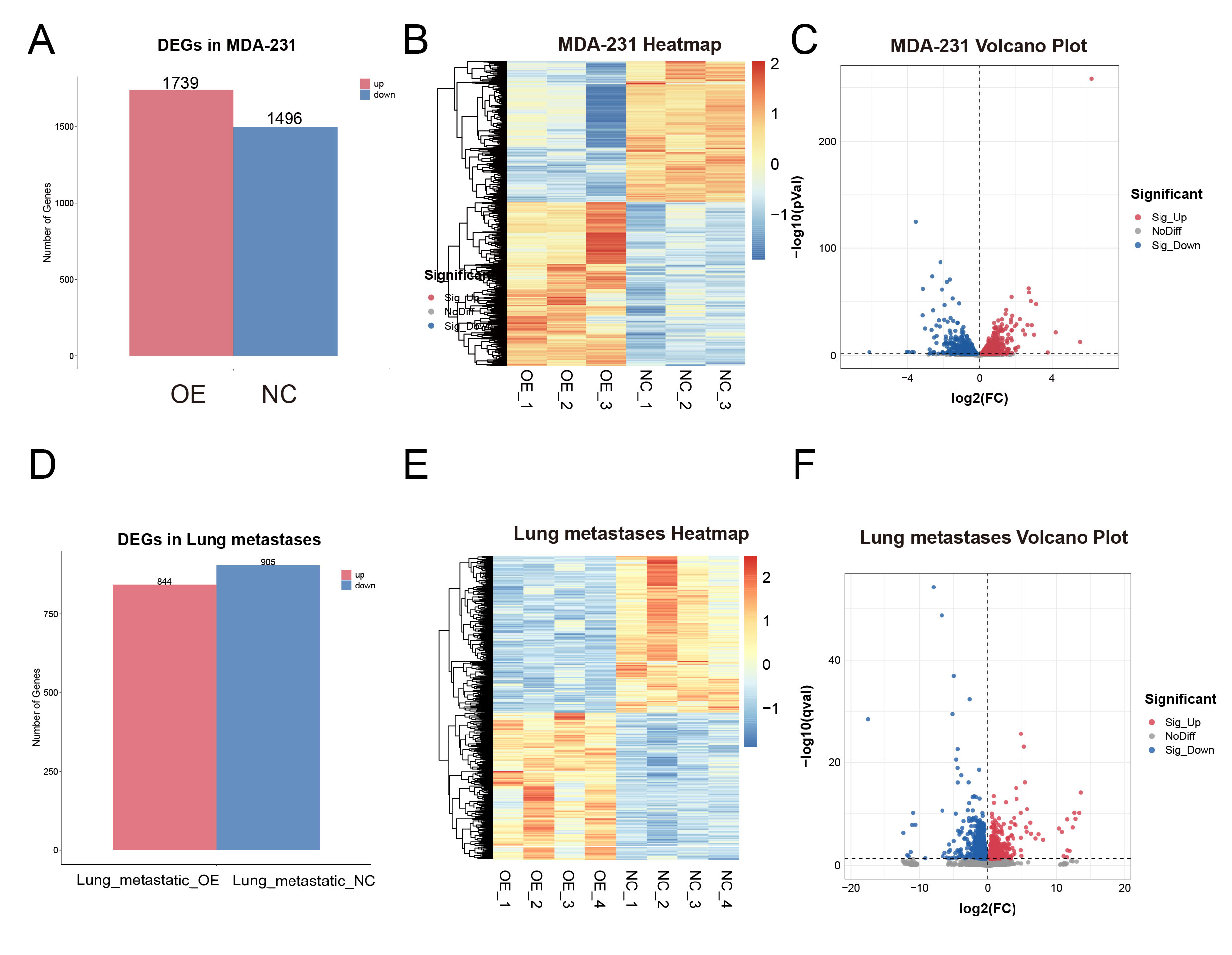
**

**Figure S9.** RNA-sequencing analysis of differentially expressed genes (DEGs). (A-C) Bar chart, heatmap, and volcano plot of DEGs in TSPYL5-overexpressing (OE) versus negative control (NC) MDA-MB-231 cells. (D-F) Bar chart, heatmap, and volcano plot of DEGs in lung metastases from an experimental model using TSPYL5-OE versus NC cells. Red and blue indicate upregulated and downregulated genes, respectively.

**
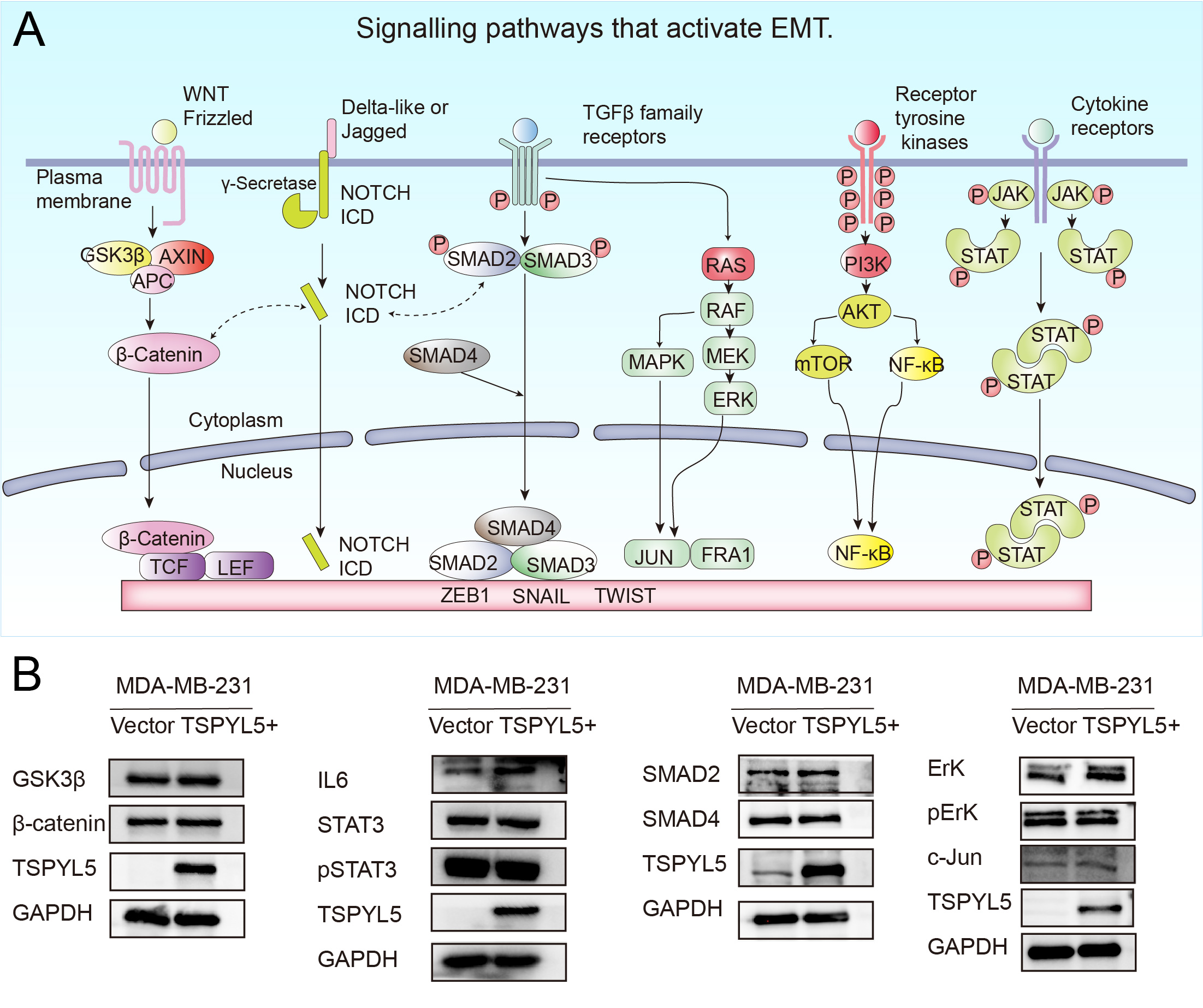
**

**Figure S10.** Investigation of EMT-Related Signaling Pathways Modulated by TSPYL5. (A) Schematic diagram illustrating the major signaling pathways known to regulate the EMT process, including TGF-β/Smad, Wnt/β-catenin, MAPK, and JAK/STAT signaling. The figure was adapted from Dongre A et al ^[1]^. (B) Western blot analysis of key protein effectors in several EMT-related pathways following *TSPYL5* overexpression in MDA-MB-231 cells. The blot assesses components of the Wnt/β-catenin, TGF-β, MAPK/ERK, and JAK/STAT3 signaling cascades.

**
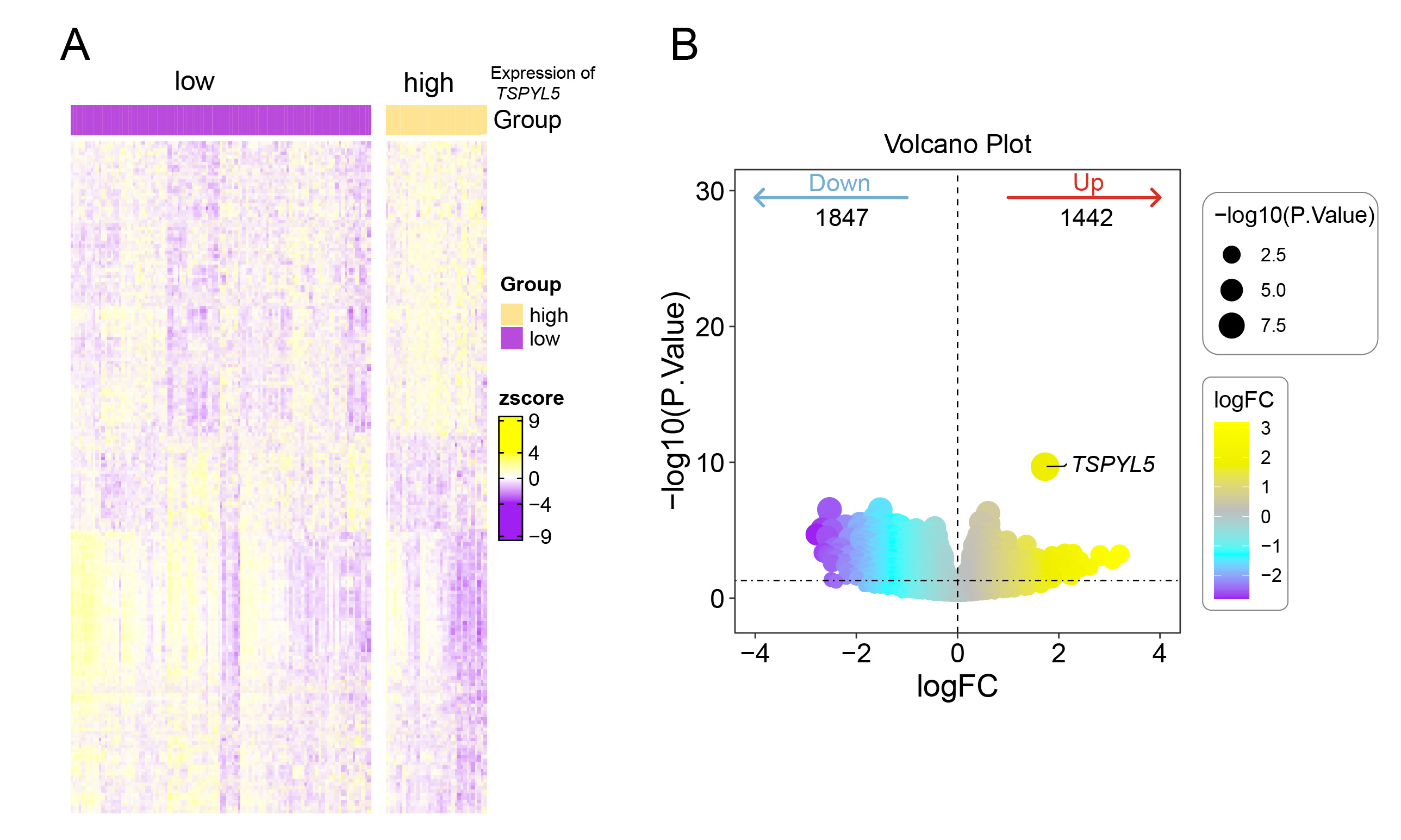
**

**Figure S11.** Differential Gene Expression Analysis in TCGA Basal-Like Breast Cancer Stratified by TSPYL5 Expression. (A) Analysis was performed using bulk RNA-seq data from the TCGA-BLBC cohort. Patients were stratified into *TSPYL5*-high (top quartile) and *TSPYL5*-low (bottom quartile) expression groups. Heatmap showing hierarchical clustering of DEGs between the *TSPYL5*-high and *TSPYL5*-low groups. (B) Volcano plot illustrating the log2 fold change and statistical significance of all detected genes.

**
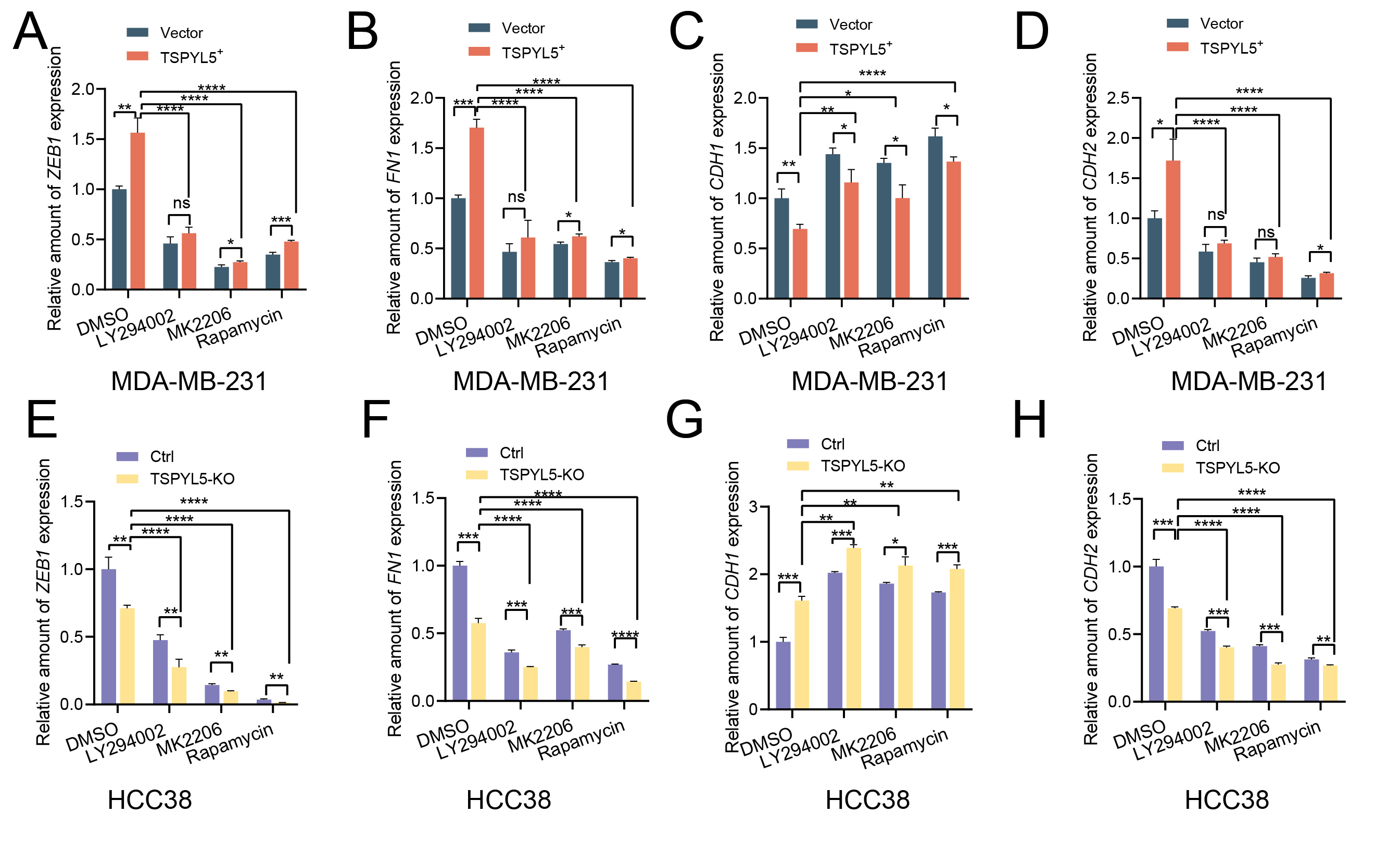
**

**Figure S12.** Pharmacological Inhibition of the PI3K/AKT/mTOR Axis Reverses *TSPYL5*-Mediated Regulation of the EMT Transcriptional Program. (A-D) RT-qPCR analysis of EMT markers in TSPYL5+ MDA-MB-231 cells following treatment with a PI3K inhibitor (LY294002), an AKT inhibitor (MK-2206), or an mTOR inhibitor (Rapamycin). Expression levels of (A) *ZEB1*, (B) *FN1*, (C) *CDH1*, and (D) *CDH2* were quantified. (E-H) RT-qPCR analysis of EMT markers in *TSPYL5*-KO HCC38 cells treated with the same panel of inhibitors. Expression levels of (E) *ZEB1*, (F) *FN1*, (G) *CDH1*, and (H) *CDH2* were quantified. Data are shown as mean ± SD from three independent experiments. Statistical significance was determined by one-way ANOVA or Student's t-test. **P* < 0.05, ***P* < 0.01, ****P* < 0.001, **** *P* < 0.0001; ns, not significant.

**
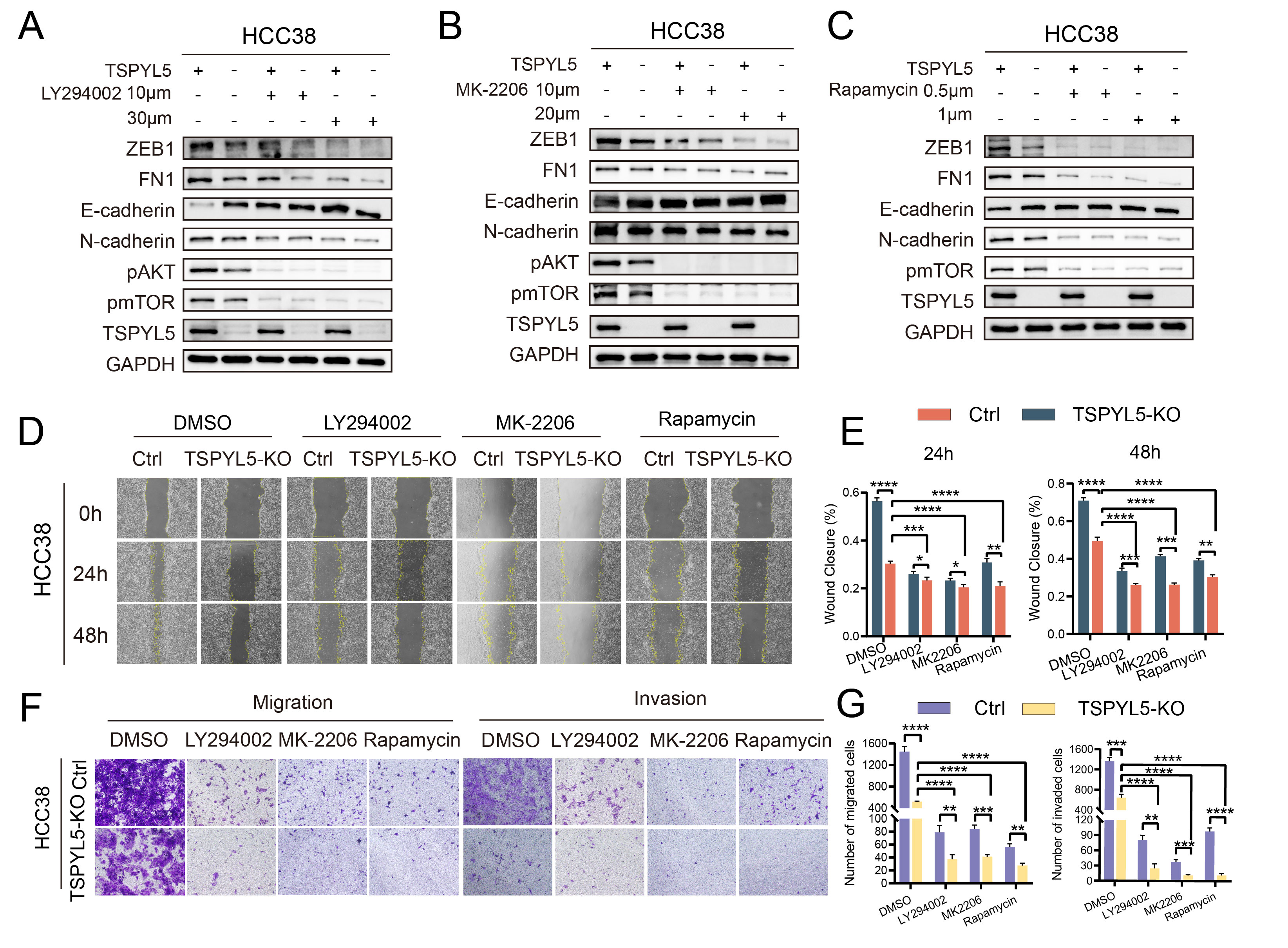
**

**Figure S13.** Genetic Depletion of TSPYL5 Cooperates with Pharmacological Blockade of PI3K/AKT/mTOR Signaling to Inhibit TNBC Cell Motility. (A-C) Western blot analysis of key EMT marker proteins in TSPYL5-KO HCC38 cells treated with the PI3K inhibitor LY294002 (A), the AKT inhibitor MK-2206 (B), or the mTOR inhibitor Rapamycin (C). (D, E) Wound healing assays showing that inhibition of the PI3K-AKT-mTOR pathway further suppressed the migration of TSPYL5-KO cells. Representative images (D) and quantification (E) are shown. (F, G) Transwell migration and invasion assays demonstrating that pharmacological blockade of the PI3K-AKT-mTOR pathway additively suppressed the migratory and invasive capacity of the *TSPYL5*-KO HCC38 cells. Representative images (F) and quantification (G) are shown. All data are presented as mean ± SD from at least three independent biological replicates. Statistical analyses were performed using one-way ANOVA or Student's t-test. **P* < 0.05, ***P* < 0.01, ****P* < 0.001, **** *P* < 0.0001; ns, not significant.

**
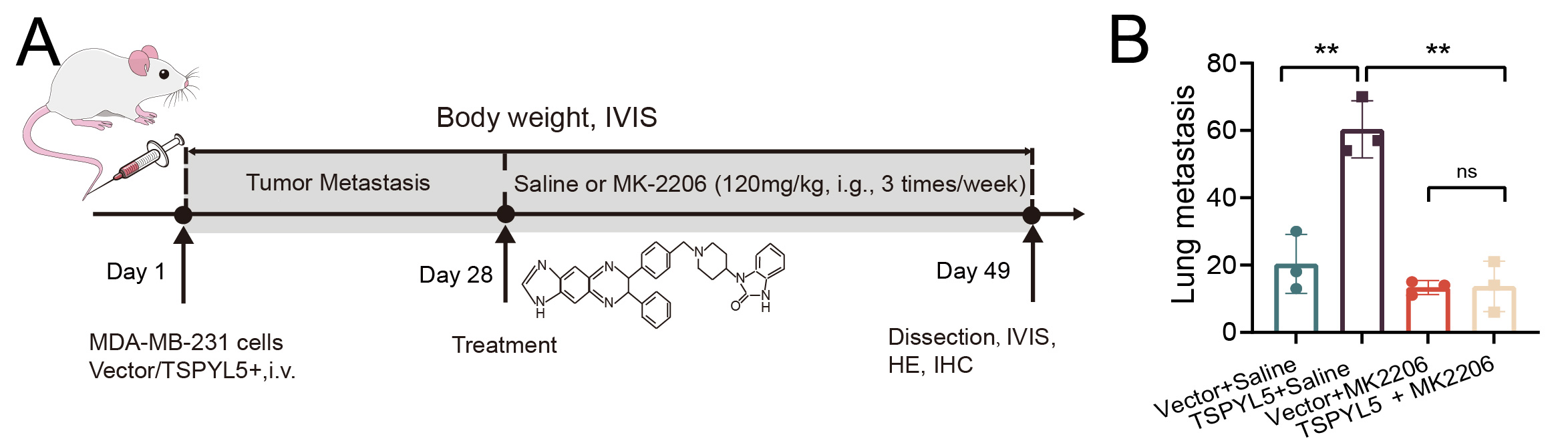
**

**Figure S14.** Pharmacological Inhibition of AKT Suppresses Experimental Lung Metastasis *In Vivo*. (A) Schematic diagram illustrating the design of the experimental metastasis model. Mice received intravenous (i.v.) injection of tumor cells, followed by treatment with the AKT inhibitor MK-2206 or a vehicle control. Metastatic progression was monitored. (B) Quantification of lung metastatic burden. The bar graph showed the number of metastatic nodules counted from Hematoxylin and Eosin (H&E) stained lung sections, demonstrating that the enhanced metastatic burden driven by TSPYL5 overexpression was reversed by pharmacological inhibition of AKT with MK-2206. Data are presented as mean ± SD (n = 3 mice per group). Significance was determined using a Student's t-test. **P* < 0.05, ***P* < 0.01, ****P* < 0.001, **** *P* < 0.0001; ns, not significant.

**
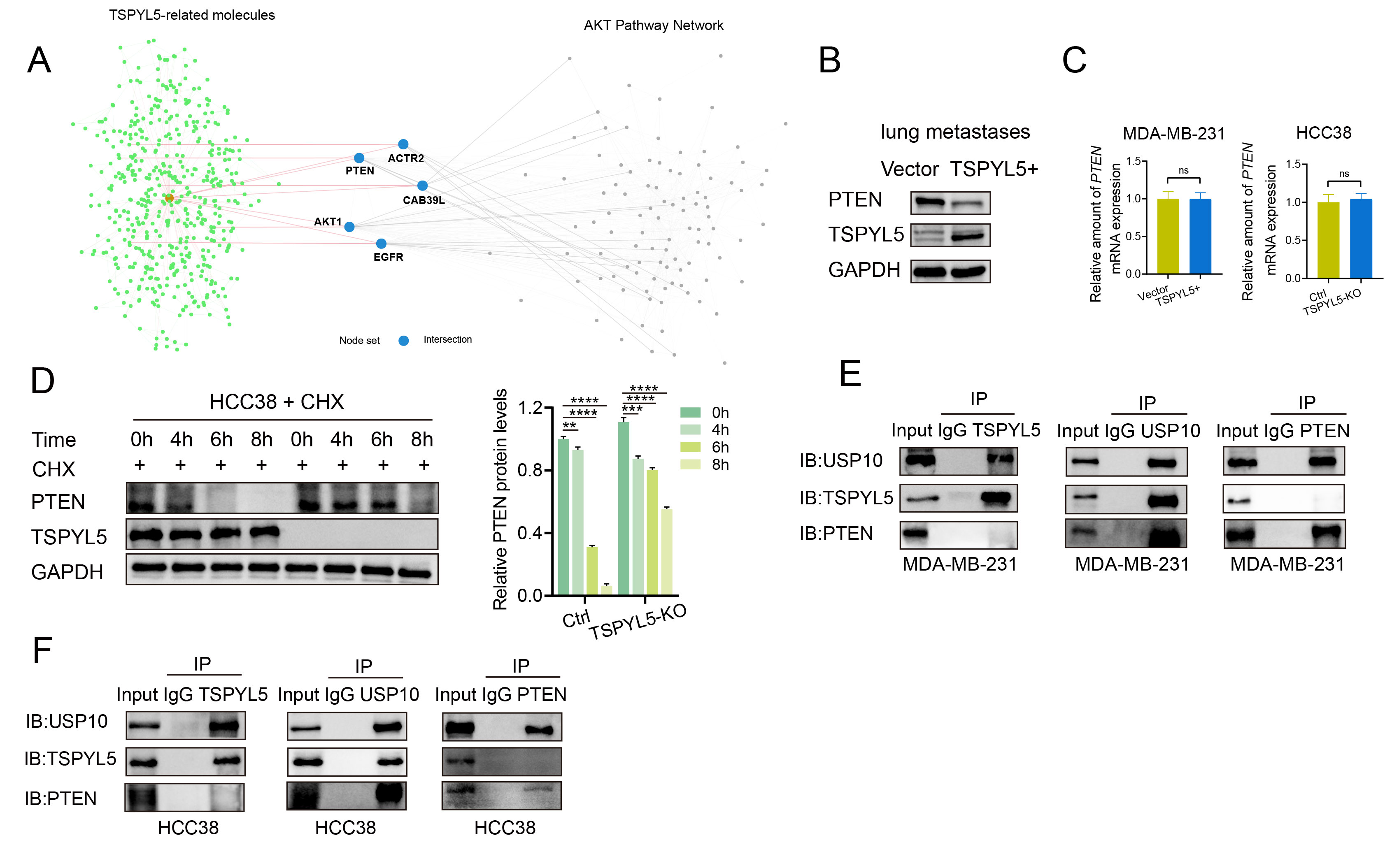
**

**Figure S15.** TSPYL5 Mediates Post-Translational Downregulation of PTEN by Promoting its Proteasomal Degradation and Interacting with the USP10 Complex. (A) Protein-protein interaction (PPI) network illustrating the intersection of TSPYL5-associated proteins and MSigDB-annotated AKT regulators. PTEN is highlighted as the central bridging node. Nodes represent proteins; edges denote interactions. (B) Western blot revealing decreased PTEN protein levels in *in vivo* lung metastases overexpressing TSPYL5. (C) RT-qPCR analysis demonstrating that *PTEN* mRNA levels remain unchanged upon TSPYL5 overexpression (TSPYL5+) in MDA-MB-231 cells or knockout (KO) in HCC38 cells. (D) Cycloheximide (CHX) chase assay demonstrating that TSPYL5 accelerates the degradation of PTEN protein in HCC38 cells. (E) Co-immunoprecipitation (Co-IP) assays revealing the endogenous interactions among TSPYL5, USP10, and PTEN in MDA-MB-231 cells (E) and HCC38 cells (F). Data in C are presented as mean ± SD. Statistical significance was determined by Student’s t-test; **P* < 0.05, ***P* < 0.01, ****P* < 0.001, **** *P* < 0.0001; ns, not significant.

**
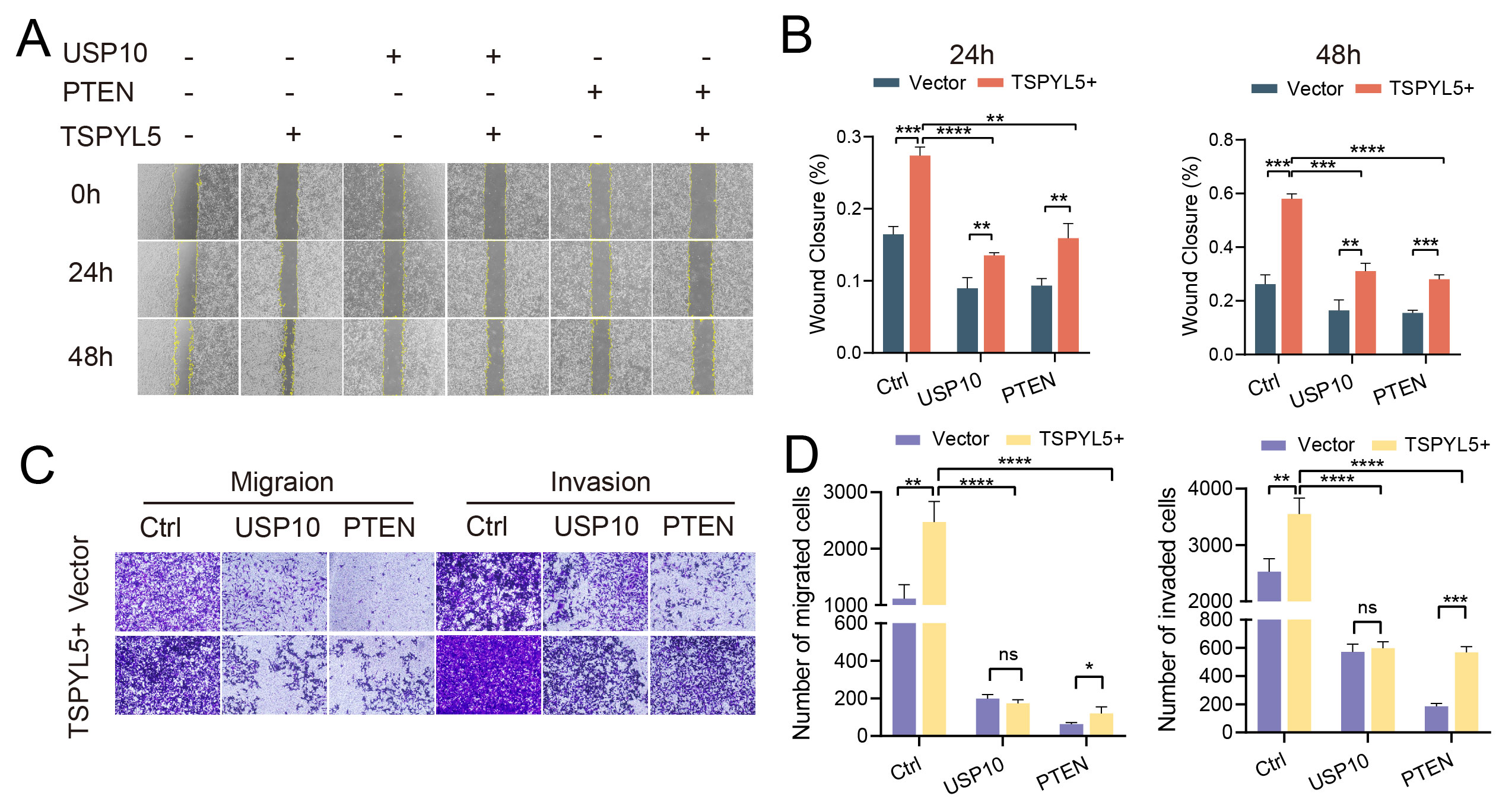
**

**Figure S16.** Overexpression of USP10 or PTEN Abrogates TSPYL5-driven Cell Migration and Invasion. (A, B) Wound healing assays showing that overexpression of USP10 or PTEN suppresses the enhanced migratory capacity of TSPYL5-overexpression MDA-MB-231 cells. Representative images (A) and the corresponding quantitative analysis (B) are shown. (C, D) Transwell migration and invasion assays confirming that overexpression of either USP10 or PTEN reverses the pro-migratory and pro-invasive phenotypes induced by TSPYL5. Representative micrographs (C) and quantifications (D) are provided. Data in B and D are presented as mean ± SD of n = 3 independent experiments. Statistical significance was determined by One-way ANOVA; **P* < 0.05, ***P* < 0.01, ****P* < 0.001, **** *P* < 0.0001; ns, not significant*.*

**
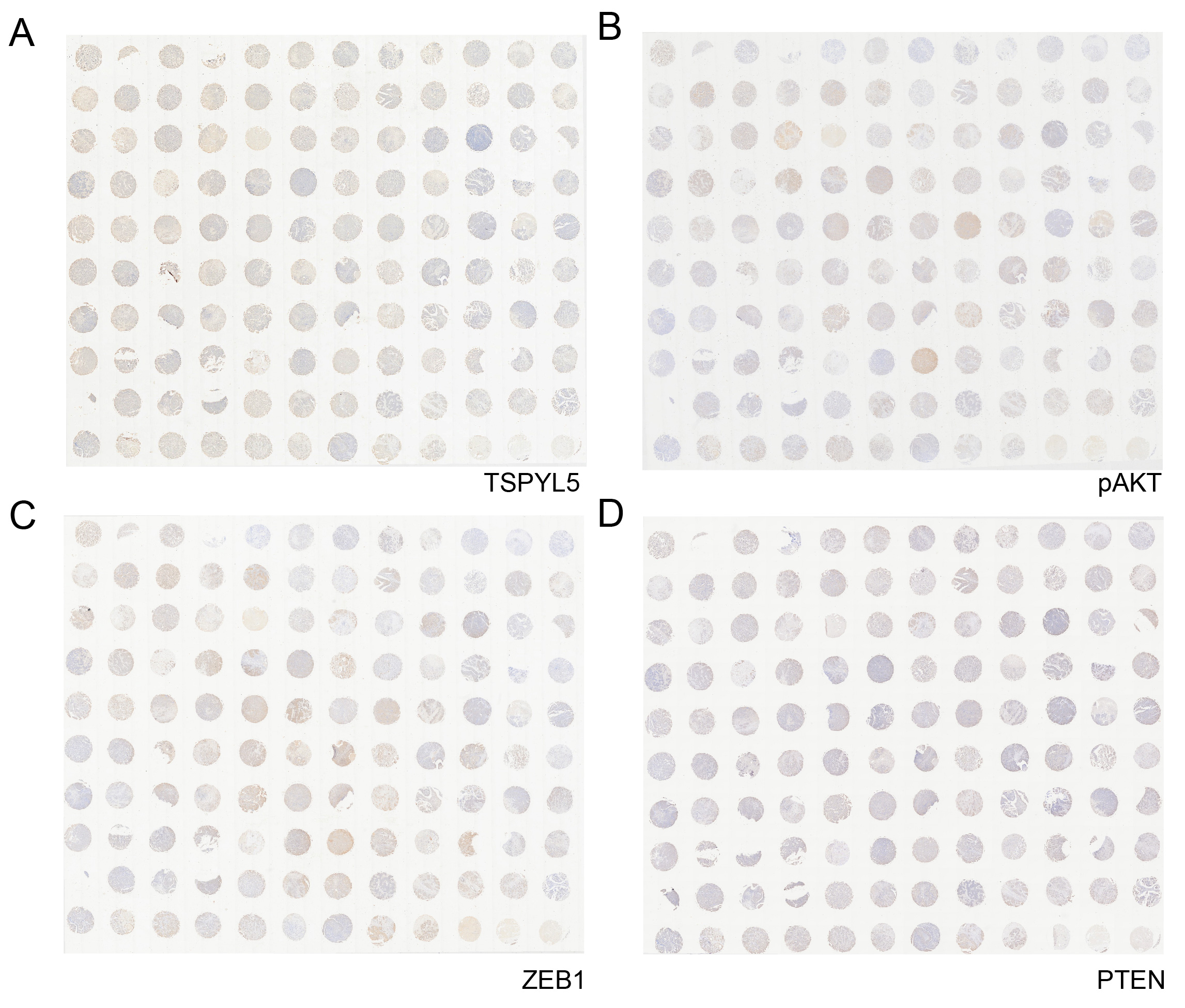
**

**Figure S17.** Overview of the TSPYL5, p-AKT, ZEB1, and PTEN Expression Landscape in a TNBC-Enriched Tissue Microarray Cohort. This figure presents a comprehensive overview of IHC staining for key proteins across an entire tissue microarray (TMA). The TMA contains 120 tissue cores, comprising 110 TNBC, 4 adjacent normal mammary tissues, and 6 cases of other breast cancer subtypes (ER+, PR+, HER2+). The composite images display the complete staining results for: TSPYL5 (A), p-AKT (B), ZEB1 (C) and PTEN (D).

**Table S1 Clinicopathological data for cohorts of 120 breast cancer patients**

| No. | Age | Sex | Pathology diagnosis | Grade | TNM | Stage | Type | ER | PR | Her-2 |
| --- | --- | --- | --- | --- | --- | --- | --- | --- | --- | --- |
| 1 | 36 | F | Invasive carcinoma of no special type | 1 | T2N0M0 | IIA | Malignant | - | - | 0 |
| 2 | 52 | F | Invasive carcinoma of no special type | 1--2 | T2N2M0 | IIIA | Malignant | - | - | 0 |
| 3 | 71 | F | Invasive carcinoma of no special type | 2 | T2N0M0 | IIA | Malignant | - | - | 0 |
| 4 | 50 | F | Invasive carcinoma of no special type | 2 | T2N1M0 | IIB | Malignant | - | +，15 | 0 |
| 5 | 55 | F | Invasive carcinoma of no special type | 2 | T3N0M0 | IIB | Malignant | - | - | 0 |
| 6 | 50 | F | Invasive carcinoma of no special type | 2 | T3N0M0 | IIB | Malignant | - | - | 0 |
| 7 | 55 | F | Invasive carcinoma of no special type | 2 | T2N0M0 | IIA | Malignant | - | +，7% | 0 |
| 8 | 68 | F | Invasive carcinoma of no special type | 2 | T1N0M0 | IA | Malignant | - | +，20% | 0 |
| 9 | 39 | F | Invasive carcinoma of no special type (breast tissue) | * | T1N0M0 | IA | Malignant | - | - | 0 |
| 10 | 46 | F | Invasive carcinoma of no special type | 2 | T1N0M0 | IA | Malignant | - | - | 0 |
| 11 | 50 | F | Invasive carcinoma of no special type | 2 | T2N0M0 | IIA | Malignant | - | - | 0 |
| 12 | 50 | F | Invasive carcinoma of no special type with necrosis | 2 | T2N0M0 | IIA | Malignant | - | - | 0 |
| 13 | 45 | F | Invasive carcinoma of no special type | 2 | T2N0M0 | IIA | Malignant | - | - | 0 |
| 14 | 59 | F | Invasive carcinoma of no special type | 2 | T2N1M0 | IIB | Malignant | +，5% | - | 0 |
| 15 | 59 | F | Invasive carcinoma of no special type | 2 | T2N0M0 | IIA | Malignant | - | - | 0 |
| 16 | 45 | F | Invasive carcinoma of no special type | 2 | T4N0M0 | IIIB | Malignant | - | - | 0 |
| 17 | 71 | F | Invasive carcinoma of no special type | 2 | T2N1M0 | IIB | Malignant | - | weakly+，3% | 0 |
| 18 | 46 | F | Invasive carcinoma of no special type | 2 | T1N0M0 | IA | Malignant | - | - | 0 |
| 19 | 48 | F | Invasive carcinoma of no special type | 2 | T3N0M0 | IIB | Malignant | - | - | 0 |
| 20 | 55 | F | Invasive carcinoma of no special type | 2 | T2N1M0 | IIB | Malignant | - | weakly+，5% | 0 |
| 21 | 47 | F | Invasive carcinoma of no special type | 2 | T3N2M0 | IIIA | Malignant | - | - | 0 |
| 22 | 53 | F | Invasive carcinoma of no special type | 2 | T2N0M0 | IIA | Malignant | - | - | 0 |
| 23 | 55 | F | Invasive carcinoma of no special type | 2 | T2N0M0 | IIA | Malignant | - | - | 0 |
| 24 | 49 | F | Invasive carcinoma of no special type with necrosis | 2 | T2N1M0 | IIB | Malignant | - | - | 0 |
| 25 | 42 | F | Invasive carcinoma of no special type | 2 | T2N2M0 | IIIA | Malignant | - | - | 0 |
| 26 | 58 | F | Invasive carcinoma of no special type | 2 | T4N1M0 | IIIB | Malignant | - | - | 0 |
| 27 | 40 | F | Invasive carcinoma of no special type | 2 | T3N0M0 | IIB | Malignant | - | - | 0 |
| 28 | 85 | F | Invasive carcinoma of no special type with necrosis | 2 | T4N0M0 | IIIB | Malignant | - | - | 0 |
| 29 | 81 | F | Invasive carcinoma of no special type (sparse breast tissue) | 2 | T3N0M0 | IIB | Malignant | - | - | 0 |
| 30 | 42 | F | Invasive carcinoma of no special type | 2 | T2N0M0 | IIA | Malignant | - | - | 0 |
| 31 | 63 | F | Invasive carcinoma of no special type with necrosis | 2 | T3N0M0 | IIB | Malignant | - | +，10% | weakly, 1+ |
| 32 | 46 | F | Invasive carcinoma of no special type | 2 | T3N0M0 | IIB | Malignant | - | - | 0 |
| 33 | 53 | F | Invasive carcinoma of no special type | 2 | T2N2M0 | IIIA | Malignant | - | - | 0 |
| 34 | 31 | F | Invasive carcinoma of no special type | 2 | T2N0M0 | IIA | Malignant | - | ++，80% | 0 |
| 35 | 61 | F | Invasive carcinoma of no special type with necrosis | 2 | T2N0M0 | IIA | Malignant | - | - | 0 |
| 36 | 46 | F | Invasive carcinoma of no special type with degeneration | 2 | T2N0M0 | IIA | Malignant | - | - | 0 |
| 37 | 57 | F | Invasive carcinoma of no special type | 2 | T2N0M0 | IIA | Malignant | - | - | 0 |
| 38 | 63 | F | Invasive carcinoma of no special type | 2 | T2N0M0 | IIA | Malignant | - | - | 0 |
| 39 | 68 | F | Invasive carcinoma of no special type | 2 | T2N1M0 | IIB | Malignant | - | +，30% | 0 |
| 40 | 61 | F | Invasive carcinoma of no special type | 2--3 | T2N0M0 | IIA | Malignant | - | - | 0 |
| 41 | 64 | F | Invasive carcinoma of no special type with necrosis | 2 | T2N0M0 | IIA | Malignant | - | weakly+，5% | 0 |
| 42 | 39 | F | Invasive carcinoma of no special type | 2--3 | T2N0M0 | IIA | Malignant | - | - | 0 |
| 43 | 53 | F | Invasive carcinoma of no special type | 3 | T2N0M0 | IIA | Malignant | - | - | weakly, 1+ |
| 44 | 43 | F | Invasive carcinoma of no special type | 3 | T2N0M0 | IIA | Malignant | - | - | 0 |
| 45 | 70 | F | Invasive carcinoma of no special type with degeneration and necrosis | 3 | T2N0M0 | IIA | Malignant | - | - | 0 |
| 46 | 47 | F | Invasive carcinoma of no special type | 3 | T2N0M0 | IIA | Malignant | - | - | 0 |
| 47 | 68 | F | Invasive carcinoma of no special type | 1--2 | T1N0M0 | IA | Malignant | - | +，15% | 0 |
| 48 | 51 | F | Invasive carcinoma of no special type | 3 | T2N0M0 | IIA | Malignant | - | - | 0 |
| 49 | 48 | F | Invasive carcinoma of no special type | 3 | T2N0M0 | IIA | Malignant | - | - | 0 |
| 50 | 50 | F | Invasive carcinoma of no special type | 3 | T4N0M0 | IIIB | Malignant | - | - | 0 |
| 51 | 55 | F | Invasive carcinoma of no special type | 3 | T3N0M0 | IIB | Malignant | - | - | 0 |
| 52 | 60 | F | Invasive carcinoma of no special type | 3 | T2N1M0 | IIB | Malignant | - | - | 0 |
| 53 | 33 | F | Invasive carcinoma of no special type with degeneration and necrosis | 3 | T3N3M0 | IIIC | Malignant | - | - | 0 |
| 54 | 34 | F | Invasive carcinoma of no special type with sparse necrosis | 3 | T2N0M0 | IIA | Malignant | - | - | 0 |
| 55 | 40 | F | Invasive carcinoma of no special type | 3 | T2N2M0 | IIIA | Malignant | - | - | 0 |
| 56 | 52 | F | Invasive carcinoma of no special type | 3 | T3N3M0 | IIIC | Malignant | - | weakly+，10% | 0 |
| 57 | 39 | F | Invasive carcinoma of no special type(sparse necrosis) | 2--3 | T4N2M0 | IIIB | Malignant | - | - | 0 |
| 58 | 60 | F | Invasive carcinoma of no special type | 3 | T2N0M0 | IIA | Malignant | - | - | 0 |
| 59 | 52 | F | Invasive carcinoma of no special type with necrosis | 3 | T3N0M0 | IIB | Malignant | - | - | 0 |
| 60 | 66 | F | Invasive carcinoma of no special type | 3 | T2N0M0 | IIA | Malignant | - | - | 0 |
| 61 | 86 | F | Invasive carcinoma of no special type | 3 | T3N0M0 | IIB | Malignant | - | - | 0 |
| 62 | 42 | F | Invasive carcinoma of no special type | 3 | T3N0M0 | IIB | Malignant | - | - | 0 |
| 63 | 46 | F | Invasive carcinoma of no special type | 3 | T3N1M0 | IIIA | Malignant | - | - | 0 |
| 64 | 38 | F | Invasive carcinoma of no special type | 3 | T2N0M0 | IIA | Malignant | - | - | 0 |
| 65 | 45 | F | Invasive carcinoma of no special type | 3 | T2N0M0 | IIA | Malignant | - | +，60% | 0 |
| 66 | 44 | F | Invasive carcinoma of no special type | 3 | T3N0M0 | IIB | Malignant | - | - | 0 |
| 67 | 48 | F | Invasive carcinoma of no special type | 3 | T3N0M0 | IIB | Malignant | - | - | 0 |
| 68 | 57 | F | Invasive carcinoma of no special type | 3 | T2N1M0 | IIB | Malignant | - | weakly+，15% | 0 |
| 69 | 38 | F | Invasive carcinoma of no special type | 3 | T3N0M0 | IIB | Malignant | - | - | weakly, 1+ |
| 70 | 55 | F | Invasive carcinoma of no special type | 3 | T2N0M0 | IIA | Malignant | - | - | 0 |
| 71 | 49 | F | Invasive carcinoma of no special type | 3 | T3N2M0 | IIIA | Malignant | - | - | 0 |
| 72 | 69 | F | Invasive carcinoma of no special type | 3 | T2N0M0 | IIA | Malignant | - | - | 0 |
| 73 | 38 | F | Invasive carcinoma of no special type | 3 | T4N0M0 | IIIB | Malignant | - | weakly+，5% | 0 |
| 74 | 58 | F | Invasive carcinoma of no special type | 3 | T2N0M0 | IIA | Malignant | - | - | 0 |
| 75 | 63 | F | Invasive carcinoma of no special type | 3 | T2N1M0 | IIB | Malignant | - | - | 0 |
| 76 | 57 | F | Mixed invasive ductal and lobular carcinoma | - | T2N0M0 | IIA | Malignant | - | +，30% | 0 |
| 77 | 62 | F | Invasive carcinoma of no special type with sparse necrosis | 3 | T4N0M0 | IIIB | Malignant | - | - | 0 |
| 78 | 51 | F | Invasive carcinoma of no special type | 3 | T2N0M0 | IIA | Malignant | - | - | 0 |
| 79 | 34 | F | Invasive carcinoma of no special type | 3 | T2N0M0 | IIA | Malignant | - | - | 0 |
| 80 | 72 | F | Invasive carcinoma of no special type | 3 | T4N0M0 | IIIB | Malignant | - | - | 0 |
| 81 | 65 | F | Invasive carcinoma of no special type with sparse necrosis | 3 | T3N0M0 | IIB | Malignant | - | - | 0 |
| 82 | 60 | F | Invasive carcinoma of no special type | 3 | T2N0M0 | IIA | Malignant | - | - | 0 |
| 83 | 50 | F | Invasive carcinoma of no special type | 3 | T2N0M0 | IIA | Malignant | - | - | 0 |
| 84 | 42 | F | Invasive carcinoma of no special type | 3 | T2N0M0 | IIA | Malignant | - | - | 0 |
| 85 | 44 | F | Invasive carcinoma of no special type with sparse necrosis | 3 | T2N0M0 | IIA | Malignant | - | - | 0 |
| 86 | 40 | F | Invasive carcinoma of no special type | 3 | T2N1M0 | IIB | Malignant | - | - | 0 |
| 87 | 40 | F | Invasive carcinoma of no special type | 3 | T3N2M0 | IIIA | Malignant | - | - | 0 |
| 88 | 49 | F | Invasive carcinoma of no special type with sparse necrosis | 3 | T2N1M0 | IIB | Malignant | - | - | 0 |
| 89 | 80 | F | Invasive ductal carcinoma with mucinous adenocarcinoma differentiation (fibrous and blood vessal tissue) | * | T3N1M0 | IIIA | Malignant | - | - | 0 |
| 90 | 33 | F | Invasive carcinoma of no special type with metaplastic carcinoma (spindle cell type) | 3 | T2N0M0 | IIA | Malignant | - | - | 0 |
| 91 | 50 | F | Invasive carcinoma of no special type with neuroendocrine differentiation | - | T2N0M0 | IIA | Malignant | - | - | 0 |
| 92 | 49 | F | Invasive carcinoma of no special type with sparse necrosis | 3 | T2N0M0 | IIA | Malignant | - | - | 0 |
| 93 | 49 | F | Mixed invasive ductal and lobular carcinoma | 2 | T3N0M0 | IIB | Malignant | - | - | 0 |
| 94 | 54 | F | Invasive carcinoma of no special type | 2 | T1N0M0 | IA | Malignant | - | - | 0 |
| 95 | 58 | F | Invasive lobular carcinoma | 2 | T1N0M0 | IA | Malignant | - | +，10% | 0 |
| 96 | 78 | F | Mixed lobular and ductal carcinoma | 3 | T1N0M0 | IA | Malignant | - | - | 0 |
| 97 | 54 | F | Medullary carcinoma with sparse necrosis | 3 | T2N0M0 | IIA | Malignant | - | weakly+，5% | 0 |
| 98 | 47 | F | Medullary carcinoma | 3 | T2N2M0 | IIA | Malignant | - | - | 0 |
| 99 | 64 | F | Medullary carcinoma | - | T2N0M0 | IIA | Malignant | - | - | 0 |
| 100 | 51 | F | Medullary carcinoma with sparse necrosis | 3 | T2N0M0 | IIA | Malignant | - | - | 0 |
| 101 | 52 | F | Medullary carcinoma | - | T2N0M0 | IIA | Malignant | - | - | 0 |
| 102 | 52 | F | Medullary carcinoma | - | T2N0M0 | IIA | Malignant | - | - | 0 |
| 103 | 48 | F | Medullary carcinoma | - | T1N0M0 | IIA | Malignant | - | - | 0 |
| 104 | 42 | F | Carcinoid | - | T2N0M0 | IIA | Malignant | - | - | 0 |
| 105 | 42 | F | Carcinoid | - | T2N0M0 | IIA | Malignant | - | - | 0 |
| 106 | 41 | F | Neuroendocrine carcinoma | - | T2N0M0 | IIA | Malignant | - | weakly+，5% | 0 |
| 107 | 40 | F | Aggressive adenocarcinoma | - | T2N0M0 | IIA | Malignant | - | - | 0 |
| 108 | 37 | F | Invasive micropapillary carcinoma | - | T2N1M0 | IIB | Malignant | - | - | 0 |
| 109 | 60 | F | Invasive micropapillary carcinoma | - | T2N0M0 | IIA | Malignant | - | - | 0 |
| 110 | 50 | F | Mucinous adenocarcinoma | - | T4N0M0 | IIIB | Malignant | - | +，5% | 1+ |
| 111 | 28 | F | Invasive carcinoma of no special type (sparse) | 2 | T2N1M0 | IIB | Malignant | ++，80% | +,5% | weakly, 1+ |
| 112 | 65 | F | Invasive carcinoma of no special type | 2 | T4N0M0 | IIIB | Malignant | ++，90% | +，30% | 0 |
| 113 | 45 | F | Invasive carcinoma of no special type | 2 | T3N0M0 | IIB | Malignant | ++，80% | +++,100% | 1+ |
| 114 | 45 | F | Mixed invasive ductal and lobular carcinoma | - | T4N2M0 | IIIB | Malignant | ++，90% | +++,100% | 0 |
| 115 | 79 | F | Medullary carcinoma | - | T3N0M0 | IIB | Malignant | - | - | 3+ |
| 116 | 36 | F | Invasive carcinoma of no special type with necrosis | 2 | T2N0M0 | IIA | Malignant | - | +,70% | 3+ |
| 117 | 39 | F | Adjacent normal breast tissue | - | - | - | NAT | - | - | - |
| 118 | 43 | F | Adjacent normal breast tissue | - | - | - | NAT | - | - | - |
| 119 | 46 | F | Adjacent normal breast tissue | - | - | - | NAT | - | - | - |
| 120 | 53 | F | Adjacent normal breast tissue | - | - | - | NAT | - | - | - |

**Table S2 Sequences of shRNAs used in this study**

| Definition | Target Sequences (5’-3’) |
| --- | --- |
| shNC | GCTTCGCGCCGTAGTCTTA |
| shZEB1-1 | GGATCAACCACCAATGGTTCC |
| shZEB1-2 | GCAACAGGGAGAATTATTAGA |
| shZEB1-3 | GCATACACCTACTCAACTACG |
| shUSP10-1 | GCTACAGTATTTCAAGCACAC |
| shUSP10-2 | CCATAAACCAGTGTCGTTGCA |
| shUSP10-3 | GGCTAATGAATGAGTTCACTA |

**Table S3 Primers for RT-qPCR analysis**

| **Genes** | **Primer sequences (5’-3’)** |
| --- | --- |
| *TSPYL5* | Forward: TAATACGACTCACTATAGGG |
|  | Reverse: CTGGAATAGCTCAGAGGC |
| *GAPDH* | Forward: ACGGATTTGGTCGTATTGGG |
|  | Reverse: CGCTCCTGGAAGATGGTGAT |
| *ZEB1* | Forward: GATGATGAATGCGAGTCAGATGC |
|  | Reverse: ACAGCAGTGTCTTGTTGTTGT |
| *FN1* | Forward: CGGTGGCTGTCAGTCAAAG |
|  | Reverse: AAACCTCGGCTTCCTCCATAA |
| *CDH1* | Forward: CGAGAGCTACACGTTCACGG |
|  | Reverse: GGGTGTCGAGGGAAAAATAGG |
| *CDH2* | Forward: TCAGGCGTCTGTAGAGGCTT |
|  | Reverse: ATGCACATCCTTCGATAAGACTG |
| *TGFB1* | Forward: GGCCAGATCCTGTCCAAGC |
|  | Reverse: GTGGGTTTCCACCATTAGCAC |
| *CCN2* | Forward: CAGCATGGACGTTCGTCTG |
|  | Reverse: AACCACGGTTTGGTCCTTGG |
| *ECM1* | Forward: GCTTCACGGCTACAGGACAG |
|  | Reverse: GAGGCTTCGGGATAGGGGT |
| *IGFBP3* | Forward: AGAGCACAGATACCCAGAACT |
|  | Reverse: GGTGATTCAGTGTGTCTTCCATT |
| *GPC1* | Forward: TGAAGCTGGTCTACTGTGCTC |
|  | Reverse: CCCAGAACTTGTCGGTGATGA |

**Table S4 List of primary antibodies used in this study**

| Antibody name | Catalogue number | Specificity | Manufacturer | RRID |
| --- | --- | --- | --- | --- |
| Anti-TSPYL5 | sc-98186 | Rabbit Polyclonal | SantaCruz Biotechnology | AB_2211377 |
| Anti-PTEN | ab267787 | Rabbit Monoclonal | Abcam | AB_2924403 |
| Anti-USP10 | 19374-1-AP | Rabbit Polyclonal | Proteintech | AB_10858617 |
| Anti-ZEB1 | 21544-1-AP | Rabbit Polyclonal | Proteintech | AB_10734325 |
| Anti-FN1 | ET1702-25 | Rabbit Monoclonal | HUABIO | AB_3070288 |
| Anti-E-cadherin | 340341 | Rabbit Polyclonal | ZENBIO | / |
| Anti-N-cadherin | 380671 | Rabbit Polyclonal | ZENBIO | / |
| Anti-AKT | 10176-2-AP | Rabbit Polyclonal | Proteintech | AB_2224574 |
| Anti-Phospho-AKT (Ser473) | 66444-1-Ig | Mouse Monoclonal | Proteintech | AB_2782958 |
| Anti-mTOR | 66888-1-Ig | Mouse Monoclonal | Proteintech | AB_2882219 |
| Anti-Phospho-mTOR (Ser2448) | 67778-1-Ig | Mouse Monoclonal | Proteintech | AB_2889842 |
| Anti-PI3K p85 | R22768 | Rabbit Polyclonal | ZENBIO | / |
| Anti-Phospho-PI3K p85/p55 (Tyr467/Tyr199) | 341468 | Rabbit Polyclonal | ZENBIO | / |
| Anti-IL6 | R1412-2 | Rabbit Polyclonal | HUABIO | AB_3073311 |
| Anti-STAT3 | ET1607-38 | Rabbit Monoclonal | HUABIO | AB_3069762 |
| Anti-Phospho-STAT3  (S727) | ET1607-39 | Rabbit Monoclonal | HUABIO | AB_3069763 |
| Anti-SMAD2 | 12570-1-AP | Rabbit Polyclonal | Proteintech | AB_2193037 |
| Anti-SMAD4 | 10231-1-AP | Rabbit Polyclonal | Proteintech | AB_2193323 |
| Anti-GSK3β | 22104-1-AP | Rabbit Polyclonal | Proteintech | AB_2878997 |
| Anti-β-catenin | 51067-2-AP | Rabbit Polyclonal | Proteintech | AB_2086128 |
| Anti-GAPDH | 10494-1-AP | Rabbit Polyclonal | Proteintech | AB_2263076 |
| Anti-GAPDH | 60004-1-Ig | Mouse Monoclonal | Proteintech | AB_2107436 |
| Normal Rabbit IgG | 2729 | Rabbit IgG | Cell Signaling Technology | AB_1031062 |
| Flag Tag Rabbit pAb | 20543-1-AP | Rabbit Polyclonal | Proteintech | AB_11232216 |
| Flag Tag Mouse mAb | M1403-2 | Mouse Monoclonal | HUABIO | AB_3073075 |
| Myc Tag Rabbit pAb | 16286-1-AP | Rabbit Polyclonal | Proteintech | AB_11182162 |
| Ubiquitin | 10201-2-AP | Rabbit Polyclonal | Proteintech | AB_671515 |

**References**

[1] Dongre A, Weinberg RA. New insights into the mechanisms of epithelial-mesenchymal transition and implications for cancer. Nat Rev Mol Cell Biol. 2019;;20(2):69-84. doi: 10.1038/s41580-018-0080-4. PMID: 30459476.
