# Supplementary material for: TSPYL5 Promotes Triple‐Negative Breast Cancer Metastasis by Antagonizing USP10‐Mediated PTEN Stabilization to Unleash a ZEB1‐Dependent EMT Program
Source: Adv Sci (Weinh). 2026 Jun 4:e20273. Online ahead of print. doi: 10.1002/advs.202520273 (PMC13336384; doi:10.1002/advs.202520273)

Figure 6E

FN1

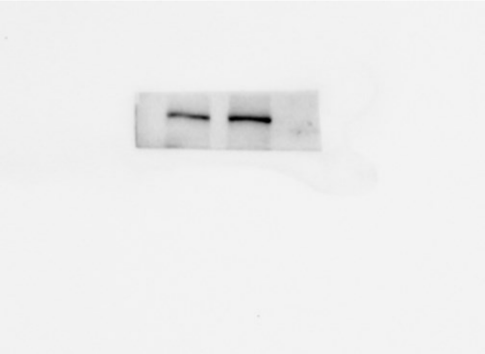

N-cadherin

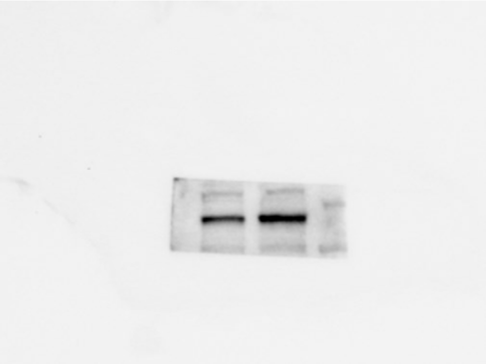

E-cadherin

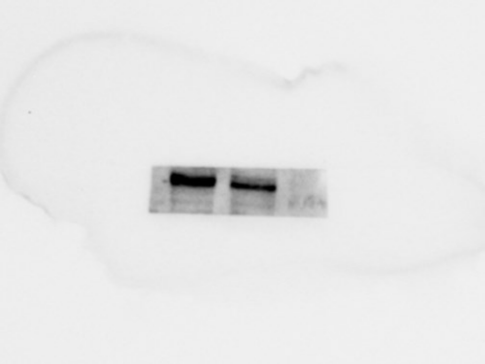

TSPYL5

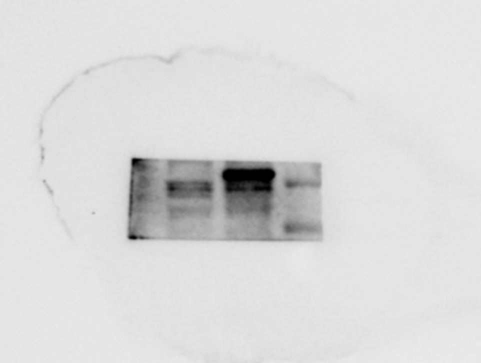

ZEB1

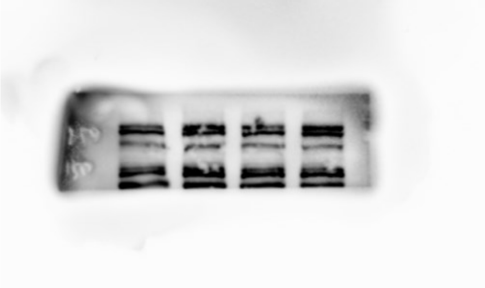

GAPDH

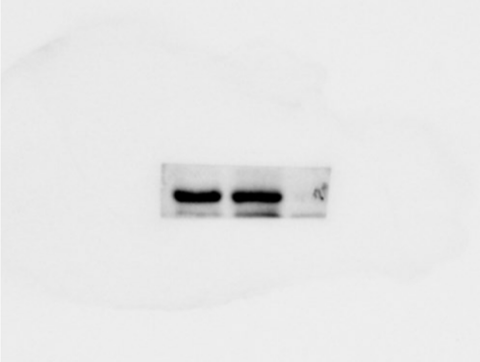

Figure 6F

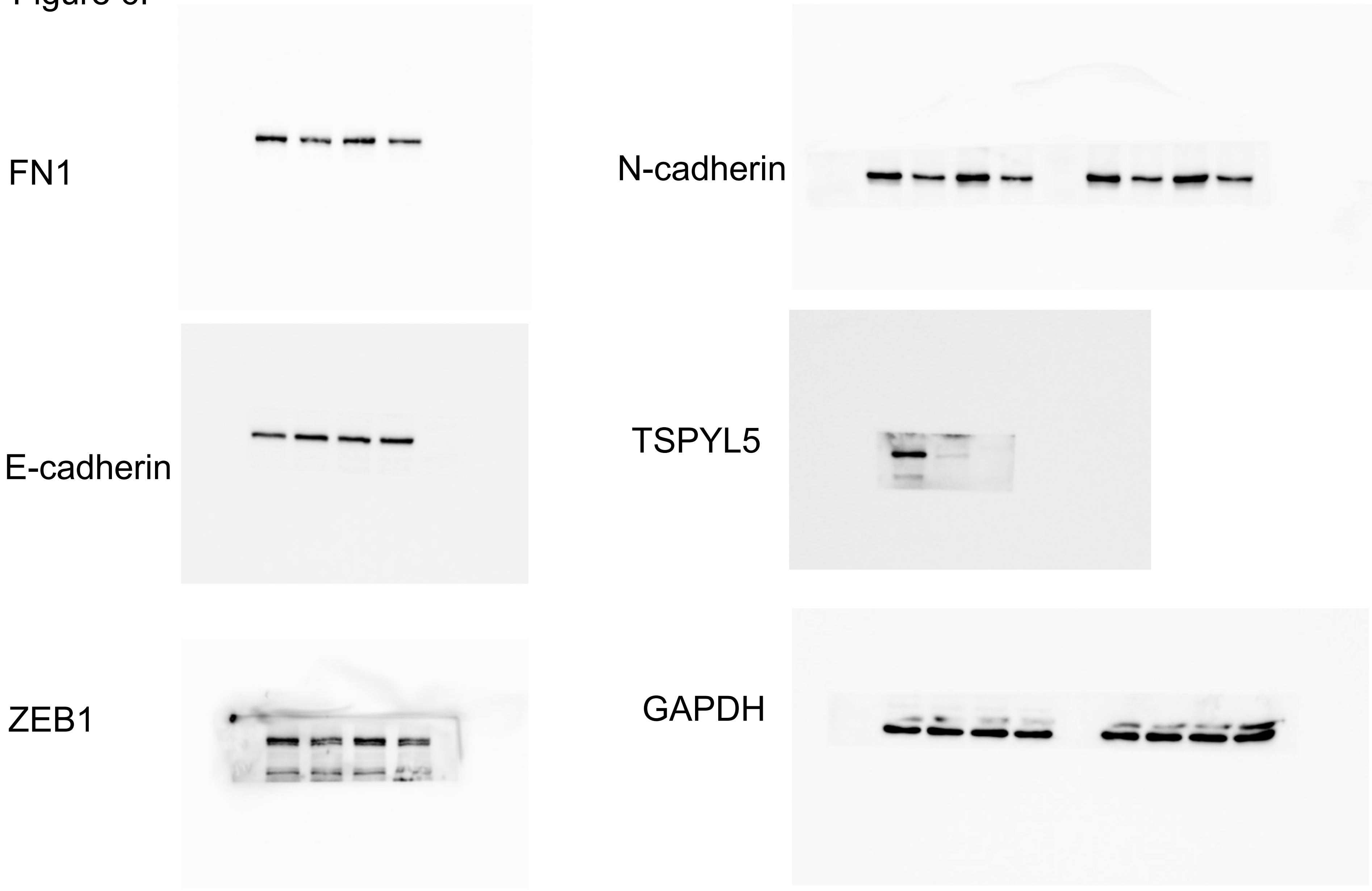

Figure 6K

ZEB1

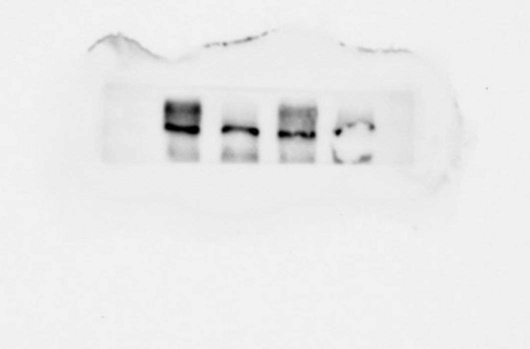

GAPDH

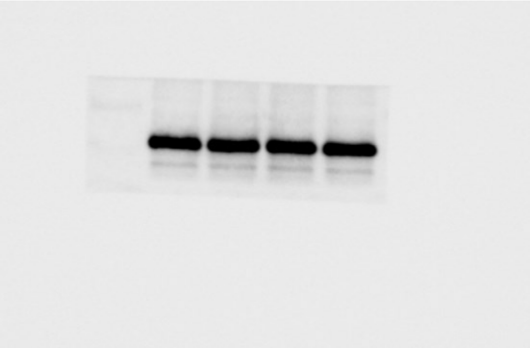

Figure 6L

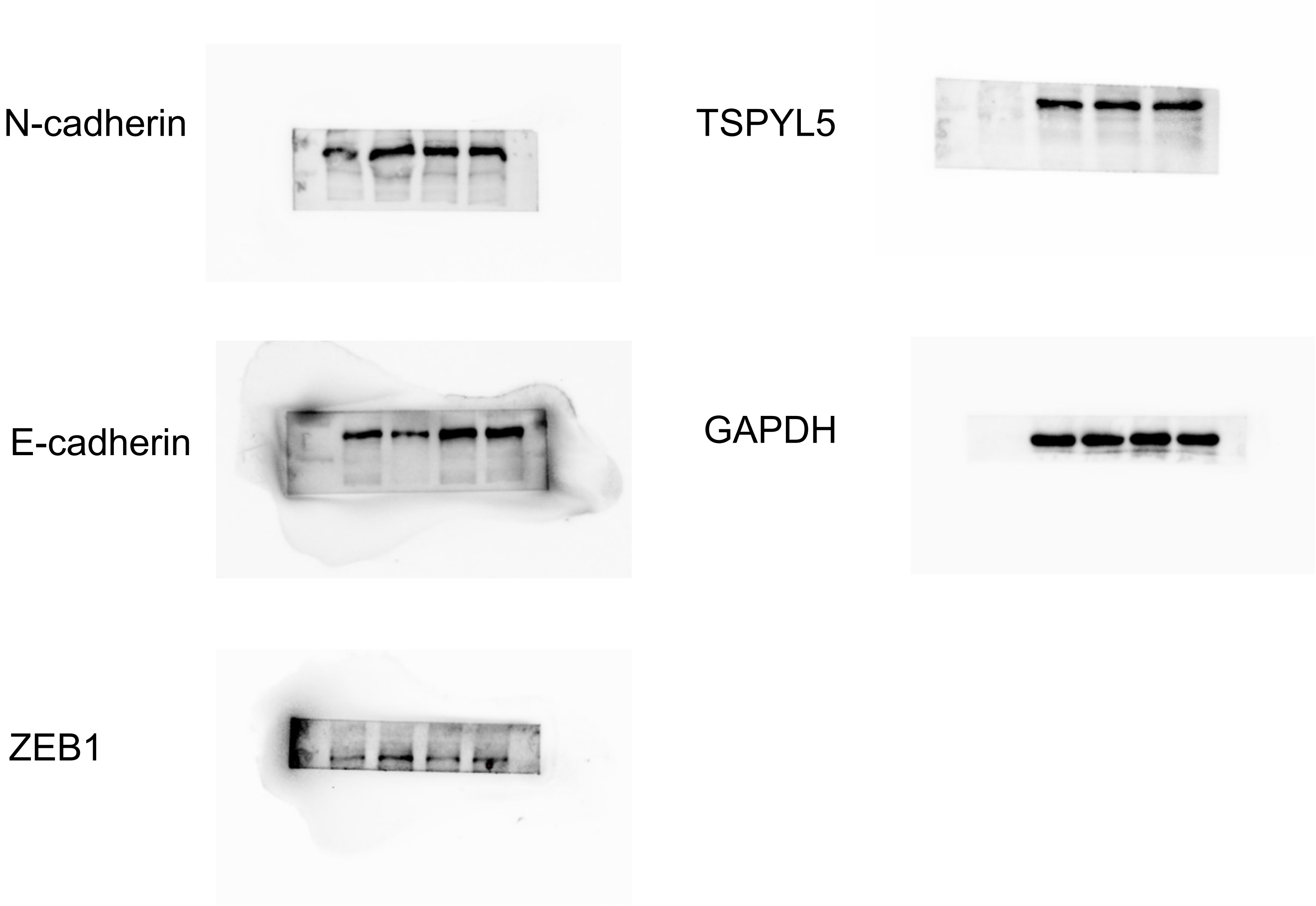

Figure 6M

N-cadherin

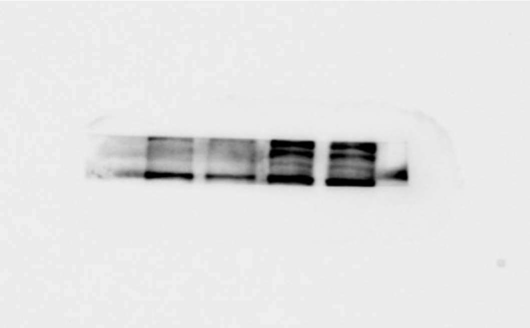

TSPYL5

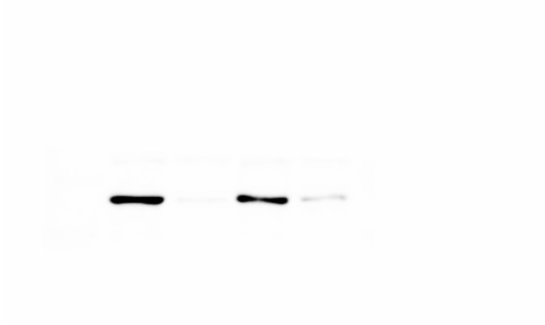

E-cadherin

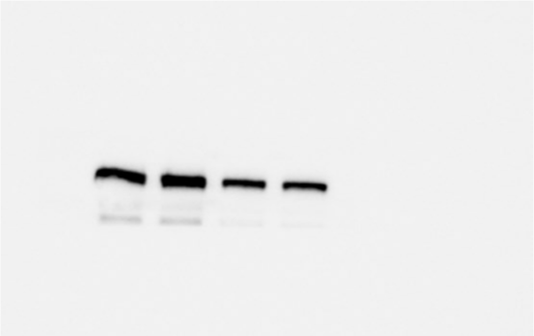

GAPDH

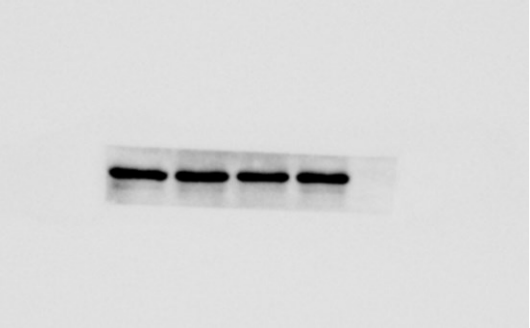

ZEB1

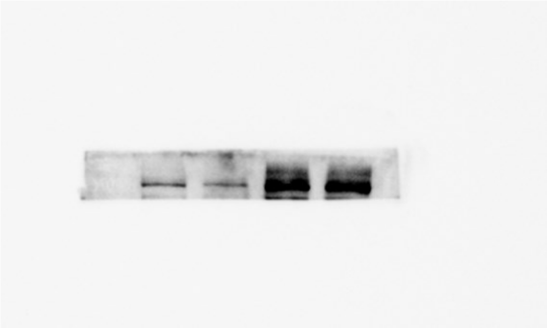

Figure 7C MDA-MB-231

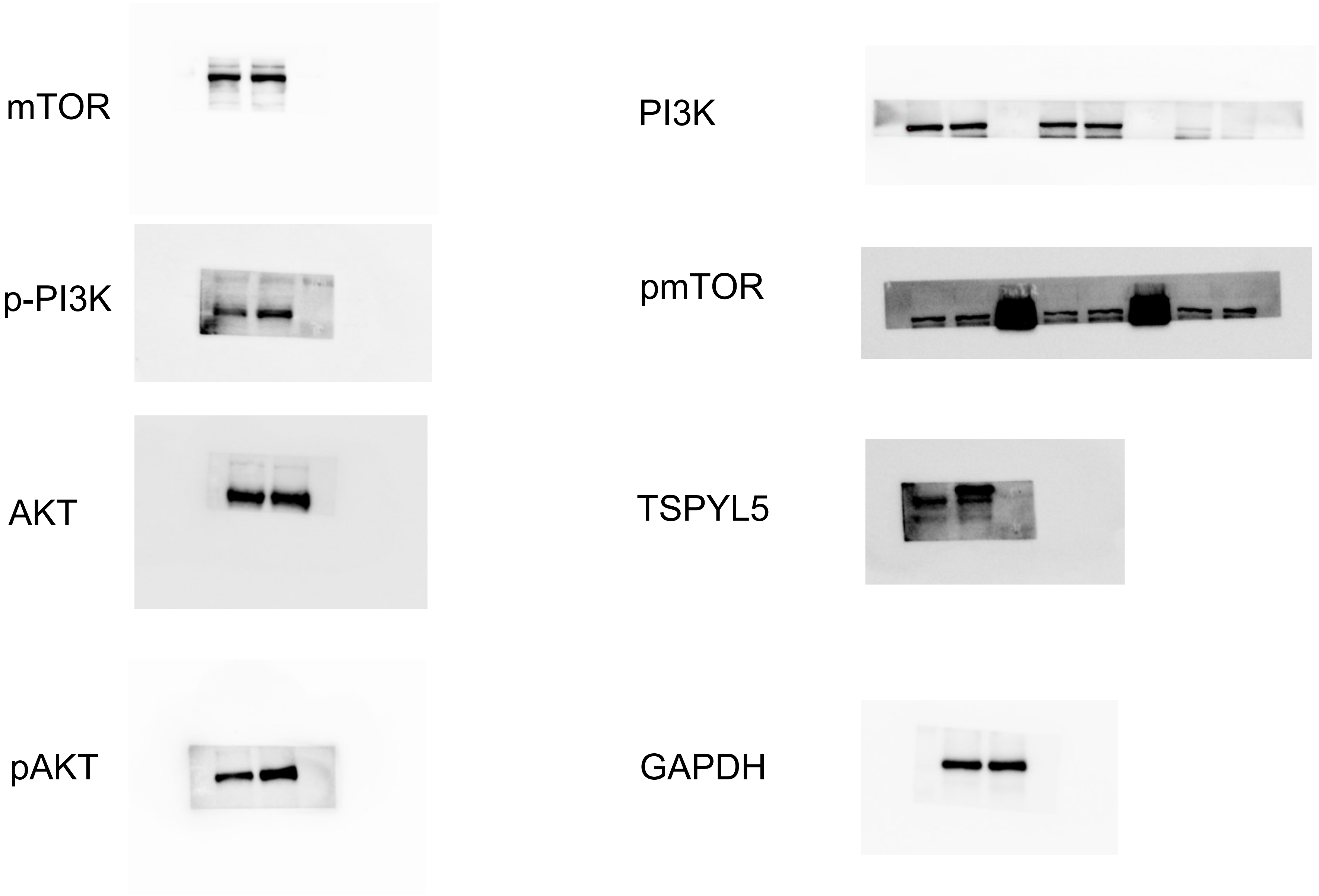

Figure 7C HCC38

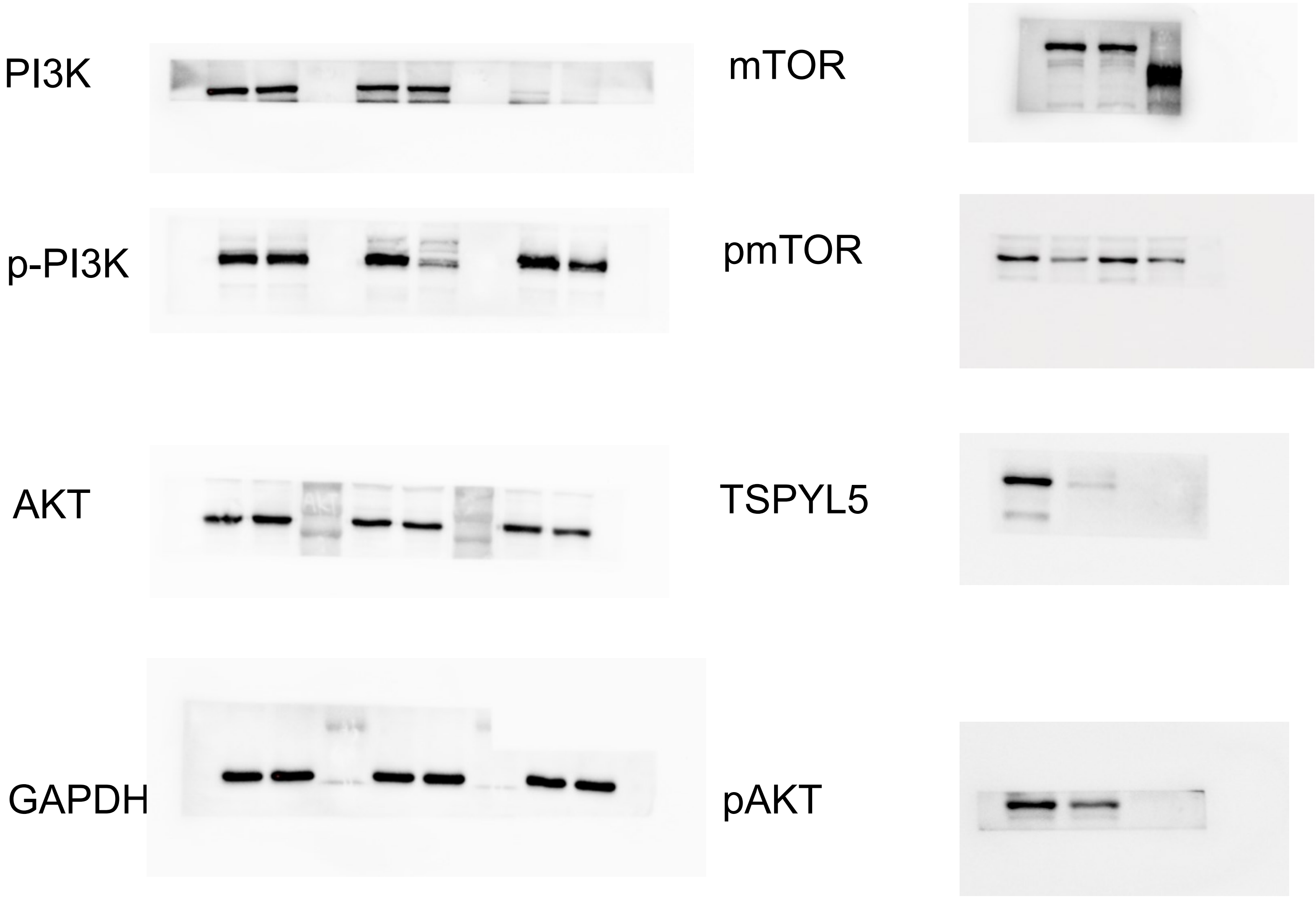

Figure 7E

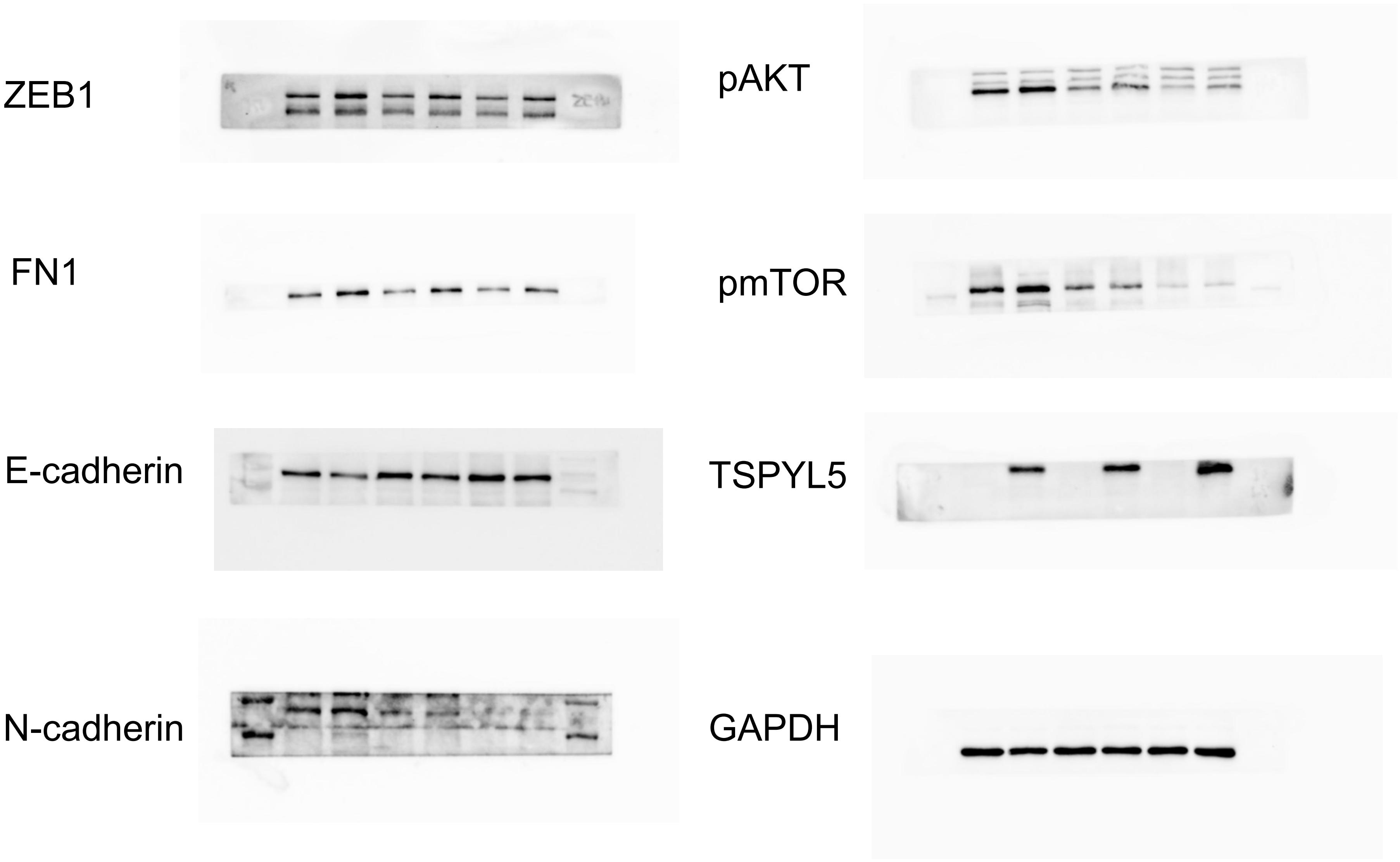

Figure 7F

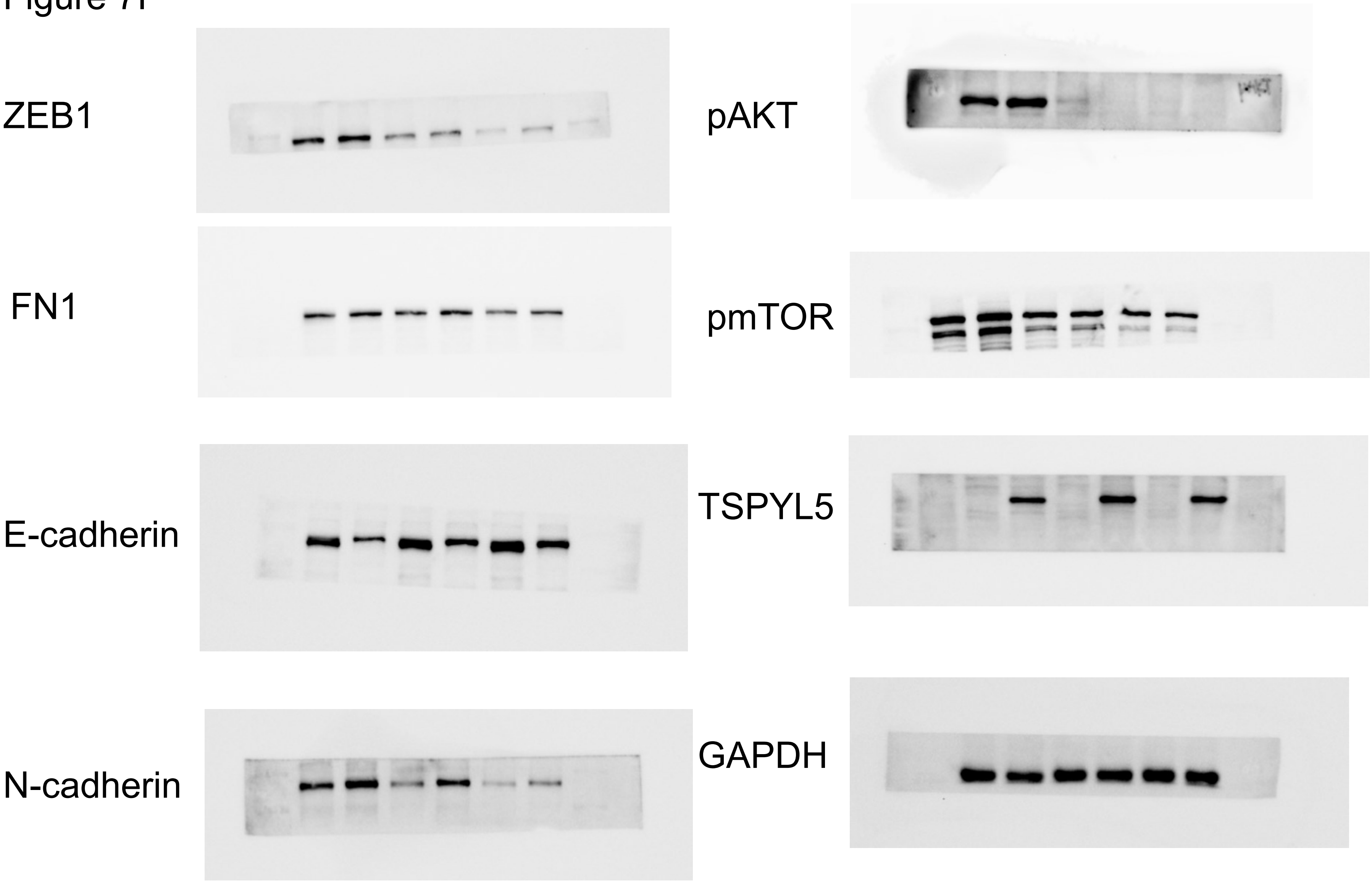

Figure 7G

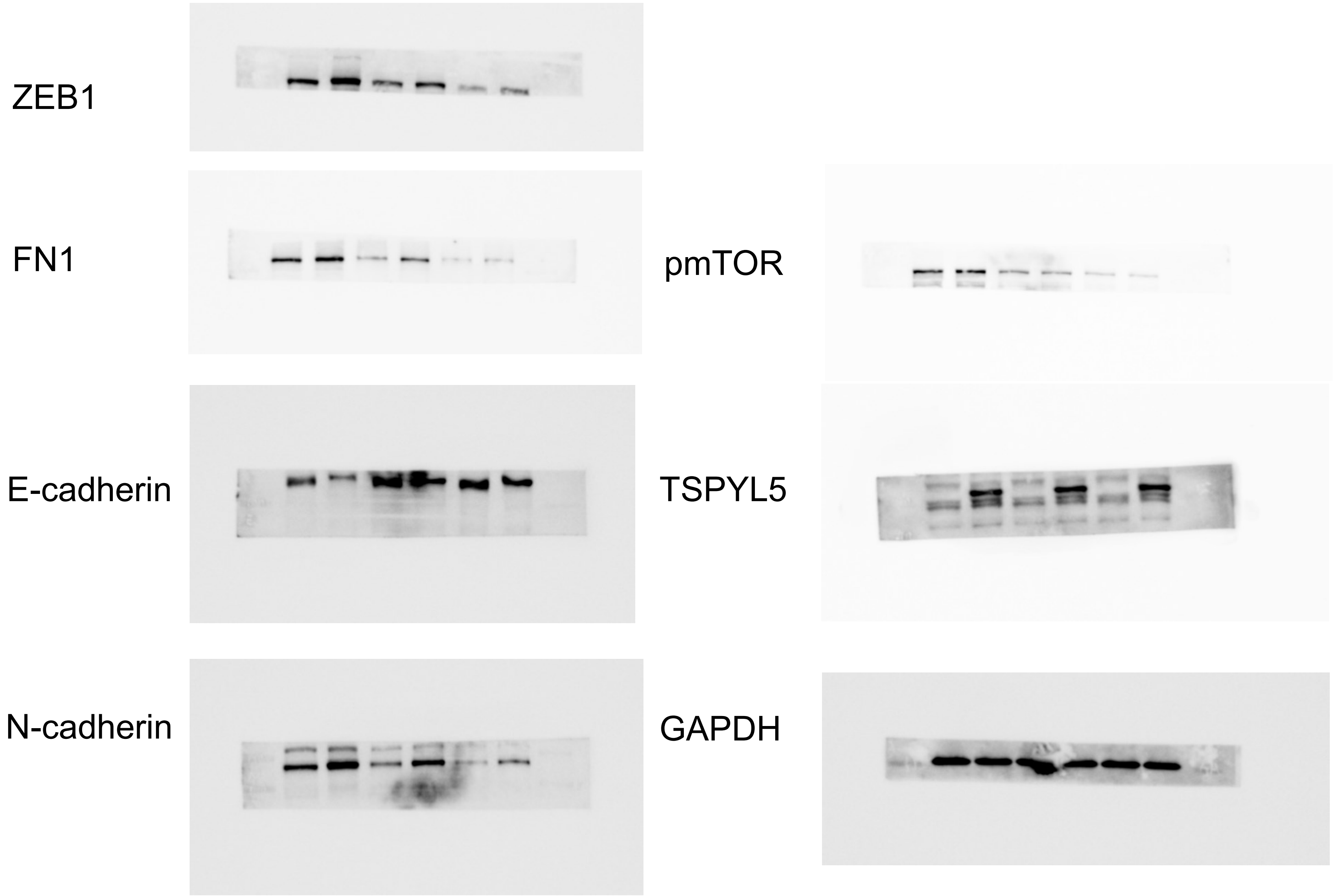

Figure 8A

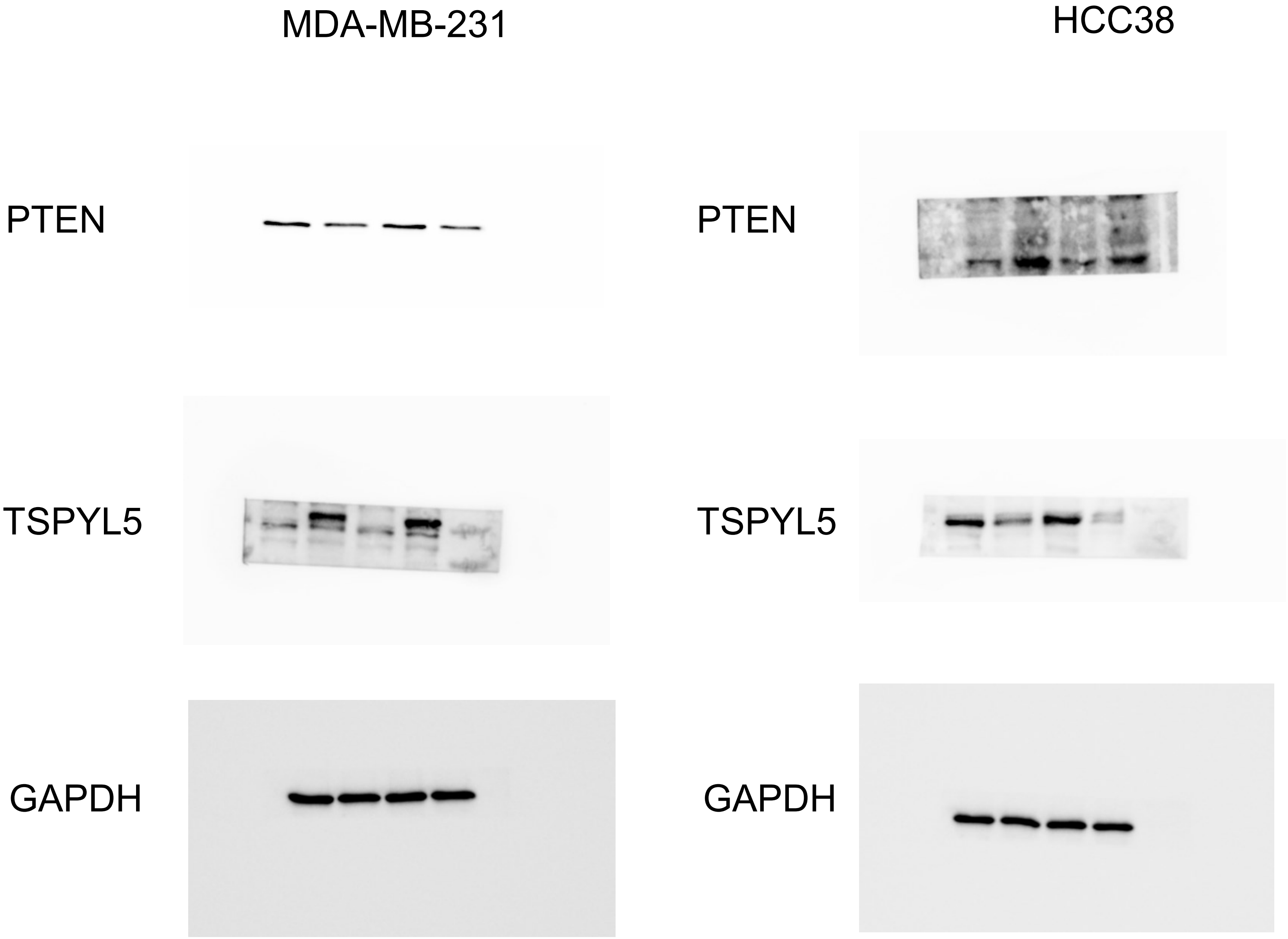

Figure 8B

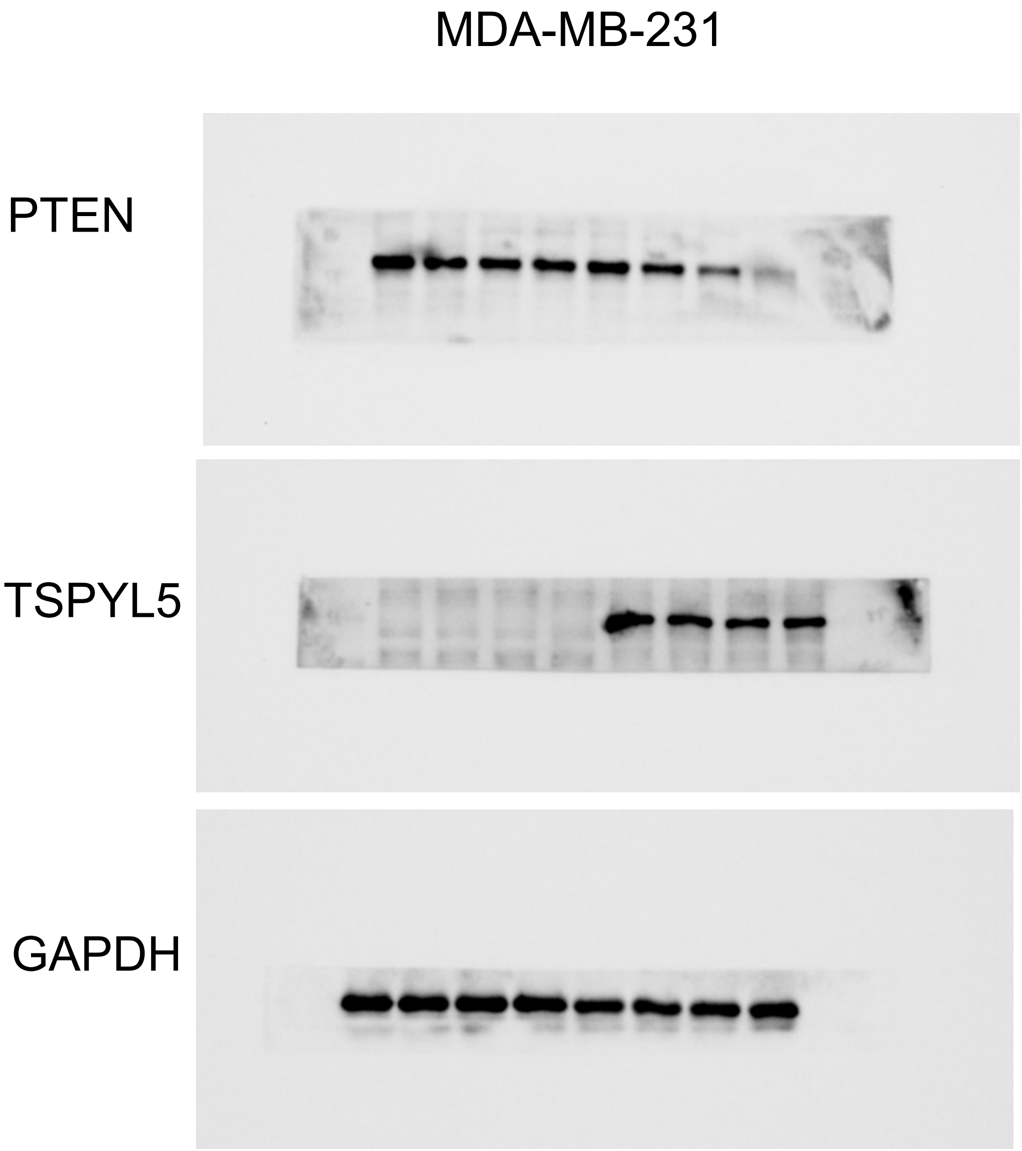

Figure 8C

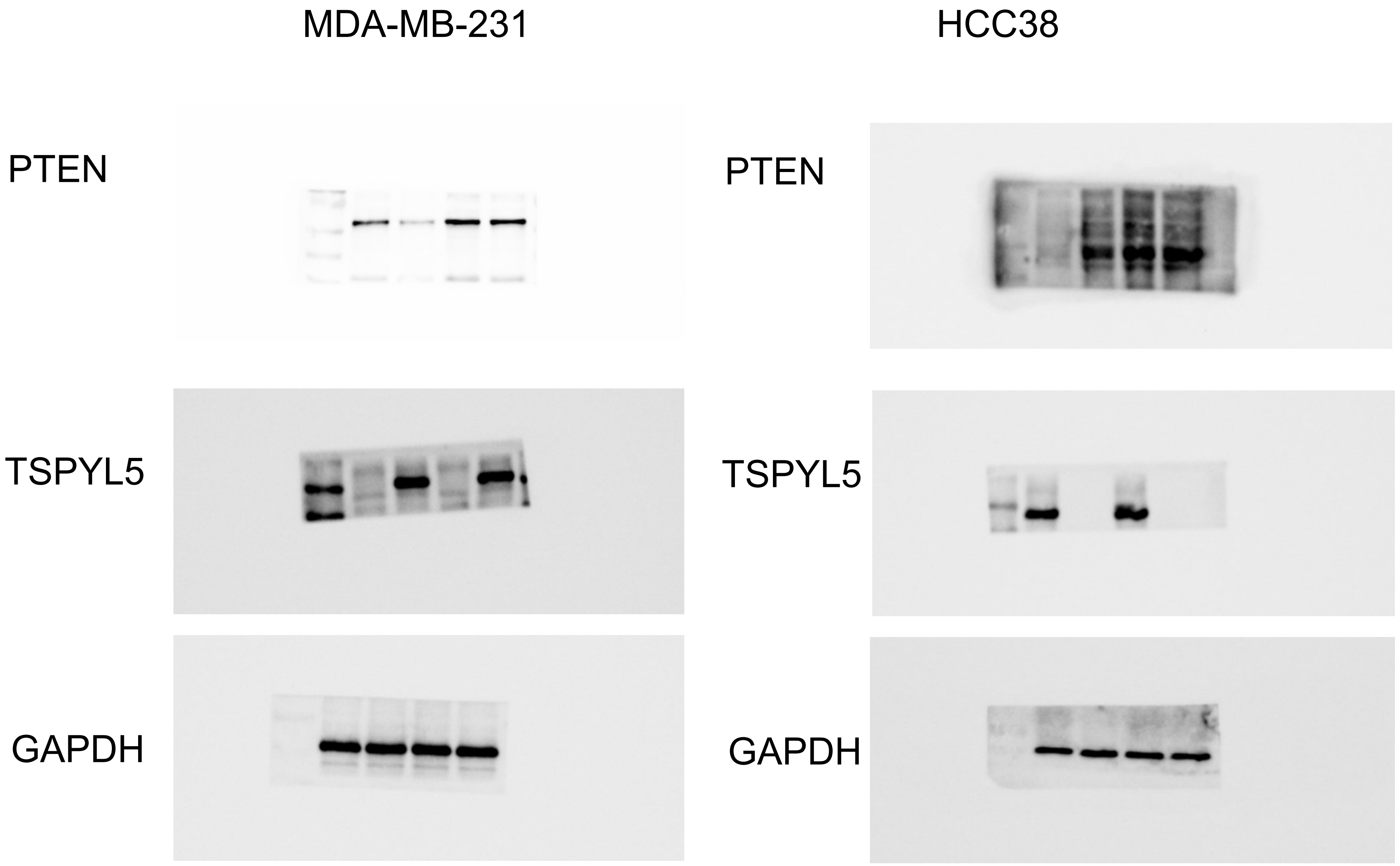

Figure 8D MDA-MB-231

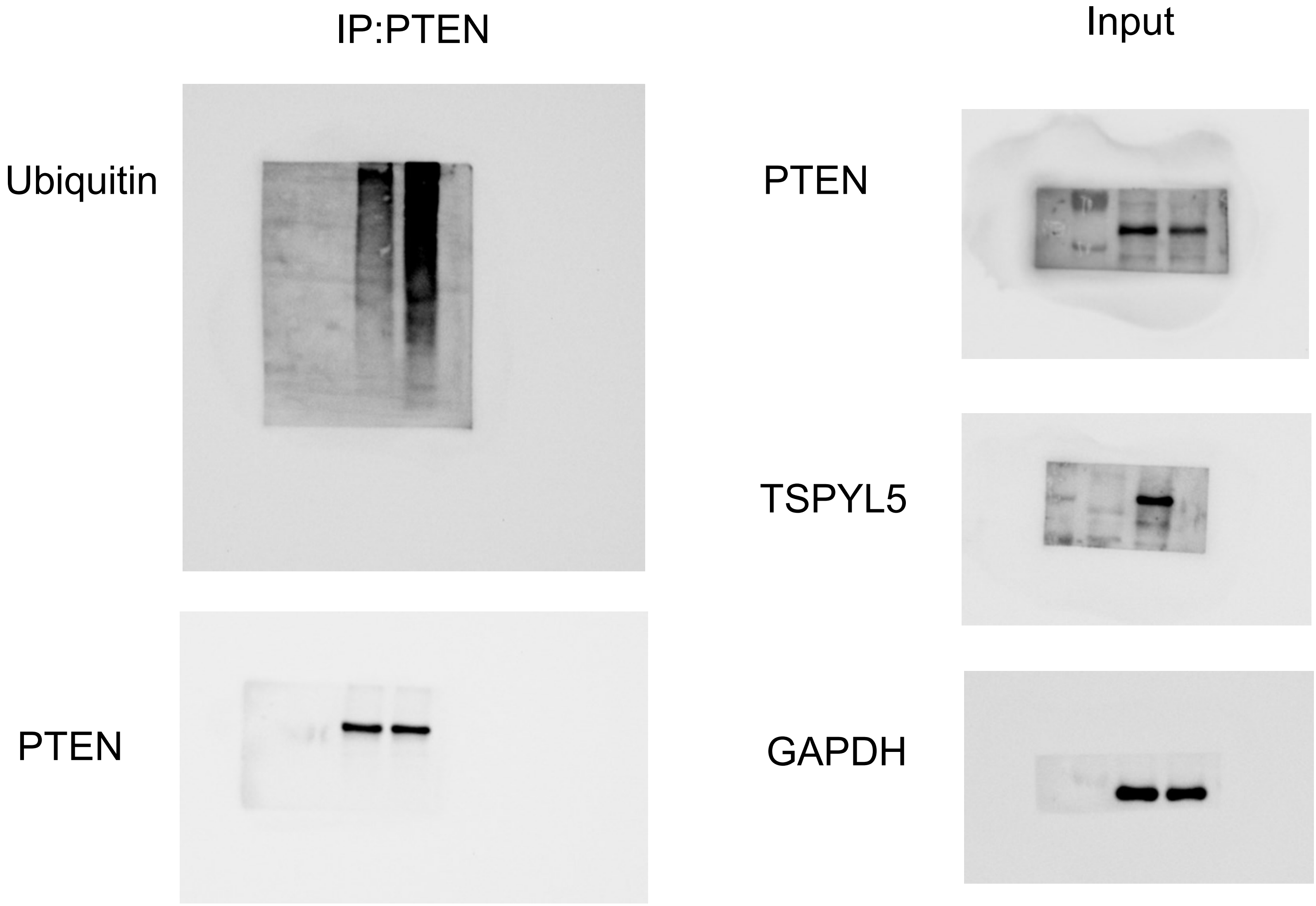

Figure 8E HCC38

IP:PTEN

Ubiquitin

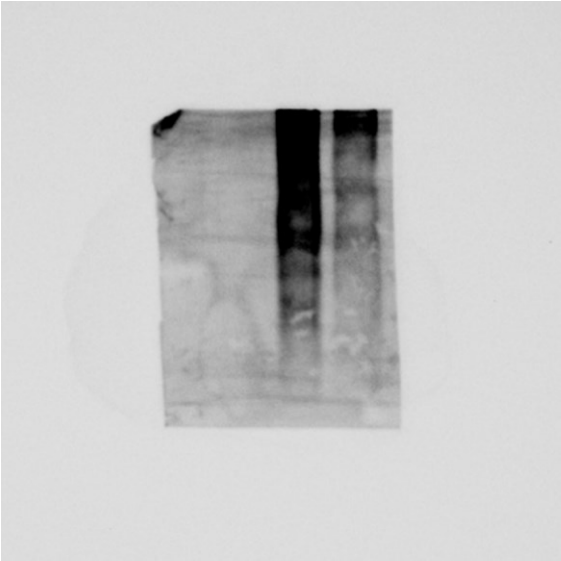

PTEN

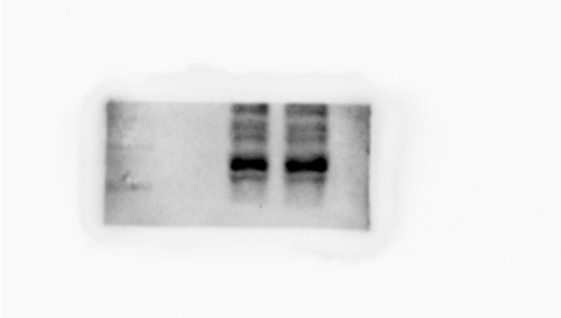

Input

PTEN

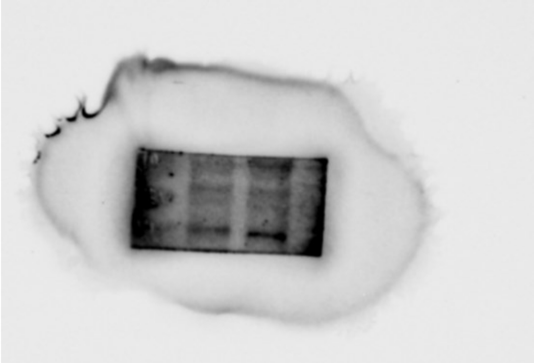

TSPYL5

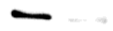

GAPDH

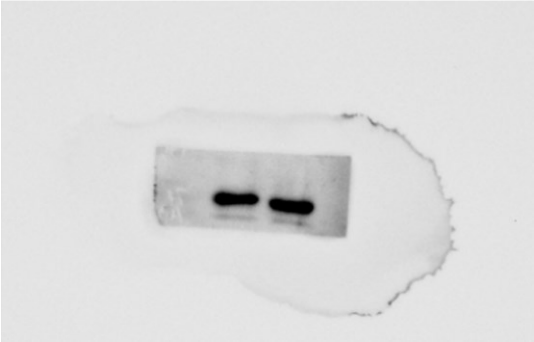

Figure 8F HCC38

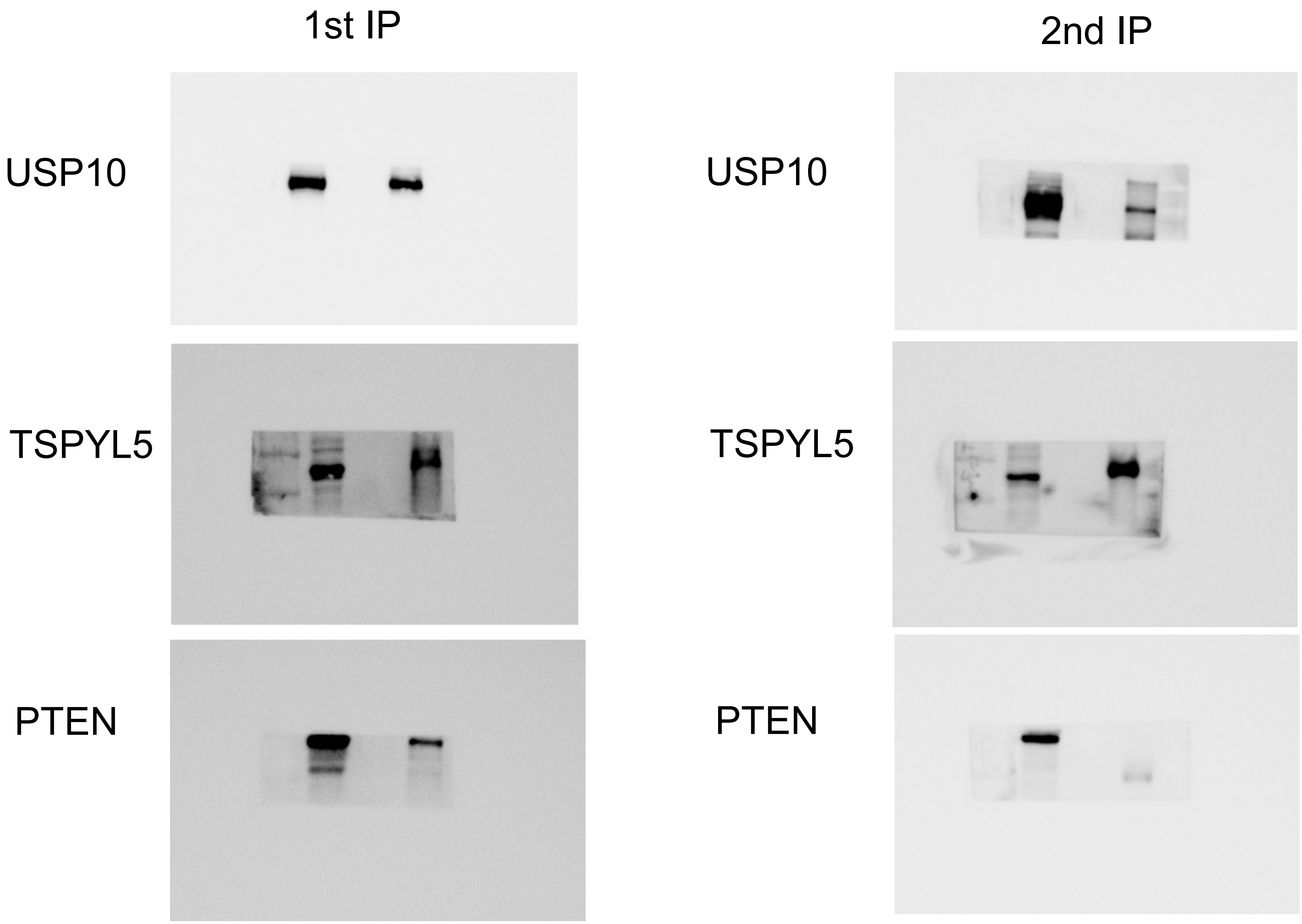

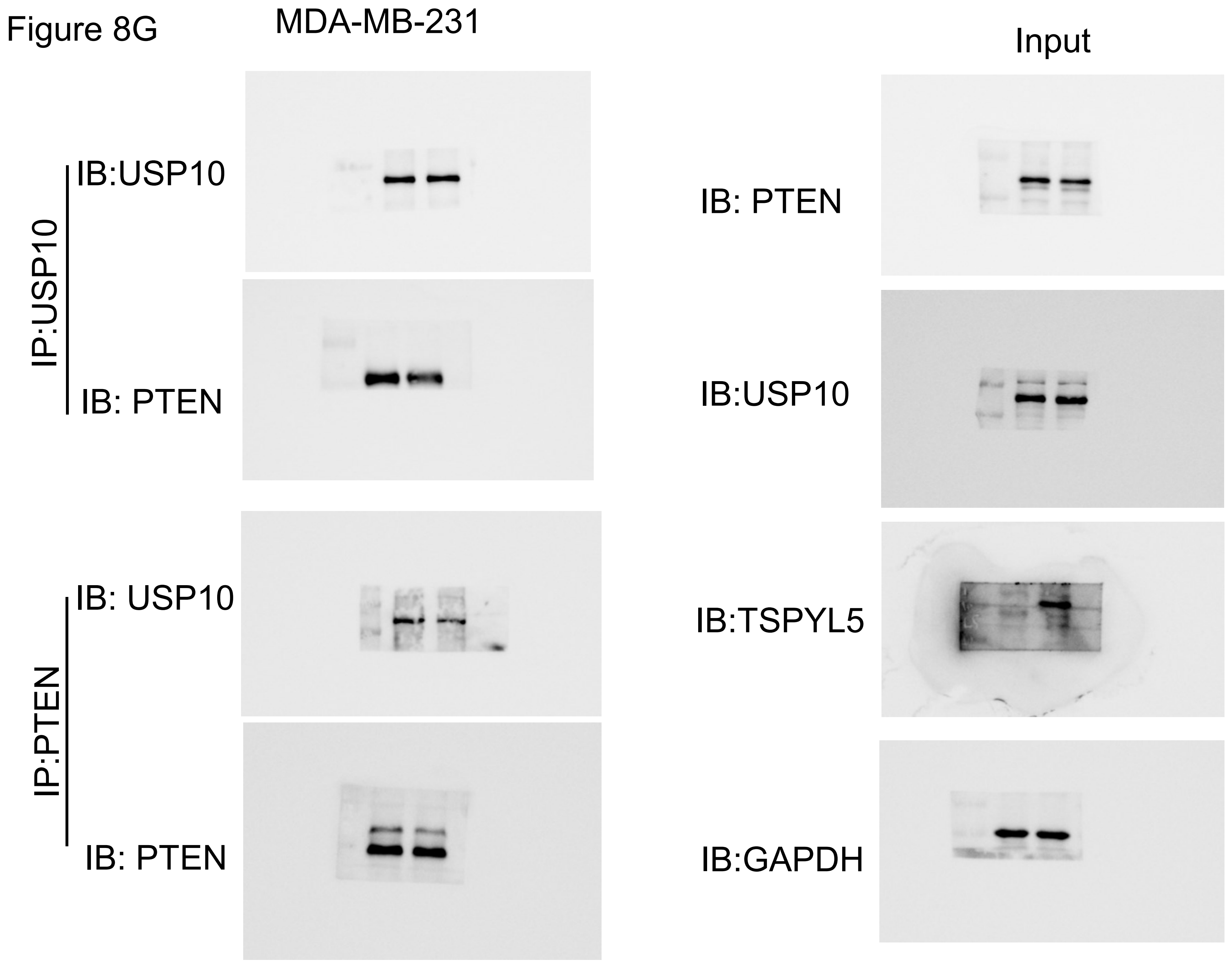
Figure 8G
MDA-MB-231
Input
IB:USP10
IP:USP10
IB: PTEN
IB: USP10
IP:PTEN
IB: PTEN
IB: PTEN
IB:USP10
IB:TSPYL5
IB:GAPDH

Figure 8H

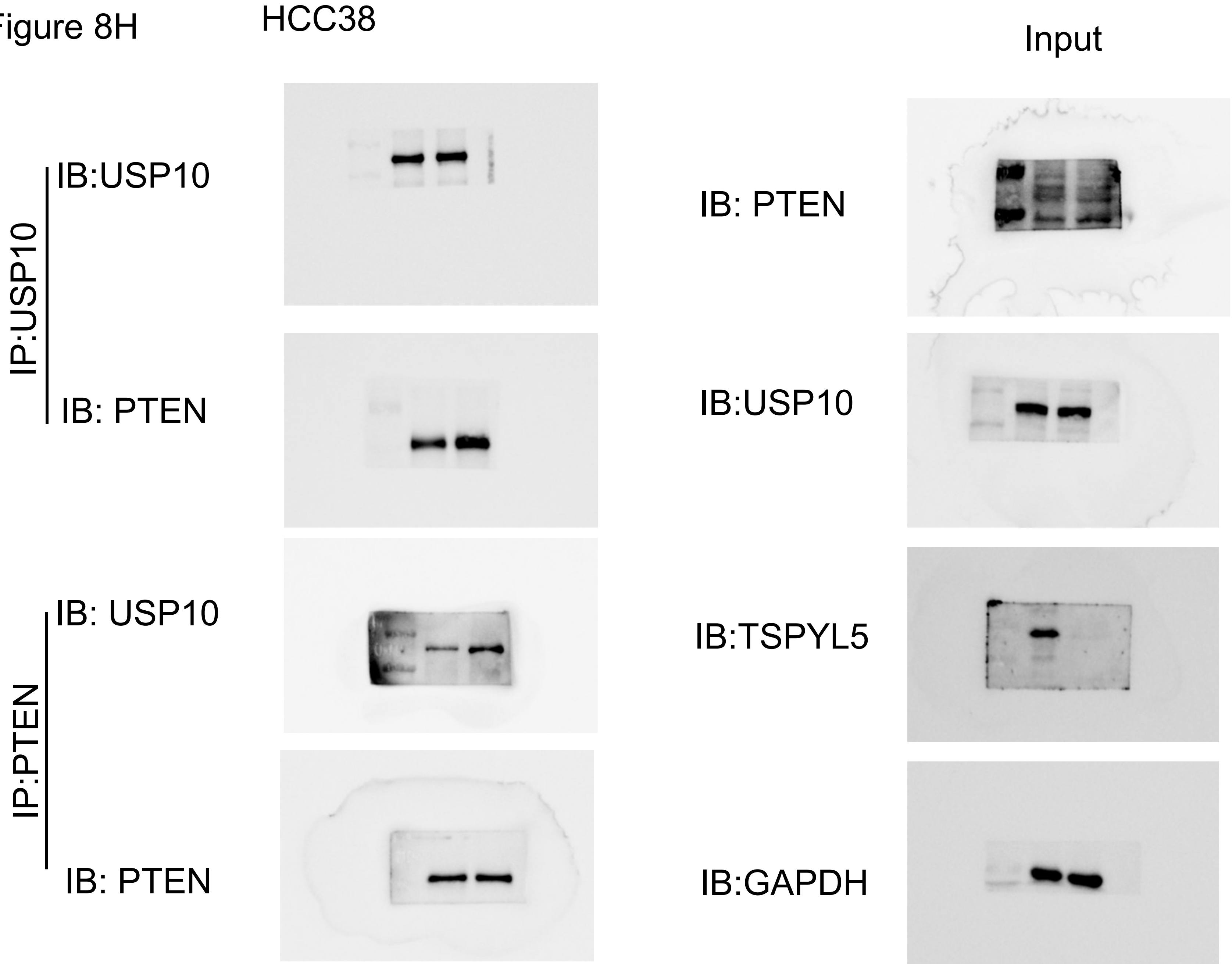
HCC38
Input
IP:USP10
IB:USP10
IB: PTEN
IP:PTEN
IB: USP10
IB: PTEN
IB: PTEN
IB:USP10
IB:TSPYL5
IB:GAPDH

Figure 8J

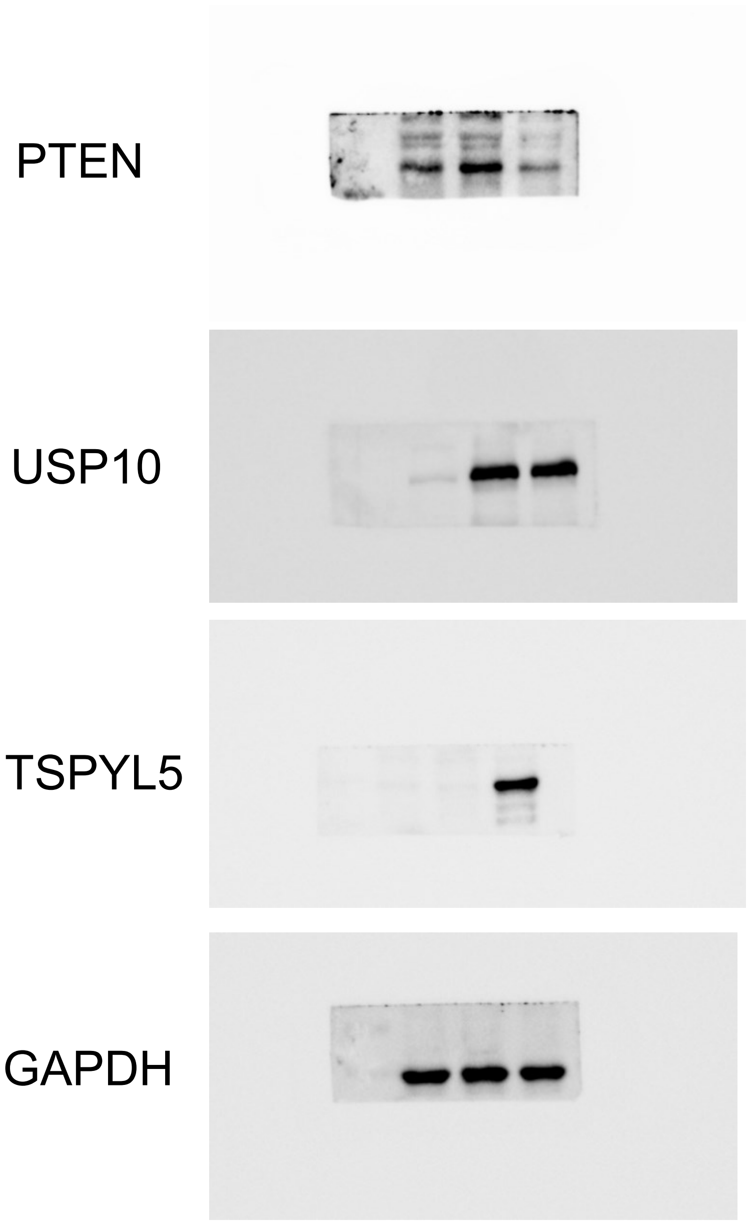

Figure 8K

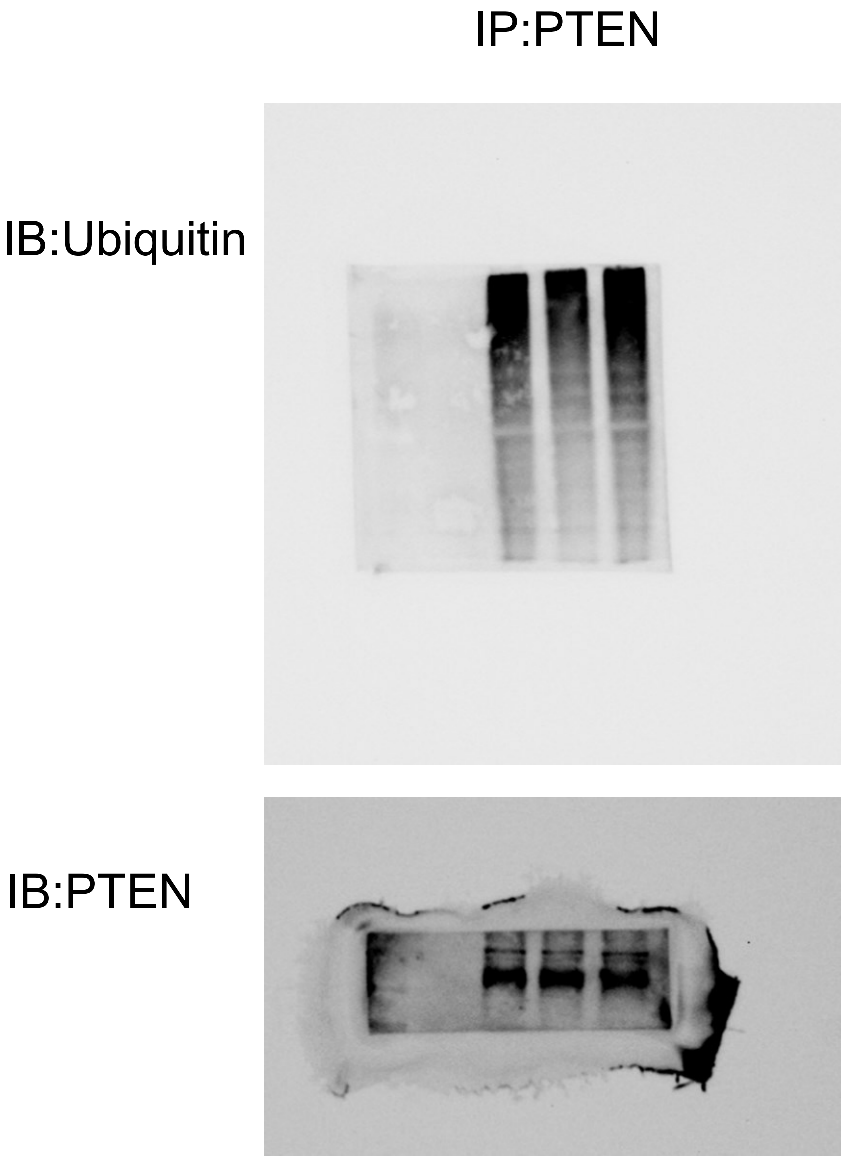

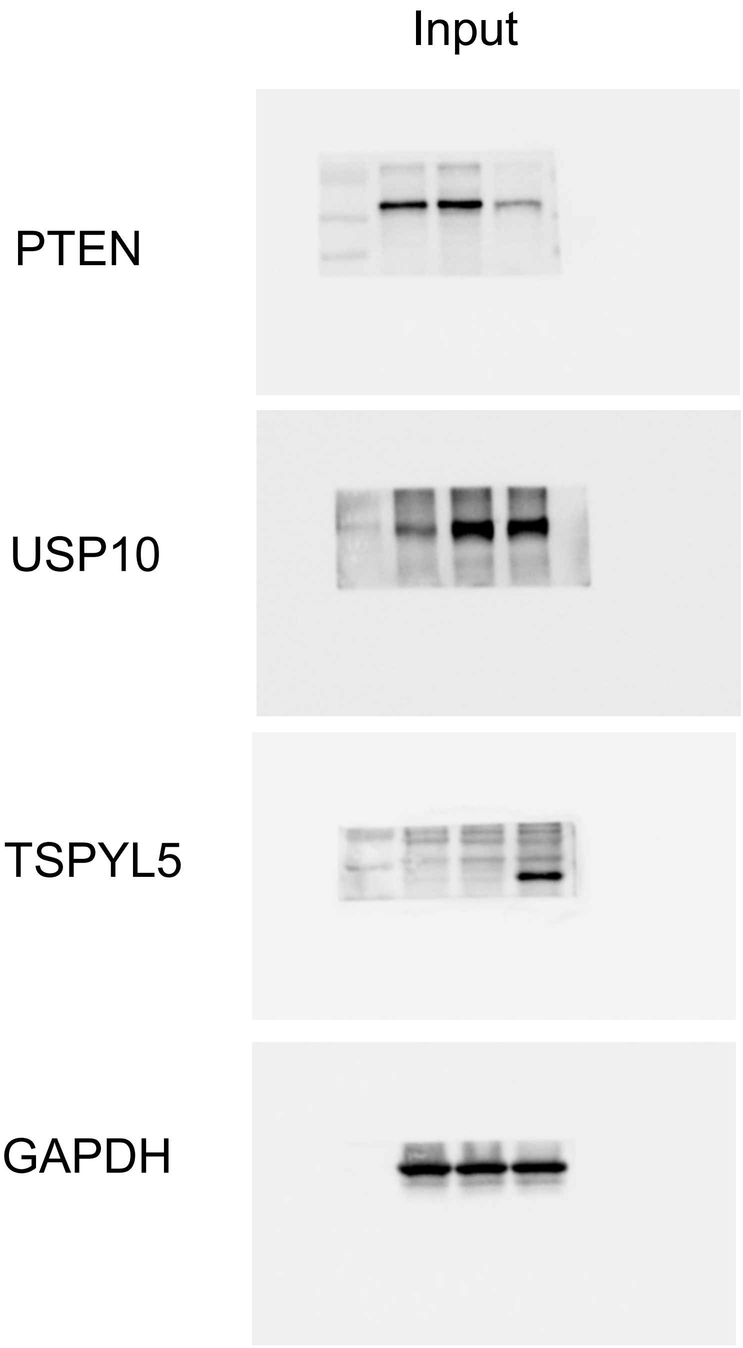

Figure 8L

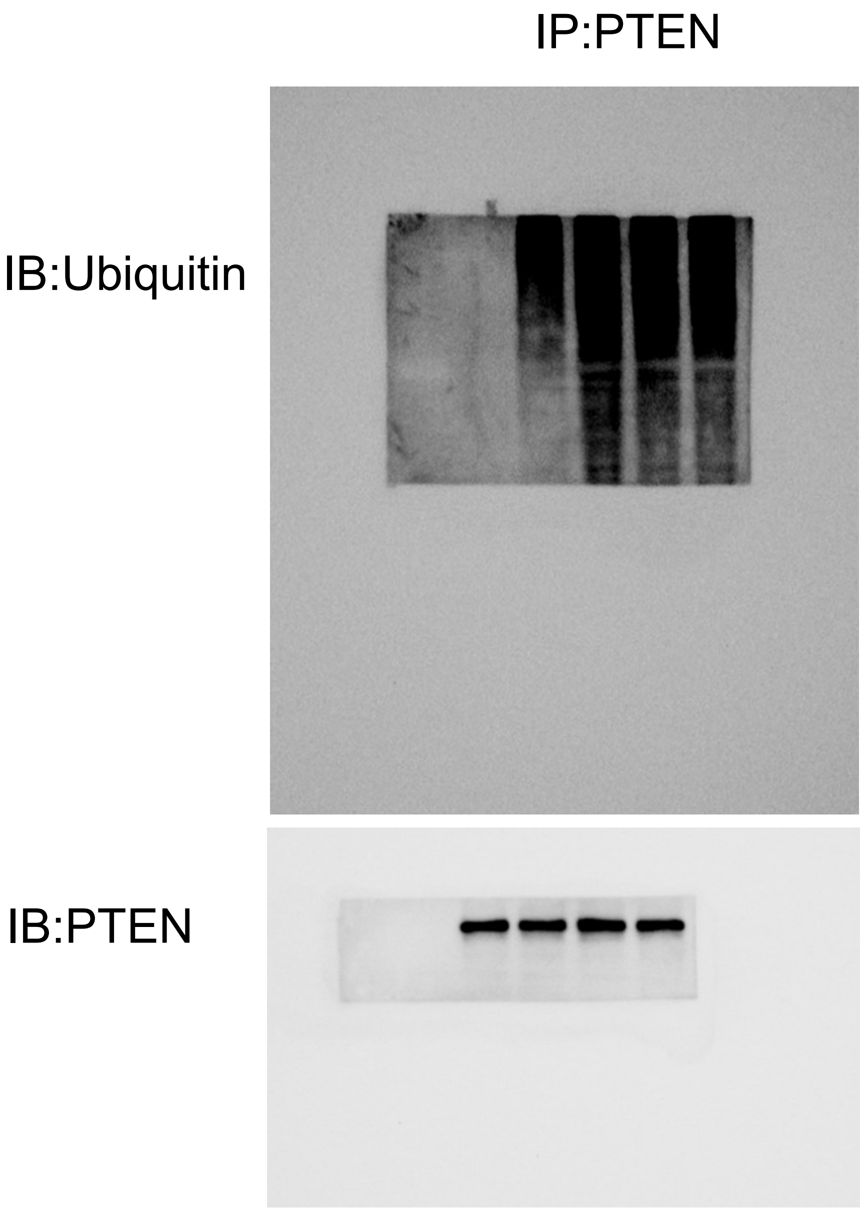

Figure 8L Input

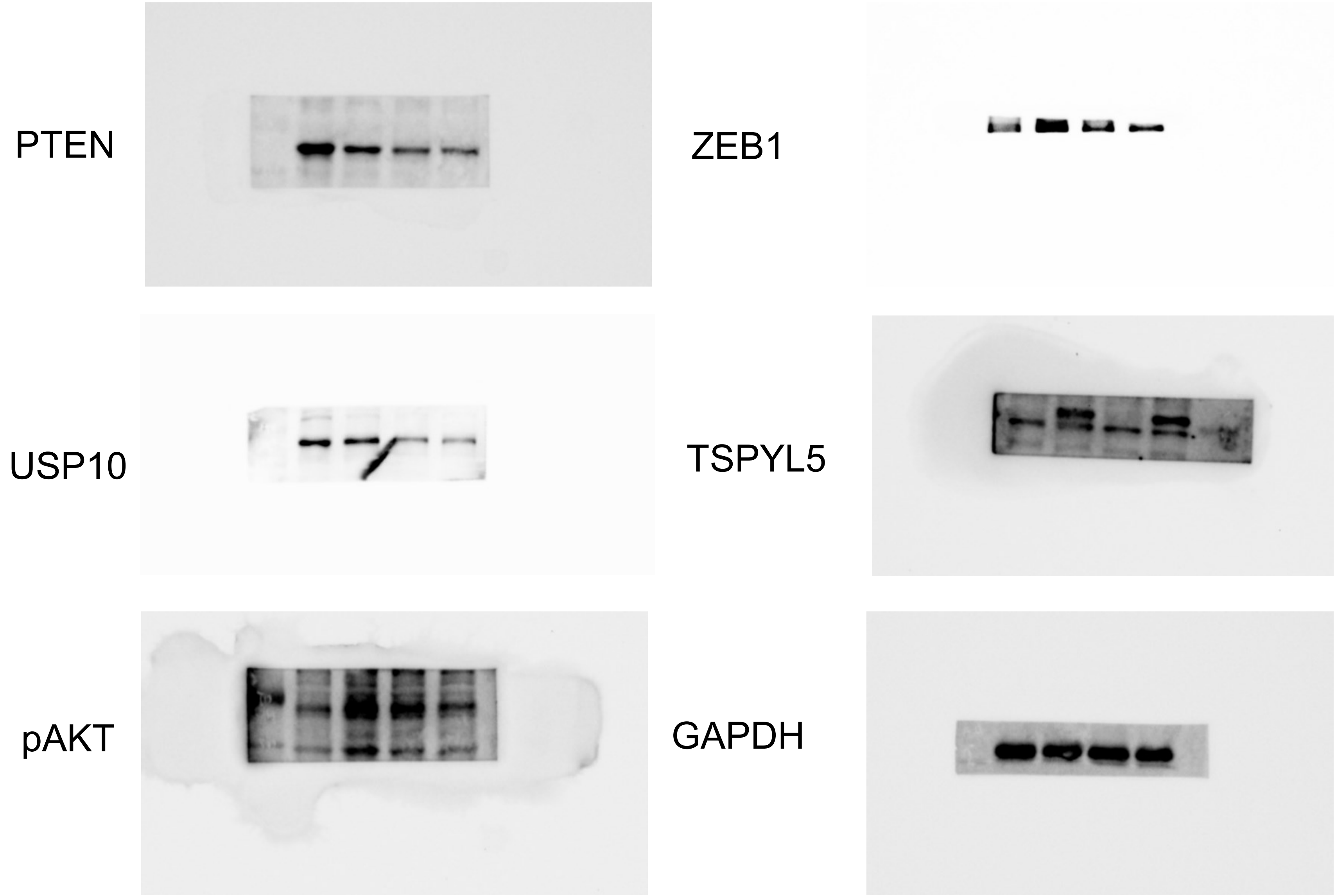

Figure 8N

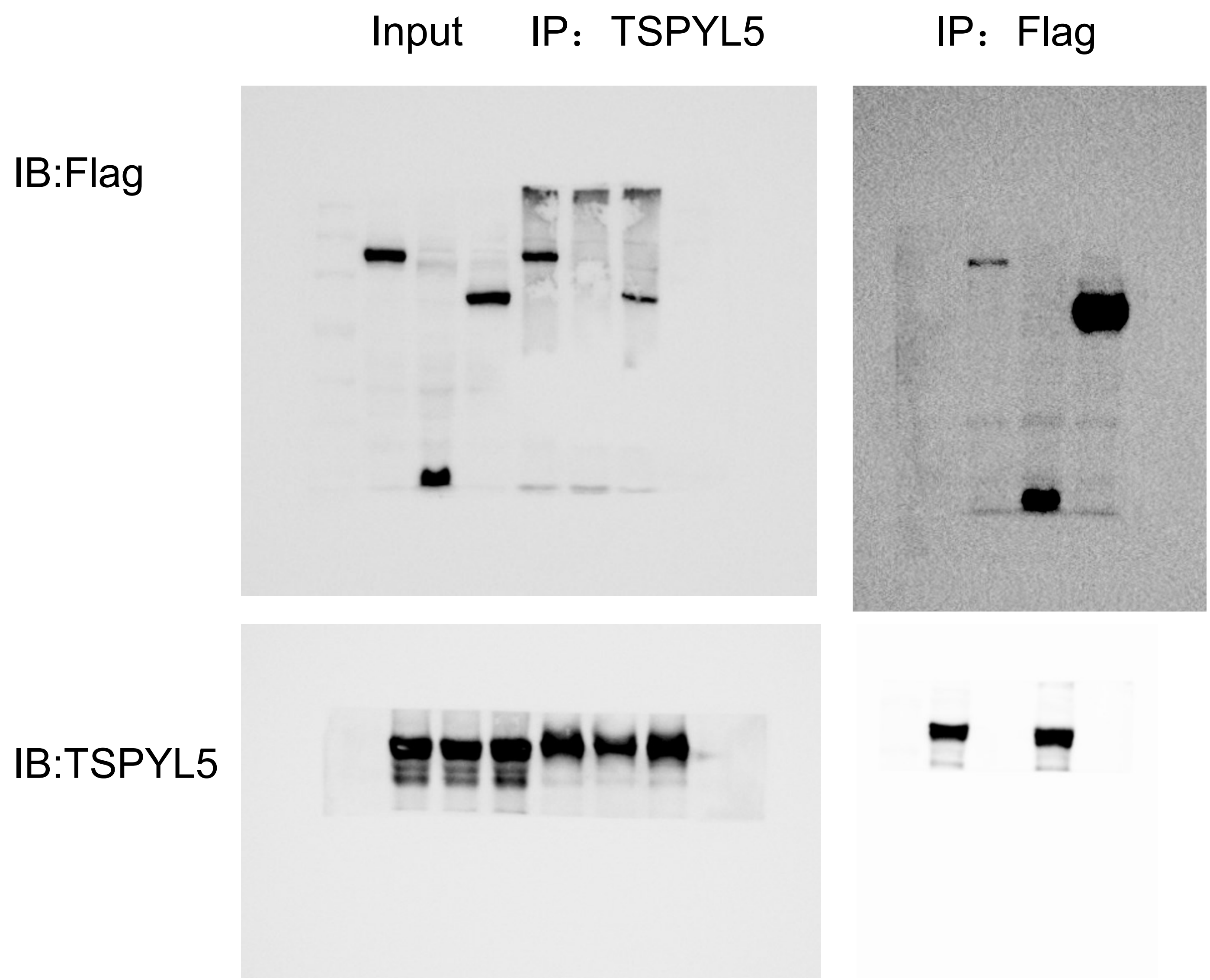

Figure 8O

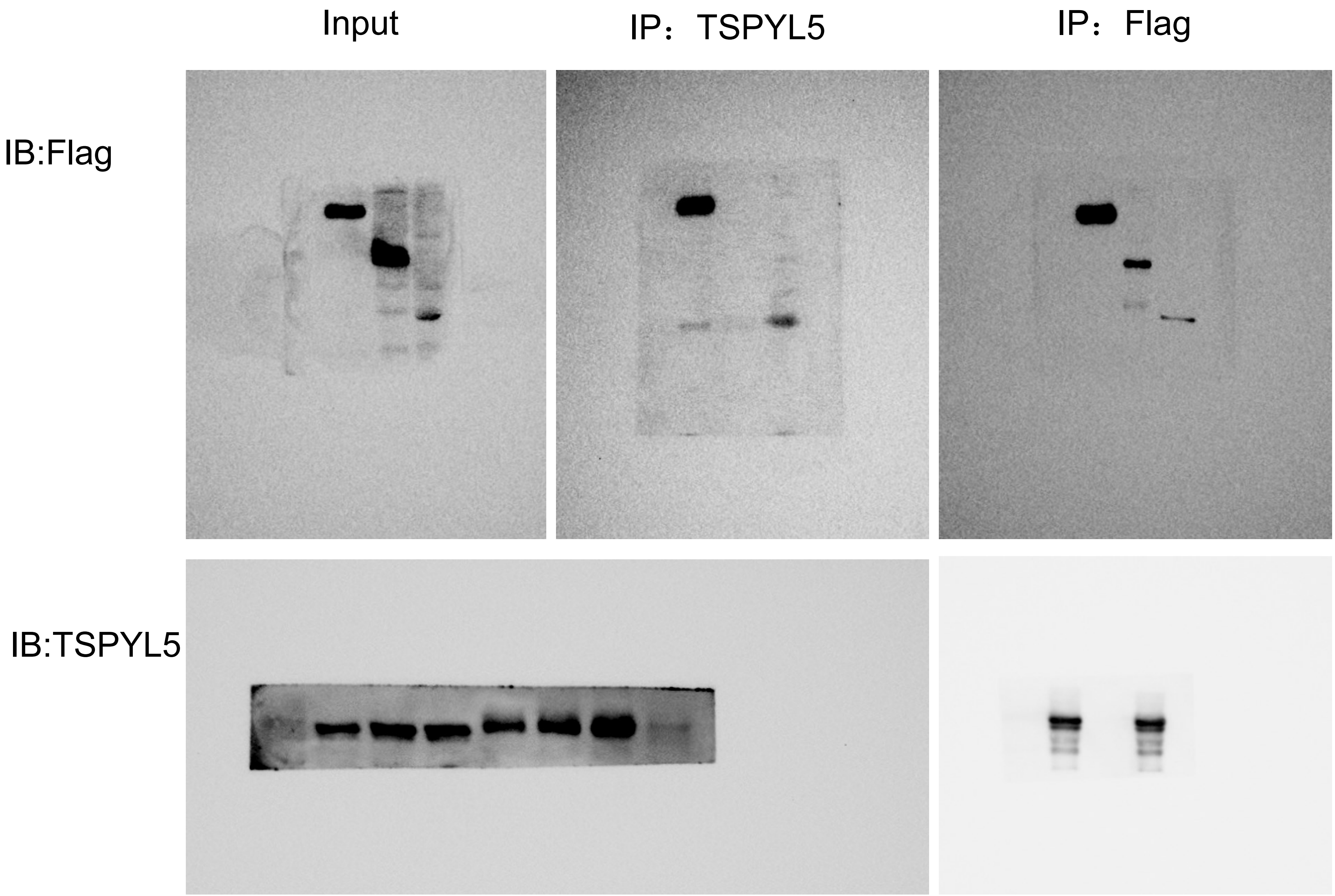

Figure 8P

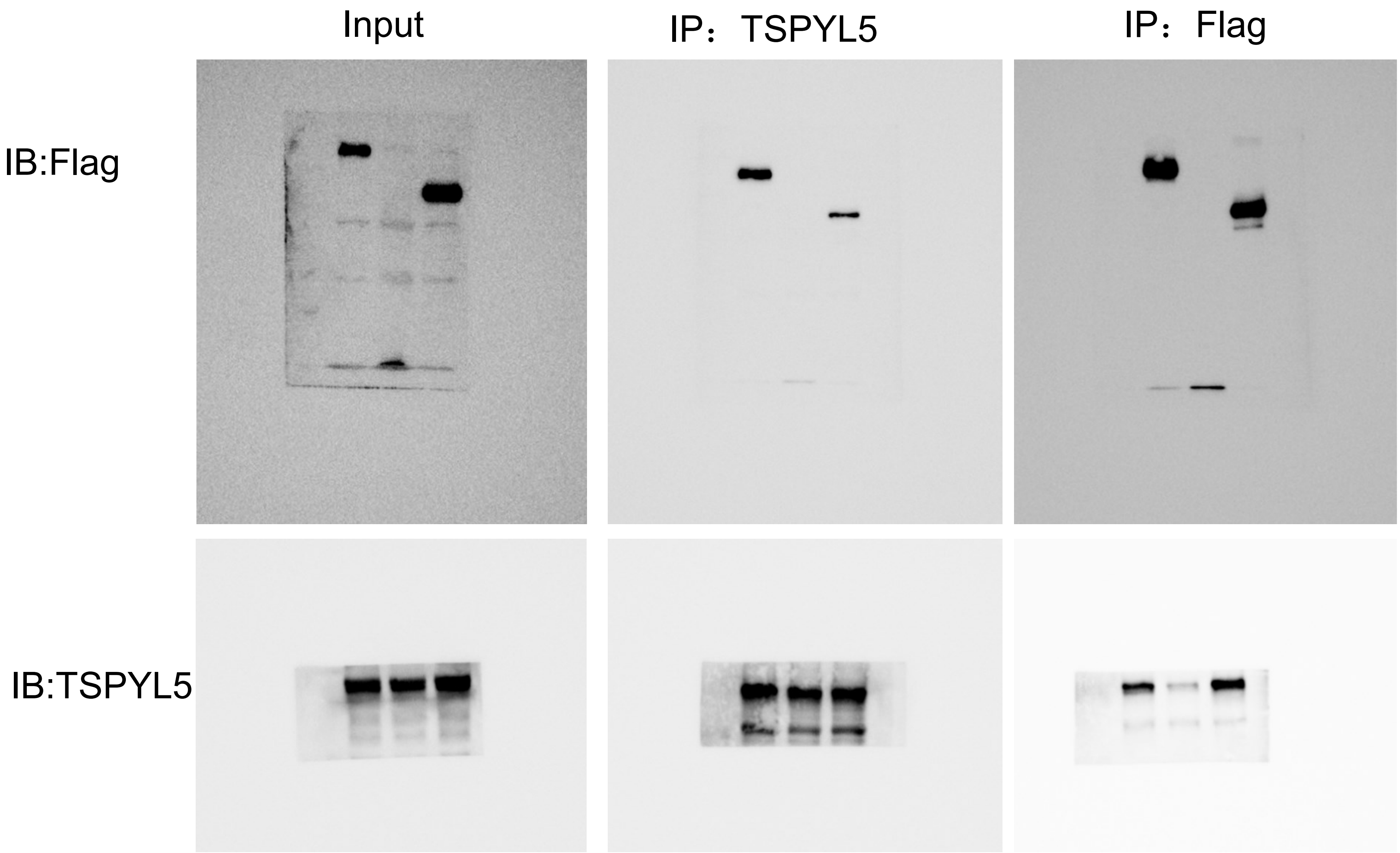

Figure 8Q

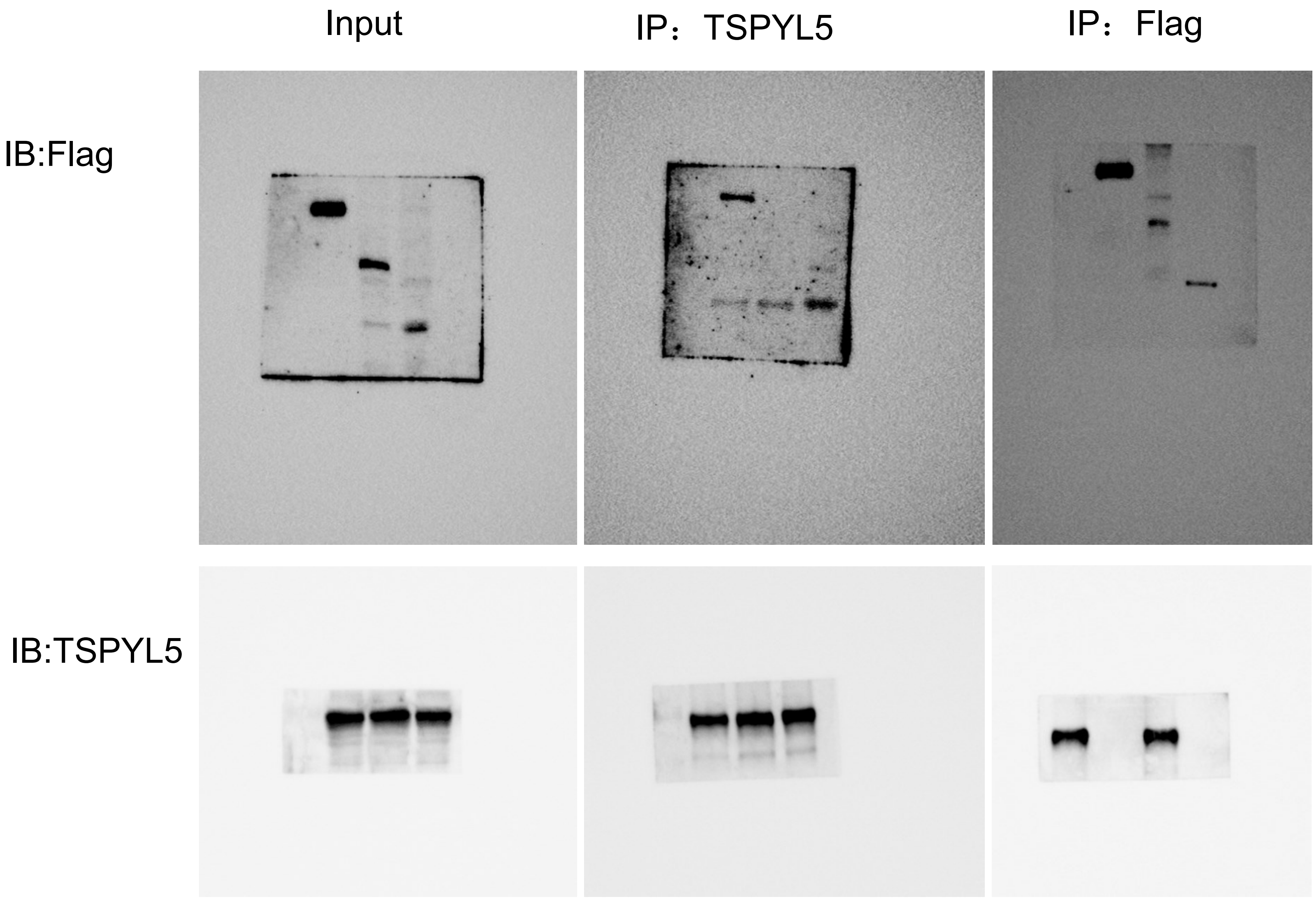

Figure S10B

GSK3β

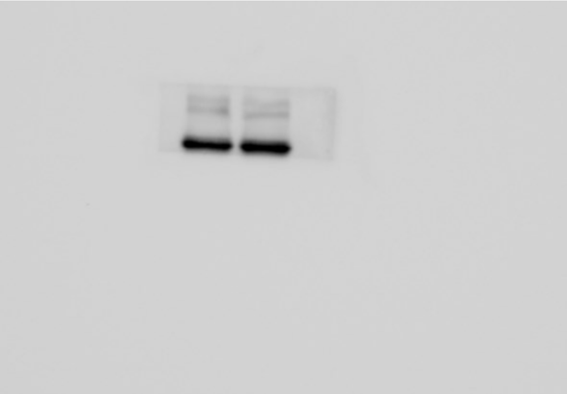

TSPYL5

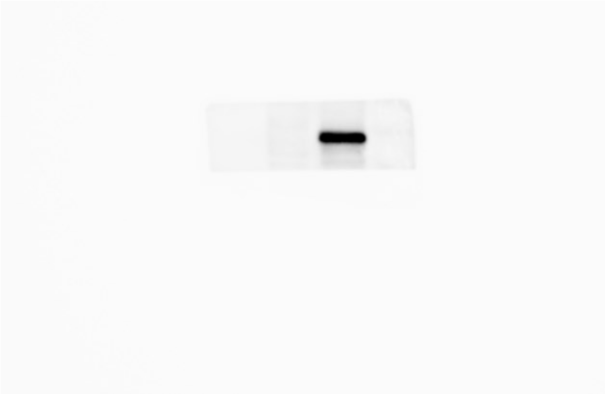

β-catenin

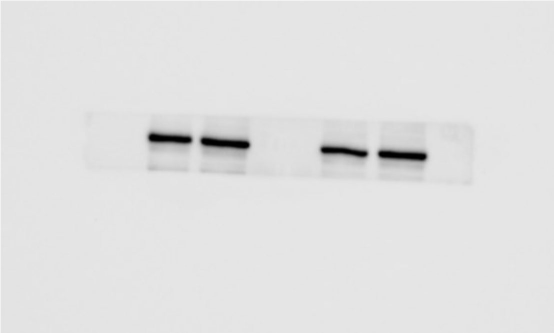

GAPDH

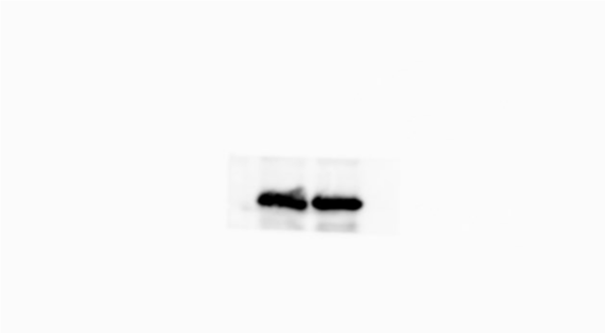

Figure S10B

IL6

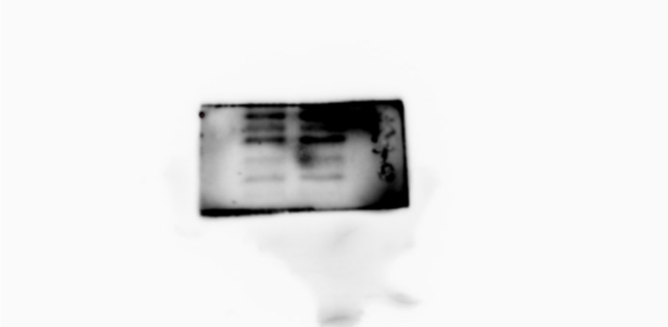

TSPYL5

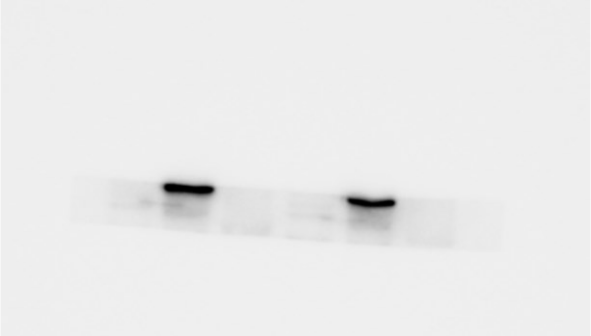

STAT3

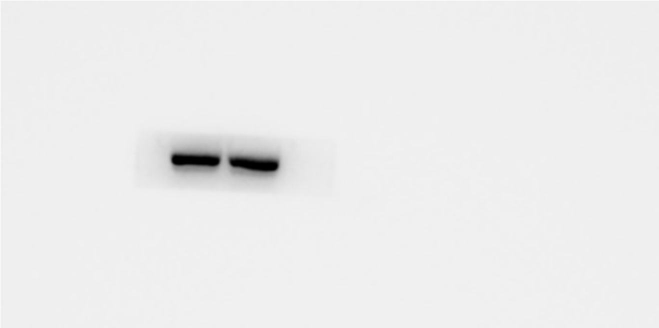

GAPDH

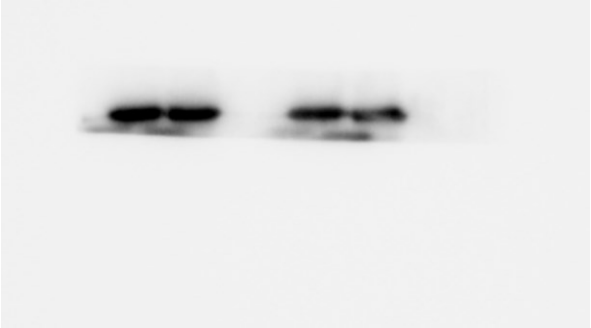

pSTAT3

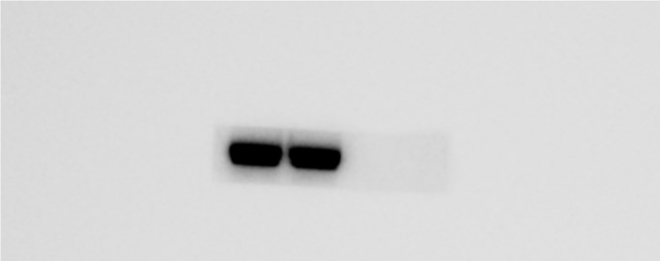

Figure S10B

SMAD2

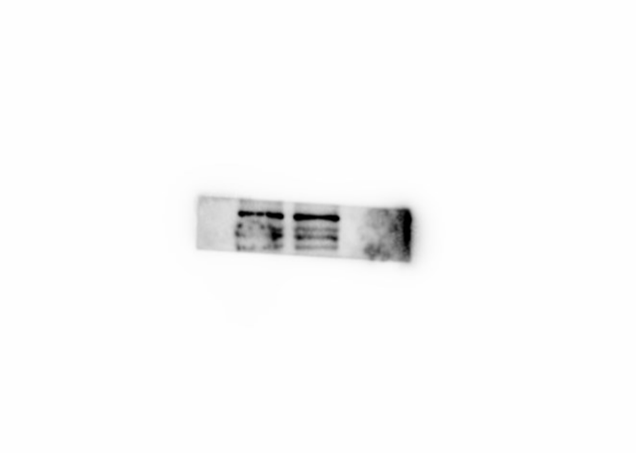

TSPYL5

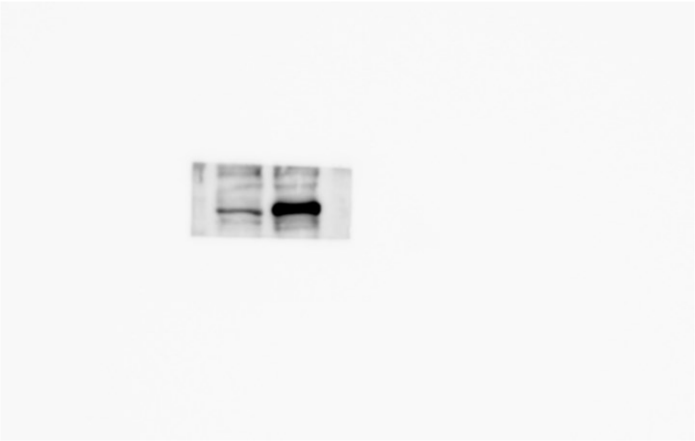

SMAD4

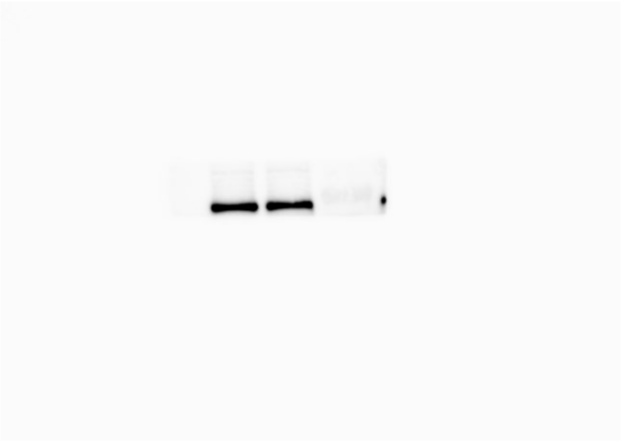

GAPDH

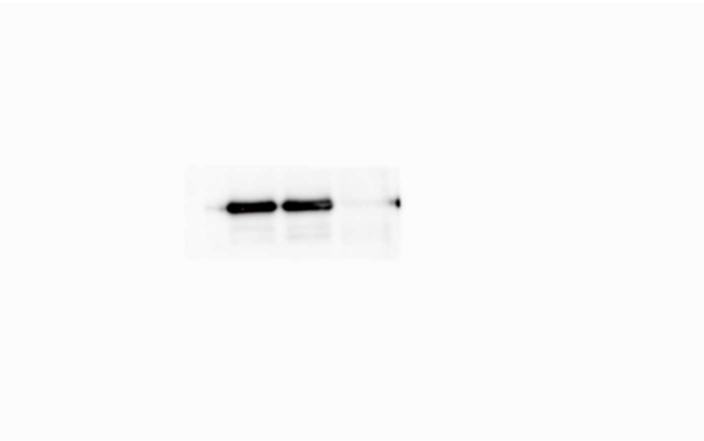

Figure S10B

ERK

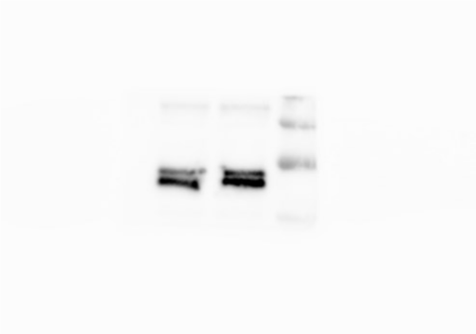

pERK

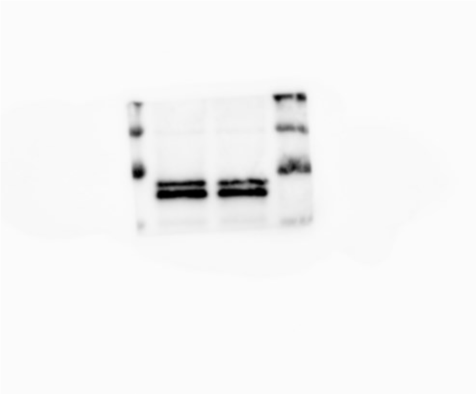

C-JUN

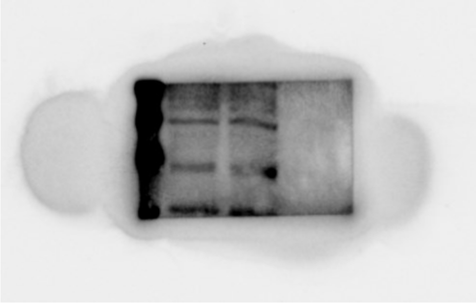

TSPYL5

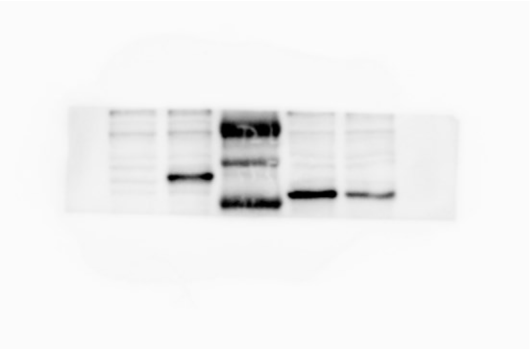

GAPDH

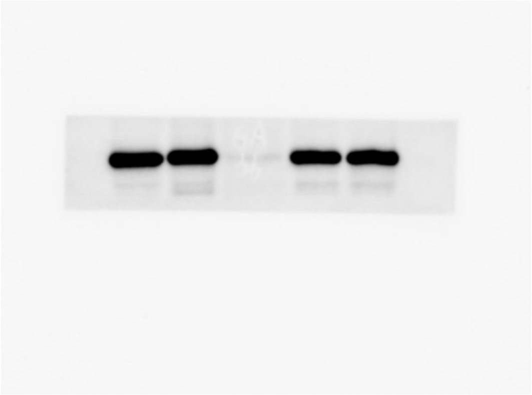

Figure S13A LY294002

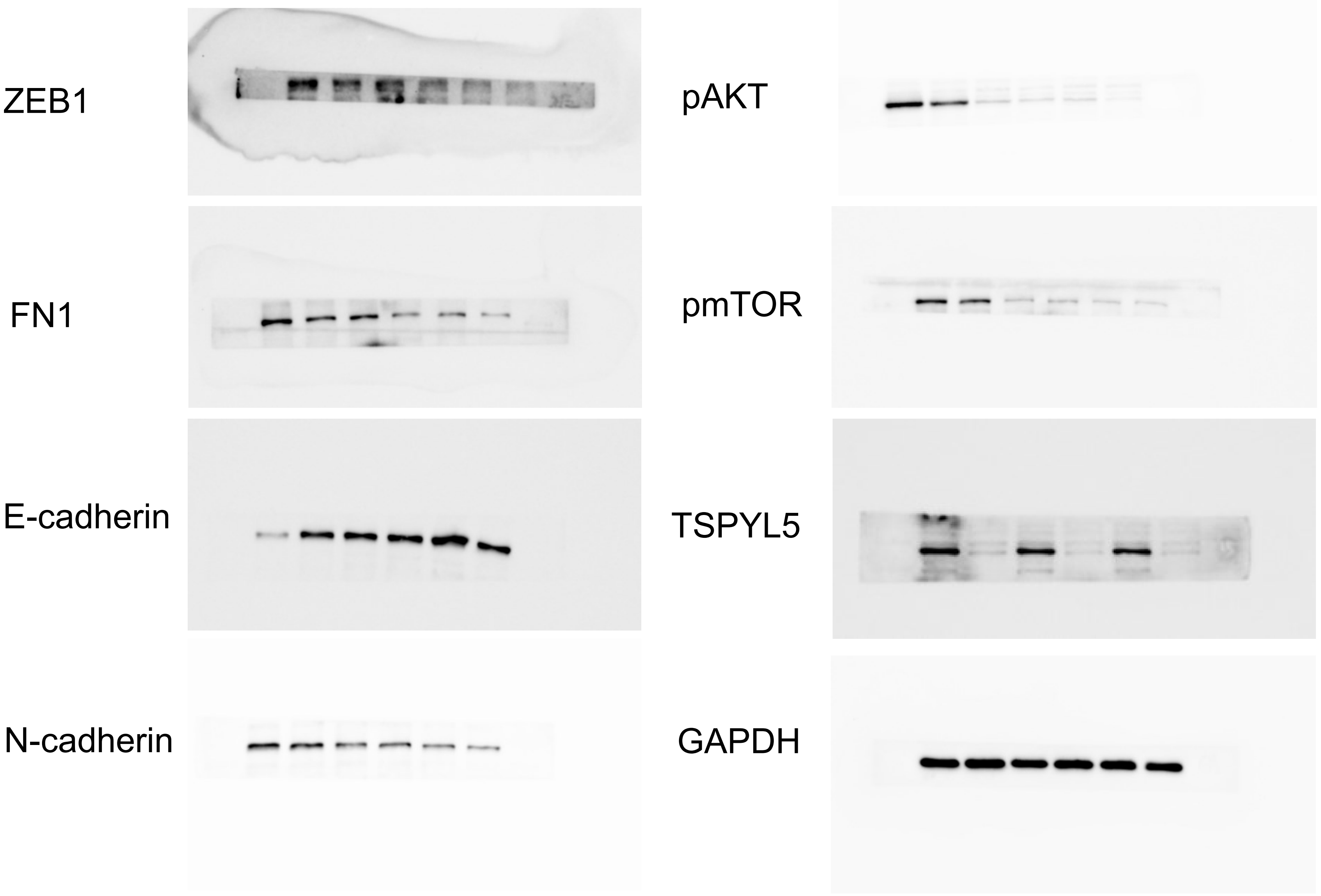

Figure S13B

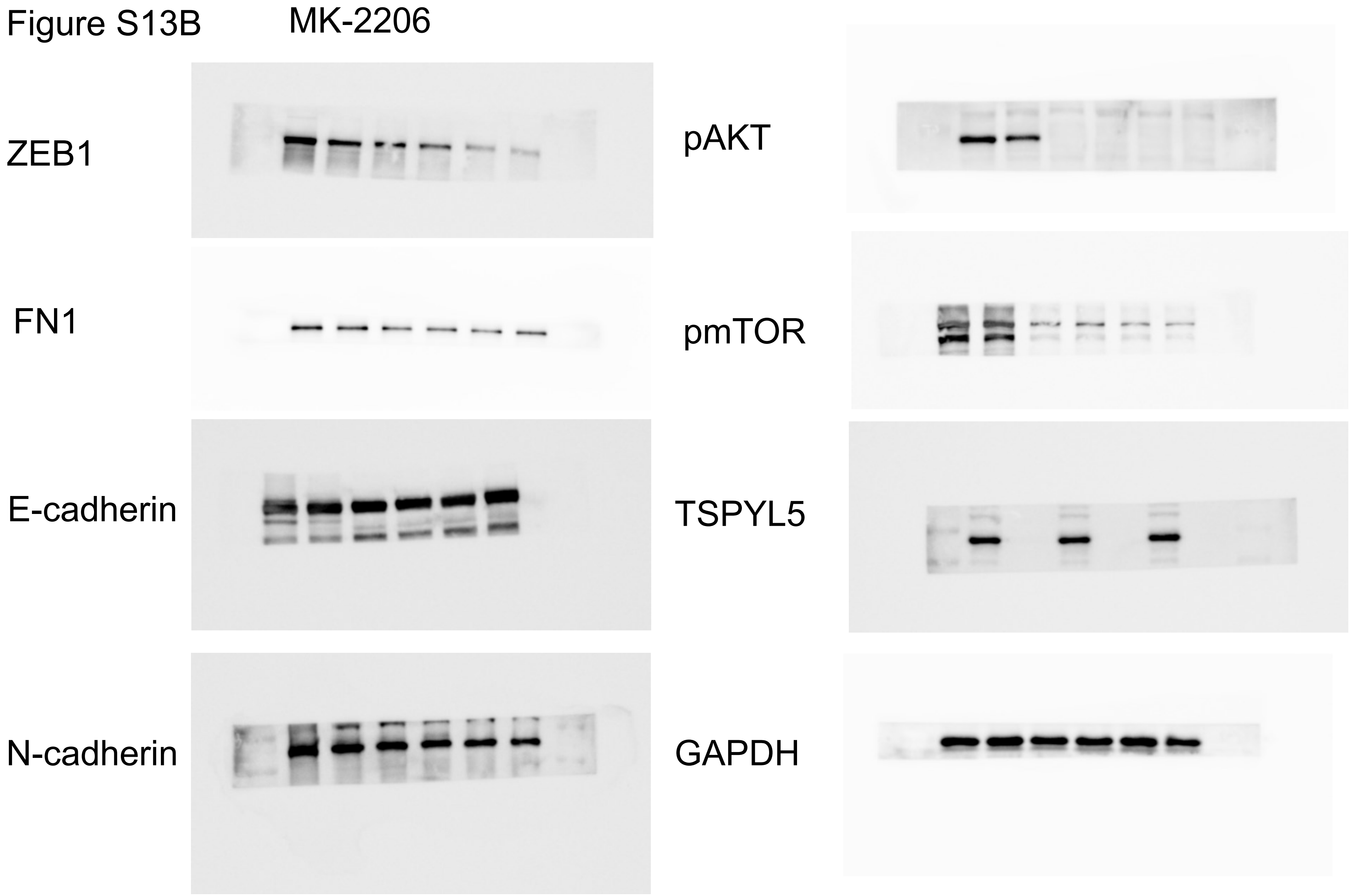
MK-2206
ZEB1
FN1
E-cadherin
N-cadherin
pAKT
pmTOR
TSPYL5
GAPDH

Figure S13C Rapamycin

ZEB1

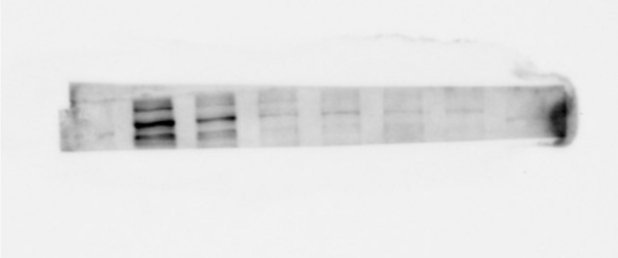

FN1

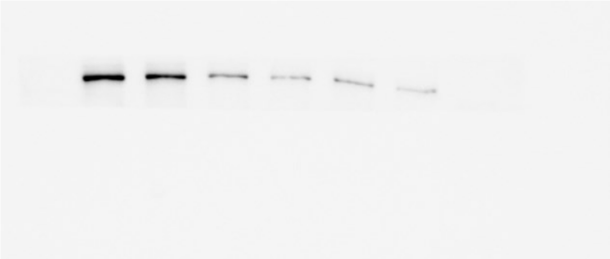

E-cadherin

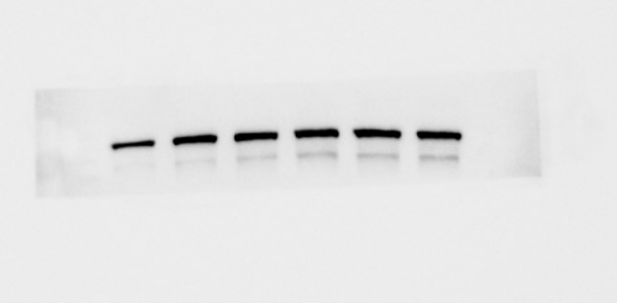

N-cadherin

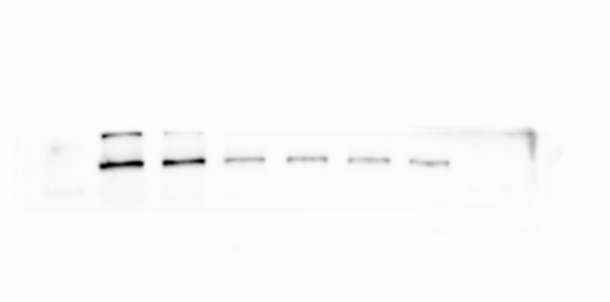

pmTOR

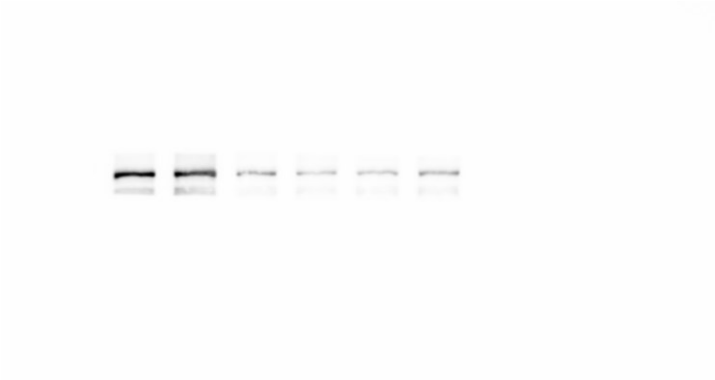

TSPYL5

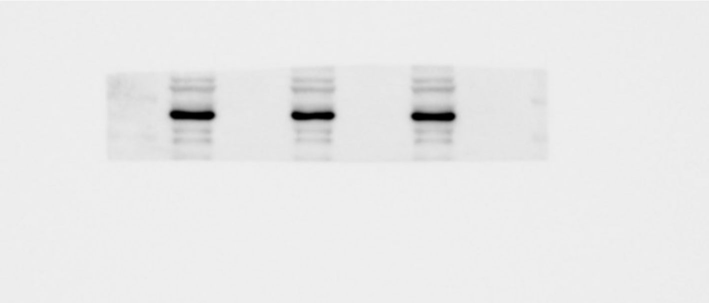

GAPDH

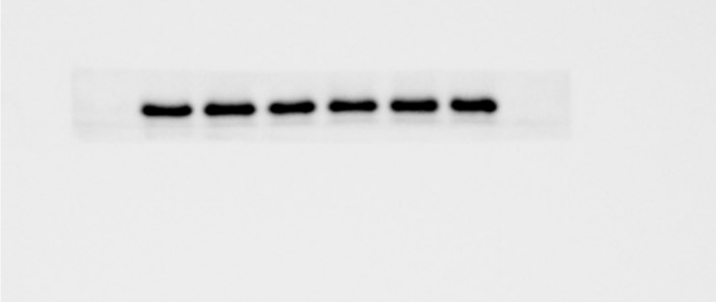

Figure S15B

PTEN

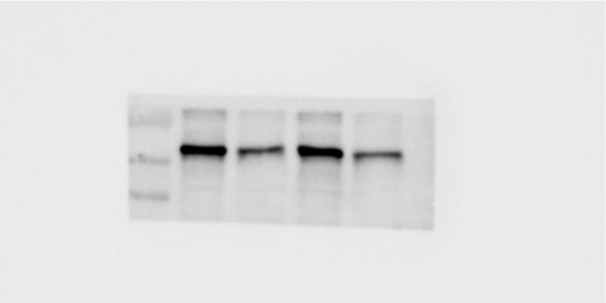

TSPYL5

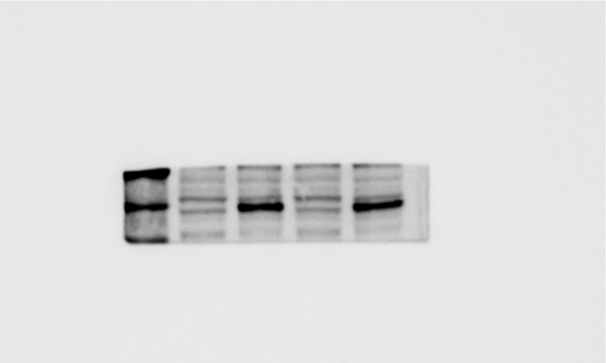

GAPDH

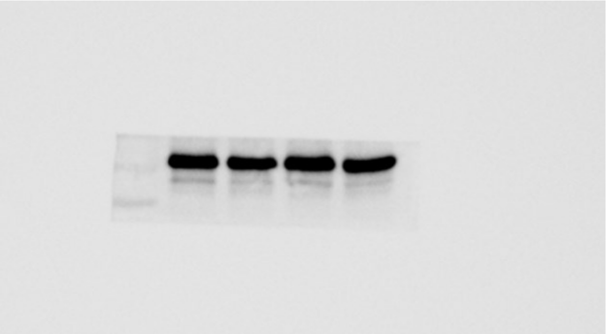

Figure S15D

PTEN

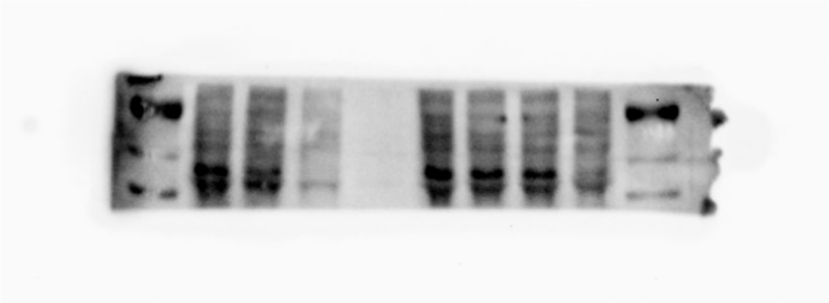

TSPYL5

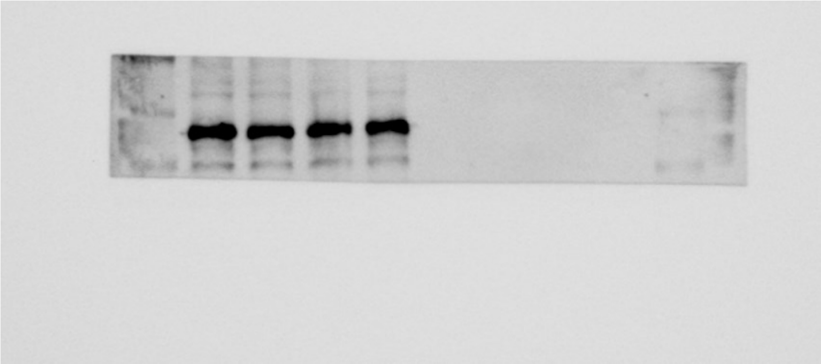

GAPDH

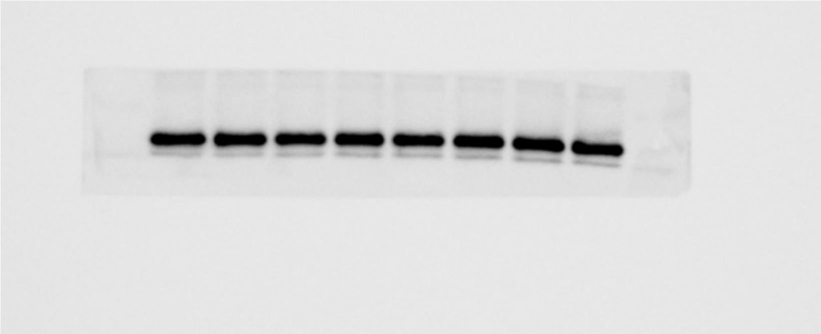

Figure S15E MDA-MB-231

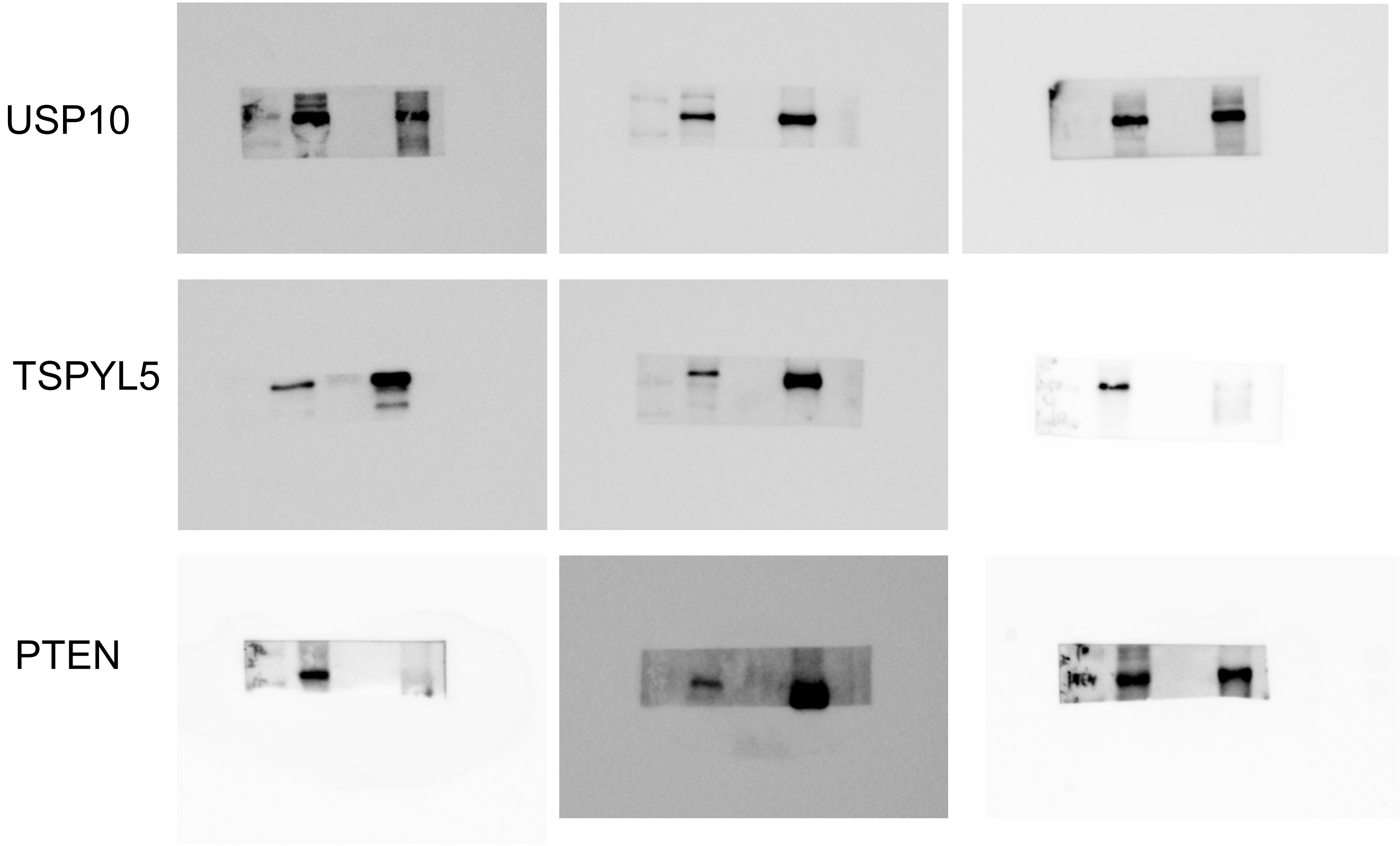

Figure S15F HCC38

USP10

TSPYL5

PTEN

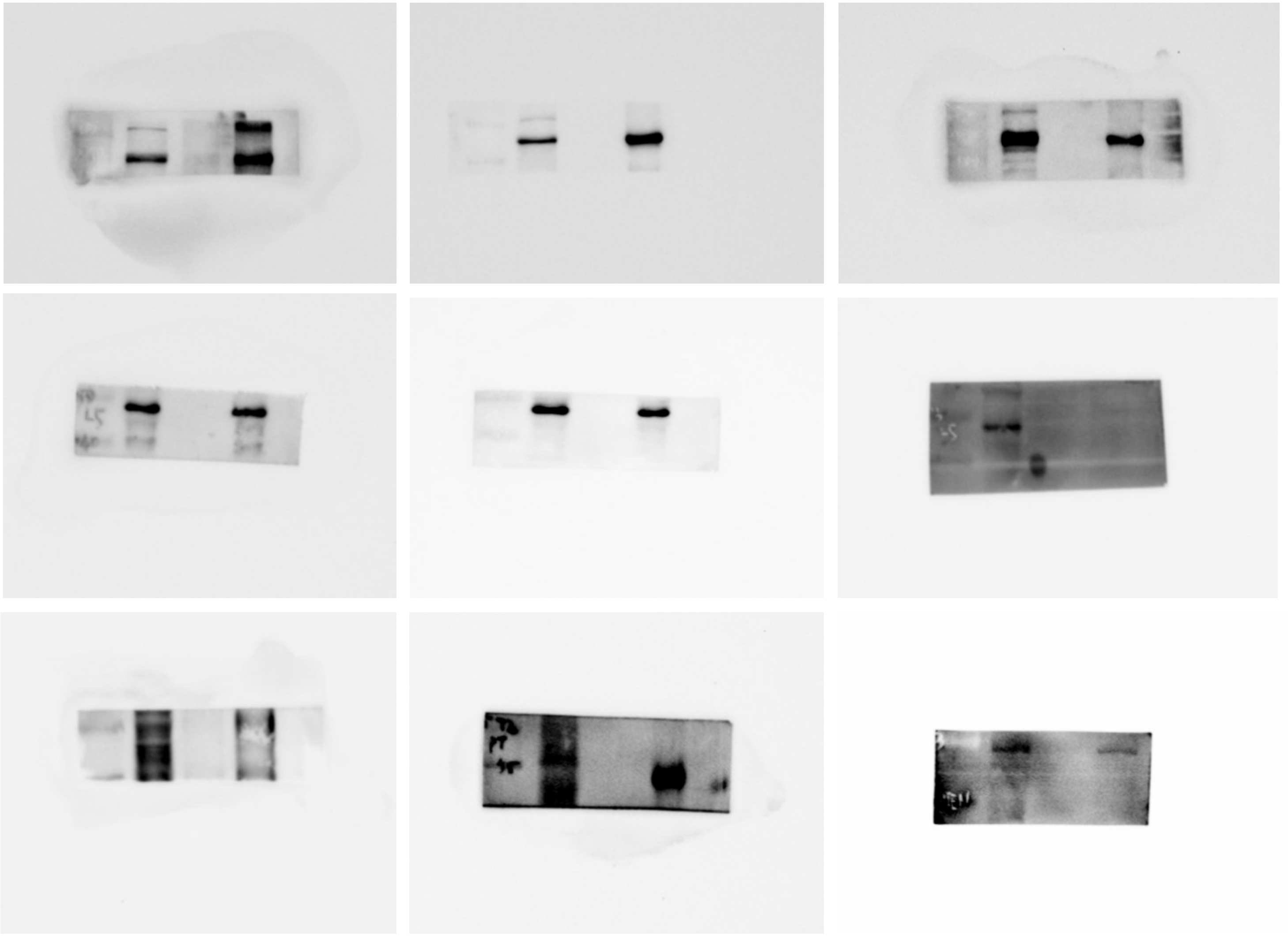

Supplement: Supplementary file 2 — Supporting File 2: advs75960‐sup‐0002‐Data.pdf. [file ADVS-9999-e20273-s001.pdf]
